# Supplementary material for: Ni-catalyzed hydroaminoalkylation of alkynes with amines
Source: Nat Commun. 2021 Jun 21;12:3800. doi: 10.1038/s41467-021-24032-9 (PMC8217523; doi:10.1038/s41467-021-24032-9)
Supplement: Supplementary file 1 — Supplementary information [file 41467_2021_24032_MOESM1_ESM.pdf]

Supplementary Information

Ni-catalyzed hydroaminoalkylation of alkynes with amines

Yao et al.

Supplementary Methods

General information

All reactions and manipulations that are sensitive to moisture or air were performed in an argon-filled glove box or using standard Schlenk techniques. Unless otherwise noted, commercially available reagents were received from commercial sources without further purification. Anhydrous tetrahydrofuran, and toluene were distilled from sodium benzophenone ketyl under nitrogen before use. The other solvents were all purified according to the standard procedures. Melting points were measured on X-4B microscope melting point apparatus and uncorrected. NMR spectra were recorded on Bruker AV 400 spectrometer at 400 MHz (^{1}H NMR), 100 MHz (^{13}C NMR). Proton and carbon chemical shifts are reported relative to the solvent used as an internal reference ($CDCl_3$: δ_H = 7.26 ppm; δ_C = 77.16 ppm). All coupling constants (*J* values) were reported in Hertz (Hz). High resolution mass spectra (HRMS) were recorded on an Agilent6520 Q-TOF LC/MS with Electron Spray Ionization (ESI) resource. All TPS-protected amines were prepared according to the reported method.[1]

Supplementary Note 1

Preparation of amines

General procedure[1]**:** To a solution of benzylamine (5 mmol, 1 equiv) and triethylamine (7.5 mmol, 1.5 equiv) in CH_2Cl_2 (25 mL) was added 2,4,6-triisopropylbenzene-1-sulfonyl chloride (5 mmol, 1 equiv) portionwise. The reaction was allowed to stir at room temperature for 3 hours before being quenched by saturated $NaHCO_3$. The aqueous layer was extracted with dichloromethane, dried over anhydrous Na_2SO_4 and concentrated in vacuo. The residue was crystallized (CH_2Cl_2/hexane) to afford the pure products.

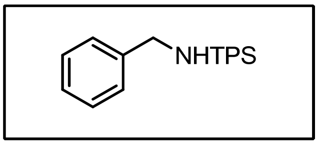

***N*-Benzyl-2,4,6-triisopropylbenzenesulfonamide (1a)**[2]
White solid (95% yield). m.p. 90-92 °C. **^{1}H NMR** (400 MHz, $CDCl_3$) δ 7.32-7.24 (m, 3H), 7.23-7.14 (m, 4H), 4.55 (t, *J* = 6.0 Hz, 1H), 4.23-4.13 (m, 2H), 4.15 (d, *J* = 6.4 Hz, 2H), 2.99-2.84 (m, 1H), 1.27 (d, *J* = 6.0 Hz, 6H), 1.26 (d, *J* = 6.4 Hz, 12H). **^{13}C NMR** (100 MHz, $CDCl_3$) δ 152.9, 150.4, 136.6, 132.4, 128.7, 128.1, 128.0, 123.9, 47.0, 34.2, 29.8, 25.0, 23.7.

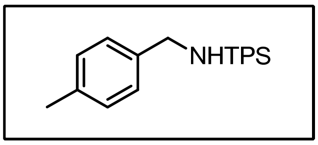

2,4,6-Triisopropyl-*N*-(4-methylbenzyl)benzenesulfonamide (1b)
White solid (83% yield). m.p. 97-98 °C. **^{1}H NMR** (400 MHz, $CDCl_3$) δ 7.18 (s, 2H), 7.09 (s, 4H), 4.48 (t, *J* = 6.0 Hz, 1H), 4.24-4.13 (m, 2H), 4.10 (d, *J* = 6.0 Hz, 2H), 2.98-2.85 (m, 1H), 2.31 (s, 3H), 1.27 (d, *J* = 6.0 Hz, 6H), 1.26 (d, *J* = 6.8 Hz, 12H). **^{13}C NMR** (100 MHz, $CDCl_3$)

δ 152.9, 150.5, 137.8, 133.5, 132.3, 129.5, 128.2, 123.9, 46.9, 34.3, 29.8, 25.0, 23.7, 21.2. **HRMS** (ESI) calcd. for $C_{23}H_{34}NO_2S$ ($[M+H]^+$) 388.2305, Found 388.2311.

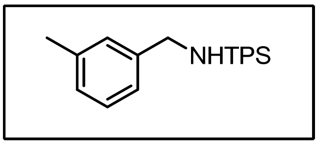

2,4,6-Triisopropyl-*N*-(3-methylbenzyl)benzenesulfonamide (1c)
White solid (85% yield). m.p. 90-91 °C. **^{1}H NMR** (400 MHz, $CDCl_3$) δ 7.18 (s, 2H), 7.16 (t, *J* = 7.2 Hz, 1H), 7.06 (d, *J* = 7.6 Hz, 1H), 7.00 (s, 1H), 6.99 (d, *J* = 8.8 Hz, 1H), 4.52 (t, *J* = 6.0 Hz, 1H), 4.23-4.13 (m, 2H), 4.12 (d, *J* = 6.0 Hz, 2H), 2.99-2.86 (m, 1H), 2.28 (s, 3H), 1.28 (d, *J* = 5.6 Hz, 6H), 1.26 (d, *J* = 6.8 Hz, 12H). **^{13}C NMR** (100 MHz, $CDCl_3$) δ 152.9, 150.4, 138.5, 136.5, 132.5, 128.9, 128.8, 128.7, 125.2, 123.9, 47.1, 34.3, 29.8, 25.0, 23.7, 21.4. **HRMS** (ESI) calcd. for $C_{23}H_{34}NO_2S$ ($[M+H]^+$) 388.2305, Found 388.2311.

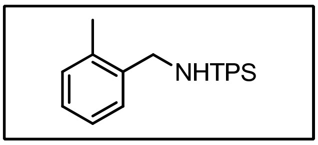

2,4,6-Triisopropyl-*N*-(2-methylbenzyl)benzenesulfonamide (1d)
White solid (70% yield). m.p. 95-96 °C. **^{1}H NMR** (400 MHz, $CDCl_3$) δ 7.22-7.17 (m, 1H), 7.19 (s, 2H), 7.17-7.13 (m, 1H), 7.13-7.07 (m, 2H), 4.35 (t, *J* = 6.0 Hz, 1H), 4.24-4.14 (m, 2H), 4.13 (d, *J* = 6.0 Hz, 2H), 2.99-2.86 (m, 1H), 2.28 (s, 3H), 1.27 (d, *J* = 6.8, 6H), 1.26 (d, *J* = 6.8, 12H). **^{13}C NMR** (100 MHz, $CDCl_3$) δ 153.0, 150.5, 136.9, 134.3, 132.1, 130.7, 128.9, 128.3, 126.3, 124.0, 45.0, 34.3, 29.8, 25.0, 23.7, 18.9. **HRMS** (ESI) calcd. for $C_{23}H_{34}NO_2S$ ($[M+H]^+$) 388.2305, Found 388.2310.

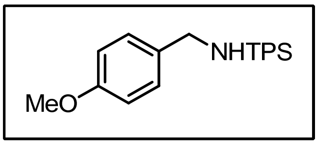

2,4,6-Triisopropyl-*N*-(4-methoxybenzyl)benzenesulfonamide (1e)[3]
White solid (81% yield). m.p. 99-100 °C. **^{1}H NMR** (400 MHz, $CDCl_3$) δ 7.18 (s, 2H), 7.11 (d, *J* = 8.8 Hz, 2H), 6.80 (d, *J* = 8.8 Hz, 2H), 4.49 (t, *J* = 6.0 Hz, 1H), 4.23-4.13 (m, 2H), 4.08 (d, *J* = 6.0 Hz, 2H), 3.77 (s, 3H), 2.99-2.85 (m, 1H), 1.28 (d, *J* = 6.4 Hz, 6H), 1.26 (d, *J* = 6.4 Hz, 12H). **^{13}C NMR** (100 MHz, $CDCl_3$) δ 159.4, 152.9, 150.5, 132.4, 129.6, 128.6, 123.9, 114.2, 55.4, 46.6, 34.3, 29.8, 25.0, 23.7.

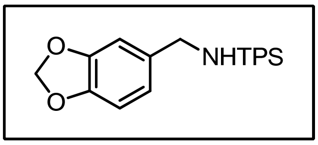

***N*-(Benzo[d][1,3]dioxol-5-ylmethyl)-2,4,6-triisopropylbenzenesulfonamide (1f)**
White solid (83% yield). m.p. 126-127 °C. **^{1}H NMR** (400 MHz, $CDCl_3$) δ 7.18 (s, 2H),

6.72-6.61 (m, 3H), 5.93 (s, 2H), 4.47 (t, *J* = 6.0 Hz, 1H), 4.22-4.10 (m, 2H), 4.05 (d, *J* = 6.0 Hz, 2H), 2.98-2.84 (m, 1H), 1.27 (d, *J* = 6.8 Hz, 6H), 1.26 (d, *J* = 6.8 Hz, 12H). **^{13}C NMR** (100 MHz, $CDCl_3$) δ 153.0, 150.4, 148.0, 147.4, 132.4, 130.3, 123.9, 121.6, 108.8, 108.3, 101.2, 47.0, 34.3, 29.8, 25.0, 23.7. **HRMS** (ESI) calcd. for $C_{23}H_{32}NO_4S$ ($[M+H]^+$) 418.2047, Found 418.2050.

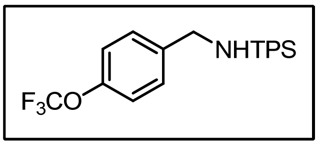

2,4,6-Triisopropyl-*N*-(4-(trifluoromethoxy)benzyl)benzenesulfonamide (1g)
White solid (85% yield). m.p. 122-123 °C. **^{1}H NMR** (400 MHz, $CDCl_3$) δ 7.23 (d, *J* = 8.8 Hz, 2H), 7.17 (s, 2H), 7.12 (d, *J* = 8.0 Hz, 2H), 4.61 (t, *J* = 6.2 Hz, 1H), 4.17 (d, *J* = 6.4 Hz, 2H), 4.21-4.06 (m, 2H), 2.98-2.84 (m, 1H), 1.26 (d, *J* = 6.8 Hz, 6H), 1.25 (d, *J* = 6.8 Hz, 12H). **^{13}C NMR** (100 MHz, $CDCl_3$) δ 153.1, 150.4, 148.8, 135.6, 132.5, 129.6, 123.9, 121.2, 120.5 (q, *J* = 255 Hz), 46.2, 34.3, 29.8, 24.9, 23.6. **HRMS** (ESI) calcd. for $C_{23}H_{31}F_3NO_3S$ ($[M+H]^+$) 458.1971, Found 458.1979.

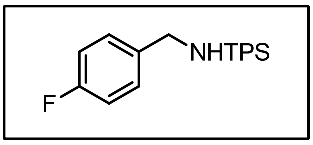

***N*-(4-Fluorobenzyl)-2,4,6-triisopropylbenzenesulfonamide (1h)**
White solid (70% yield). m.p. 126-127 °C. **^{1}H NMR** (400 MHz, $CDCl_3$) δ 7.22-7.13 (m, 2H), 7.17 (s, 2H), 6.97 (d, *J* = 8.4 Hz, 1H), 6.95 (d, *J* = 8.8 Hz, 1H), 4.53 (t, *J* = 6.0 Hz, 1H), 4.22-4.09 (m, 2H), 4.13 (d, *J* = 6.0 Hz, 1H) 2.98-2.86 (m, 1H), 1.27 (d, *J* = 6.4 Hz, 6H), 1.25 (d, *J* = 6.4 Hz, 12H). **^{13}C NMR** (100 MHz, $CDCl_3$) δ 162.4 (d, *J* = 245 Hz), 153.1, 150.4, 132.5 (d, *J* = 3.2 Hz), 132.4, 129.9 (d, *J* = 8.3 Hz), 123.9, 115.6 (d, *J* = 21.3 Hz), 46.4, 34.3, 29.8, 25.0, 23.7. **HRMS** (ESI) calcd. for $C_{22}H_{31}FNO_2S$ ($[M+H]^+$) 392.2054, Found 392.2062.

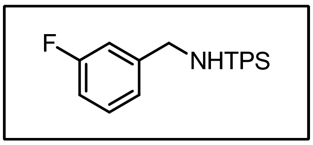

***N*-(3-Fluorobenzyl)-2,4,6-triisopropylbenzenesulfonamide (1i)**
White solid (83% yield). m.p. 113-115 °C. **^{1}H NMR** (400 MHz, $CDCl_3$) δ 7.26-7.20 (m, 1H), 7.18 (s, 2H), 6.99 (d, *J* = 7.6 Hz, 1H), 6.96-6.86 (m, 2H), 4.65 (t, *J* = 6.4 Hz, 1H), 4.23-4.08 (m, 2H), 4.16 (d, *J* = 6.4 Hz, 2H), 2.99-2.84 (m, 1H), 1.27 (d, *J* = 6.8 Hz, 6H), 1.26 (d, *J* = 6.8 Hz, 12H). **^{13}C NMR** (100 MHz, $CDCl_3$) δ 162.9 (d, *J* = 245.2 Hz), 153.1, 150.4, 139.3 (d, *J* = 7.1 Hz), 132.4, 130.2 (d, *J* = 8.1 Hz), 123.9, 123.6 (d, *J* = 2.9 Hz), 115.0 (d, *J* = 21.8 Hz), 114.8 (d, *J* = 20.8 Hz), 46.5, 46.4, 34.3, 29.8, 24.9, 23.7. **HRMS** (ESI) calcd. for $C_{22}H_{31}FNO_2S$ ($[M+H]^+$) 392.2054, Found 392.2060.

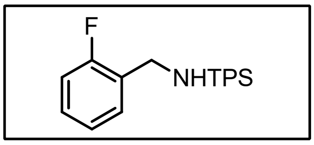

***N*-(2-Fluorobenzyl)-2,4,6-triisopropylbenzenesulfonamide (1j)**
White solid (74% yield). m.p. 102-103 °C. **^{1}H NMR** (400 MHz, $CDCl_3$) δ 7.25-7.18 (m, 2H), 7.15 (s, 2H), 7.03 (t, *J* = 7.6 Hz, 1H), 6.98 (t, *J* = 9.2 Hz, 1H), 4.68 (t, *J* = 6.4 Hz, 1H), 4.22 (d, *J* = 6.4 Hz, 2H), 4.19-4.09 (m, 2H), 2.96-2.83 (m, 1H), 1.30-1.19 (m, 18H). **^{13}C NMR** (100 MHz, $CDCl_3$) δ 160.9 (d, *J* = 244.9 Hz), 153.0, 150.4, 132.4, 130.3 (d, *J* = 3.8 Hz), 129.8 (d, *J* = 8.1 Hz), 124.4 (d, *J* = 3.3 Hz), 124.0, 123.9, 115.5 (d, *J* = 20.9 Hz), 41.0, 40.9, 34.3, 29.9, 24.9, 23.7. **HRMS** (ESI) calcd. for $C_{22}H_{31}FNO_2S$ ($[M+H]^+$) 392.2054, Found 392.2059.

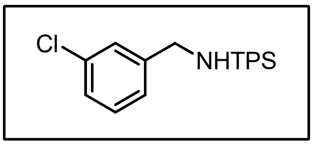

***N*-(3-Chlorobenzyl)-2,4,6-triisopropylbenzenesulfonamide (1k)**
White solid (77% yield). m.p. 112-113 °C. **^{1}H NMR** (400 MHz, $CDCl_3$) δ 7.25-7.19 (m, 2H), 7.19-7.13 (m, 3H), 7.12-7.05 (m, 1H), 4.60 (t, *J* = 6.0 Hz, 1H), 4.22-4.06 (m, 2H), 4.15 (d, *J* = 6.4 Hz, 1H), 2.98-2.83 (m, 1H), 1.36-1.16 (m, 18H). **^{13}C NMR** (100 MHz, $CDCl_3$) δ 153.1, 150.4, 138.8, 134.6, 132.4, 130.0, 128.2, 128.1, 126.2, 124.0, 46.5, 34.3, 29.8, 25.0, 23.7. **HRMS** (ESI) calcd. for $C_{22}H_{31}ClNO_2S$ ($[M+H]^+$) 408.1759, Found 408.1760.

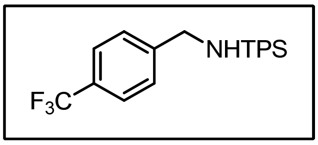

2,4,6-Triisopropyl-*N*-(4-(trifluoromethyl)benzyl)benzenesulfonamide (1l)
White solid (90% yield). m.p. 148-150 °C. **^{1}H NMR** (400 MHz, $CDCl_3$) δ 7.53 (d, *J* = 8.0 Hz, 2H), 7.34 (d, *J* = 8.0 Hz, 2H), 7.17 (s, 2H), 4.68 (t, *J* = 6.4 Hz, 1H), 4.23 (d, *J* = 6.4 Hz, 2H), 4.19-4.07 (m, 2H), 2.98-2.85 (m, 1H), 1.27 (d, *J* = 7.2 Hz, 6H), 1.25 (d, *J* = 6.8 Hz, 12H). **^{13}C NMR** (100 MHz, $CDCl_3$) δ 153.2, 150.4, 140.9, 132.4, 130.1 (q, *J* = 32.2 Hz), 128.3, 125.6 (q, *J* = 3.7 Hz), 124.1 (q, *J* = 270.5 Hz), 124.0, 46.5, 34.3, 29.8, 25.0, 23.7. **HRMS** (ESI) calcd. for $C_{23}H_{31}F_3NO_2S$ ($[M+H]^+$) 442.2022, Found 442.2024.

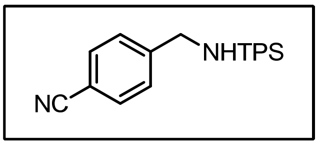

***N*-(4-Cyanobenzyl)-2,4,6-triisopropylbenzenesulfonamide (1m)**
White solid (83% yield). m.p. 188-190 °C. **^{1}H NMR** (400 MHz, $CDCl_3$) δ 7.57 (d, *J* = 8.0 Hz, 2H), 7.35 (d, *J* = 8.0 Hz, 2H), 7.17 (s, 2H), 4.73 (t, *J* = 6.4 Hz, 1H), 4.23 (d, *J* = 6.4 Hz, 2H), 4.18-4.05 (m, 2H), 2.98-2.85 (m, 1H), 1.27 (d, *J* = 6.8 Hz, 6H), 1.25 (d, *J* = 6.4 Hz, 12H). **^{13}C**

NMR (100 MHz, $CDCl_3$) δ 153.2, 150.3, 142.5, 132.4, 132.3, 128.6, 123.9, 118.6, 111.5, 46.4, 34.2, 29.8, 24.9, 23.7. **HRMS** (ESI) calcd. for $C_{23}H_{31}N_2O_2S$ ($[M+H]^+$) 399.2101, Found 399.2108.

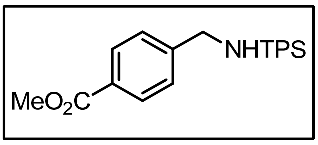

Methyl 4-((2,4,6-triisopropylphenylsulfonamido)methyl)benzoate (1n)
White solid (87% yield). m.p. 164-165 °C. **^{1}H NMR** (400 MHz, $CDCl_3$) δ 7.94 (d, J = 8.4 Hz, 2H), 7.28 (d, J = 8.4 Hz, 2H), 7.17 (s, 2H), 4.66 (t, J = 6.2 Hz, 1H), 4.22 (d, J = 6.4 Hz, 2H), 4.19-4.08 (m, 2H), 3.90 (s, 3H), 2.97-2.85 (m, 1H), 1.26 (d, J = 6.8 Hz, 6H), 1.25 (d, J = 6.8 Hz, 12H). **^{13}C NMR** (100 MHz, $CDCl_3$) δ 166.8, 153.1, 150.4, 141.9, 132.3, 130.0, 129.7, 127.9, 123.9, 52.2, 46.6, 34.2, 29.8, 25.0, 23.7. **HRMS** (ESI) calcd. for $C_{24}H_{34}NO_4S$ ($[M+H]^+$) 432.2203, Found 432.2211.

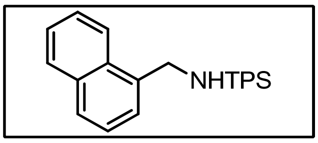

2,4,6-Triisopropyl-*N*-(naphthalen-1-ylmethyl)benzenesulfonamide (1o)
White solid (85% yield). m.p. 107-108 °C. **^{1}H NMR** (400 MHz, $CDCl_3$) δ 7.99-7.91 (m, 1H), 7.89-7.83 (m, 1H), 7.80 (d, J = 8.0 Hz, 1H), 7.54-7.45 (m, 2H), 7.35 (t, J = 7.6 Hz, 1H), 7.31-7.26 (m, 1H), 7.21 (s, 2H), 4.58 (d, J = 6.0 Hz, 2H), 4.52 (t, J = 6.0 Hz, 1H), 4.26-4.14 (m, 2H), 3.01-2.88 (m, 1H), 1.29 (d, J = 6.8 Hz, 6H), 1.25 (d, J = 6.8 Hz, 12H). **^{13}C NMR** (100 MHz, $CDCl_3$) δ 153.1, 150.6, 133.9, 132.1, 131.7, 131.4, 129.2, 128.8, 127.1, 126.9, 126.2, 125.3, 124.0, 123.5, 45.1, 34.3, 29.9, 25.0, 23.8. **HRMS** (ESI) calcd. for $C_{26}H_{34}NO_2S$ ($[M+H]^+$) 424.2305, Found 424.2308.

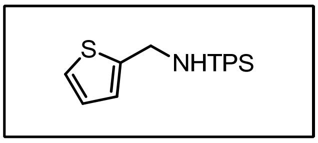

2,4,6-Triisopropyl-*N*-(thiophen-2-ylmethyl)benzenesulfonamide (1p)
Off-white solid (80% yield). m.p. 109-110 °C. **^{1}H NMR** (400 MHz, $CDCl_3$) δ 7.19 (dd, J = 4.8, 1.2 Hz, 1H), 7.17 (s, 2H), 6.9-6.87 (m, 1H), 6.86-6.82 (m, 1H), 4.58 (t, J = 6.0 Hz, 1H), 4.36 (d, J = 6.0 Hz, 2H), 4.23-4.09 (m, 2H), 2.98-2.84 (m, 1H), 1.27 (d, J = 6.8 Hz, 6H), 1.26 (d, J = 6.8 Hz, 12H). **^{13}C NMR** (100 MHz, $CDCl_3$) δ 153.1, 150.5, 139.0, 132.2, 127.0, 126.7, 125.9, 124.0, 41.8, 34.3, 29.8, 25.0, 23.7. **HRMS** (ESI) calcd. for $C_{20}H_{30}NO_2S_2$ ($[M+H]^+$) 380.1712, Found 380.1715.

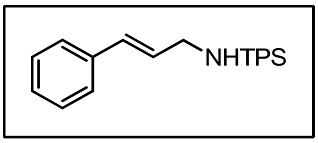

N-Cinnamyl-2,4,6-triisopropylbenzenesulfonamide (1q)
White solid (70% yield). m.p. 103-105 °C. **^{1}H NMR** (400 MHz, $CDCl_3$) δ 7.31-7.25 (m, 2H), 7.25-7.20 (m, 3H), 7.18 (s, 2H), 6.46 (d, J = 15.6 Hz, 1H), 6.01 (dt, J = 16.0, 6.6 Hz, 1H), 4.44 (t, J = 6.2 Hz, 1H), 4.27-4.12 (m, 2H), 3.79 (t, J = 6.4 Hz, 2H), 2.96-2.84 (m, 1H), 1.28 (d, J = 6.8 Hz, 12H), 1.26 (d, J = 7.6 Hz, 6H). **^{13}C NMR** (100 MHz, $CDCl_3$) δ 152.9, 150.3, 136.2, 133.1, 132.7, 128.7, 128.0, 126.5, 124.5, 123.9, 45.4, 34.2, 29.8, 25.0, 23.7. **HRMS** (ESI) calcd. for $C_{24}H_{34}NO_2S$ ($[M+H]^+$) 400.2305, Found 400.2300.

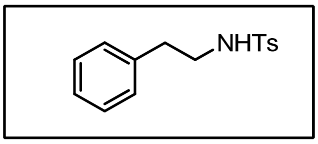

N-Cinnamyl-2,4,6-triisopropylbenzenesulfonamide (1r)[4]
White solid (89% yield). m.p. 64-66 °C. **^{1}H NMR** (400 MHz, $CDCl_3$) δ 7.69 (d, J = 8.4 Hz, 2H), 7.34-7.19 (m, 5H), 7.08 (d, J = 6.8 Hz, 2H), 4.40 (t, J = 6.0 Hz, 1H), 3.21 (q, J = 6.8 Hz, 2H), 2.76 (t, J = 6.8 Hz, 2H), 2.43 (s, 3H). **^{13}C NMR** (100 MHz, $CDCl_3$) δ 143.4, 137.9, 136.8, 129.7, 128.8, 128.7, 127.1, 126.7, 44.3, 35.8, 21.5.

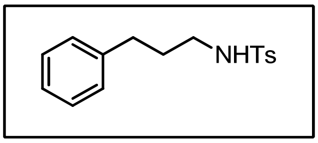

4-Methyl-N-(3-phenylpropyl)benzenesulfonamide (1s)[5]
White solid (97% yield). m.p. 65-67 °C. **^{1}H NMR** (400 MHz, $CDCl_3$) δ 7.73 (d, J = 8.4 Hz, 2H), 7.30 (d, J = 8.0 Hz, 2H), 7.28-7.22 (m, 2H), 7.18 (t, J = 7.2 Hz, 1H), 7.08 (d, J = 6.8 Hz, 2H), 4.59-4.41 (m, 1H), 2.97 (q, J = 6.4 Hz, 2H), 2.60 (t, J = 7.6 Hz, 2H), 2.43 (s, 3H), 1.86-1.72 (m, 2H). **^{13}C NMR** (100 MHz, $CDCl_3$) δ 143.4, 141.0, 137.0, 129.8, 128.5, 128.4, 127.2, 126.1, 42.7, 32.7, 31.2, 21.6.

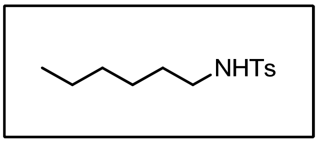

N-Hexyl-4-methylbenzenesulfonamide (1t)[6]
White solid (84% yield). m.p. 63-65 °C. **^{1}H NMR** (400 MHz, $CDCl_3$) δ 7.74 (d, J = 8.4 Hz, 2H), 7.31 (d, J = 8.0 Hz, 2H), 4.43-4.29 (m, 1H), 2.92 (q, J = 6.4 Hz, 2H), 2.43 (s, 3H), 1.51-1.38 (m, 2H), 1.32-1.14 (m, 6H), 0.84 (t, J = 6.8 Hz, 3H). **^{13}C NMR** (100 MHz, $CDCl_3$) δ 143.1, 137.0, 129.6, 127.1, 43.2, 31.2, 29.4, 26.1, 22.4, 21.4, 13.9.

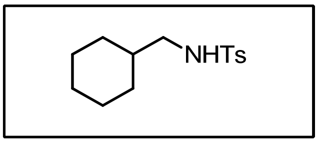

N-(Cyclohexylmethyl)-4-methylbenzenesulfonamide (1u)[5]

White solid (74% yield). m.p. 79-81 °C. **^{1}H NMR** (400 MHz, $CDCl_3$) δ 7.74 (d, J = 8.4 Hz, 2H), 7.30 (d, J = 8.0 Hz, 2H), 4.45 (t, J = 6.4 Hz, 1H), 2.75 (t, J = 6.6 Hz, 2H), 2.43 (s, 3H), 1.74-1.60 (m, 5H), 1.46-1.32 (m, 1H), 1.24-1.02 (m, 3H), 0.91-0.77 (m, 2H). **^{13}C NMR** (100 MHz, $CDCl_3$) δ 143.2, 137.2, 129.7, 127.1, 49.4, 37.8, 30.6, 26.3, 25.7, 21.6.

Supplementary Note 2

Reaction optimization

Supplementary Table 1. Screening of NHC ligand.

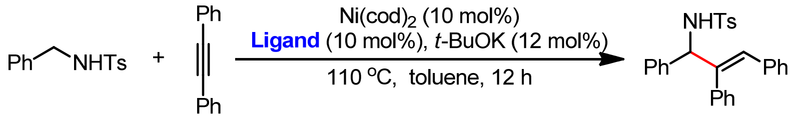

1a **2a** **3a**

entry	Ligand	yield (%)^{a,b}
1	IPr·HCl	19
2	IPr^{Me}·HCl	8
3	**IMes· HCl**	**29**
4	SIPr·HCl	0
5	IPr^{NQ}·HCl	0
6	IMe·HI	9
7	ICy·HCl	trace
8	I^{t}Bu·HBF_4	20

^{a}Reaction conditions: **1a** (0.2 mmol), **2a** (0.22 mmol), toluene (2 mL). ^{b}Determined by ^{1}H NMR with TCE as the internal standard.

Supplementary Table 2. Screening of protecting groups

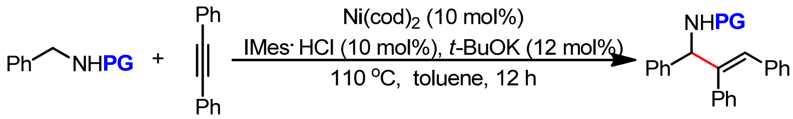

1a **2a** **3a**

entry	PG	yield (%)^{a,b}
1	p-CF_3-$C_6H_4SO_2$	42
2	3,5-(CF_3)2-$C_6H_3SO_2$	40
3	p-NO_2-$C_6H_4SO_2$	0
4	p-Me-$C_6H_4SO_2$ (Ts)	29
5	2,4,6-Me_3-$C_6H_2SO_2$ (TMS)	22
6	2,4,6-^{i}Pr_3-$C_6H_2SO_2$ (TPS)	56
7	p-OMe-$C_6H_4SO_2$	32
8	CH_3-SO_2 (Ms)	35
9	^{t}Bu-SO_2 (Bs)	15
10	CF_3-SO_2 (Tf)	19
11^c	**2,4,6-^{i}Pr_3-$C_6H_2SO_2$ (TPS)**	**68**
12^d	2,4,6-^{i}Pr_3-$C_6H_2SO_2$ (TPS)	24

^{a}Reaction conditions: **1a** (0.2 mmol), **2a** (0.22 mmol), toluene (2 mL). ^{b}Determined by ^{1}H NMR with TCE as the internal standard. ^{c}IPr· HCl (10 mol%). ^{d}IPr^{Me}· HCl (10 mol%).

Supplementary Table 3. Phosphine ligand effects

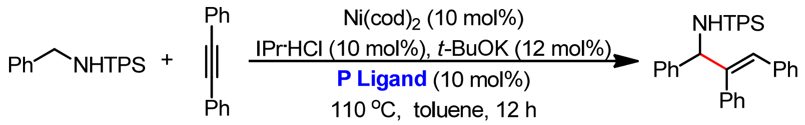

1a **2a** **3a**

entry	P Ligand	yield (%)[a,b]
1	0	68
2	PPh_3	76
3	BINAP	19
4	dppe	26
5	$^{n}Bu_3P$	38
6	$^{t}Bu_3P$	70
7	PCy_{p3}	83
8	PCy_3	99
9	dcype	23
10[c]	**PCy_3**	**99**

[a]Reaction conditions: **1a** (0.2 mmol), **2a** (0.22 mmol), toluene (2 mL). [b]Determined by ^{1}H NMR with TCE as the internal standard.[c]$Ni(cod)_2$ (5 mol%), IPr·HCl (5 mol%), *t*-BuOK (6 mol%)

Supplementary Table 4. Temperature effects

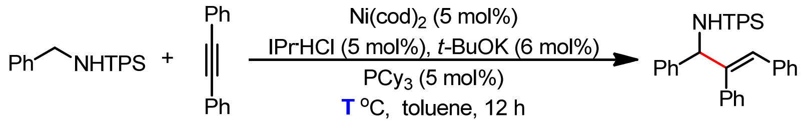

1a **2a** **3a**

entry	T	yield (%)[a,b]
1	50	67
2	60	75
3	70	89
4	**80**	**99**
5	90	99
6	100	99

[a]Reaction conditions: **1a** (0.2 mmol), **2a** (0.22 mmol), toluene (2 mL). [b]Determined by ^{1}H NMR with TCE as the internal standard.

Supplementary Table 5. Substrate loading effects

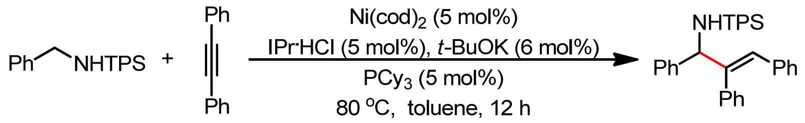

1a **2a** **3a**

entry	1a (mmol)	2a (mmol)	yield (%)[a,b]
1	0.15	0.1	78
2	0.12	0.1	92
3	0.11	0.1	95
4	0.1	0.1	95
5	**0.1**	**0.11**	**99**
6	0.1	0.12	84
7	0.1	0.15	79

[a]Reaction conditions: toluene (2 mL). [b]Determined by ^{1}H NMR with TCE as the internal standard.

Supplementary Table 6. Ni catalyst effects

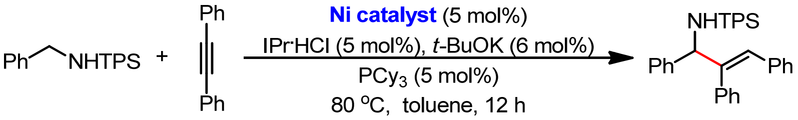

entry	Ni catalyst	additive	yield (%)[a,b]
1	$Ni(cod)_2$	-	99
2	$Ni(OAc)_2{\cdot}4H_2O$	-	0
3	$Ni(PPh_3)_2Cl_2$	-	0
4	$NiBr_2$	-	0
5	$NiCl_2{\cdot}$glyme	-	0
6	$NiCl_2{\cdot}$glyme	Mn	0
7	$Ni(acac)_2$	-	0

[a]Reaction conditions: **1a** (0.2 mmol), **2a** (0.22 mmol), toluene (2 mL). [b]Determined by ^{1}H NMR with TCE as the internal standard.

Supplementary Note 3

General procedure for hydroaminoalkylation

To a 15 mL pressure tube were added $Ni(cod)_2$ (2.75 mg, 0.01 mmol), IPr·HCl (4.25 mg, 0.01 mmol), PCy_3 (2.8 mg, 0.01 mmol), KO^{t}Bu (1.34 mg, 0.012 mmol), toluene (2.0 mL), alkynes (0.22 mmol) and amines (0.20 mmol) in a glove box. The tube was sealed with a Teflon cap and the mixture was stirred at 80 °C or 110 °C for 1-12 h. After cooled to room temperature, the crude product was filtered through a short pad of Celite, and the filtrate was concentrated under vacuum. The resulting residue was obtained by chromatography on silica gel column with petroleum ether/ethyl acetate as the eluent. The analytic data for the products are listed below.

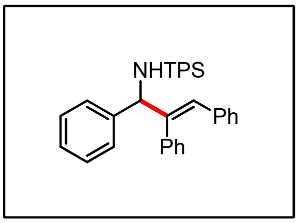

(*E*)-2,4,6-Triisopropyl-*N*-(1,2,3-triphenylallyl)benzenesulfonamide (3a)
Purified by column (PE/EtOAc = 50:1 to 30:1), white solid (94% yield). m.p. 118-119 °C. **^{1}H NMR** (400 MHz, $CDCl_3$) δ 7.23-7.13 (m, 6H), 7.13-7.07 (m, 4H), 7.06-6.99 (m, 3H), 6.86-6.81 (m, 2H), 6.79-6.74 (m, 2H), 6.73 (s, 1H), 5.39 (d, J = 5.6 Hz, 1H), 4.78 (d, J = 5.6 Hz, 1H), 4.17-4.04 (m, 2H), 2.94-2.81 (m, 1H), 1.24 (d, J = 6.4 Hz, 12H), 1.12 (d, J = 6.8 Hz, 6H). **^{13}C NMR** (100 MHz, $CDCl_3$) δ 152.8, 150.0, 140.8, 139.2, 138.1, 136.1, 134.0, 129.4, 129.3, 128.8, 128.7, 128.7, 128.0, 127.9, 127.6, 127.6, 127.1, 123.8, 64.5, 34.3, 30.0, 25.1, 24.8, 23.8, 23.7. **HRMS** (ESI) calcd. for $C_{36}H_{41}NNaO_2S$ ($[M+Na]^+$) 574.2750, Found 574.2742.

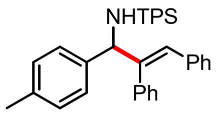

(*E*)-*N*-(2,3-Diphenyl-1-(*p*-tolyl)allyl)-2,4,6-triisopropylbenzenesulfonamide (3b)
Purified by column (PE/EtOAc = 50:1 to 30:1), white solid (89% yield). m.p. 56-58 °C. **^{1}H NMR** (400 MHz, $CDCl_3$) δ 7.23-7.14 (m, 3H), 7.11 (s, 2H), 7.07-7.02 (m, 3H), 7.00 (s, 4H), 6.86 (d, J = 7.2 Hz, 2H), 6.76 (d, J = 6.8 Hz, 2H), 6.71 (s, 1H), 5.36 (d, J = 5.6 Hz, 1H), 4.75 (d, J = 5.6 Hz, 1H), 4.19-4.05 (m, 2H), 2.95-2.81 (m, 1H), 2.28 (s, 3H), 1.25 (d, J = 6.4 Hz, 12H), 1.14 (d, J = 6.8 Hz, 6H). **^{13}C NMR** (100 MHz, $CDCl_3$) δ 152.8, 150.0, 141.0, 138.3, 137.7, 136.2, 136.2, 134.1, 129.4, 129.3, 129.3, 128.7, 128.6, 127.9, 127.6, 127.0, 123.8, 64.3, 34.3, 30.0, 25.1, 24.8, 23.8, 23.8, 21.2. **HRMS** (ESI) calcd. for $C_{37}H_{43}NNaO_2S$ ($[M+Na]^+$) 588.2907, Found 588.2899.

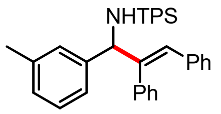

(*E*)-*N*-(2,3-Diphenyl-1-(*m*-tolyl)allyl)-2,4,6-triisopropylbenzenesulfonamide (3c)
Purified by column (PE/EtOAc = 50:1 to 30:1), white solid (89% yield). m.p. 74-76 °C. **^{1}H NMR** (400 MHz, $CDCl_3$) δ 7.23-7.15 (m, 3H), 7.14-7.10 (m, 2H), 7.10-6.97 (m, 5H), 6.91 (d, J = 7.6 Hz, 1H), 6.89-6.84 (m, 3H), 6.82-6.76 (m, 2H), 6.74 (s, 1H), 5.37 (d, J = 5.6 Hz, 1H), 4.77 (d, J = 5.6 Hz, 1H), 4.18-4.04 (m, 2H), 2.97-2.80 (m, 1H), 2.20 (s, 3H), 1.30-1.17 (m, 12H), 1.13 (d, J = 6.8 Hz, 6H). **^{13}C NMR** (100 MHz, $CDCl_3$) δ 152.7, 149.9, 140.9, 139.1, 138.3, 138.2, 136.2, 134.0, 129.4, 129.3, 128.9, 128.7, 128.7, 128.5, 128.4, 127.9, 127.6, 127.0, 124.7, 123.8, 64.5, 34.3, 30.0, 25.1, 24.8, 23.7, 21.5. **HRMS** (ESI) calcd. for $C_{37}H_{43}NNaO_2S$ ($[M+Na]^+$) 588.2907, Found 588.2907.

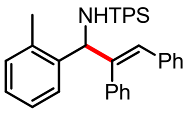

(*E*)-*N*-(2,3-Diphenyl-1-(*o*-tolyl)allyl)-2,4,6-triisopropylbenzenesulfonamide (3d)
Purified by column (PE/EtOAc = 50:1 to 30:1), white solid (62% yield). m.p. 126-127 °C. **^{1}H NMR** (400 MHz, $CDCl_3$) δ 7.25-7.20 (m, 1H), 7.20-7.13 (m, 3H), 7.12-7.06 (m, 4H), 7.05-6.96 (m, 4H), 6.92-6.86 (m, 2H), 6.70 (d, J = 7.6 Hz, 2H), 6.68 (s, 1H), 5.58 (d, J = 4.8 Hz, 1H), 4.77 (d, J = 4.4 Hz, 1H), 4.16-4.02 (m, 2H), 2.93-2.78 (m, 1H), 1.90 (s, 3H), 1.25 (d, J = 6.4 Hz, 6H), 1.21 (dd, J = 6.8, 2.4 Hz, 6H), 1.09 (d, J = 6.8 Hz, 6H). **^{13}C NMR** (100 MHz, $CDCl_3$) δ 152.7, 149.8, 140.5, 138.8, 137.4, 136.2, 136.2, 134.4, 130.7, 129.2, 129.2, 128.6,

128.2, 128.0, 127.9, 127.5, 127.3, 126.9, 126.4, 123.8, 60.3, 34.3, 30.0, 25.1, 24.6, 23.7, 23.7, 19.0. **HRMS** (ESI) calcd. for $C_{37}H_{43}NNaO_2S$ ([M+Na]$^+$) 588.2907, Found 588.2901.

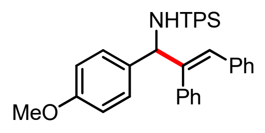

(*E*)-2,4,6-Triisopropyl-*N*-(1-(4-methoxyphenyl)-2,3-diphenylallyl)benzenesulfonamide (3e)

Purified by column (PE/EtOAc = 50:1 to 30:1), white solid (85% yield). m.p. 59-61 °C. **^{1}H NMR** (400 MHz, $CDCl_3$) δ 7.22-7.13 (m, 3H), 7.11 (s, 2H), 7.07-6.97 (m, 5H), 6.88-6.82 (m, 2H), 6.77-6.71 (m, 3H), 6.71-6.67 (m, 2H), 5.34 (d, J = 5.6 Hz, 1H), 4.73 (d, J = 5.2 Hz, 1H), 4.18-4.04 (m, 2H), 3.75 (s, 3H), 2.94-2.81 (m, 1H), 1.29-1.19 (m, 12H), 1.13 (d, J = 6.8 Hz, 6H). **^{13}C NMR** (100 MHz, $CDCl_3$) δ 159.3, 152.8, 149.9, 141.0, 138.4, 136.1, 134.0, 131.2, 129.4, 129.3, 128.9, 128.7, 128.4, 127.9, 127.6, 127.0, 123.8, 114.0, 64.0, 55.3, 34.3, 30.0, 25.1, 24.8, 23.8, 23.7. **HRMS** (ESI) calcd. for $C_{37}H_{43}NNaO_3S$ ([M+Na]$^+$) 604.2856, Found 604.2859.

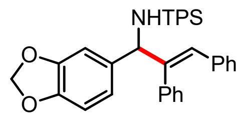

(*E*)-*N*-(1-(Benzo[d][1,3]dioxol-5-yl)-2,3-diphenylallyl)-2,4,6-triisopropylbenzenesulfonamide (3f)

Purified by column (PE/EtOAc = 50:1 to 30:1), white solid (84% yield). m.p. 55-57 °C. **^{1}H NMR** (400 MHz, $CDCl_3$) δ 7.25-7.15 (m, 3H), 7.11 (s, 2H), 7.09-6.99 (m, 3H), 6.92-6.84 (m, 2H), 6.78-6.71 (m, 2H), 6.68 (s, 1H), 6.64-6.52 (m, 3H), 5.94-5.88 (m, 2H), 5.29 (d, J = 5.2 Hz, 1H), 4.72 (d, J = 5.6 Hz, 1H), 4.17-4.04 (m, 2H), 2.94-2.81 (m, 1H), 1.31-1.19 (m, 12H), 1.14 (d, J = 6.8 Hz, 6H). **^{13}C NMR** (100 MHz, $CDCl_3$) δ 152.8, 149.9, 147.9, 147.3, 140.8, 138.2, 136.0, 133.9, 133.1, 129.3, 129.3, 128.7, 128.6, 127.9, 127.6, 127.0, 123.8, 121.3, 108.2, 108.0, 101.2, 64.2, 34.2, 30.0, 25.1, 24.8, 23.7, 23.7. **HRMS** (ESI) calcd. for $C_{37}H_{41}NNaO_4S$ ([M+Na]$^+$) 618.2649, Found 618.2655.

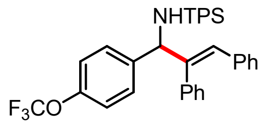

(*E*)-*N*-(2,3-Diphenyl-1-(4-(trifluoromethoxy)phenyl)allyl)-2,4,6-triisopropylbenzenesulfonamide (3g)

Purified by column (PE/EtOAc = 50:1 to 30:1), white solid (90% yield). m.p. 124-126 °C. **^{1}H**

NMR (400 MHz, $CDCl_3$) *δ* 7.25-7.15 (m, 3H), 7.14-7.08 (m, 4H), 7.08-6.97 (m, 5H), 6.85-6.80 (m, 2H), 6.79-6.74 (m, 2H), 6.67 (s, 1H), 5.45 (d, *J* = 5.6 Hz, 1H), 4.78 (d, *J* = 6.0 Hz, 1H), 4.12-3.98 (m, 2H), 2.94-2.81 (m, 1H), 1.23 (dd, *J* = 7.2, 1.2 Hz, 6H), 1.20 (d, *J* = 6.8 Hz, 6H), 1.13 (d, *J* = 6.4 Hz, 6H). **^{13}C NMR** (100 MHz, $CDCl_3$) *δ* 153.1, 150.0, 148.8, 140.5, 138.0, 137.7, 135.8, 133.8, 129.5, 129.3, 129.1, 128.9, 128.0, 127.9, 127.3, 123.8, 120.9, 120.5 (q, *J* = 255.7 Hz), 63.9, 34.3, 30.0, 25.0, 24.8, 23.7, 23.7. **HRMS** (ESI) calcd. for $C_{37}H_{40}F_3NNaO_3S$ ($[M+Na]^+$) 658.2573, Found 658.2568.

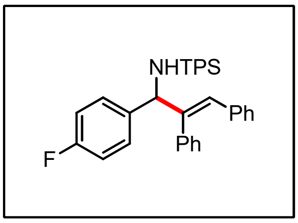

(*E*)-*N*-(1-(4-Fluorophenyl)-2,3-diphenylallyl)-2,4,6-triisopropylbenzenesulfonamide (3h)
Purified by column (PE/EtOAc = 50:1 to 30:1), white solid (89% yield). m.p. 54-56 °C. **^{1}H NMR** (400 MHz, $CDCl_3$) *δ* 7.24-7.15 (m, 3H), 7.12 (s, 2H), 7.10-7.00 (m, 5H), 6.92-6.81 (m, 4H), 6.80-6.73 (m, 2H), 6.70 (s, 1H), 5.41 (d, *J* = 5.6 Hz, 1H), 4.79 (d, *J* = 5.6 Hz, 1H), 4.18-4.02 (m, 2H), 2.95-2.82 (m, 1H), 1.32-1.20 (m, 12H), 1.15 (d, *J* = 6.8 Hz, 6H). **^{13}C NMR** (100 MHz, $CDCl_3$) *δ* 162.3 (d, *J* = 245.6 Hz), 153.0, 150.0, 140.7, 137.9, 135.9, 135.0 (d, *J* = 2.9 Hz), 133.8, 129.4, 129.3, 129.3, 129.0, 128.8, 128.0, 127.8, 127.2, 123.8, 115.4(d, *J* = 21.5 Hz), 63.9, 34.3, 30.0, 25.0, 24.8, 23.7. **HRMS** (ESI) calcd. for $C_{36}H_{40}FNNaO_2S$ ($[M+Na]^+$) 592.2656, Found 592.2652.

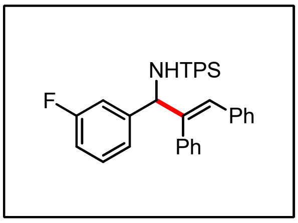

(*E*)-*N*-(1-(3-Fluorophenyl)-2,3-diphenylallyl)-2,4,6-triisopropylbenzenesulfonamide (3i)
Purified by column (PE/EtOAc = 50:1 to 30:1), white solid (94% yield). m.p. 139-141 °C. **^{1}H NMR** (400 MHz, $CDCl_3$) *δ* 7.24-7.15 (m, 4H), 7.12 (s, 2H), 7.10-7.00 (m, 3H), 6.97-6.88 (m, 2H), 6.85 (d, *J* = 6.8 Hz, 2H), 6.82-6.74 (m, 3H), 6.70 (s, 1H), 5.43 (d, *J* = 6.0 Hz, 1H), 4.80 (d, *J* = 6.0 Hz, 1H), 4.17-4.01 (m, 2H), 2.95-2.82 (m, 1H), 1.30-1.19 (m, 12H), 1.16 (d, *J* = 6.8 Hz, 6H). **^{13}C NMR** (100 MHz, $CDCl_3$) *δ* 162.9 (d, *J* = 245.5 Hz), 153.1, 150.0, 142.0 (d, *J* = 6.7 Hz), 140.4, 137.6, 135.8, 133.7, 130.2, 130.1, 129.6, 129.4, 128.9, 128.0, 127.9, 127.3, 123.9, 123.3 (d, *J* = 2.3 Hz), 114.9 (d, *J* = 20.9 Hz), 114.6 (d, *J* = 22.3 Hz), 64.0, 34.3, 30.0, 25.1, 24.8, 23.7. **HRMS** (ESI) calcd. for $C_{36}H_{40}FNNaO_2S$ ($[M+Na]^+$) 592.2656, Found 592.2651.

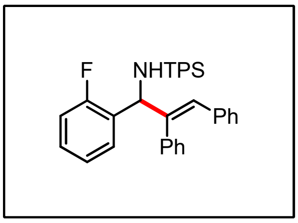

(*E*)-*N*-(1-(2-Fluorophenyl)-2,3-diphenylallyl)-2,4,6-triisopropylbenzenesulfonamide (3j)
Purified by column (PE/EtOAc = 50:1 to 30:1), white solid (82% yield). m.p. 97-99 °C. **^{1}H NMR** (400 MHz, $CDCl_3$) δ 7.24-7.12 (m, 4H), 7.08 (s, 2H), 7.06-6.95 (m, 4H), 6.94-6.85 (m, 4H), 6.79-6.73 (m, 2H), 6.63 (s, 1H), 5.67 (d, *J* = 6.8 Hz, 1H), 5.00 (d, *J* = 6.8 Hz, 1H), 4.16-4.00 (m, 2H), 2.93-2.78 (m, 1H), 1.26-1.18 (m, 12H), 1.15 (d, *J* = 6.8 Hz, 6H). **^{13}C NMR** (100 MHz, $CDCl_3$) δ 160.3 (d, *J* = 246.0 Hz), 152.8, 150.0, 139.9, 137.8, 136.0, 133.6, 129.5 (d, *J* = 8.3 Hz), 129.3, 129.3, 129.1, 128.8, 127.9, 127.7, 127.1, 126.8, 126.7, 124.0 (d, *J* = 3.2 Hz), 123.7, 115.7 (d, *J* = 21.5 Hz), 58.6, 34.3, 30.1, 25.0, 24.8, 23.7. **HRMS** (ESI) calcd. for $C_{36}H_{40}FNNaO_2S$ ($[M+Na]^+$) 592.2656, Found 592.2656.

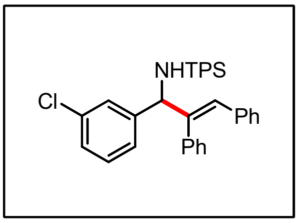

(*E*)-*N*-(1-(3-Chlorophenyl)-2,3-diphenylallyl)-2,4,6-triisopropylbenzenesulfonamide (3k)
Purified by column (PE/EtOAc = 50:1 to 20:1), white solid (93% yield). m.p. 111-113 °C. **^{1}H NMR** (400 MHz, $CDCl_3$) δ 7.24-7.13 (m, 4H), 7.12-7.09 (m, 3H), 7.09-6.98 (m, 5H), 6.88-6.82 (m, 2H), 6.82-6.76 (m, 2H), 6.68 (s, 1H), 5.41 (d, *J* = 6.0 Hz, 1H), 4.77 (d, *J* = 6.0 Hz, 1H), 4.13-3.97 (m, 2H), 2.94-2.79 (m, 1H), 1.24 (dd, *J* = 6.8, 1.2 Hz, 6H), 1.21 (d, *J* = 6.8 Hz, 6H), 1.15 (d, *J* = 6.8 Hz, 6H). **^{13}C NMR** (100 MHz, $CDCl_3$) δ 153.0, 150.0, 141.4, 140.3, 137.5, 135.8, 134.5, 133.6, 129.8, 129.7, 129.4, 128.9, 128.1, 128.0, 128.0, 127.7, 127.3, 125.8, 123.9, 64.0, 34.3, 30.0, 25.0, 24.9, 23.7, 23.7. **HRMS** (ESI) calcd. for $C_{36}H_{40}ClNNaO_2S$ ($[M+Na]^+$) 608.2360, Found 608.2356.

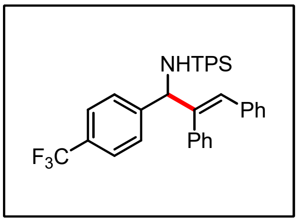

(*E*)-*N*-(2,3-Diphenyl-1-(4-(trifluoromethyl)phenyl)allyl)-2,4,6-triisopropylbenzenesulfonamide (3l)
Purified by column (PE/EtOAc = 50:1 to 30:1), white solid (88% yield). m.p. 155-156 °C. **^{1}H NMR** (400 MHz, $CDCl_3$) δ 7.43 (d, *J* = 8.4 Hz, 2H), 7.26-7.16 (m, 5H), 7.10 (s, 2H), 7.09-7.02 (m, 3H), 6.86-6.74 (m, 4H), 6.66 (s, 1H), 5.52 (d, *J* = 5.6 Hz, 1H), 4.84 (d, *J* = 6.0 Hz, 1H), 4.11-3.96 (m, 2H), 2.96-2.81 (m, 1H), 1.24 (dd, *J* = 6.8, 1.6 Hz, 6H), 1.19 (d, *J* = 6.8 Hz, 6H), 1.16 (d, *J* = 6.8 Hz, 6H). **^{13}C NMR** (100 MHz, $CDCl_3$) δ 153.2, 150.0, 143.4, 140.3,

137.4, 135.7, 133.7, 130.0 (q, *J* = 32.3 Hz), 130.0, 129.3, 129.0, 128.1, 128.0, 127.5, 125.4 (q, *J* = 3.3 Hz), 123.8, 121.4 (q, *J* = 270.3 Hz), 64.2, 34.3, 30.0, 24.9, 24.9, 23.7. **HRMS** (ESI) calcd. for $C_{37}H_{40}F_3NNaO_2S$ ($[M+Na]^+$) 642.2624, Found 642.2621.

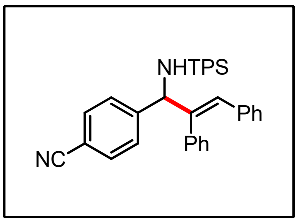

(*E*)-*N*-(1-(4-Cyanophenyl)-2,3-diphenylallyl)-2,4,6-triisopropylbenzenesulfonamide (3m)
Purified by column (PE/EtOAc = 50:1 to 10:1), white solid (84% yield). m.p. 96-98 °C. **^{1}H NMR** (400 MHz, $CDCl_3$) δ 7.48 (d, *J* = 8.4 Hz, 2H), 7.26 (s, 1H), 7.24 (s, 1H), 7.23-7.16 (m, 3H), 7.11 (s, 2H), 7.09-7.02 (m, 3H), 6.81-6.73 (m, 4H), 6.63 (s, 1H), 5.50 (d, *J* = 5.6 Hz, 1H), 4.87 (d, *J* = 6.0 Hz, 1H), 4.10-3.95 (m, 2H), 2.96-2.81 (m, 1H), 1.25 (dd, *J* = 6.8, 1.6 Hz, 6H), 1.18 (d, *J* = 6.0 Hz, 6H), 1.17 (d, *J* = 6.4 Hz, 6H). **^{13}C NMR** (100 MHz, $CDCl_3$) δ 153.3, 150.1, 144.9, 139.9, 137.0, 135.4, 133.4, 132.2, 130.3, 129.3, 129.3, 129.1, 128.3, 128.2, 128.1, 127.6, 123.9, 118.6, 111.7, 64.2, 34.3, 30.0, 24.9, 23.8, 23.7. **HRMS** (ESI) calcd. for $C_{37}H_{40}N_2NaO_2S$ ($[M+Na]^+$) 599.2703, Found 599.2700.

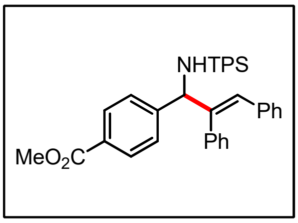

(*E*)-Methyl 4-(2,3-diphenyl-1-(2,4,6-triisopropylphenylsulfonamido)allyl)benzoate (3n)
Purified by column (PE/EtOAc = 50:1 to 15:1), white solid (92% yield). m.p. 121-123 °C. **^{1}H NMR** (400 MHz, $CDCl_3$) δ 7.85 (d, *J* = 8.4 Hz, 2H), 7.25-7.14 (m, 5H), 7.10 (s, 2H), 7.08-7.01 (m, 3H), 6.85-6.75 (m, 4H), 6.69 (s, 1H), 5.49 (d, *J* = 6.0 Hz, 1H), 4.84 (d, *J* = 6.0 Hz, 1H), 4.14-4.02 (m, 2H), 3.89 (s, 3H), 2.94-2.81 (m, 1H), 1.24 (dd, *J* = 6.8, 1.2 Hz, 6H), 1.20 (d, *J* = 6.8 Hz, 6H), 1.15 (d, *J* = 6.8 Hz, 6H). **^{13}C NMR** (100 MHz, $CDCl_3$) δ 166.7, 153.1, 150.0, 144.4, 140.3, 137.5, 135.7, 133.6, 129.8, 129.7, 129.6, 129.3, 129.3, 128.9, 128.0, 127.9, 127.6, 127.3, 123.8, 64.3, 52.2, 34.3, 30.0, 25.0, 24.9, 23.7, 23.7. **HRMS** (ESI) calcd. for $C_{38}H_{43}NNaO_4S$ ($[M+Na]^+$) 632.2805, Found 632.2806.

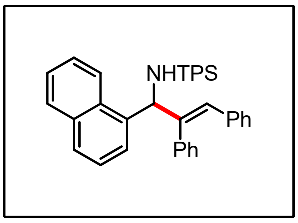

(*E*)-2,4,6-Triisopropyl-*N*-(1-(naphthalen-1-yl)-2,3-diphenylallyl)benzenesulfonamide (3o)
Purified by column (PE/EtOAc = 50:1 to 20:1), white solid (86% yield). m.p. 60-62 °C. **^{1}H NMR** (400 MHz, $CDCl_3$) δ 8.03 (d, *J* = 8.0 Hz, 1H), 7.81 (d, *J* = 7.6 Hz, 1H), 7.74 (d, *J* = 8.4

Hz, 1H), 7.48-7.36 (m, 3H), 7.28 (t, *J* = 8.0 Hz, 1H), 7.18-7.09 (m, 3H), 7.07 (s, 2H), 7.05-6.95 (m, 5H), 6.75-6.65 (m, 3H), 6.29 (d, *J* = 4.8 Hz, 1H), 4.92 (d, *J* = 4.8 Hz, 1H), 4.15-3.99 (m, 2H), 2.91-2.77 (m, 1H), 1.24-1.15 (m, 12H), 1.07 (d, *J* = 6.8 Hz, 6H). **^{13}C NMR** (100 MHz, $CDCl_3$) δ 152.7, 149.6, 140.0, 138.7, 136.2, 134.7, 134.4, 134.1, 131.1, 129.8, 129.2, 129.2, 129.1, 128.9, 128.6, 127.9, 127.6, 127.0, 126.8, 126.4, 125.9, 125.1, 123.8, 122.9, 60.7, 34.2, 30.1, 25.0, 24.7, 23.8, 23.7. **HRMS** (ESI) calcd. for $C_{40}H_{43}NNaO_2S$ ([M+Na]$^+$) 624.2907, Found 624.2900.

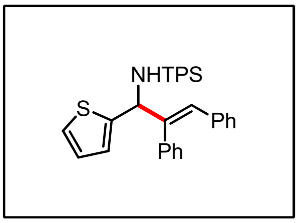

(*E*)-*N*-(2,3-Diphenyl-1-(thiophen-2-yl)allyl)-2,4,6-triisopropylbenzenesulfonamide (3p)
Purified by column (PE/EtOAc = 50:1 to 30:1), white solid (86% yield). m.p. 115-117 °C. **^{1}H NMR** (400 MHz, $CDCl_3$) δ 7.24-7.16 (m, 4H), 7.12 (s, 2H), 7.09-7.01 (m, 3H), 6.99-6.93 (m, 2H), 6.88-6.83 (m, 1H), 6.83-6.77 (m, 2H), 6.77-6.74 (m, 1H), 6.73 (s, 1H), 5.67 (d, *J* = 6.8 Hz, 1H), 4.86 (d, *J* = 6.8 Hz, 1H), 4.19-4.05 (m, 2H), 2.94-2.81 (m, 1H), 1.25 (d, *J* = 6.8 Hz, 6H), 1.23 (dd, *J* = 6.8, 2.0 Hz, 6H), 1.16 (d, *J* = 6.8 Hz, 6H). **^{13}C NMR** (100 MHz, $CDCl_3$) δ 152.9, 150.1, 144.0, 140.8, 137.6, 135.9, 133.8, 129.5, 129.4, 128.9, 128.8, 128.0, 127.9, 127.2, 127.1, 126.1, 125.9, 123.9, 60.2, 34.3, 30.0, 25.1, 24.9, 23.7, 23.7. **HRMS** (ESI) calcd. for $C_{34}H_{39}NNaO_2S_2$ ([M+Na]$^+$) 580.2314, Found 580.2322.

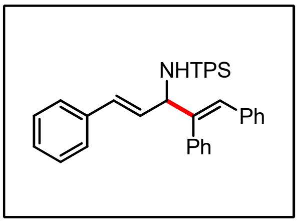

2,4,6-Triisopropyl-*N*-((1*E*,4*E*)-1,2,5-triphenylpenta-1,4-dien-3-yl)benzenesulfonamide (3q)
Purified by column (PE/EtOAc = 50:1 to 20:1), white solid (45% yield). m.p. 118-120 °C. **^{1}H NMR** (400 MHz, $CDCl_3$) δ 7.32-7.27 (m, 3H), 7.25-7.17 (m, 3H), 7.17-7.12 (m, 2H), 7.12-7.03 (m, 7H), 6.87-6.81 (m, 2H), 6.62 (s, 1H), 6.36 (d, *J* = 16.0 Hz, 1H), 6.02-5.92 (m, 1H), 5.04 (t, *J* = 6.8 Hz, 1H), 4.64 (d, *J* = 6.8 Hz, 1H), 4.20-4.06 (m, 2H), 2.92-2.77 (m, 1H), 1.28-1.12 (m, 18H). **^{13}C NMR** (100 MHz, $CDCl_3$) δ 152.8, 150.0, 140.6, 137.7, 136.3, 136.0, 134.1, 132.3, 129.5, 129.5, 129.4, 129.0, 128.6, 128.0, 128.0, 127.8, 127.3, 126.6, 123.8, 62.9, 34.2, 30.0, 25.0, 24.9, 23.7, 23.7. **HRMS** (ESI) calcd. for $C_{38}H_{43}NNaO_2S$ ([M+Na]$^+$) 600.2907, Found 600.2915.

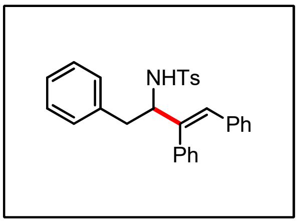

(*E*)-4-Methyl-*N*-(1,3,4-triphenylbut-3-en-2-yl)benzenesulfonamide (3r)
Purified by column (PE/EtOAc = 50:1 to 10:1), white solid (54% yield). m.p. 117-119 °C. **^{1}H NMR** (400 MHz, $CDCl_3$) δ 7.56 (d, *J* = 7.6 Hz, 2H), 7.36-7.29 (m, 3H), 7.24-7.18 (m, 3H), 7.14-7.07 (m, 4H), 7.06-6.97 (m, 5H), 6.75-6.65 (m, 2H), 6.30 (s, 1H), 4.53 (d, *J* = 6.4 Hz, 1H), 4.39 (q, *J* = 6.8 Hz, 1H), 2.93 (dd, *J* = 6.0, 13.6 Hz, 1H), 2.65 (dd, *J* = 8.0, 13.6 Hz, 1H), 2.30 (s, 3H).. **^{13}C NMR** (100 MHz, $CDCl_3$) δ 143.2, 140.2, 138.2, 137.5, 136.5, 136.2, 129.6, 129.5, 129.5, 129.2, 129.0, 128.7, 127.9, 127.7, 127.2, 126.9, 61.9, 40.9, 21.5. **HRMS** (ESI) calcd. for $C_{29}H_{26}NO_2S$ ($[M-H]^-$) 452.1684, Found 452.1685.

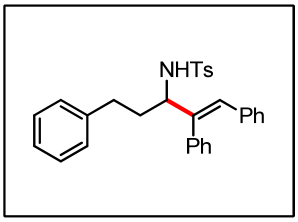

(*E*)-4-Methyl-N-(1,2,5-triphenylpent-1-en-3-yl)benzenesulfonamide (3s)
Purified by column (PE/EtOAc = 50:1 to 10:1), white semi-solid (41% yield). **^{1}H NMR** (400 MHz, $CDCl_3$) δ 7.74 (d, *J* = 8.4 Hz, 2H), 7.30-7.23 (m, 5H), 7.23-7.16 (m, 3H), 7.09 (d, *J* = 7.2 Hz, 2H), 7.07-6.98 (m, 3H), 6.94-6.86 (m, 2H), 6.75-6.64 (m, 2H), 6.31 (s, 1H), 4.50 (d, *J* = 8.8 Hz, 1H), 4.20 (q, *J* = 7.6 Hz, 1H), 2.79-2.61 (m, 2H), 2.32 (s, 3H), 1.95-1.84 (m, 1H), 1.84-1.71 (m, 1H). **^{13}C NMR** (100 MHz, $CDCl_3$) δ 143.5, 141.1, 139.9, 138.1, 137.4, 136.0, 129.7, 129.4, 129.2, 129.0, 128.6, 127.9, 127.8, 127.4, 127.1, 126.2, 61.3, 36.8, 32.3, 21.6. **HRMS** (ESI) calcd. for $C_{30}H_{33}N_2O_2S$ ($[M+NH_4]^+$) 485.2257, Found 485.2259.

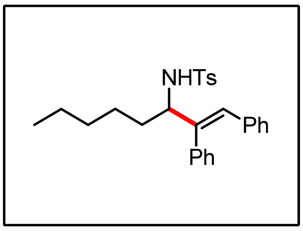

(*E*)-*N*-(1,2-Diphenyloct-1-en-3-yl)-4-methylbenzenesulfonamide (3t)
Purified by column (PE/EtOAc = 50:1 to 10:1), white solid (46% yield). m.p. 117-119 °C. **^{1}H NMR** (400 MHz, $CDCl_3$) δ 7.77 (d, *J* = 8.0 Hz, 2H), 7.30-7.26 (m, 3H), 7.21 (d, *J* = 8.0 Hz, 2H), 7.08-6.98 (m, 3H), 6.94-6.86 (m, 2H), 6.74-6.65 (m, 2H), 6.32 (s, 1H), 4.42 (d, *J* = 8.4 Hz, 1H), 4.14 (q, *J* = 7.6 Hz, 1H), 2.31 (s, 3H), 1.60-1.12 (m, 8H), 0.83 (t, *J* = 6.8 Hz, 3H). **^{13}C NMR** (100 MHz, $CDCl_3$) δ 143.4, 140.5, 138.3, 137.6, 136.1, 129.6, 129.5, 129.2, 129.2, 128.9, 127.9, 127.7, 127.4, 126.9, 61.6, 35.2, 31.4, 25.7, 22.6, 21.5, 14.1. **HRMS** (ESI) calcd. for $C_{27}H_{35}N_2O_2S$ ($[M+NH_4]^+$) 451.2414, Found 451.2416.

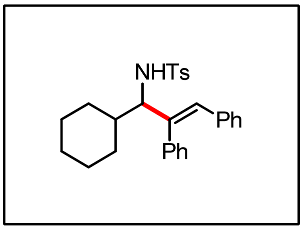

(*E*)-4-Methyl-*N*-(1,3,4-triphenylbut-3-en-2-yl)benzenesulfonamide (3u)[7]
Purified by column (PE/EtOAc = 50:1 to 10:1), white solid (45% yield). m.p. 155-157 °C. **^{1}H NMR** (400 MHz, $CDCl_3$) δ 7.77 (d, J = 8.4 Hz, 2H), 7.30-7.24 (m, 3H), 7.21 (d, J = 8.0 Hz, 2H), 7.09-6.96 (m, 3H), 6.88-6.78 (m, 2H), 6.68-6.59 (m, 2H), 6.19 (s, 1H), 4.43 (d, J = 9.6 Hz, 1H), 3.92 (t, J = 9.0 Hz, 1H), 2.30 (s, 3H), 1.96 (d, J = 12.4 Hz, 1H), 1.86-1.68 (m, 3H), 1.64 (s, 1H), 1.43-1.29 (m, 1H), 1.24-1.02 (m, 4H), 1.01-0.84 (m, 1H). **^{13}C NMR** (100 MHz, $CDCl_3$) δ 143.3, 138.8, 138.4, 137.7, 136.1, 130.1, 129.6, 129.4, 129.1, 128.9, 127.8, 127.7, 127.4, 126.8, 66.8, 40.1, 31.0, 28.9, 26.4, 26.1, 21.5.

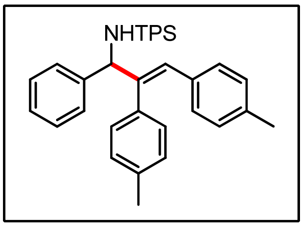

(*E*)-2,4,6-Triisopropyl-*N*-(1-phenyl-2,3-di-p-tolylallyl)benzenesulfonamide (4a)
Purified by column (PE/EtOAc = 50:1 to 30:1), white solid (86% yield). m.p. 63-65 °C. **^{1}H NMR** (400 MHz, $CDCl_3$) δ 7.21-7.16 (m, 3H), 7.13-7.07 (m, 4H), 6.96 (d, J = 7.6 Hz, 2H), 6.85 (d, J = 8.0 Hz, 2H), 6.71 (d, J = 8.0 Hz, 2H), 6.67 (d, J = 8.0 Hz, 2H), 6.63 (s, 1H), 5.37 (d, J = 6.0 Hz, 1H), 4.76 (d, J = 6.0 Hz, 1H), 4.16-4.02 (m, 2H), 2.94-2.80 (m, 1H), 2.27 (s, 3H), 2.21 (s, 3H), 1.29-1.18 (m, 12H), 1.13 (d, J = 6.8 Hz, 6H). **^{13}C NMR** (100 MHz, $CDCl_3$) δ 152.8, 150.0, 139.9, 139.6, 137.2, 136.8, 135.1, 134.0, 133.4, 129.5, 129.3, 129.2, 128.7, 128.7, 128.6, 127.8, 127.6, 123.8, 64.5, 34.3, 30.0, 25.1, 24.8, 23.8, 23.8, 21.3, 21.2. **HRMS** (ESI) calcd. for $C_{38}H_{45}NNaO_2S$ ($[M+Na]^+$) 602.3063, Found 602.3063.

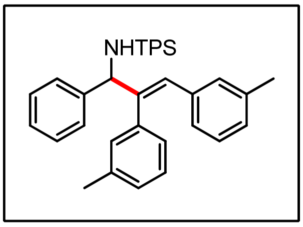

(*E*)-2,4,6-Triisopropyl-*N*-(1-phenyl-2,3-di-*m*-tolylallyl)benzenesulfonamide (4b)
Purified by column (PE/EtOAc = 50:1 to 30:1), white solid (84% yield). m.p. 50-52 °C. **^{1}H NMR** (400 MHz, $CDCl_3$) δ 7.21-7.14 (m, 3H), 7.10 (s, 2H), 7.09-7.05 (m, 2H), 7.04-6.98 (m, 2H), 6.94-6.85 (m, 2H), 6.68 (d, J = 7.6 Hz, 2H), 6.67 (s, 1H), 6.60-6.52 (m, 2H), 5.36 (d, J = 6.0 Hz, 1H), 4.78 (d, J = 6.0 Hz, 1H), 4.17-4.03 (m, 2H), 2.94-2.81 (m, 1H), 2.18 (s, 3H), 2.13 (s, 3H), 1.30-1.19 (m, 12H), 1.13 (d, J = 6.8 Hz, 6H). **^{13}C NMR** (100 MHz, $CDCl_3$) δ 152.8, 150.0, 140.8, 139.4, 138.2, 138.0, 137.3, 136.1, 133.9, 130.3, 129.9, 128.9, 128.5, 128.5, 128.4, 127.9, 127.8, 127.8, 127.6, 126.5, 126.2, 123.8, 64.5, 34.3, 30.0, 25.1, 24.8,

23.8, 23.7, 21.5, 21.4. **HRMS** (ESI) calcd. for $C_{38}H_{45}NNaO_2S$ ([M+Na]$^+$) 602.3063, Found 602.3061.

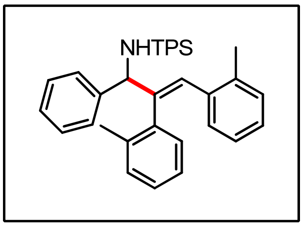

(*E*)-2,4,6-Triisopropyl-*N*-(1-phenyl-2,3-di-o-tolylallyl)benzenesulfonamide (4c)
Purified by column (PE/EtOAc = 50:1 to 30:1), white solid (79% yield, *Z*/*E* = 1:1). **^{1}H NMR** (400 MHz, $CDCl_3$) δ 7.20-7.09 (m, 9H), 7.09-7.06 (m, 6H), 7.06-7.00 (m, 5H), 7.00-6.92 (m, 2H), 6.92-6.80 (m, 5H), 6.77-6.66 (m, 2H), 6.63 (d, *J* = 7.6 Hz, 1H), 6.59 (d, *J* = 7.6 Hz, 1H), 6.49 (d, *J* = 8.0 Hz, 1H), 5.40 (d, *J* = 4.8 Hz, 1H), 5.12 (d, *J* = 5.2 Hz, 1H), 4.94 (d, *J* = 4.8 Hz, 1H), 4.91 (d, *J* = 5.6 Hz, 1H), 4.20-4.00 (m, 4H), 2.95-2.77 (m, 2H), 2.35 (s, 3H), 2.27 (s, 3H), 1.89 (s, 3H), 1.40 (s, 3H), 1.25 (d, *J* = 6.4 Hz, 6H), 1.24 (d, *J* = 6.8 Hz, 6H), 1.20 (d, *J* = 6.8 Hz, 6H), 1.17 (d, *J* = 6.8 Hz, 6H), 1.14 (d, *J* = 6.8 Hz, 6H), 1.05 (d, *J* = 6.8 Hz, 6H). **^{13}C NMR** (100 MHz, $CDCl_3$) δ 152.8, 152.8, 150.0, 149.8, 140.7, 140.3, 139.3, 139.2, 137.2, 137.0, 136.5, 136.5, 136.4, 135.9, 135.7, 135.5, 134.0, 133.5, 130.4, 130.3, 130.2, 129.7, 129.6, 128.6, 128.5, 128.4, 128.0, 127.8, 127.7, 127.6, 127.5, 127.4, 127.3, 127.0, 127.0, 126.7, 125.7, 125.3, 125.2, 125.2, 123.7, 65.0, 64.2, 34.3, 30.0, 29.9, 25.0, 24.8, 24.6, 23.8, 23.7, 23.7, 20.2, 20.1, 19.2, 18.9. **HRMS** (ESI) calcd. for $C_{38}H_{45}NNaO_2S$ ([M+Na]$^+$) 602.3063, Found 602.3074.

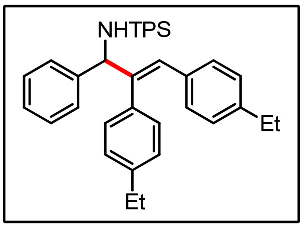

(*E*)-*N*-(2,3-Bis(4-ethylphenyl)-1-phenylallyl)-2,4,6-triisopropylbenzenesulfonamide (4d)
Purified by column (PE/EtOAc = 50:1 to 30:1), white solid (92% yield). m.p. 54-56 °C. **^{1}H NMR** (400 MHz, $CDCl_3$) δ 7.22-7.16 (m, 3H), 7.16-7.11 (m, 2H), 7.10 (s, 2H), 7.00 (d, *J* = 8.0 Hz, 2H), 6.88 (d, *J* = 8.0 Hz, 2H), 6.74 (d, *J* = 8.0 Hz, 2H), 6.70 (d, *J* = 8.0 Hz, 2H), 6.63 (s, 1H), 5.39 (d, *J* = 6.0 Hz, 1H), 4.77 (d, *J* = 6.0 Hz, 1H), 4.17-4.03 (m, 2H), 2.95-2.80 (m, 1H), 2.59 (q, *J* = 7.6 Hz, 2H), 2.51 (q, *J* = 7.6 Hz, 2H), 1.28-1.09 (m, 24H). **^{13}C NMR** (100 MHz, $CDCl_3$) δ 152.7, 150.0, 143.6, 143.2, 139.8, 139.6, 135.2, 134.0, 133.6, 129.3, 128.9, 128.5, 128.2, 127.8, 127.6, 127.5, 123.8, 64.6, 34.3, 30.0, 28.6, 28.6, 25.1, 24.8, 23.8, 23.8, 15.4, 15.4. **HRMS** (ESI) calcd. for $C_{40}H_{49}NNaO_2S$ ([M+Na]$^+$) 630.3376, Found 630.3372.

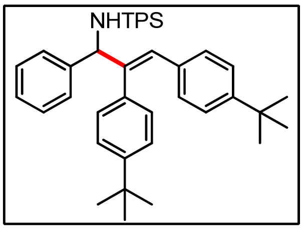

(*E*)-*N*-(2,3-Bis(4-(tert-butyl)phenyl)-1-phenylallyl)-2,4,6-triisopropylbenzenesulfonamide (4e)

Purified by column (PE/EtOAc = 50:1 to 30:1), white solid (88% yield). m.p. 140-142 °C. ^{1}**H NMR** (400 MHz, $CDCl_3$) δ 7.23-7.16 (m, 5H), 7.17-7.12 (m, 2H), 7.10 (s, 2H), 7.06 (d, *J* = 8.4 Hz, 2H), 6.74 (d, *J* = 8.4 Hz, 2H), 6.72 (d, *J* = 8.4 Hz, 2H), 6.62 (s, 1H), 5.40 (d, *J* = 6.4 Hz, 1H), 4.77 (d, *J* = 6.4 Hz, 1H), 4.15-4.02 (m, 2H), 2.94-2.81 (m, 1H), 1.28 (s, 9H), 1.24 (dd, *J* = 6.8, 1.6 Hz, 6H), 1.22 (s, 9H), 1.20 (d, *J* = 6.8 Hz, 6H), 1.13 (d, *J* = 6.8 Hz, 6H). ^{13}**C NMR** (100 MHz, $CDCl_3$) δ 152.7, 150.6, 150.1, 150.0, 139.8, 139.7, 134.8, 134.1, 133.3, 129.1, 129.0, 128.9, 128.5, 127.8, 127.6, 125.7, 124.9, 123.8, 64.6, 34.6, 34.6, 34.3, 31.4, 31.3, 30.0, 25.1, 24.9, 23.8, 23.8. **HRMS** (ESI) calcd. for $C_{44}H_{57}NNaO_2S$ ($[M+Na]^+$) 686.4002, Found 686.4003.

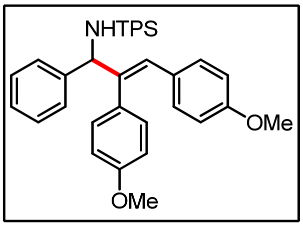

(*E*)-*N*-(2,3-Bis(4-methoxyphenyl)-1-phenylallyl)-2,4,6-triisopropylbenzenesulfonamide (4f)

Purified by column (PE/EtOAc = 50:1 to 10:1), white solid (92% yield). m.p. 64-66 °C. ^{1}**H NMR** (400 MHz, $CDCl_3$) δ 7.23-7.15 (m, 3H), 7.14-7.07 (m, 4H), 6.78-6.67 (m, 6H), 6.63-6.56 (m, 3H), 5.36 (d, *J* = 6.0 Hz, 1H), 4.76 (d, *J* = 6.0 Hz, 1H), 4.17-4.01 (m, 2H), 3.75 (s, 3H), 3.71 (s, 3H), 2.96-2.81 (m, 1H), 1.28-1.18 (m, 12H), 1.13 (d, *J* = 6.8 Hz, 6H). ^{13}**C NMR** (100 MHz, $CDCl_3$) δ 158.9, 158.5, 152.7, 150.0, 139.6, 138.3, 134.0, 130.7, 130.5, 130.2, 128.9, 128.5, 128.3, 127.8, 127.5, 123.8, 114.2, 113.4, 64.6, 55.2, 34.3, 30.0, 25.1, 24.8, 23.8, 23.7. **HRMS** (ESI) calcd. for $C_{38}H_{45}NNaO_4S$ ($[M+Na]^+$) 634.2962, Found 634.2965.

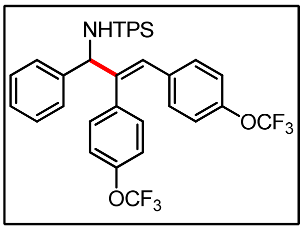

(*E*)-2,4,6-Triisopropyl-*N*-(1-phenyl-2,3-bis(4-(trifluoromethoxy)phenyl)allyl)benzenesulfonamide (4g)

Purified by column (PE/EtOAc = 50:1 to 20:1), white solid (60% yield). m.p. 122-124 °C. ^{1}**H NMR** (400 MHz, $CDCl_3$) δ 7.25-7.18 (m, 3H), 7.11 (s, 2H), 7.06-6.99 (m, 4H), 6.90 (d, *J* = 8.4 Hz, 2H), 6.85 (d, *J* = 8.4 Hz, 2H), 6.80 (s, 1H), 6.75 (d, *J* = 8.8 Hz, 2H), 5.32 (d, *J* = 5.2 Hz, 1H), 4.75 (d, *J* = 5.6 Hz, 1H), 4.16-4.01 (m, 2H), 2.94-2.81 (m, 1H), 1.29-1.19 (m, 12H), 1.09 (d, *J* = 6.4 Hz, 6H). ^{13}**C NMR** (100 MHz, $CDCl_3$) δ 153.1, 150.0, 148.9, 148.2, 140.5, 138.6, 136.6, 134.5, 133.8, 130.9, 130.6, 129.0, 128.5, 128.0, 127.6, 123.9, 121.2, 120.5 (q, *J* = 255.6 Hz), 120.5, 64.4, 34.3, 30.0, 25.1, 24.7, 23.8, 23.7. **HRMS** (ESI) calcd. for $C_{38}H_{39}F_6NNaO_4S$ ($[M+Na]^+$) 742.2396, Found 742.2388.

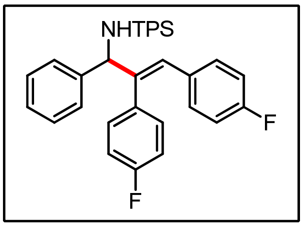

(*E*)-*N*-(2,3-Bis(4-fluorophenyl)-1-phenylallyl)-2,4,6-triisopropylbenzenesulfonamide (4h)

Purified by column (PE/EtOAc = 50:1 to 25:1), colorless semi-solid (87% yield). **^{1}H NMR** (400 MHz, $CDCl_3$) δ 7.24-7.16 (m, 3H), 7.11 (s, 2H), 7.07-6.99 (m, 2H), 6.87 (t, *J* = 8.8 Hz, 2H), 6.83-6.76 (m, 2H), 6.76-6.68 (m, 5H), 5.31 (d, *J* = 5.6 Hz, 1H), 4.75 (d, *J* = 5.6 Hz, 1H), 4.18-4.01 (m, 2H), 2.95-2.80 (m, 1H), 1.30-1.18 (m, 12H), 1.10 (d, *J* = 6.4 Hz, 6H). **^{13}C NMR** (100 MHz, $CDCl_3$) δ 162.2 (d, *J* = 245.8 Hz), 161.8 (d, *J* = 246.1 Hz), 153.0, 150.0, 139.7, 138.9, 133.9 (d, *J* = 3.4 Hz), 133.8, 132.0 (d, *J* = 3.3 Hz), 131.2 (d, *J* = 7.8 Hz), 130.9 (d, *J* = 7.8 Hz), 128.8, 128.3, 128.0, 127.6, 123.9, 115.8 (d, *J* = 21.2 Hz), 115.0 (d, *J* = 21.3 Hz), 64.5, 34.3, 30.0, 25.1, 24.7, 23.8, 23.7. **HRMS** (ESI) calcd. for $C_{36}H_{39}F_2NNaO_2S$ ($[M+Na]^+$) 610.2562, Found 610.2571.

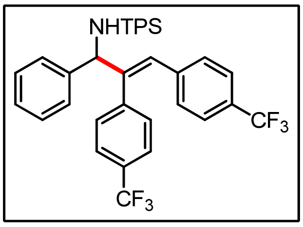

(*E*)-2,4,6-Triisopropyl-*N*-(1-phenyl-2,3-bis(4-(trifluoromethyl)phenyl)allyl)benzenesulfonamide (4i)

Purified by column (PE/EtOAc = 50:1 to 30:1), white solid (65% yield). m.p. 127-128 °C. **^{1}H NMR** (400 MHz, $CDCl_3$) δ 7.43 (d, *J* = 8.0 Hz, 2H), 7.31 (d, *J* = 8.4 Hz, 2H), 7.25-7.17 (m, 3H), 7.11 (s, 2H), 7.04-6.98 (m, 3H), 6.97 (s, 1H), 6.93-6.89 (m, 1H), 6.84 (d, *J* = 8.0 Hz, 2H), 5.33 (d, *J* = 5.2 Hz, 1H), 4.77 (d, *J* = 5.2 Hz, 1H), 4.18-4.03 (m, 2H), 2.94-2.80 (m, 1H), 1.26 (d, *J* = 6.8 Hz, 6H), 1.22 (dd, *J* = 6.8, 2.8 Hz, 6H), 1.09 (d, *J* = 6.8 Hz, 6H). **^{13}C NMR** (100 MHz, $CDCl_3$) δ 153.2, 150.0, 142.2, 141.9, 139.3, 138.2, 133.6, 131.3, 130.0 (q, *J* = 32.4 Hz), 129.7, 129.4, 129.1, 129.1 (q, *J* = 32.2 Hz), 128.6, 128.2, 127.6, 125.8 (q, *J* = 3.3 Hz), 125.0 (q, *J* = 3.6 Hz), 124.1 (q, *J* = 270.3 Hz), 124.0, 124.0 (q, *J* = 270.5 Hz), 64.3, 34.3, 30.0, 25.1, 24.6, 23.7, 23.6. **HRMS** (ESI) calcd. for $C_{38}H_{39}F_6NNaO_2S$ ($[M+Na]^+$) 710.2498, Found 710.2491.

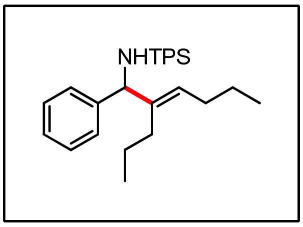

(*E*)-2,4,6-Triisopropyl-*N*-(1-phenyl-2-propylhex-2-en-1-yl)benzenesulfonamide (4j)

Purified by column (PE/EtOAc = 50:1 to 40:1), white solid (91% yield). m.p. 66-68 °C. **^{1}H NMR** (400 MHz, $CDCl_3$) δ 7.19-7.13 (m, 3H), 7.10 (s, 2H), 7.04-6.98 (m, 2H), 5.40 (t, *J* =

7.2 Hz, 1H), 4.90 (d, J = 6.0 Hz, 1H), 4.59 (d, J = 6.0 Hz, 1H), 4.11-3.95 (m, 2H), 2.86-2.82 (m, 1H), 2.01-1.84 (m, 3H), 1.74-1.61 (m, 1H), 1.33-1.18 (m, 4H), 1.25 (d, J = 6.8 Hz, 6H), 1.21 (d, J = 6.8 Hz, 6H), 1.15 (d, J = 6.8 Hz, 6H), 0.84 (t, J = 7.2 Hz, 3H), 0.77 (t, J = 7.2 Hz, 3H). **^{13}C NMR** (100 MHz, $CDCl_3$) δ 152.6, 149.8, 140.4 138.5, 134.0, 128.5, 128.4, 127.6, 127.4, 123.6, 61.9, 34.3, 31.3, 29.9, 29.8, 25.0, 24.8, 23.8, 23.7, 22.8, 21.9, 14.2, 14.0. **HRMS** (ESI) calcd. for $C_{30}H_{45}NNaO_2S$ ([M+Na]$^+$) 506.3063, Found 506.3069.

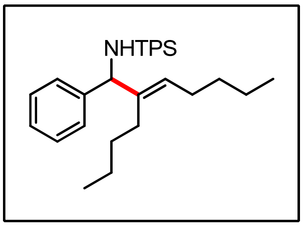

(*E*)-*N*-(2-Butyl-1-phenylhept-2-en-1-yl)-2,4,6-triisopropylbenzenesulfonamide (4k)
Purified by column (PE/EtOAc = 50:1 to 40:1), colorless semi-solid (84% yield). **^{1}H NMR** (400 MHz, $CDCl_3$) δ 7.19-7.13 (m, 3H), 7.10 (s, 2H), 7.03-6.97 (m, 2H), 5.38 (t, J = 7.2 Hz, 1H), 4.89 (d, J = 6.0 Hz, 1H), 4.58 (d, J = 6.0 Hz, 1H), 4.08-3.95 (m, 2H), 2.95-2.82 (m, 1H), 2.03-1.85 (m, 3H), 1.75-1.62 (m, 1H), 1.32-1.23 (m, 4H), 1.25 (d, J = 6.8 Hz, 6H), 1.20 (d, J = 6.8 Hz, 6H), 1.18-1.09 (m, 4H), 1.14 (d, J = 6.8 Hz, 6H), 0.86 (t, J = 6.8 Hz, 3H), 0.78 (t, J = 7.0 Hz, 3H). **^{13}C NMR** (100 MHz, $CDCl_3$) δ 152.6, 149.8, 140.4, 138.5, 134.0, 128.4, 128.4, 127.5, 127.4, 123.6, 61.8, 34.2, 31.8, 30.9, 29.9, 28.9, 27.4, 25.0, 24.8, 23.8, 23.7, 22.9, 22.5, 14.0, 13.9. **HRMS** (ESI) calcd. for $C_{32}H_{49}NNaO_2S$ ([M+Na]$^+$) 534.3376, Found 534.3378.

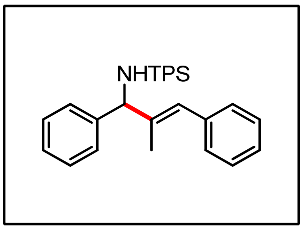

(*E*)-2,4,6-Triisopropyl-*N*-(2-methyl-1,3-diphenylallyl)benzenesulfonamide
(*E*)-*N*-(1,2-diphenylbut-2-en-1-yl)-2,4,6-triisopropylbenzenesulfonamide (8.1 : 1 mixture) (4l)
Purified by column (PE/EtOAc = 50:1 to 30:1), colorless semi-solid (67% yield). **^{1}H NMR** (400 MHz, $CDCl_3$) δ 7.29-7.23 (m, 4H), 7.23-7.16 (m, 4H), 7.14 (s, 2H), 6.96 (d, J = 6.8 Hz, 2H), 6.51 (s, 1H), 5.10 (d, J = 5.6 Hz, 1H), 4.76 (d, J = 5.6 Hz, 1H), 4.14-3.99 (m, 2H), 2.98-2.83 (m, 1H), 1.65 (d, J = 0.8 Hz, 3H), 1.30-1.22 (m, 12H), 1.14 (d, J = 6.8 Hz, 6H). **^{13}C NMR** (100 MHz, $CDCl_3$) δ 152.8, 150.0, 139.6, 137.1, 135.9, 134.1, 129.0, 128.9, 128.1, 127.6, 127.4, 126.7, 123.8, 64.6, 34.3, 30.1, 25.1, 24.8, 23.8, 23.8, 15.9. **HRMS** (ESI) calcd. for $C_{31}H_{39}NNaO_2S$ ([M+Na]$^+$) 512.2594, Found 512.2586.

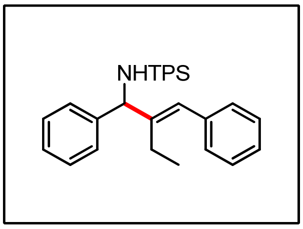

(*E*)-*N*-(2-Benzylidene-1-phenylbutyl)-2,4,6-triisopropylbenzenesulfonamide (*E*)-*N*-(1,2-diphenylpent-2-en-1-yl)-2,4,6-triisopropylbenzenesulfonamide (20 : 1 mixture) (4m)

Purified by column (PE/EtOAc = 50:1 to 30:1), colorless semi-solid (67% yield). **^{1}H NMR** (400 MHz, $CDCl_3$) δ 7.30-7.26 (m, 1H), 7.25-7.16 (m, 5H), 7.16-7.09 (m, 4H), 7.01 (d, J = 7.6 Hz, 2H), 6.54 (s, 1H), 5.18 (d, J = 5.6 Hz, 1H), 4.69 (d, J = 5.6 Hz, 1H), 4.15-3.98 (m, 2H), 2.98-2.83 (m, 1H), 2.38-2.24 (m, 1H), 1.99-1.84 (m, 1H), 1.31-1.22 (m, 12H), 1.12 (d, J = 6.8 Hz, 6H), 0.92 (t, J = 7.6 Hz, 3H). **^{13}C NMR** (100 MHz, $CDCl_3$) δ 152.7, 149.8, 142.3, 139.9, 137.2, 134.3, 128.8, 128.7, 128.2, 128.1, 127.7, 127.1, 126.7, 123.8, 61.8, 34.3, 30.1, 25.1, 24.7, 23.8, 23.8, 22.9, 13.0. **HRMS** (ESI) calcd. for $C_{32}H_{41}NNaO_2S$ ([M+Na]$^+$) 526.2750, Found 526.2755.

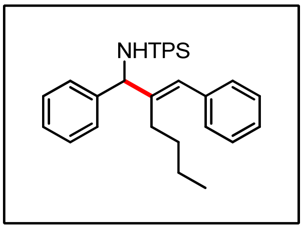

(*E*)-*N*-(2-Benzylidene-1-phenylhexyl)-2,4,6-triisopropylbenzenesulfonamide (4n)

Purified by column (PE/EtOAc = 50:1 to 30:1), colorless semi-solid (70% yield). **^{1}H NMR** (400 MHz, $CDCl_3$) δ 7.29-7.26 (m, 1H), 7.25-7.16 (m, 5H), 7.16-7.09 (m, 4H), 7.02 (d, J = 7.2 Hz, 2H), 6.55 (s, 1H), 5.13 (d, J = 5.6 Hz, 1H), 4.69 (d, J = 6.0 Hz, 1H), 4.13-3.99 (m, 2H), 2.98-2.83 (m, 1H), 2.29-2.16 (m, 1H), 1.90-1.78 (m, 1H), 1.35-1.28 (m, 2H), 1.27 (d, J = 6.4 Hz, 6H), 1.25 (d, J = 6.4 Hz, 6H), 1.20-1.14 (m, 2H), 1.12 (d, J = 6.8 Hz, 6H), 0.76 (t, J = 7.2 Hz, 3H). **^{13}C NMR** (100 MHz, $CDCl_3$) δ 152.7, 149.8, 141.4, 140.0, 137.3, 134.2, 128.8, 128.7, 128.1, 128.0, 127.6, 127.4, 126.6, 123.8, 62.0, 34.3, 30.4, 30.0, 29.6, 25.1, 24.7, 23.8, 23.8, 22.8, 13.8. **HRMS** (ESI) calcd. for $C_{34}H_{45}NNaO_2S$ ([M+Na]$^+$) 554.3063, Found 554.3057.

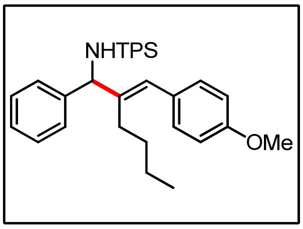

(*E*)-2,4,6-Triisopropyl-*N*-(2-(4-methoxybenzylidene)-1-phenylhexyl)benzenesulfonamide (4o)

Purified by column (PE/EtOAc = 50:1 to 10:1), white solid (88% yield). m.p. 87-89 °C. **^{1}H NMR** (400 MHz, $CDCl_3$) δ 7.25-7.19 (m, 3H), 7.17-7.09 (m, 4H), 6.96 (d, J = 8.8 Hz, 2H),

6.80 (d, *J* = 8.8 Hz, 2H), 6.45 (s, 1H), 5.12 (d, *J* = 5.6 Hz, 1H), 4.68 (d, *J* = 6.0 Hz, 1H), 4.13-4.00 (m, 2H), 3.79 (s, 3H), 2.97-2.83 (m, 1H), 2.26-2.12 (m, 1H), 1.93-1.80 (m, 1H), 1.35-1.22 (m, 2H), 1.27 (d, *J* = 6.8 Hz, 6H), 1.24 (d, *J* = 6.4 Hz, 6H), 1.21-1.15 (m, 2H), 1.13 (d, *J* = 6.4 Hz, 6H), 0.77 (t, *J* = 7.2 Hz, 3H). **^{13}C NMR** (100 MHz, $CDCl_3$) δ 158.3, 152.7, 149.9, 140.2, 139.9, 134.3, 129.9, 129.8, 128.7, 128.0, 127.6, 127.0, 123.8, 113.6, 62.2, 55.3, 34.3, 30.4, 30.0, 29.7, 25.1, 24.7, 23.8, 23.8, 22.9, 13.9. **HRMS** (ESI) calcd. for $C_{35}H_{47}NNaO_3S$ ($[M+Na]^+$) 584.3169, Found 584.3174.

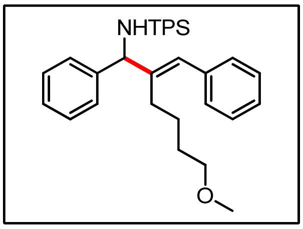

(*E*)-*N*-(2-Benzylidene-6-methoxy-1-phenylhexyl)-2,4,6-triisopropylbenzenesulfonamide (4p)

Purified by column (PE/EtOAc = 50:1 to 20:1), colorless semi-solid (50% yield). **^{1}H NMR** (400 MHz, $CDCl_3$) δ 7.30-7.16 (m, 6H), 7.15-7.08 (m, 4H), 7.02 (d, *J* = 7.6 Hz, 2H), 6.57 (s, 1H), 5.14 (d, *J* = 5.6 Hz, 1H), 4.74 (d, *J* = 6.0 Hz, 1H), 4.13-3.98 (m, 2H), 3.25 (s, 3H), 3.20 (t, *J* = 6.0 Hz, 2H), 2.97-2.82 (m, 1H), 2.33-2.19 (m, 1H), 1.96-1.81 (m, 1H), 1.49-1.35 (m, 4H), 1.26 (d, *J* = 6.8 Hz, 6H), 1.24 (d, *J* = 6.8 Hz, 6H), 1.12 (d, *J* = 6.4 Hz, 6H). **^{13}C NMR** (100 MHz, $CDCl_3$) δ 152.8, 149.9, 141.1, 139.9, 137.3, 134.3, 128.8, 128.7, 128.2, 128.1, 127.8, 127.6, 126.7, 123.8, 72.2, 62.0, 58.6, 34.3, 30.0, 29.5, 29.4, 25.1, 24.7, 23.8, 23.8. **HRMS** (ESI) calcd. for $C_{35}H_{47}NNaO_3S$ ($[M+Na]^+$) 584.3169, Found 584.3170.

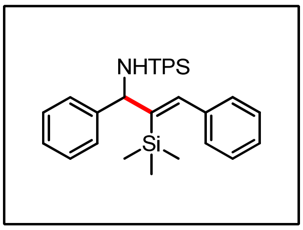

(*Z*)-*N*-(1,3-Diphenyl-2-(trimethylsilyl)allyl)-2,4,6-triisopropylbenzenesulfonamide (4q)

Purified by column (PE/EtOAc = 50:1 to 40:1), white solid (55% yield). m.p. 105-107 °C. **^{1}H NMR** (400 MHz, $CDCl_3$) δ 7.48 (s, 1H), 7.26-7.20 (m, 6H), 7.17 (s, 2H), 7.10-7.04 (m, 2H), 6.96-6.89 (m, 2H), 5.42 (d, *J* = 6.4 Hz, 1H), 4.58 (d, *J* = 6.4 Hz, 1H), 4.15-3.97 (m, 2H), 3.02-2.86 (m, 1H), 1.30 (dd, *J* = 6.8, 1.6 Hz, 6H), 1.27 (d, *J* = 6.8 Hz, 6H), 1.10 (d, *J* = 6.8 Hz, 6H), -0.29 (s, 9H). **^{13}C NMR** (100 MHz, $CDCl_3$) δ 152.7, 149.7, 141.9, 141.8, 140.5, 139.7, 134.9, 128.9, 128.6, 128.5, 128.2, 127.9, 127.2, 123.9, 62.2, 34.4, 30.1, 25.1, 24.8, 23.9, 23.9, 0.4. **HRMS** (ESI) calcd. for $C_{33}H_{45}NNaO_2SSi$ ($[M+Na]^+$) 570.2832, Found 570.2837.

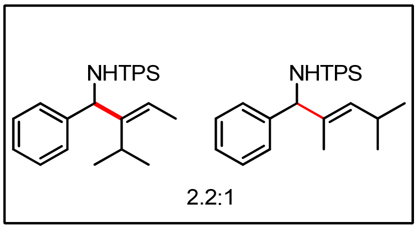

(*E*)-2,4,6-Triisopropyl-*N*-(2-isopropyl-1-phenylbut-2-en-1-yl)benzenesulfonamide (*E*)-*N*-(2,4-dimethyl-1-phenylpent-2-en-1-yl)-2,4,6-triisopropylbenzenesulfonamide (2.2 : 1 mixture) (4r)

Purified by column (PE/EtOAc = 50:1 to 40:1), colorless oil (69% yield). **^{1}H NMR** (400 MHz, $CDCl_3$) δ 7.23-7.18 (m, 1.38H), 7.15-7.09 (m, 4.92H), 7.08 (s, 2H), 7.01-6.94 (m, 1.92H), 5.46 (q, J = 6.8 Hz, 1H), 5.21 (d, J = 9.2 Hz, 0.46H), 5.05 (d, J = 5.6 Hz, 1H), 4.88 (d, J = 6.0 Hz, 0.46H), 4.64 (d, J = 6.4 Hz, 0.46H), 4.52 (d, J = 5.6 Hz, 1H), 4.10-3.93 (m, 2.92H), 2.96-2.81 (m, 1.46H), 2.76-2.62 (m, 1H), 2.47-2.34 (m, 0.46H), 1.60 (d, J = 6.8 Hz, 3H), 1.42 (s, 1.38H), 1.30-1.21 (m, 12H), 1.21-1.15 (m, 8.52H), 1.13 (d, J = 6.8 Hz, 6H), 1.02 (d, J = 7.2 Hz, 3H), 0.82 (d, J = 6.4 Hz, 1.38H), 0.78 (d, J = 7.2 Hz, 2.76H), 0.75 (d, J = 6.8 Hz, 1.38H). **^{13}C NMR** (100 MHz, $CDCl_3$) δ 152.6, 152.6, 149.9, 149.7, 144.3, 141.1, 140.0, 135.6, 134.4, 134.1, 130.8, 128.6, 128.4, 127.7, 127.5, 127.5, 127.3, 123.8, 123.6, 122.7, 63.9, 59.3, 34.3, 30.0, 30.0, 28.9, 27.0, 25.0, 24.9, 24.7, 23.8, 23.8, 22.8, 22.6, 21.5, 20.8, 13.9, 13.3. **HRMS** (ESI) calcd. for $C_{28}H_{41}NNaO_2S$ ($[M+Na]^+$) 478.2750, Found 478.2746.

Supplementary Note 4

Gram-scale reaction and product transformation

Gram-scale reaction

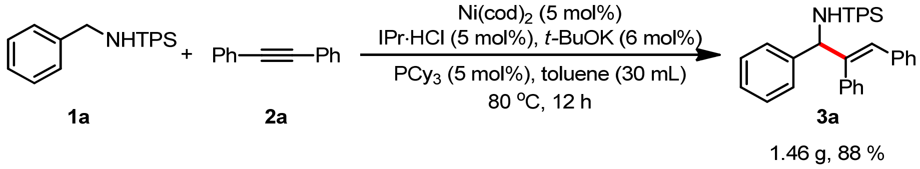

To a 48 mL pressure tube were added $Ni(cod)_2$ (41.3 mg, 0.15 mmol), IPr·HCl (63.8 mg, 0.15 mmol), PCy_3 (42.1 mg, 0.15 mmol), KO^{t}Bu (20.2 mg, 0.18 mmol), toluene (30 mL), **1a** (3 mmol, 1.12 g) and **2a** (3.3 mmol, 588.2 mg) in a glove box. The tube was sealed with a Teflon cap and the mixture was stirred at 80 °C for 12 h. After cooled to room temperature, the crude product was filtered through a short pad of Celite, and the filtrate was concentrated under vacuum. The resulting residue was obtained by chromatography on silica gel column with petroleum ether/ethyl acetate as the eluent to afford pure product **3a** (88% yield, 1.46 g).

Transformation of product 3a

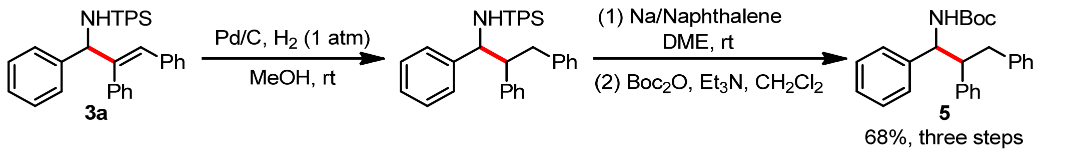

The solution of compound **3a** (0.21 mmol) and Pd/C (contain 10% Pd) (5 mol%) in CH_3OH (5 mL) was stirred under 1 atm pressure of hydrogen at room temperature for 4 hours. The mixture was filtered through a short pad of Silica gel, and the filtrate was concentrated under vacuum to obtain a crude product, which was pure enough for next step without further purification. To a solution of the crude product in DME (2 mL), sodium-naphthalene (8 equiv) was added dropwise. The reaction was stirred at room temperature for 5 h then quenched with water. The aqueous and organic layers were separated and the aqueous layer was extracted using CH_2Cl_2. The combined organic layers were dried over anhydrous Na_2SO_4, filtered, and concentrated *in vacuo*. Then Boc_2O (59.6 mg, 1.3 equiv), Et_3N (88 μL, 3 equiv) and CH_2Cl_2 (2 mL) were added and stirred for 12 h at room temperature. The mixture was washed with saturated NH_4Cl, dried over anhydrous Na_2SO_4 and concentrated *in vacuo*. The residue was purified by silica column chromatography with petroleum ether/ethyl acetate to give compound **5** in 68% yield (55 mg, three steps). White solid. m.p. 178-179 °C. **^{1}H NMR** (400 MHz, $CDCl_3$) δ 7.26 (s, 1H), 7.22-7.15 (m, 3H), 7.15-7.04 (m, 5H), 6.98 (d, J = 7.2 Hz, 4H), 6.88 (d, J = 7.6 Hz, 2H), 4.99 (s, 2H), 3.38-3.28 (m, 1H), 3.24 (d, J = 16.4 Hz, 1H), 2.93 (dd, J = 13.6, 10.4 Hz, 1H), 1.43 (s, 9H). **^{13}C NMR** (100 MHz, $CDCl_3$) δ 155.4, 140.6, 140.1, 129.2, 129.1, 128.2, 128.1, 128.0, 127.5, 127.2, 126.6, 125.9, 79.7, 59.4, 53.5, 38.8, 28.5. **HRMS** (ESI) calcd. for $C_{26}H_{30}NO_2$ ($[M+H]^+$) 388.2271, Found 388.2269. Note: For sodium-naphthalene preparation, a flame-dried 50 mL round bottom flask was charged with a stir bar, naphthalene (790 mg, 6.16 mmol), DME (6 mL), and small pieces of sodium metal (118 mg, 5.13 mmol). The mixture was allowed to vigorously stir overnight for approximately 12 h, resulting into a dark green solution.

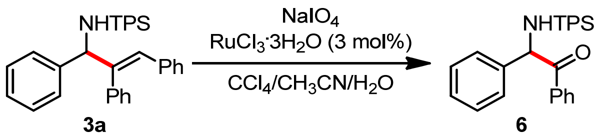

To a solution of **3a** (105 mg, 0.19 mmol) in a mixed solvent $CCl_4/CH_3CN/H_2O$ (1:1:1.6, v/v/v, 1.1 mL) was added $NaIO_4$ (165 mg, 0.77 mmol), and the mixture was stirred for 5 min before the addition of $RuCl_3{\cdot}3H_2O$ (1.5 mg, 0.0056 mmol). The resulting brown mixture was stirred for 24 h and quenched with 1N HCl. The mixture was extracted with CH_2Cl_2 and the extract was concentrated. The residue was purified by silica column chromatography with petroleum ether/ethyl acetate to give product **6** in 90% yield (82 mg). White solid. m.p. 115-117 °C. **^{1}H NMR** (400 MHz, $CDCl_3$) δ 7.82 (d, J = 7.6 Hz, 2H), 7.49 (t, J = 7.4 Hz, 1H), 7.35 (t, J = 7.8 Hz, 2H), 7.19-7.08 (m, 5H), 7.03 (s, 2H), 6.25 (d, J = 6.8 Hz, 1H), 6.04 (d, J = 7.2 Hz, 1H), 4.20-4.02 (m, 2H), 2.91-2.75 (m, 1H), 1.23 (d, J = 6.8 Hz, 6H), 1.20 (d, J = 7.2 Hz, 6H), 1.16 (d, J = 6.8 Hz, 6H). **^{13}C NMR** (100 MHz, $CDCl_3$) δ 194.9, 152.9, 150.1, 136.2, 134.0, 133.2, 129.1, 128.8, 128.5, 128.1, 123.6, 61.3, 34.2, 29.9, 24.9, 24.9, 23.7, 23.7.

HRMS (ESI) calcd. for $C_{29}H_{36}NO_3S$ ($[M+H]^+$) 478.2410, Found 478.2407.

Supplementary Note 5

Mechanistic experiments

Deuterium labeling experiments

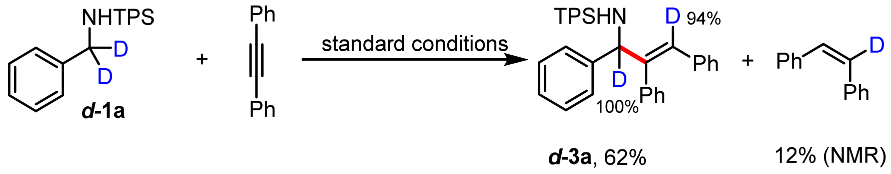

To a 15 mL pressure tube were added Ni(cod)$_2$ (2.75 mg, 0.01 mmol), IPr·HCl (4.3 mg, 0.01 mmol), PCy$_3$ (2.8 mg, 0.01 mmol), KO^{t}Bu (1.4 mg, 0.012 mmol), toluene (2 mL), ***d*-1a** (0.20 mmol, 75.1 mg) and **2a** (0.22 mmol, 39.2 mg) in a glove box. The tube was sealed with a Teflon cap and the mixture was stirred at 80 °C for 12 h. After cooled to room temperature, the crude product was filtered through a short pad of Celite, and the filtrate was concentrated under vacuum. The resulting residue was obtained by chromatography on silica gel column with petroleum ether/ethyl acetate as the eluent to afford pure product ***d*-3a** (62% yield, 68.1 mg).

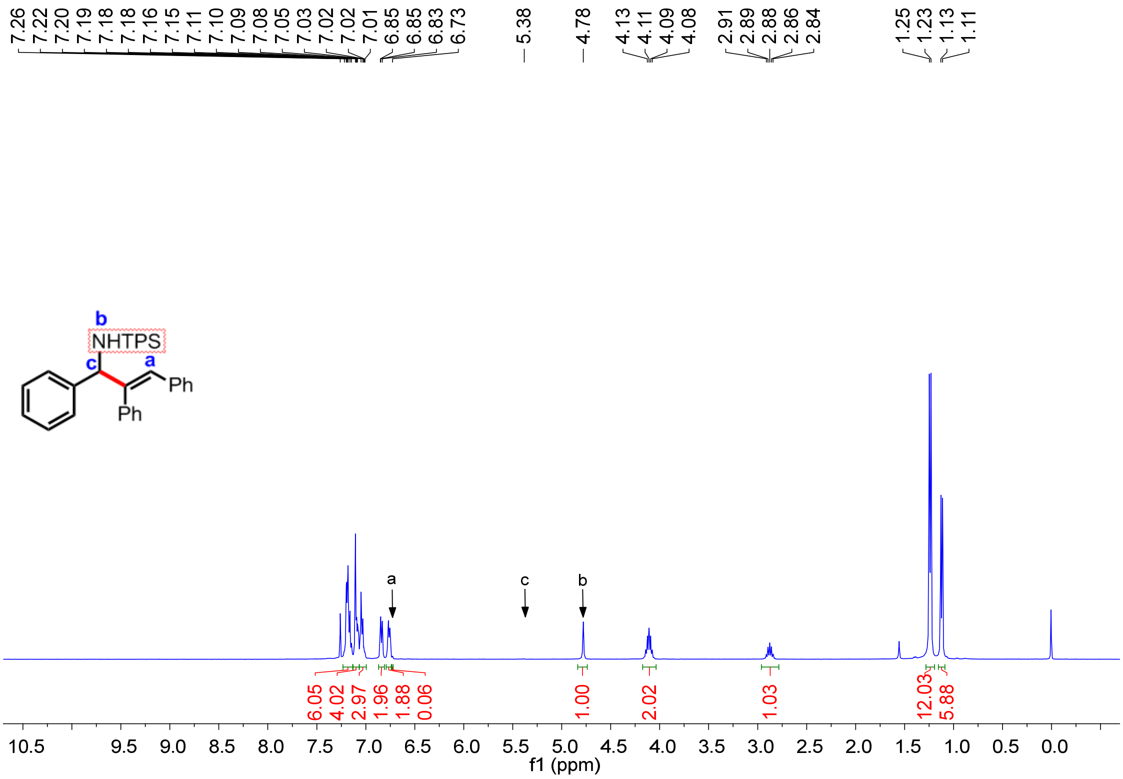

Supplementary Figure 1. Deuterium labeling experiment.

KIE determination

Competitive experiment

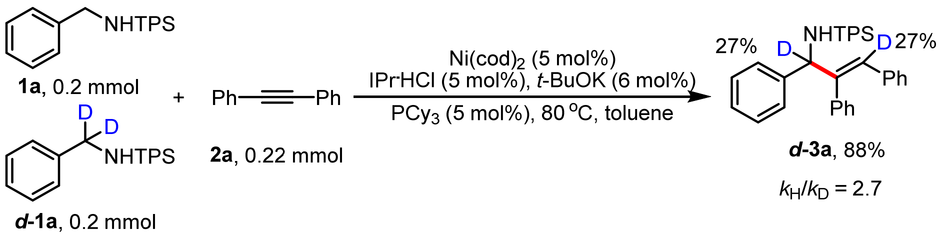

To a 15 mL pressure tube were added Ni(cod)$_2$ (2.75 mg, 0.01 mmol), IPr·HCl (4.3 mg, 0.01 mmol), PCy$_3$ (2.8 mg, 0.01 mmol), KO^{t}Bu (1.4 mg, 0.012 mmol), toluene (2 mL), ***d*-1a** (0.20 mmol, 75.1 mg), **1a** (0.20 mmol, 74.7 mg) and **2a** (0.22 mmol, 39.2 mg) in a glove box. The tube was sealed with a Teflon cap and the mixture was stirred at 80 °C for 12 h. After cooled to room temperature, the crude product was filtered through a short pad of Celite, and the filtrate was concentrated under vacuum. The resulting residue was obtained by chromatography on silica gel column with petroleum ether/ethyl acetate as the eluent to afford pure product ***d*-3a** (88% yield, 97.6 mg).

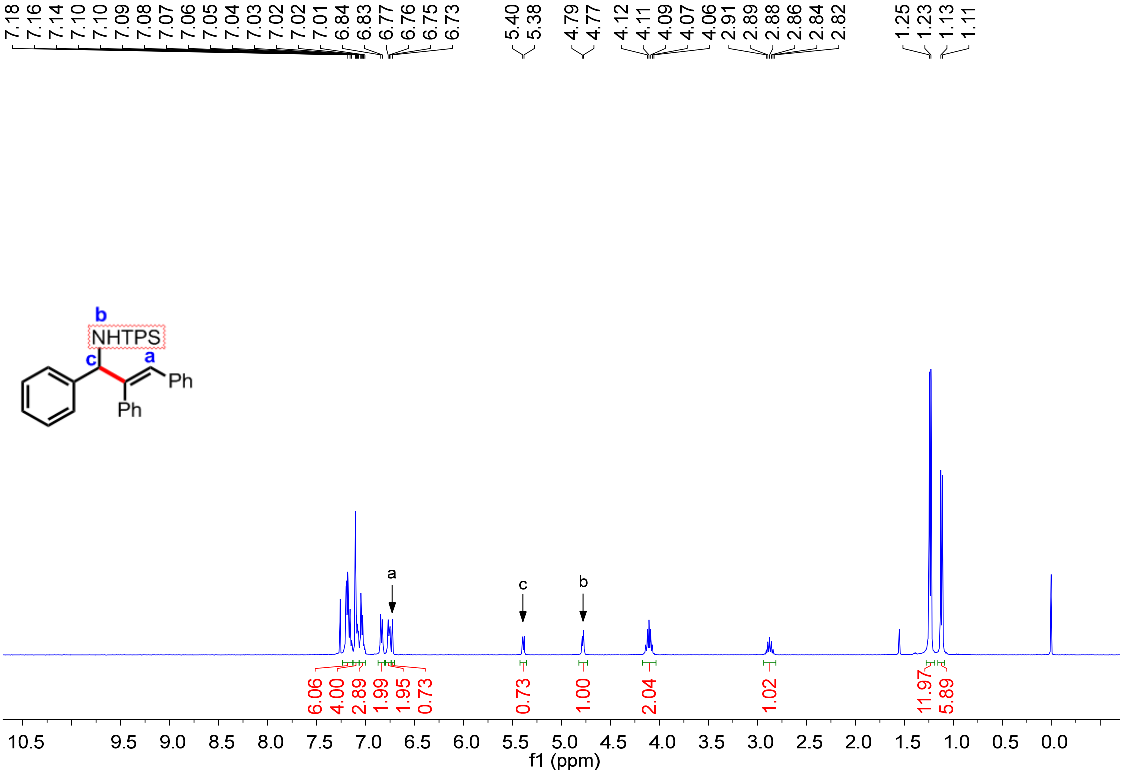

Supplementary Figure 2. Competitive experiment.

Parallel experiments

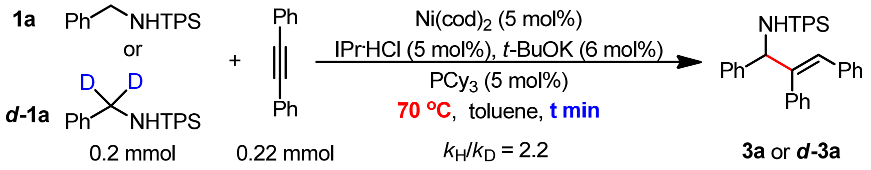

Six side-by-side reactions were conducted following the general procedure for the redox-neutral coupling of alkynes with **1a** (0.2 mmol) and ***d*-1a** (0.2 mmol), respectively. Aliquots were taken at 10 minute intervals in the first hour. Product yields were determined by ^{1}H NMR using $Cl_2CHCHCl_2$ as the internal standard. Data points represent the average of two runs.

time/min	0	10	20	30	40	50	60
yield of **3a**/%	0	8	11	16	22	29	32
yield of ***d*-3a**/%	0	2	5	7	11	12	14

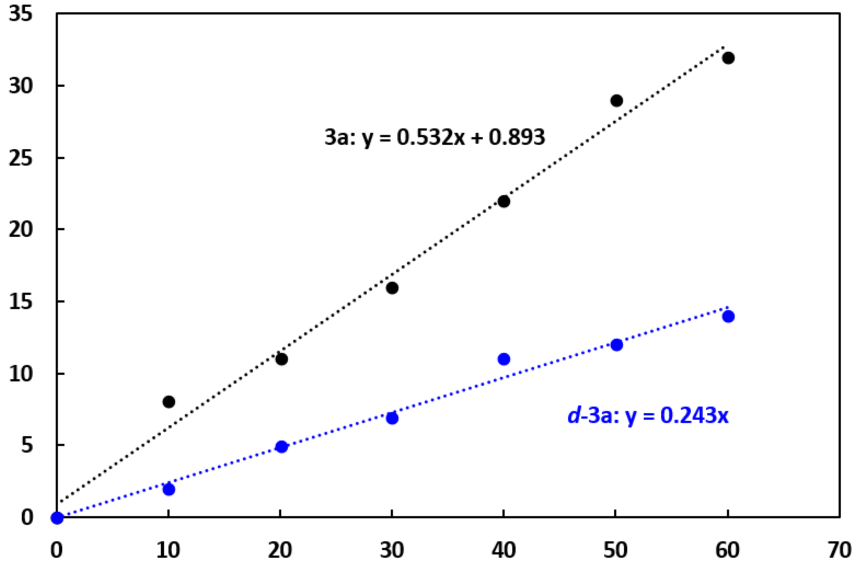

Supplementary Figure 3. KIE determination via parallel experiments.

Competitive experiments

Crossover experiments

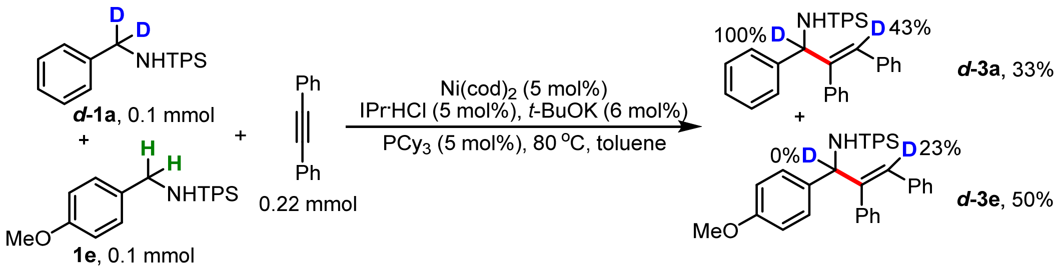

To a 15 mL pressure tube were added $Ni(cod)_2$ (2.75 mg, 0.01 mmol), IPr·HCl (4.3 mg, 0.01 mmol), PCy_3 (2.8 mg, 0.01 mmol), KO^{t}Bu (1.4 mg, 0.012 mmol), toluene (2 mL), ***d*-1a** (0.1 mmol, 37.6 mg), **1e** (0.1 mmol, 40.4 mg) and **2a** (0.22 mmol, 39.2 mg) in a glove box.

The tube was sealed with a Teflon cap and the mixture was stirred at 80 °C for 12 h. After cooled to room temperature, the crude product was filtered through a short pad of Celite, and the filtrate was concentrated under vacuum. The resulting residue was obtained by chromatography on silica gel column with petroleum ether/ethyl acetate as the eluent to afford pure product ***d*-3a** (33% yield, 36.6 mg) and ***d*-3e** (50% yield, 57.7 mg).

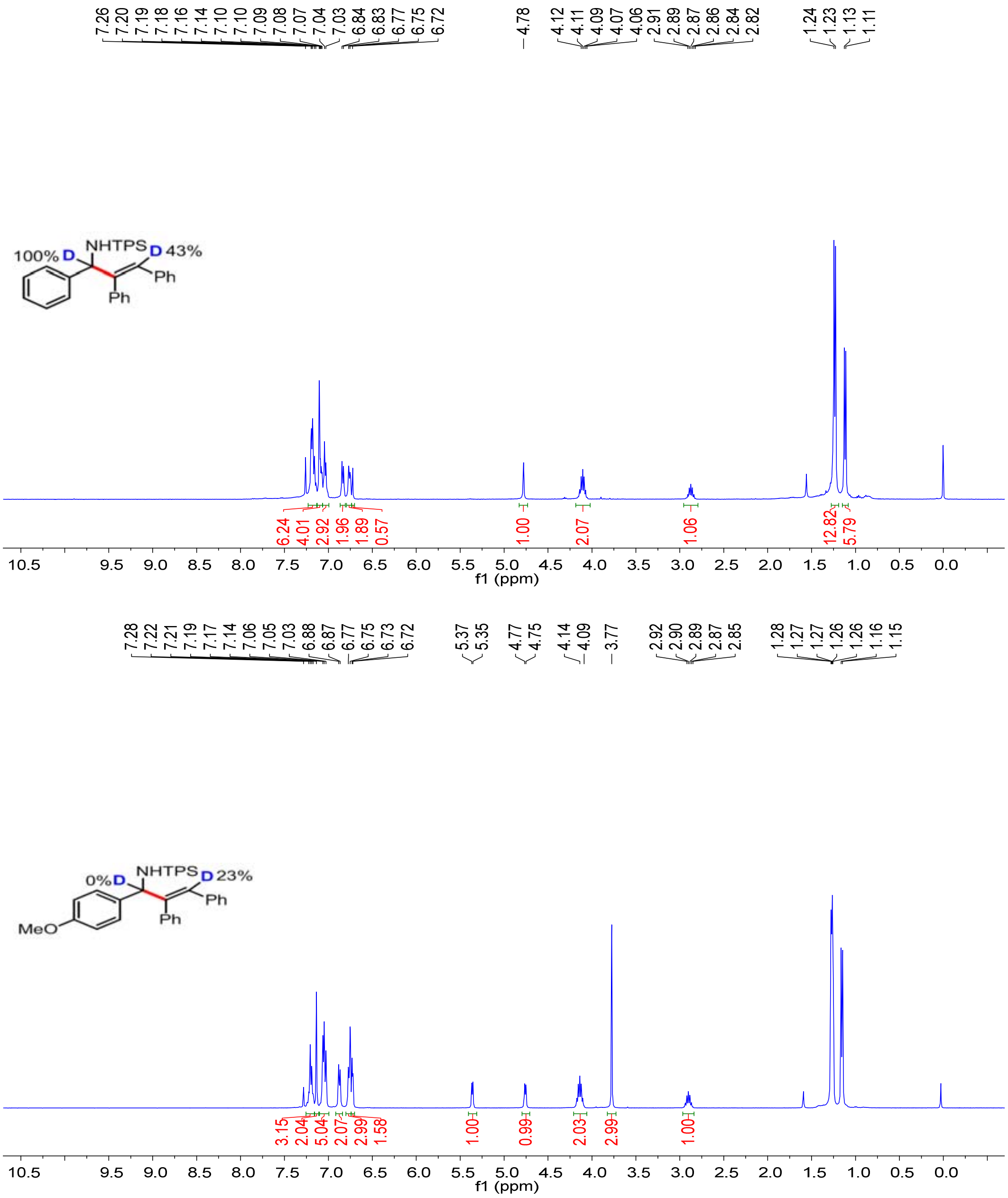

Supplementary Figure 4. ^{1}H NMR spectra of crossover experiments.

Electronic effect

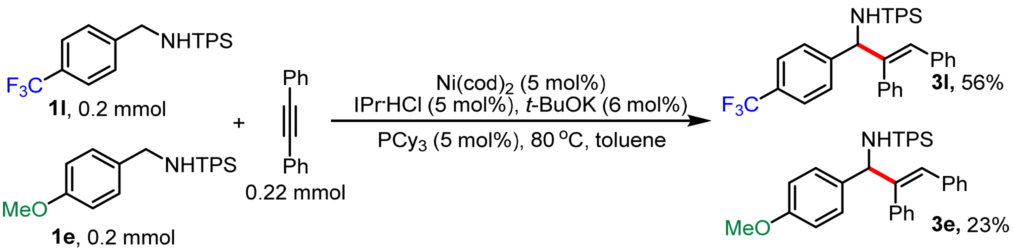

To a 15 mL pressure tube were added Ni(cod)2 (2.75 mg, 0.01 mmol), IPr·HCl (4.3 mg, 0.01 mmol), PCy3 (2.8 mg, 0.01 mmol), KO^{t}Bu (1.4 mg, 0.012 mmol), toluene (2 mL), **1l** (0.20 mmol, 88.3 mg), **1e** (0.2 mmol, 80.7 mg) and **2a** (0.22 mmol, 39.2 mg) in a glove box. The tube was sealed with a Teflon cap and the mixture was stirred at 80 °C for 12 h. After cooled to room temperature, the crude product was filtered through a short pad of Celite, and the filtrate was concentrated under vacuum. The resulting residue was obtained by chromatography on silica gel column with petroleum ether/ethyl acetate as the eluent to afford pure product **3l** (56% yield, 69.7 mg) and **3e** (23% yield, 26.5 mg). This experiment suggested electron-deficient group on the amide had a positive effect to the reaction rate.

Role of imine

Detection of imine

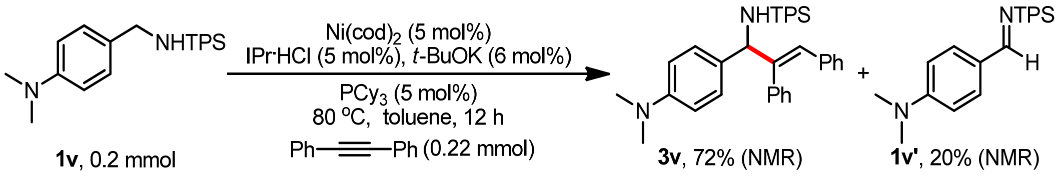

Under the standard conditions, the reaction of substrate **1v** with diphenylethyne gave the desired product **3v** along with small amounts of imine **1v′** that was confirmed by ^{1}H NMR using Cl2CHCHCl2 as the internal standard. In all other examined reactions, the crude NMR also showed the presence of imines, but which were trace amounts and cannot be isolated.

Competitive experiment of imine and amide

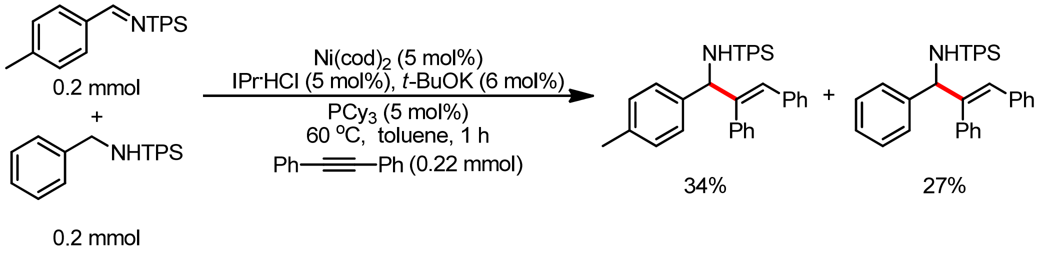

The reaction of 1:1 of the imine and the amide with alkyne was run for 1 h under the standard conditions but at 60 °C (hoping to achieve a lower conversion), and the crude product was detected by ^{1}H NMR using Cl2CHCHCl2 as the internal standard.

Effects of imine as additive in reaction of Ts-protected amide

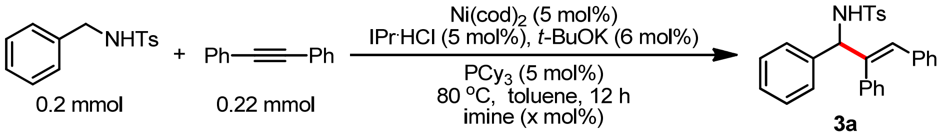

entry		imine (x mol%)	yield of **3a** (%)
1		0	13
2	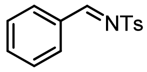	10	14
3	imine	20	13
4		50	13

The reaction of Ts-amide and alkyne was selected to detect the effects of imine because the reaction in general gave low yields under the standard conditions. But whatever loading of imine, no acceleration was observed, suggesting the generation of the imine at the induction stage was not the rate-determinating step.

Reaction of nickelacycle

Preparation of five-membered nickelacycle with PCy_3 as a ligand

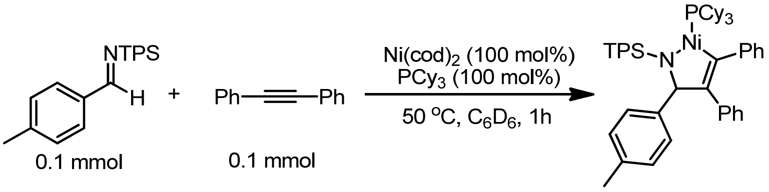

According to the literature,[8] the nickelacycle was prepared from the imine and alkyne, and the corresponding spectrum of ^{1}H and ^{31}P NMR was shown below (Supplementary Figure).

Reaction of five-membered nickelacycle with amide

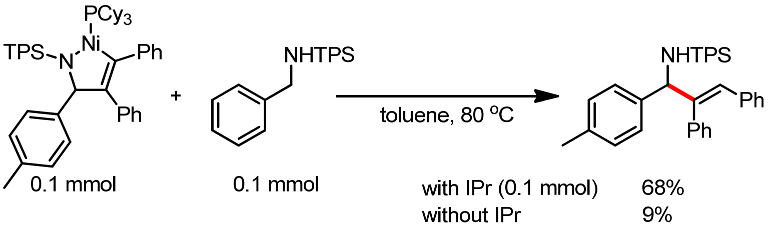

The reaction of nickelacycle (0.1 mmol) was treated with amide (0.1 mmol) at 80 °C. With the IPr ligand, the desired product was obtained in 68% yield. While without the ligand, only 9% of product was afforded.

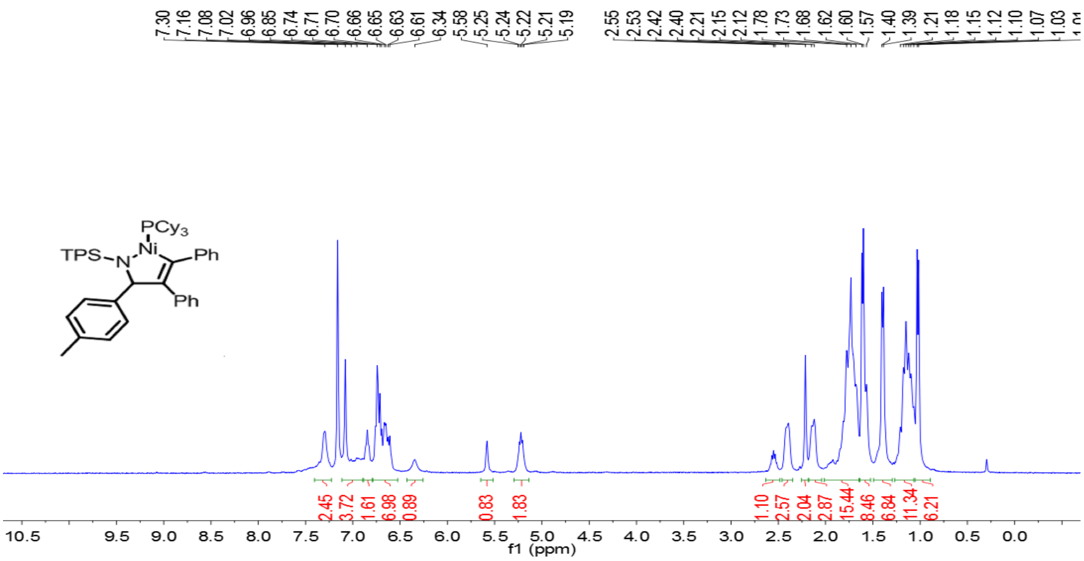

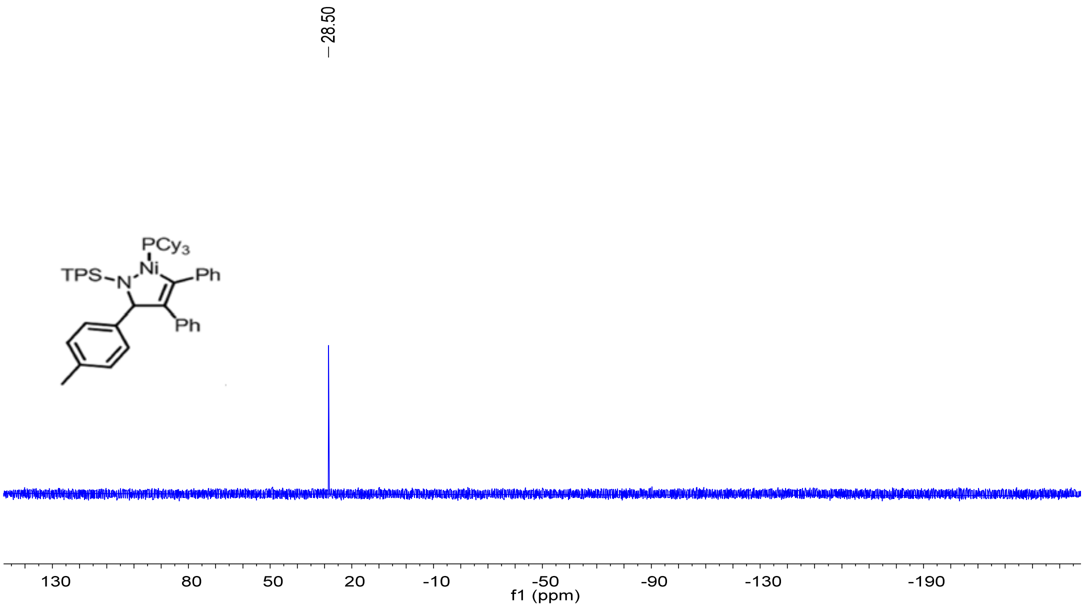

Supplementary Figure 5. ^{1}H and ^{31}P NMR spectra of nickelacycle.

Supplementary Note 6

DFT calculations

Computational methods

All DFT calculations were carried out with Gaussian 09.[9] Geometry optimizations were performed at the B3LYP-D3/LANL2DZ(Ni)-6-31G(d) level (pruned integration grids with 75 radial shells and 302 angular points per shell were used).[10–14] Unscaled harmonic frequency calculations at the same level were performed to validate each structure as either a minimum or a transition state and to evaluate its zero-point energy and thermal corrections at 298 K. Quasiharmonic corrections were applied during the entropy calculations by setting all positive frequencies that are less than 100 cm^{-1} to 100 cm^{-1}.[15,16] On the basis of the optimized structures, single-point energies were computed at the SMD(toluene)/M06-D3/SDD(Ni)/

6-311++G(d,p) level (pruned integration grids with 99 radial shells and 590 angular points per shell were used).[12,14,17–19] All discussed energy differences were based on Gibbs energies at 298 K unless otherwise specified. Standard states are the hypothetical states at 1 mol/L.

Gibbs energy profiles of the induction stage and the product-formation stage

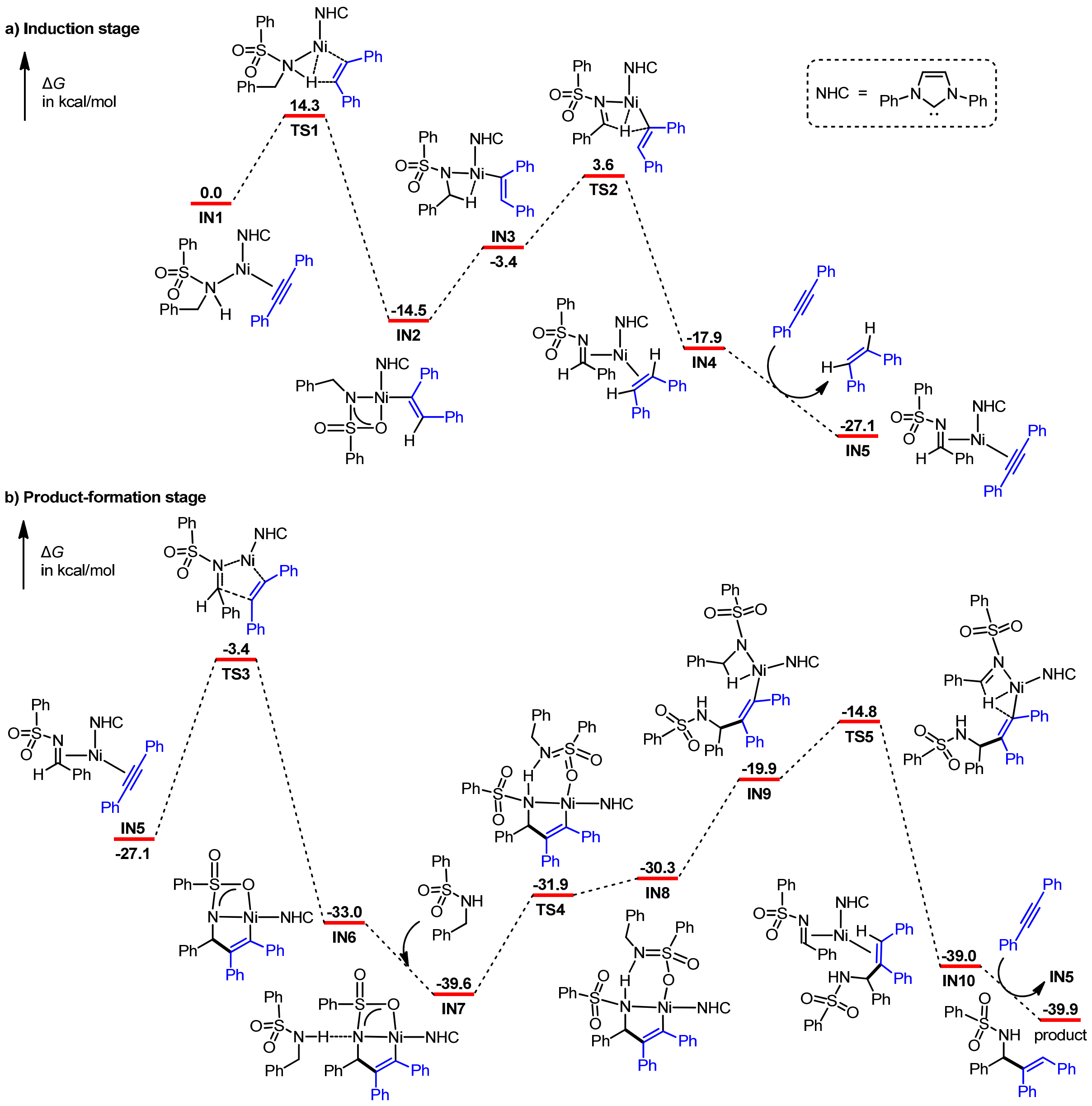

Supplementary Figure 6. Gibbs energy profiles of (a) the induction stage and (b) the product-formation stage. Computed at the SMD(toluene)/M06-D3/SDD-6-311++G(d,p)// B3LYP-D3/LANL2DZ -6-31G(d) level.

Two reaction pathways of IN5

There are two reaction pathways of Ni−imine−alkyne complex **IN5** (Figure S2). One is the oxidative cyclometallation pathway via **TS3** with an activation Gibbs energy of 23.7 kcal/mol. In the other pathway, **IN5** may first undergo a ligand exchange with the amine substrate. Then, the resulting Ni−amine−alkyne complex **IN1** may proceed through a ligand-to-ligand hydrogen transfer (LLHT) via **TS1** with an overall activation Gibbs energy of 31.7 kcal/mol.

Therefore, the oxidative cyclometallation pathway is favored over the ligand exchange/LLHT pathway by 8.0 kcal/mol. As a result, instead of triggering the transfer hydrogenation at the induction stage, **IN5** prefers to initiate a new catalytic cycle for C−C bond formation.

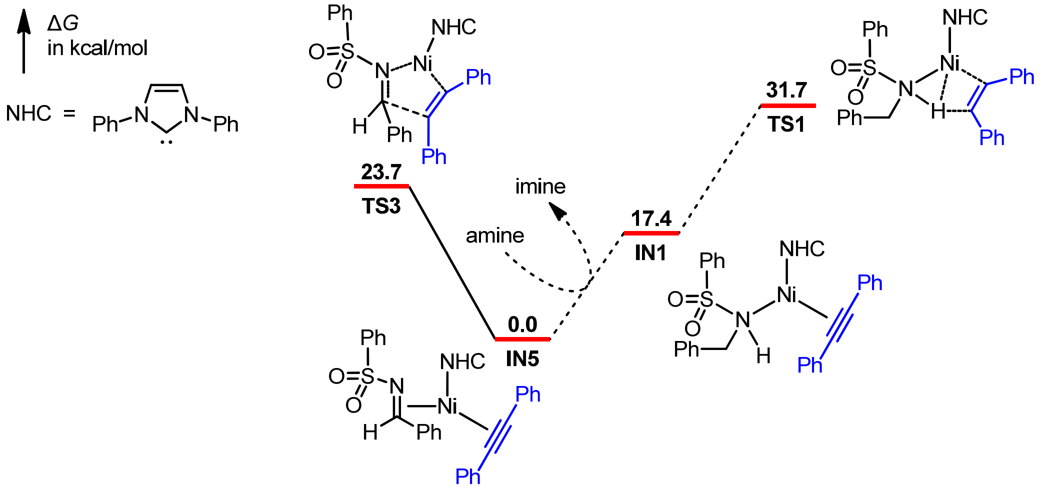

Supplementary Figure 7. Two reaction pathways of **IN5**. Computed at the SMD(toluene)/ M06-D3/SDD- 6-311++G(d,p)//B3LYP-D3/LANL2DZ-6-31G(d) level.

Explanation on ligand-to-ligand transfer pathway in TS5

For **TS5**, an alternative pathway via traditional *β*-H elimination followed by reductive elimination was also calculated. But even the Ni–H intermediate after *β*-H elimination was not located, indicating high energy of the expected stationary point and higher energy of the corresponding transition state for *β*-H elimination. In fact, although transition state for *β*-H elimination was not found, the un-converged structure already has higher electronic energy compared with **TS5** (ΔE > 10 kcal/mol). As a result, the LLHT pathway was proposed more favorable.

Role of PCy_3

To verify whether the addition of PCy_3 could reduce the overall activation Gibbs energy of the [Ni(NHC)]-catalyzed reaction, we replaced the NHC ligand of the turnover-limiting transition state **TS5** by PCy_3.

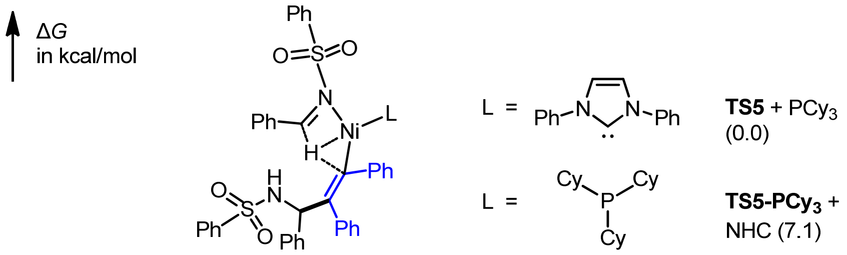

Supplementary Figure 8. Relative Gibbs energies of **TS5** and **TS5-PCy3**. Computed at the SMD(toluene)/ M06-D3/SDD-6-311++G(d,p)//B3LYP-D3/LANL2DZ-6-31G(d) level.

DFT calculations indicated that the resulting transition state **TS5-PCy3** is disfavored over **TS5** by 7.1 kcal/mol. Therefore, PCy_3 could not promote the reaction through accelerating the rate-determining step. Though the actual role of PCy_3 is unclear at the current stage, we

proposed that it might act as an auxiliary ligand to facilitate the generation of the catalytic species and/or to inhibit catalyst deactivation.

Supplementary Figures

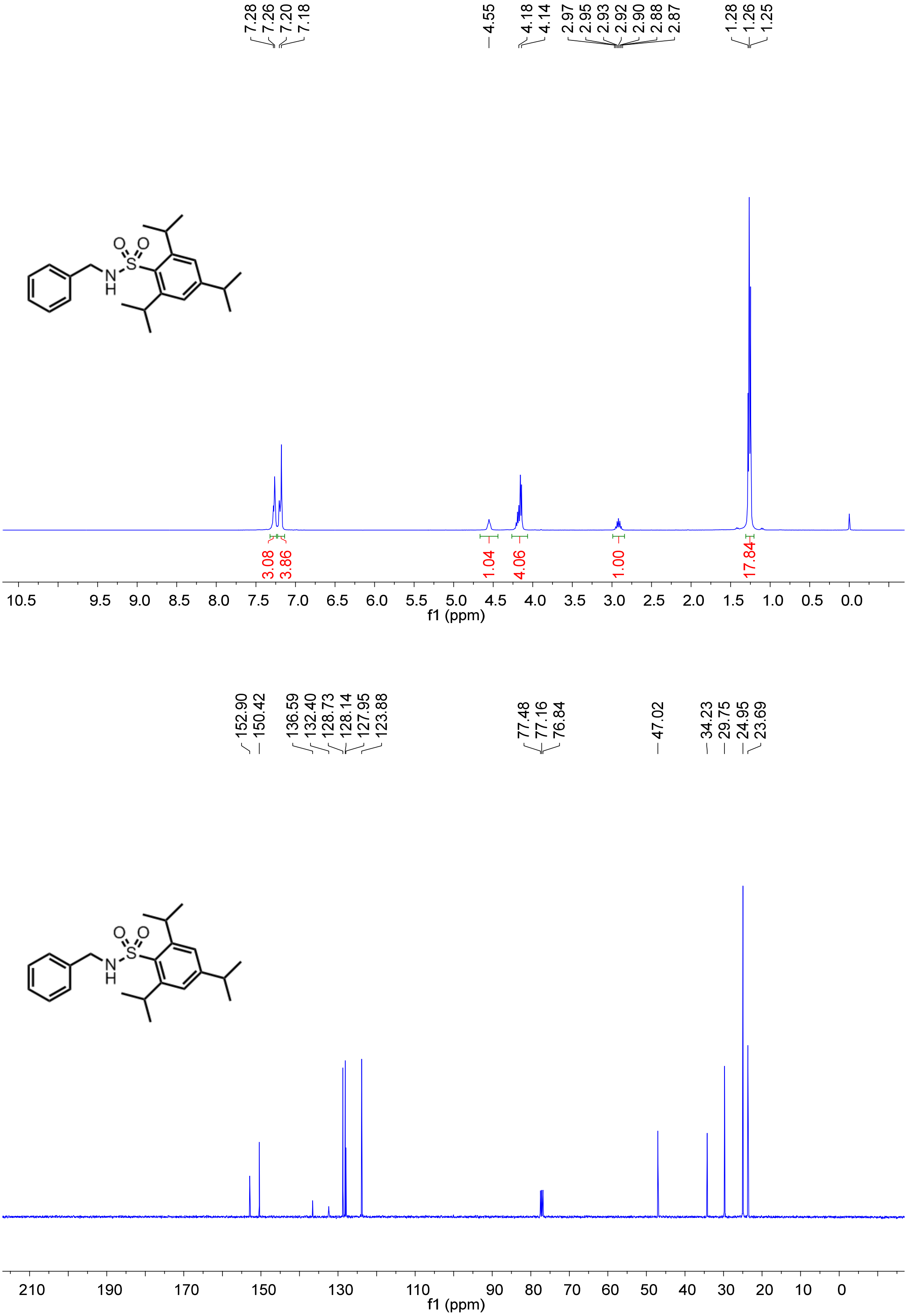

Supplementary Figure 9. ^{1}H and ^{13}C NMR spectra of compound **1a** in $CDCl_3$.

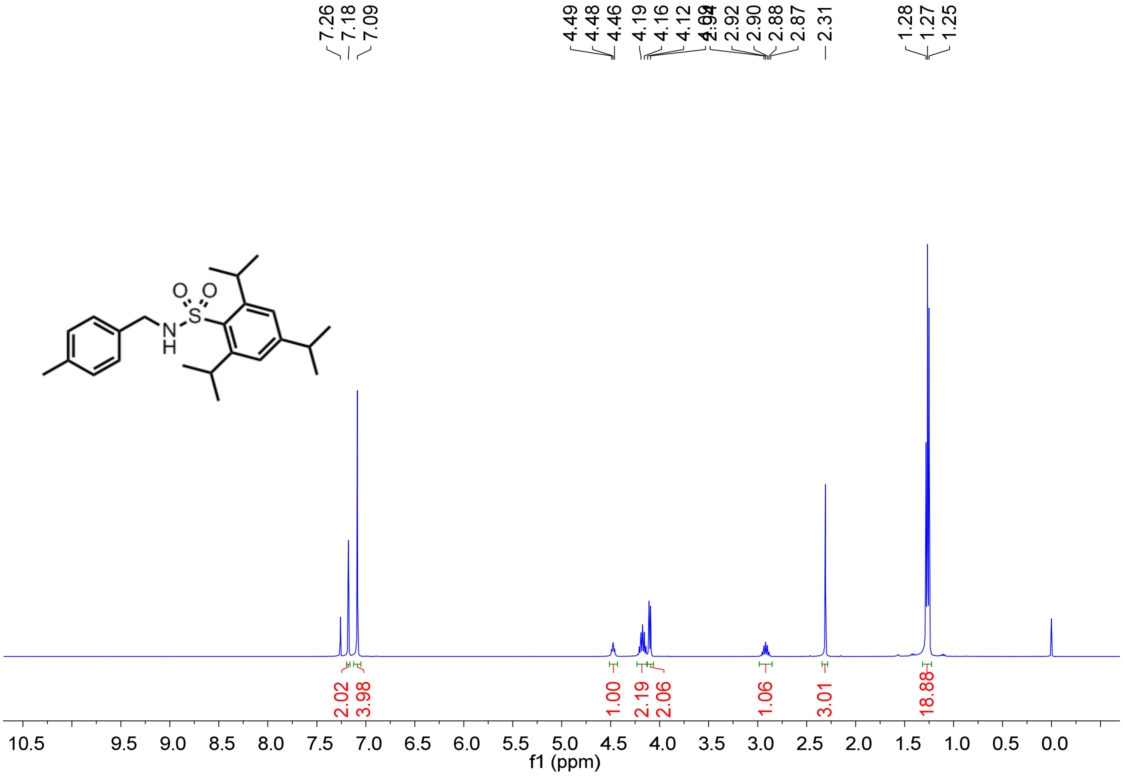

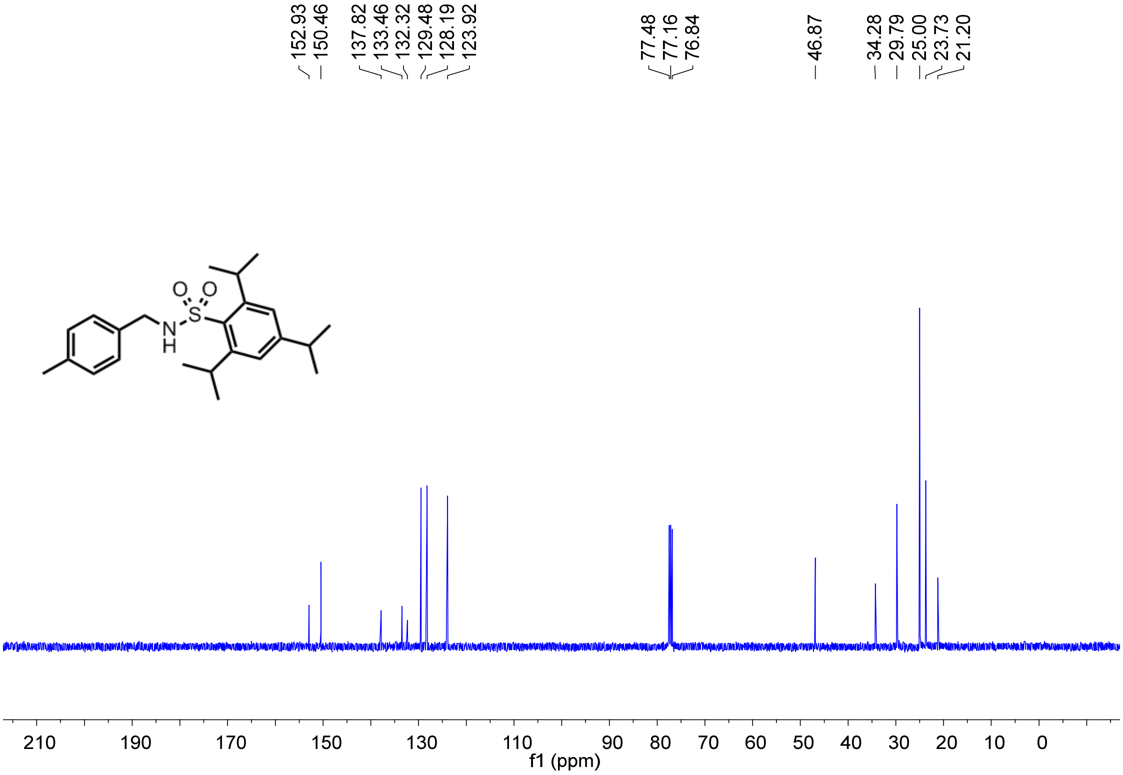

Supplementary Figure 10. ^{1}H and ^{13}C NMR spectra of compound **1b** in $CDCl_3$.

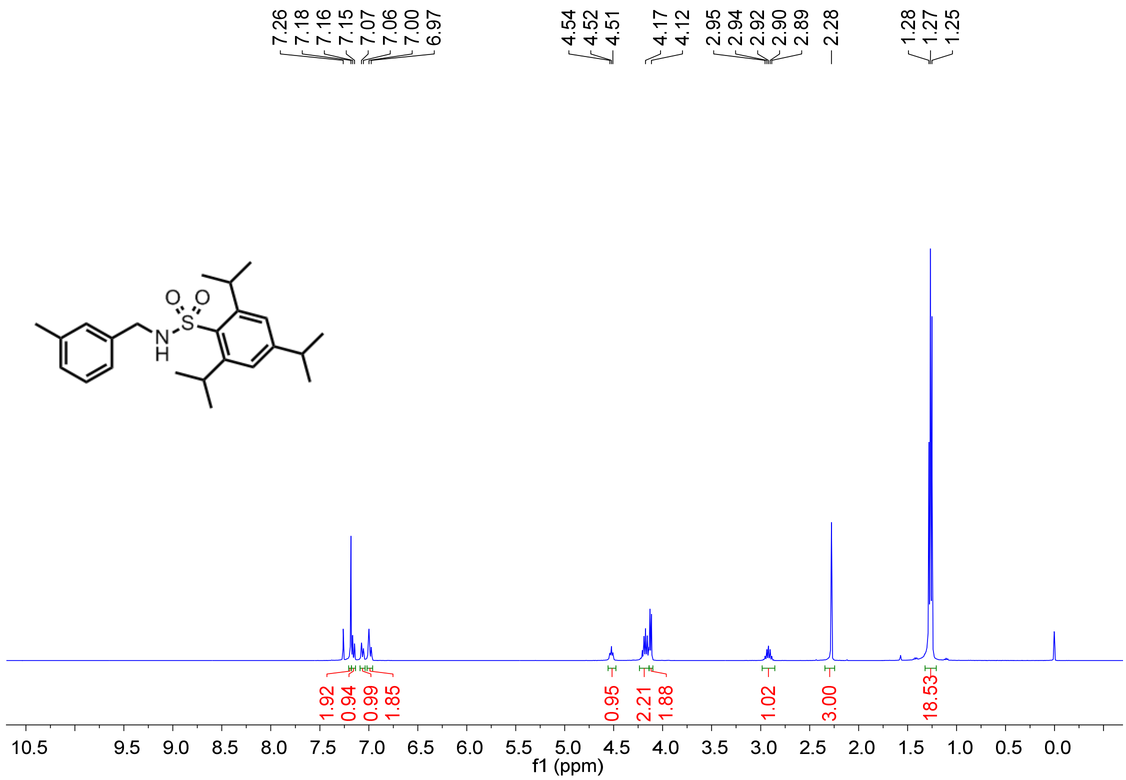

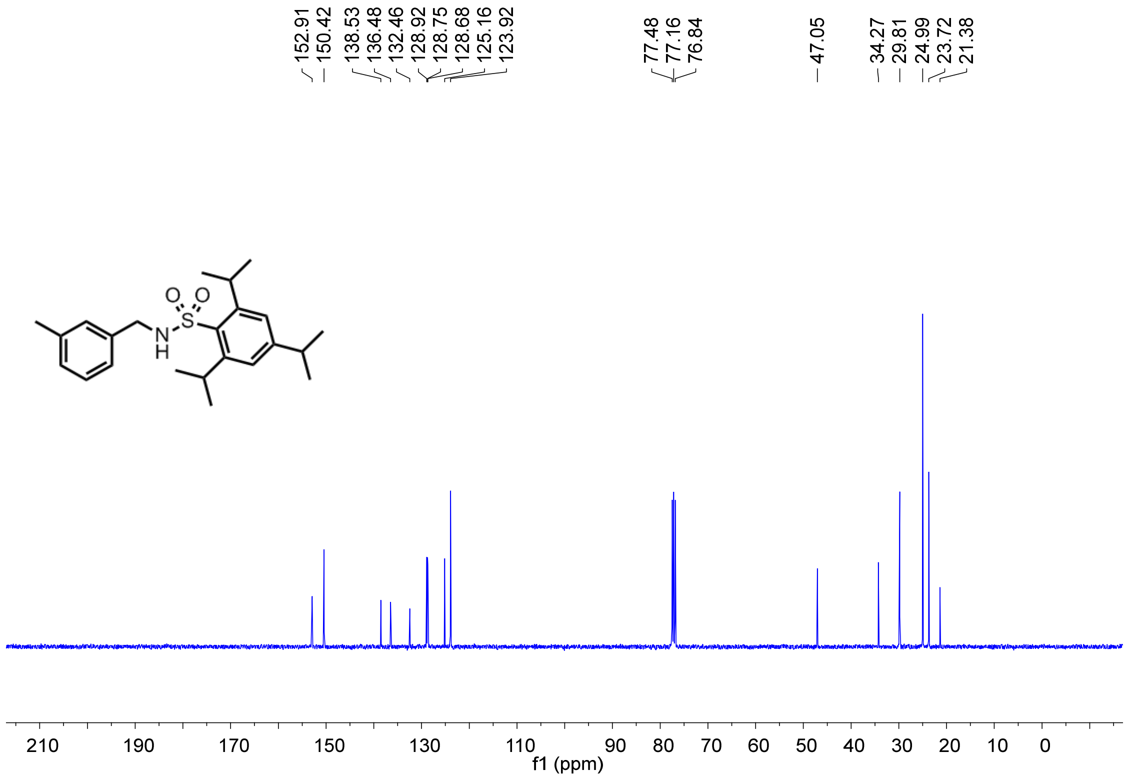

Supplementary Figure 11. ^{1}H and ^{13}C NMR spectra of compound **1c** in $CDCl_3$.

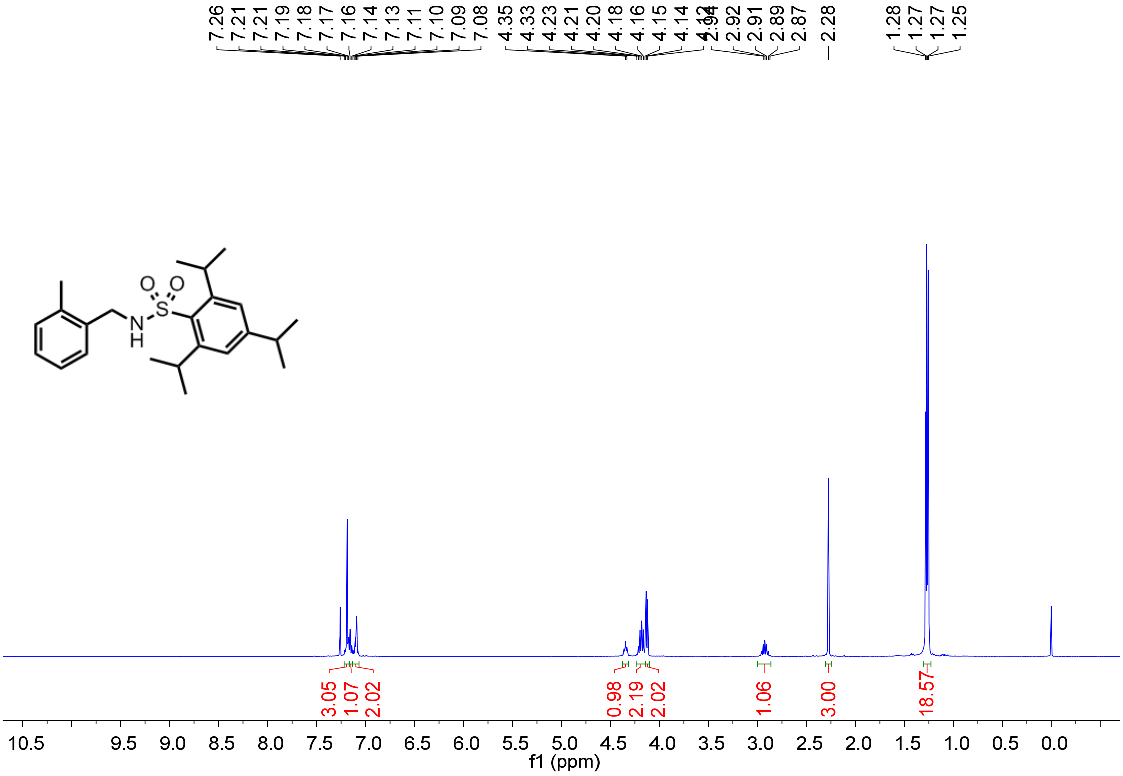

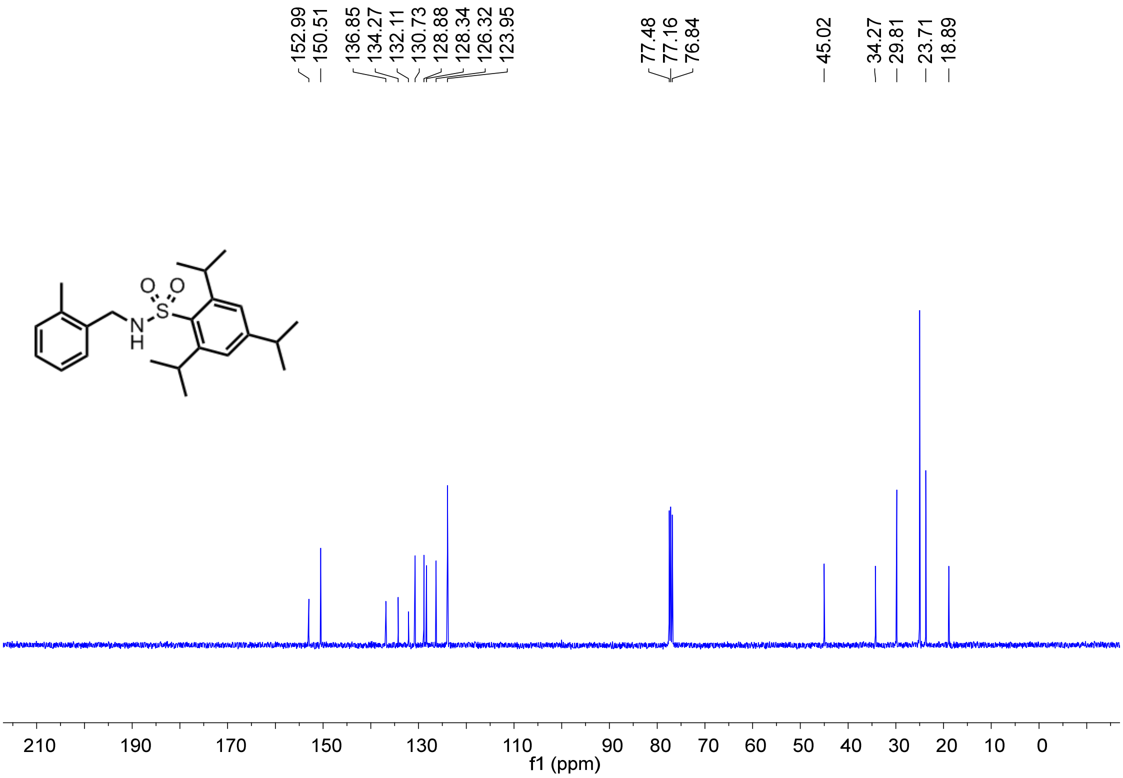

Supplementary Figure 12. ^{1}H and ^{13}C NMR spectra of compound **1d** in $CDCl_3$.

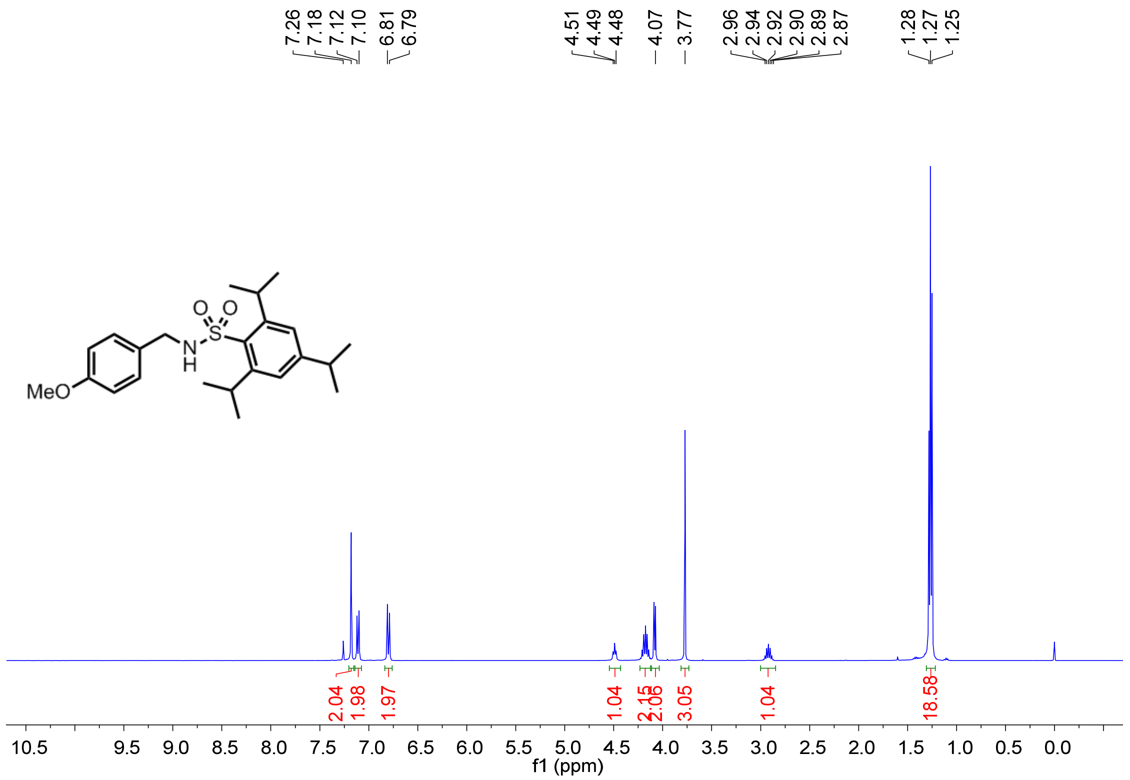

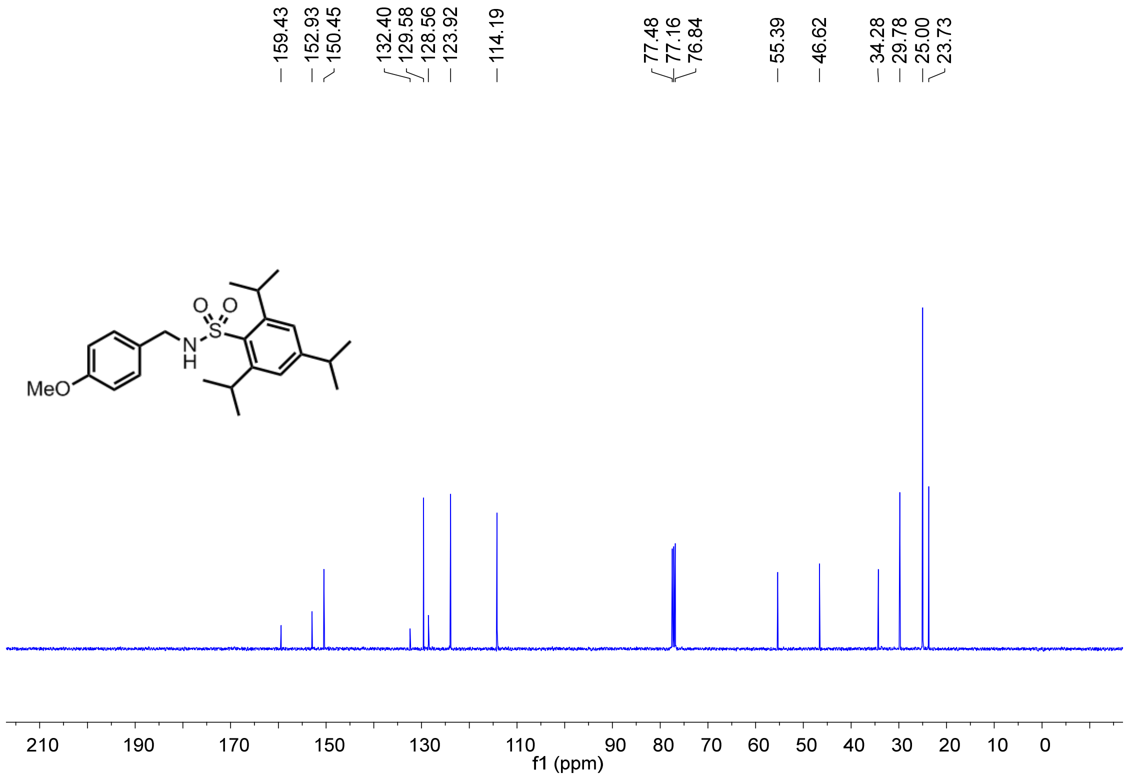

Supplementary Figure 13. ^{1}H and ^{13}C NMR spectra of compound **1e** in $CDCl_3$.

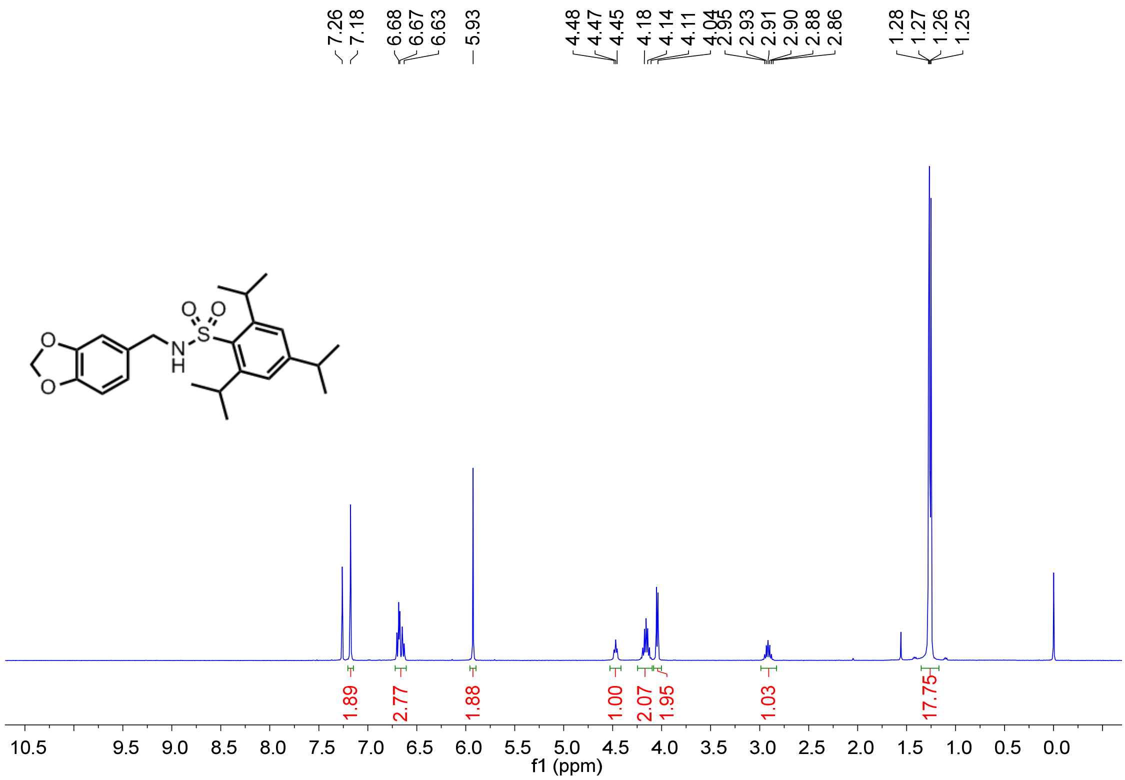

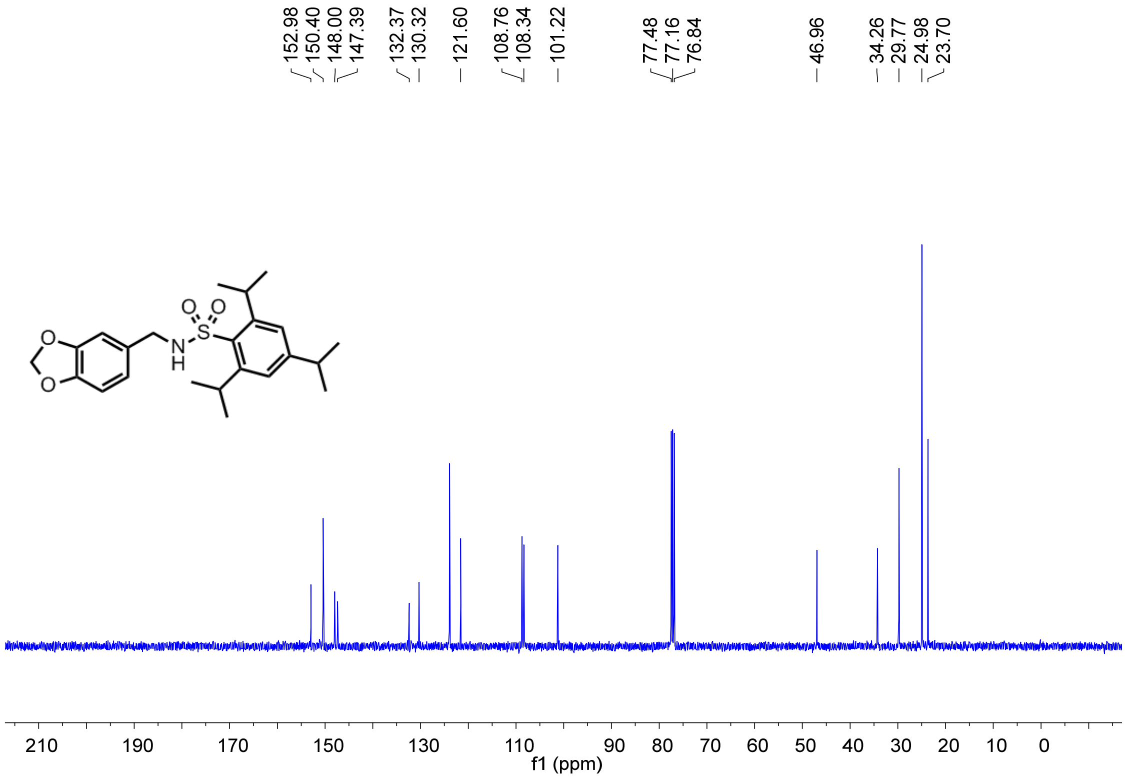

Supplementary Figure 14. ^{1}H and ^{13}C NMR spectra of compound **1f** in $CDCl_3$.

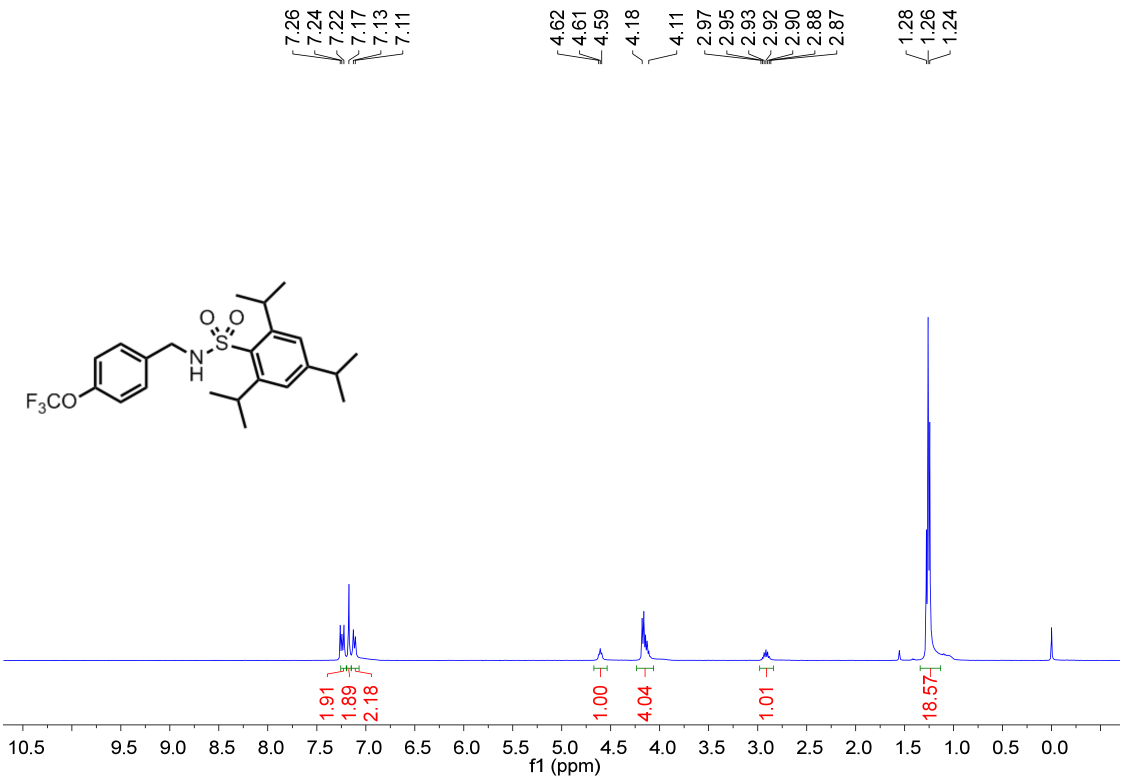

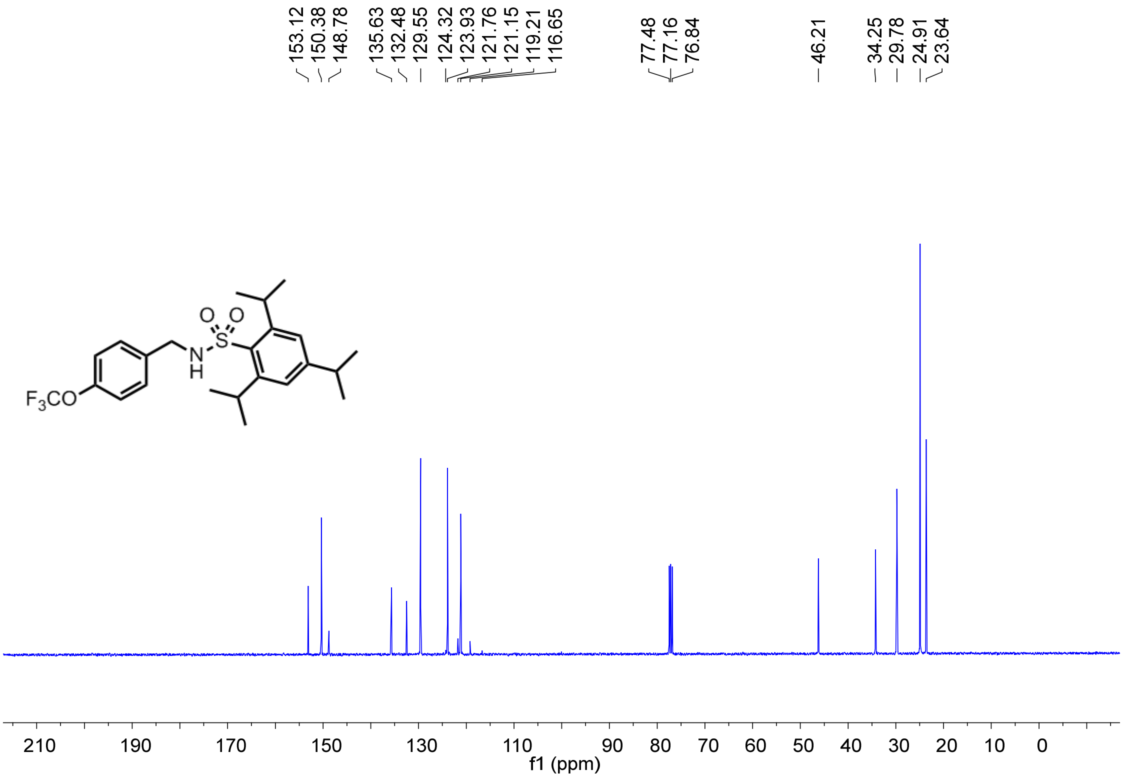

Supplementary Figure 15. ^{1}H and ^{13}C NMR spectra of compound **1g** in $CDCl_3$.

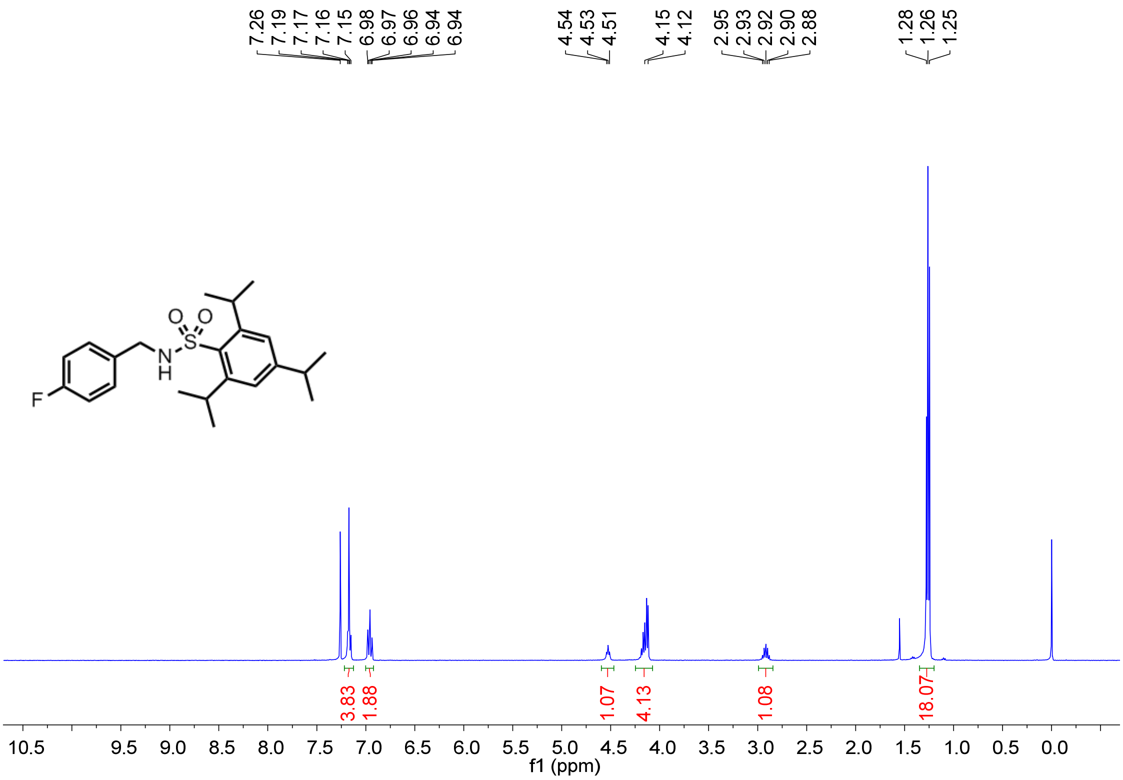

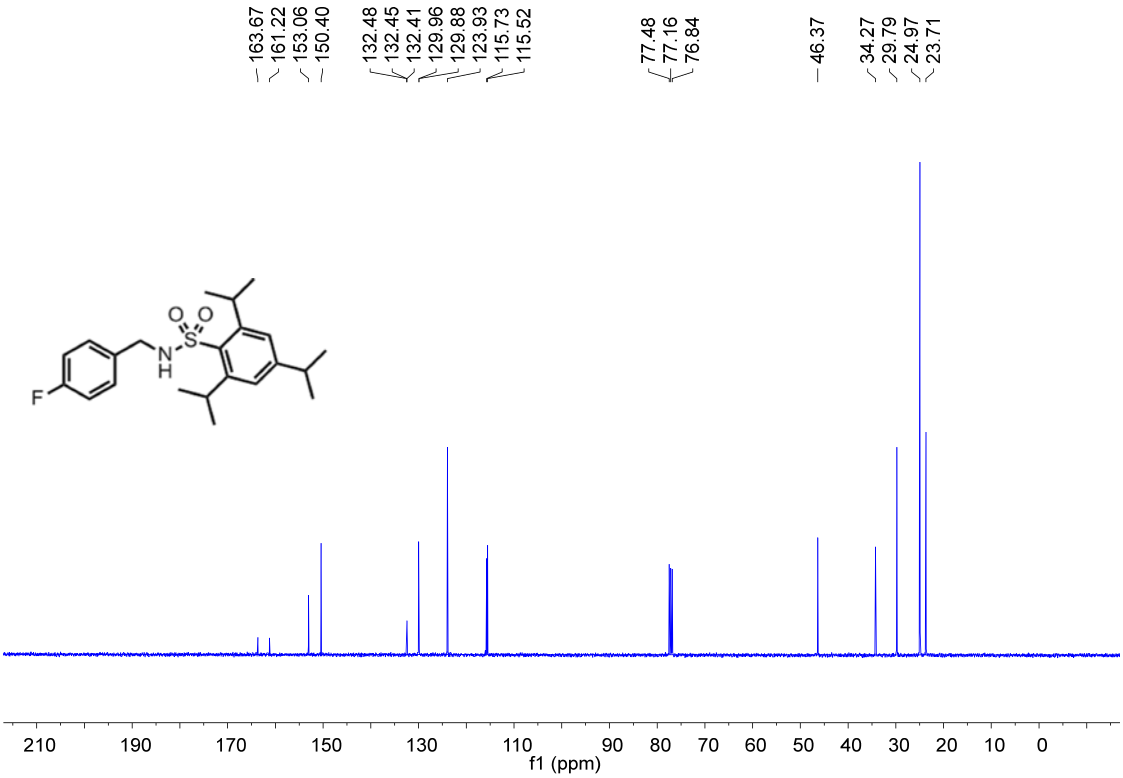

Supplementary Figure 16. ^{1}H and ^{13}C NMR spectra of compound **1h** in $CDCl_3$.

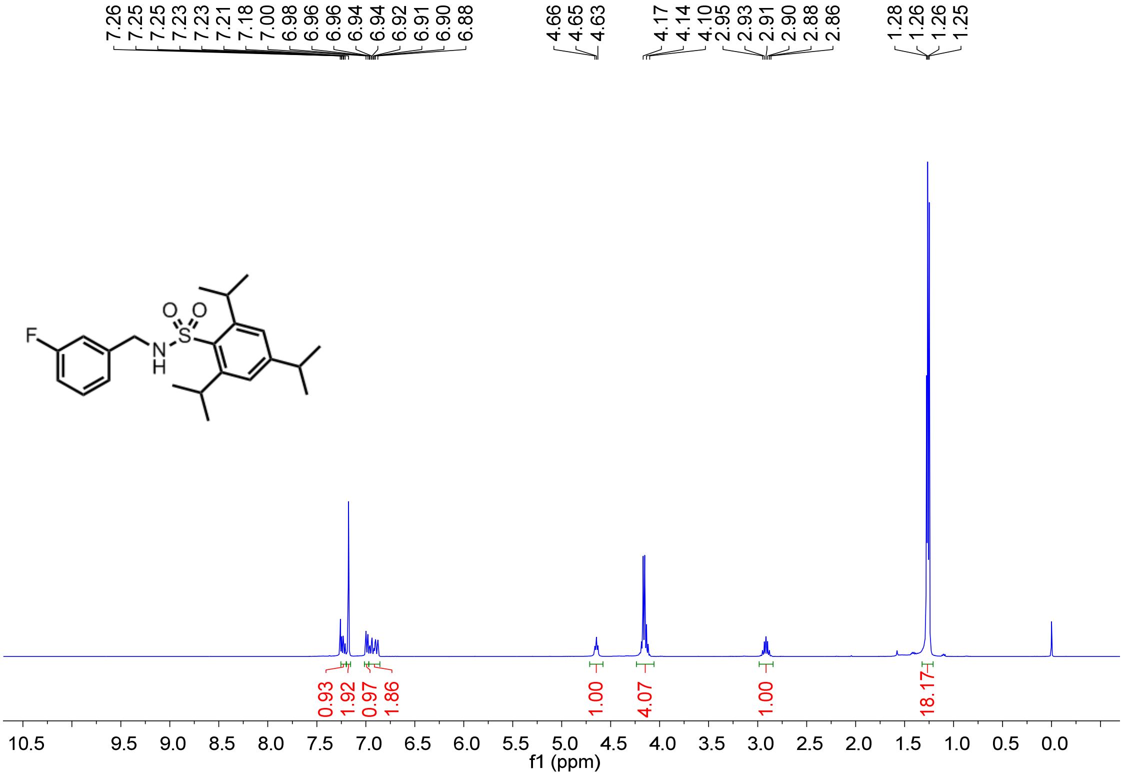

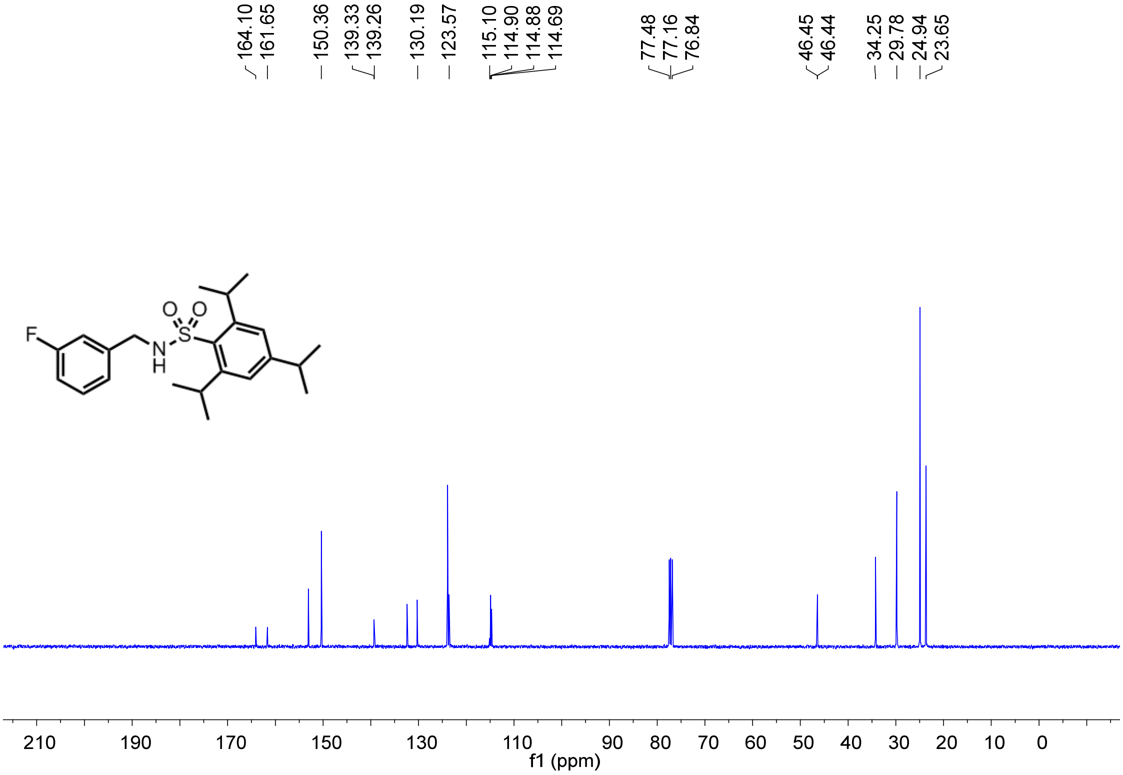

Supplementary Figure 17. ^{1}H and ^{13}C NMR spectra of compound **1i** in $CDCl_3$.

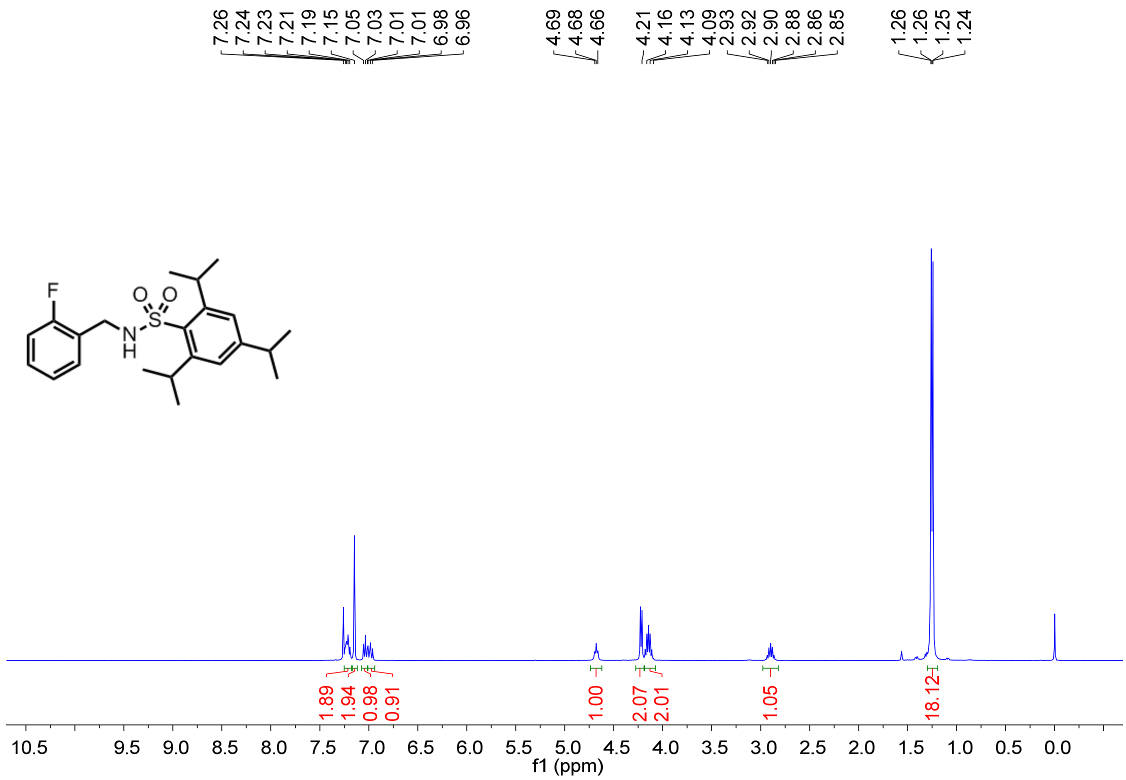

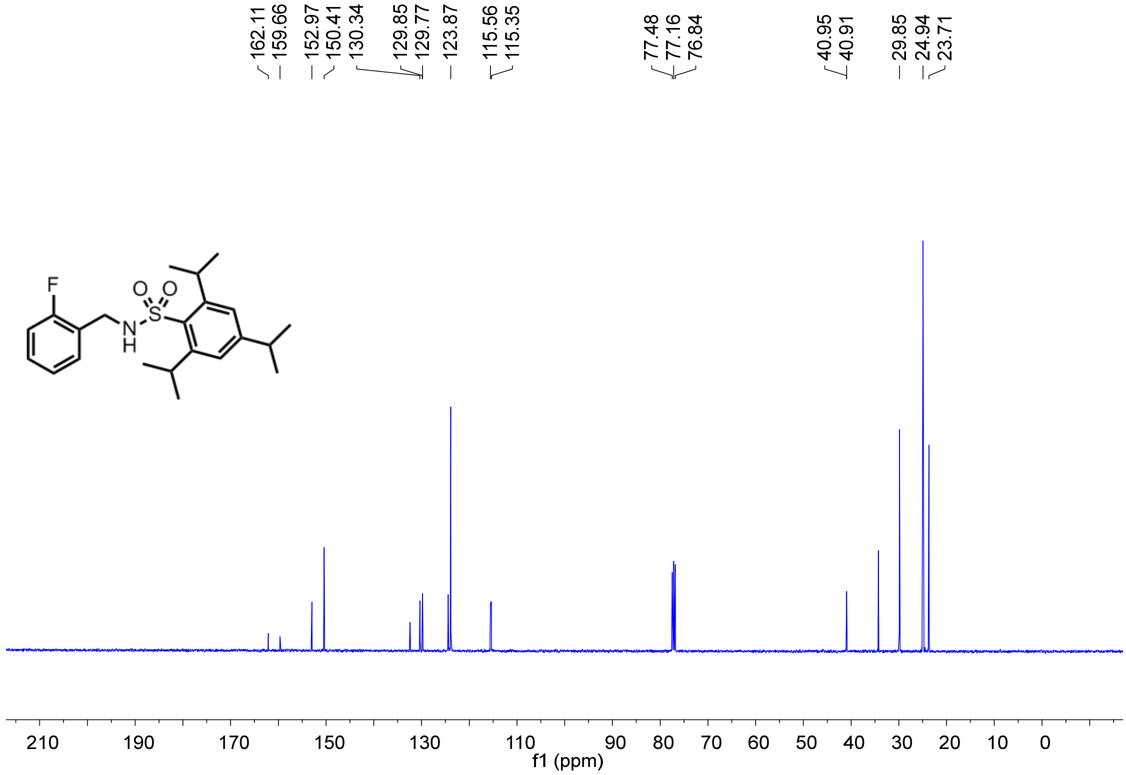

Supplementary Figure 18. ^{1}H and ^{13}C NMR spectra of compound **1j** in $CDCl_3$.

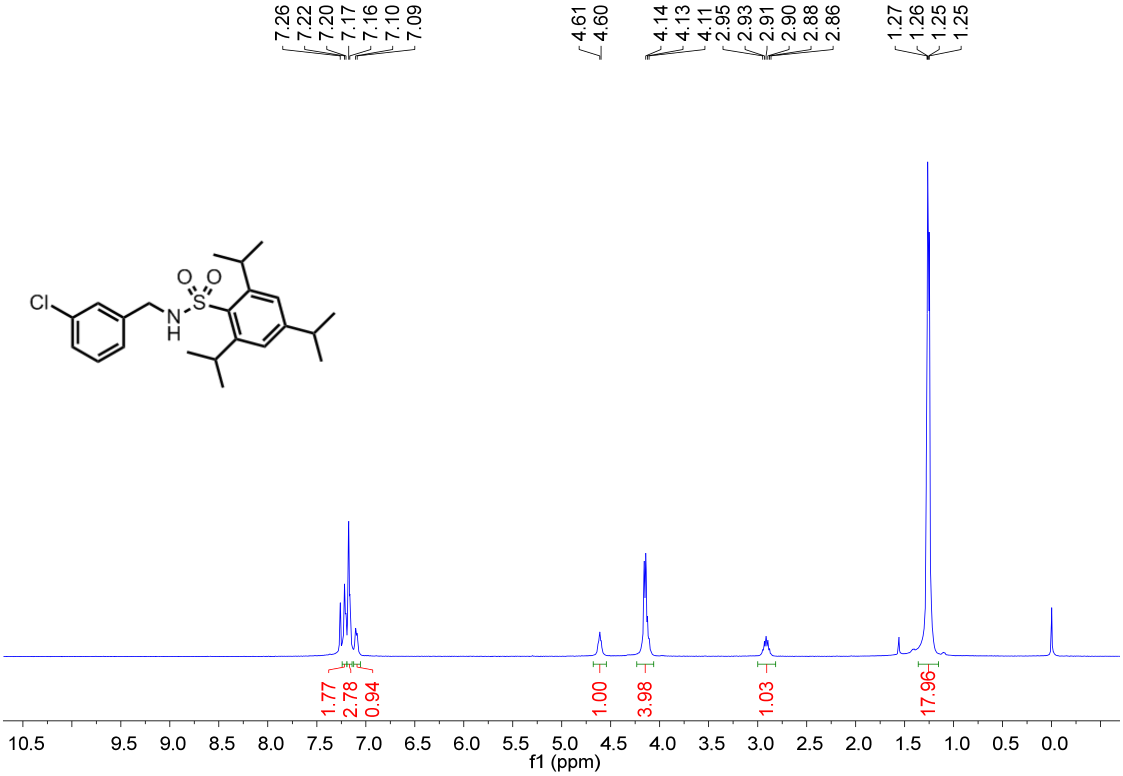

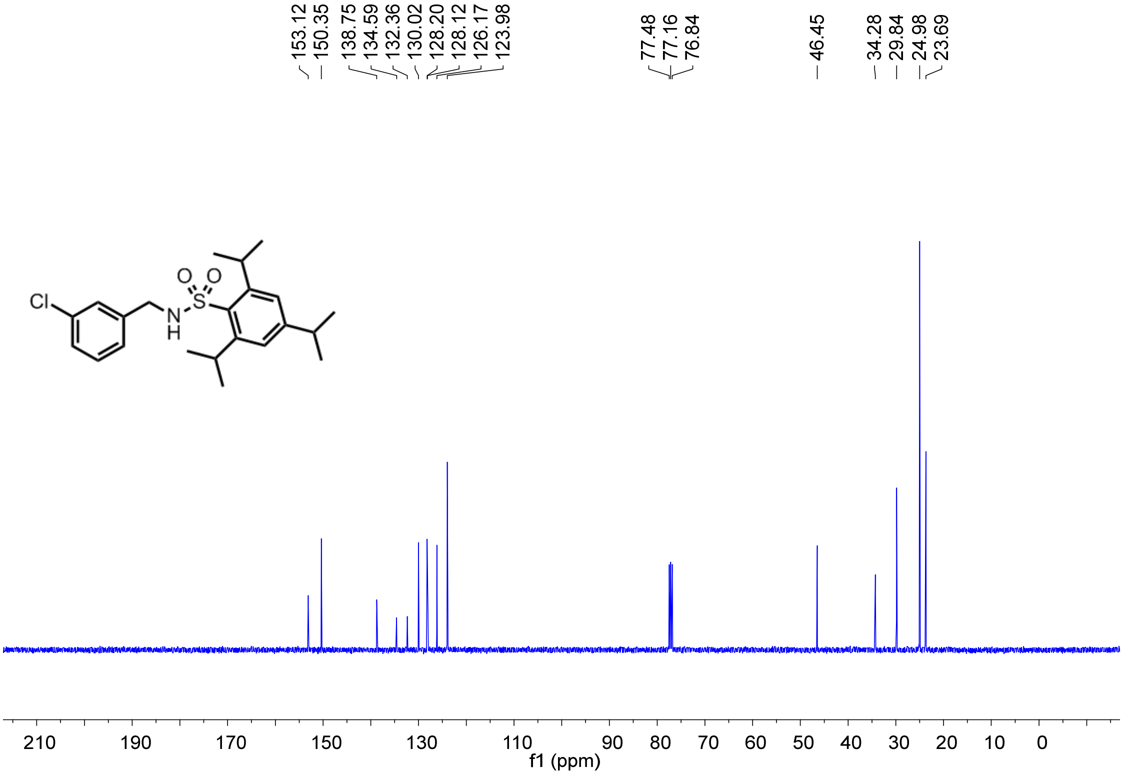

Supplementary Figure 19. ^{1}H and ^{13}C NMR spectra of compound **1k** in $CDCl_3$.

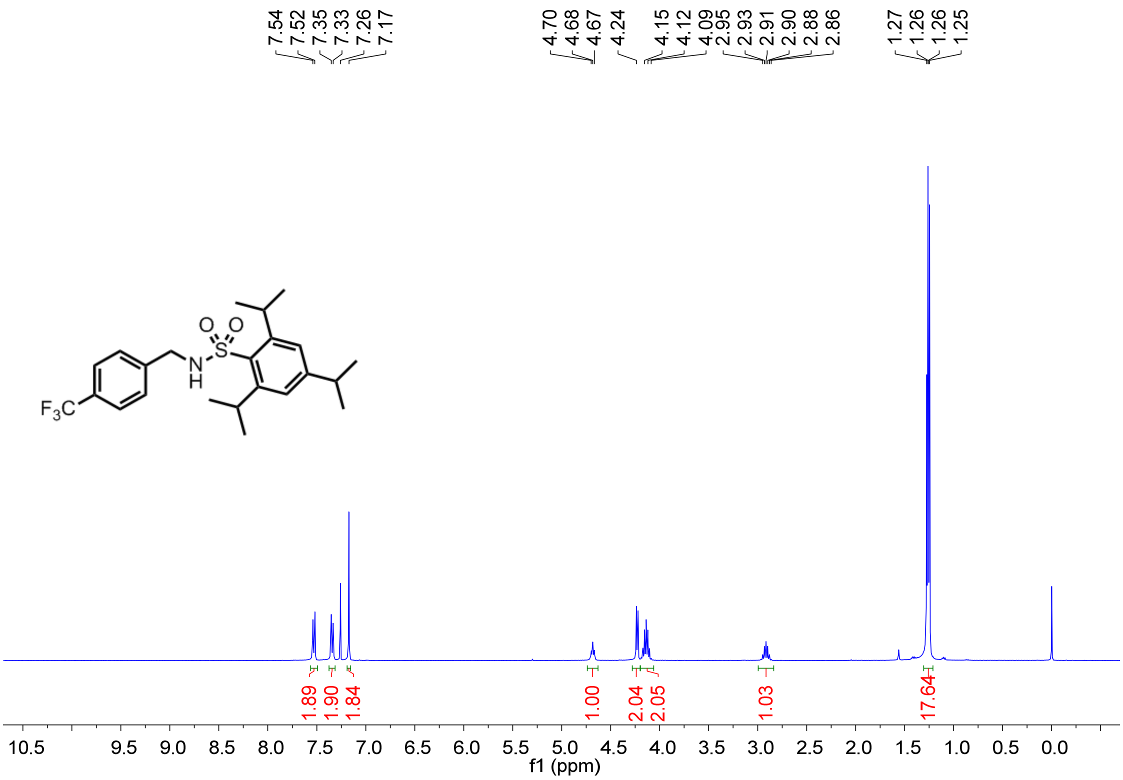

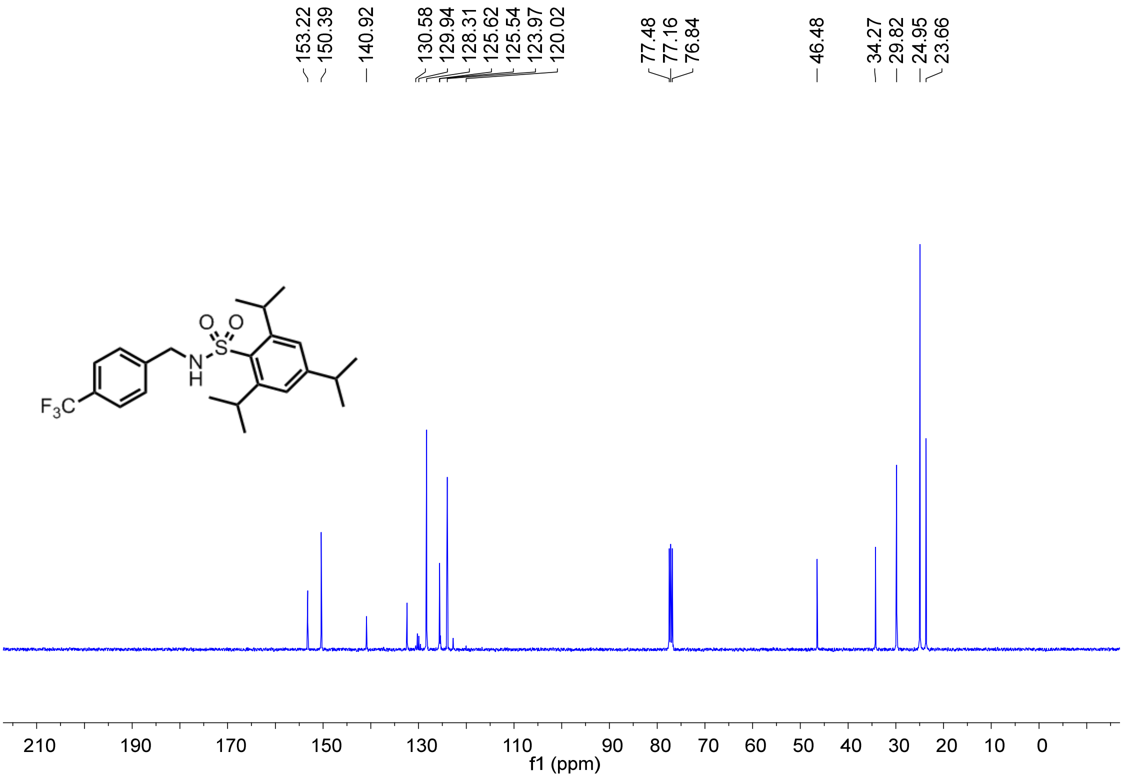

Supplementary Figure 20. ^{1}H and ^{13}C NMR spectra of compound **1l** in $CDCl_3$.

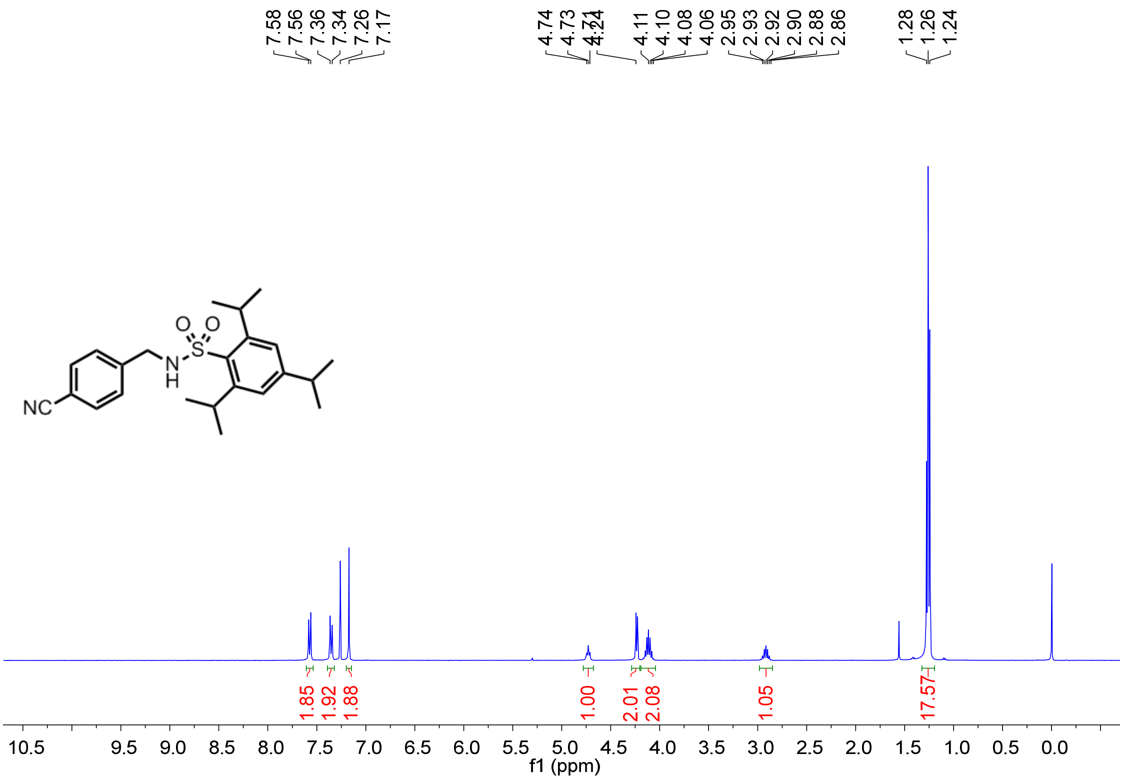

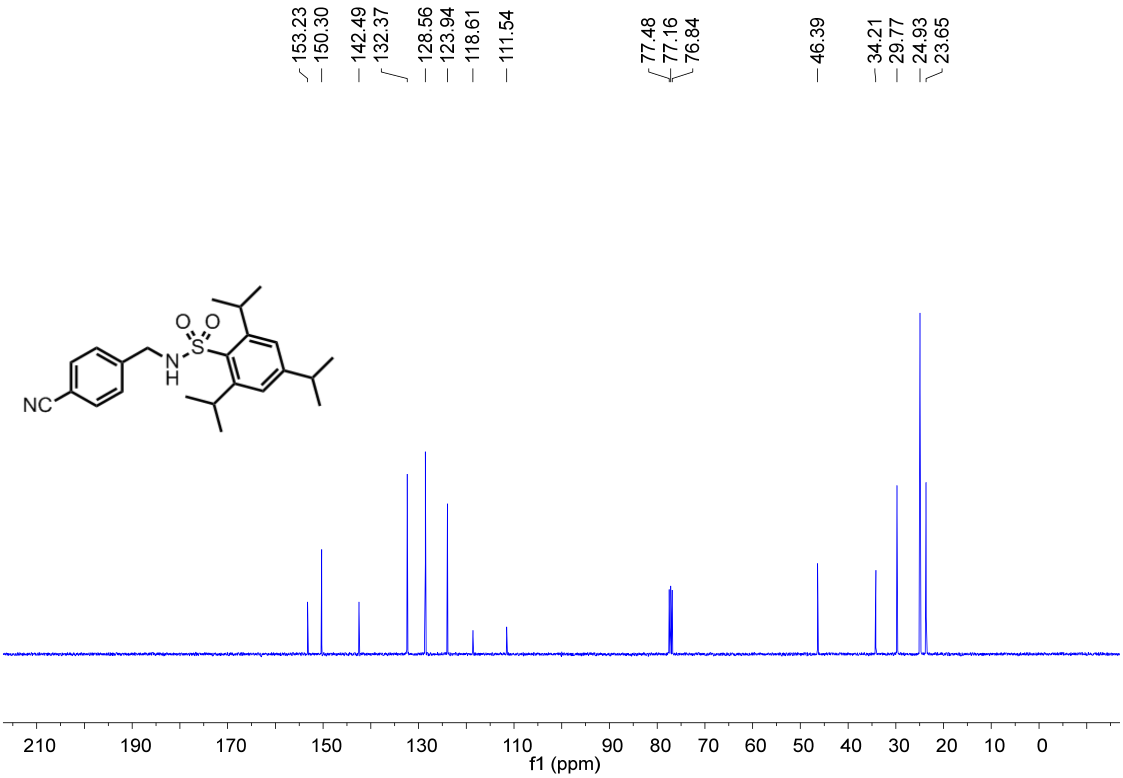

Supplementary Figure 21. ^{1}H and ^{13}C NMR spectra of compound **1m** in $CDCl_3$.

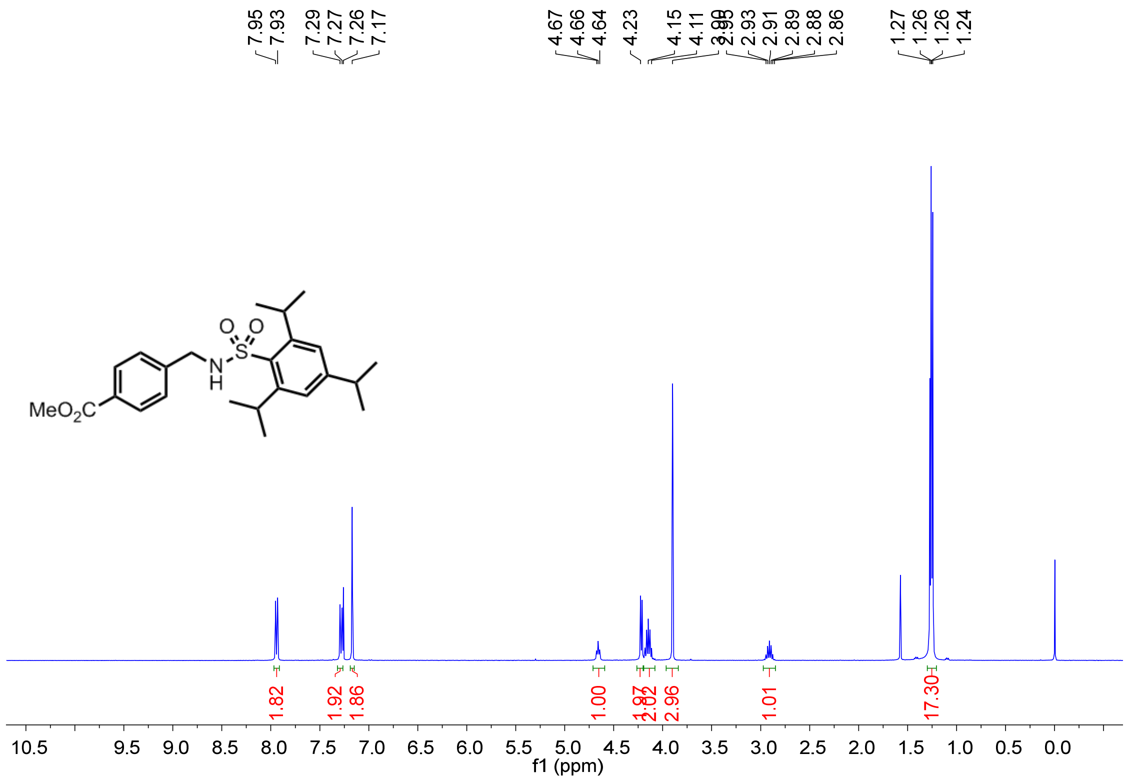

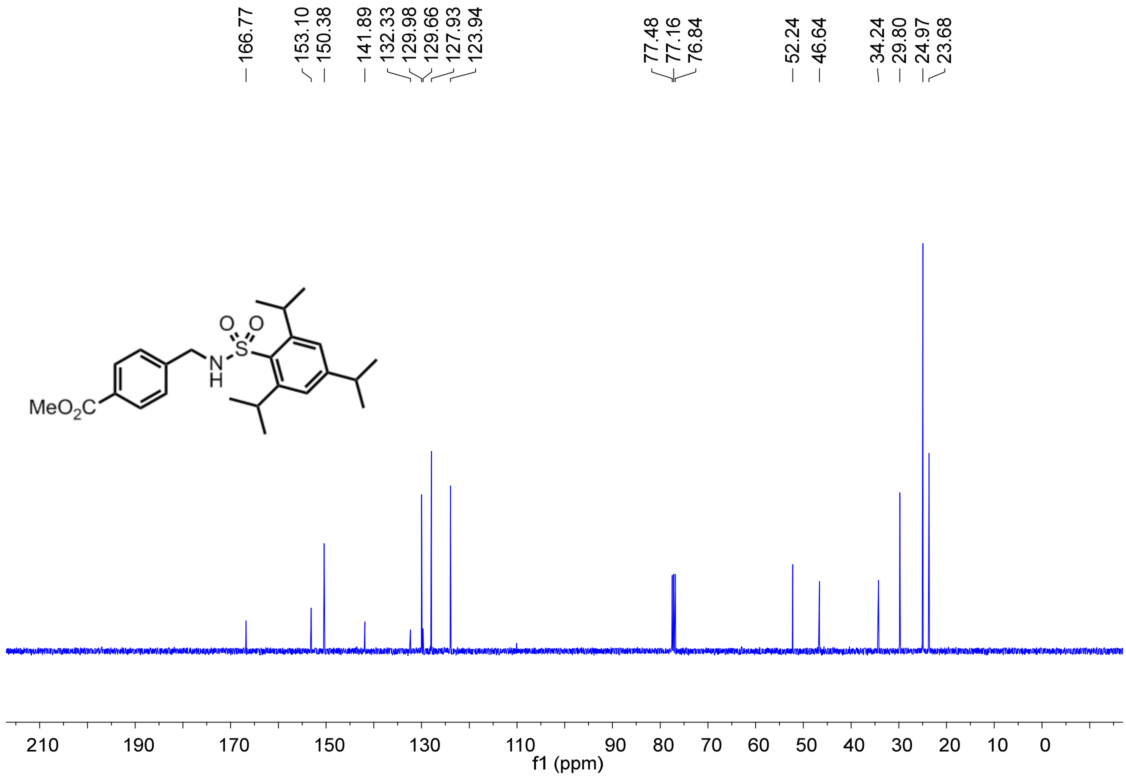

Supplementary Figure 22. ^{1}H and ^{13}C NMR spectra of compound **1n** in $CDCl_3$.

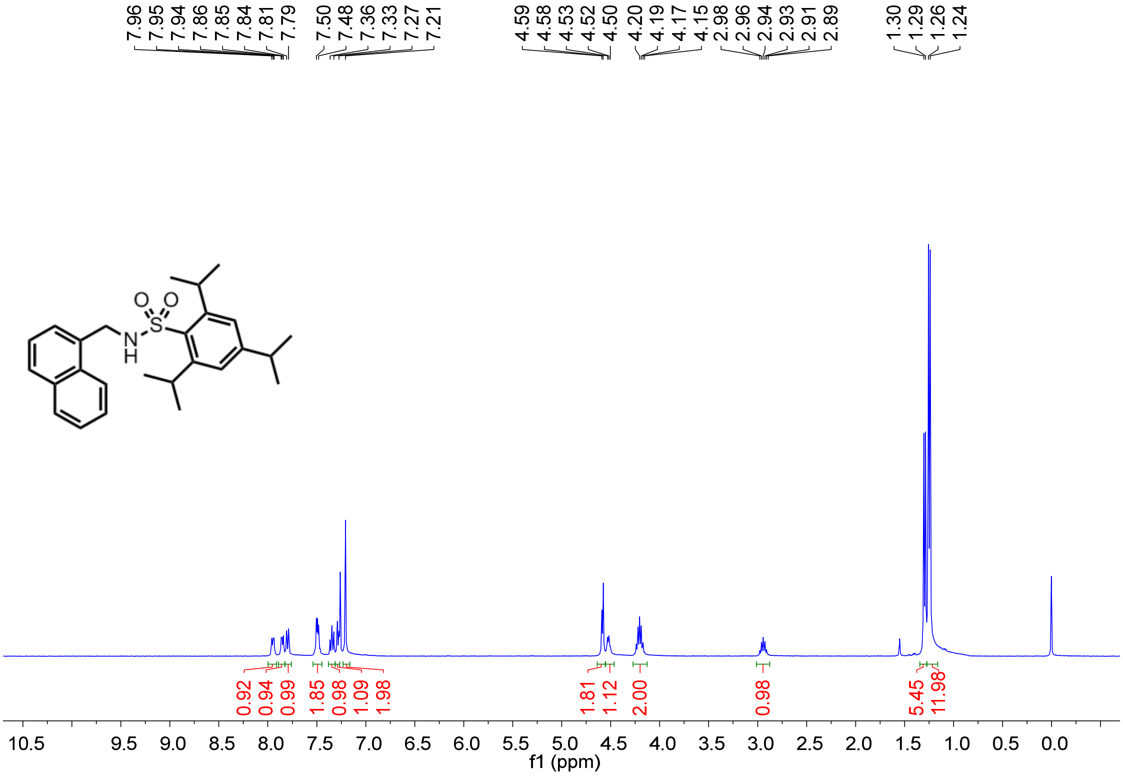

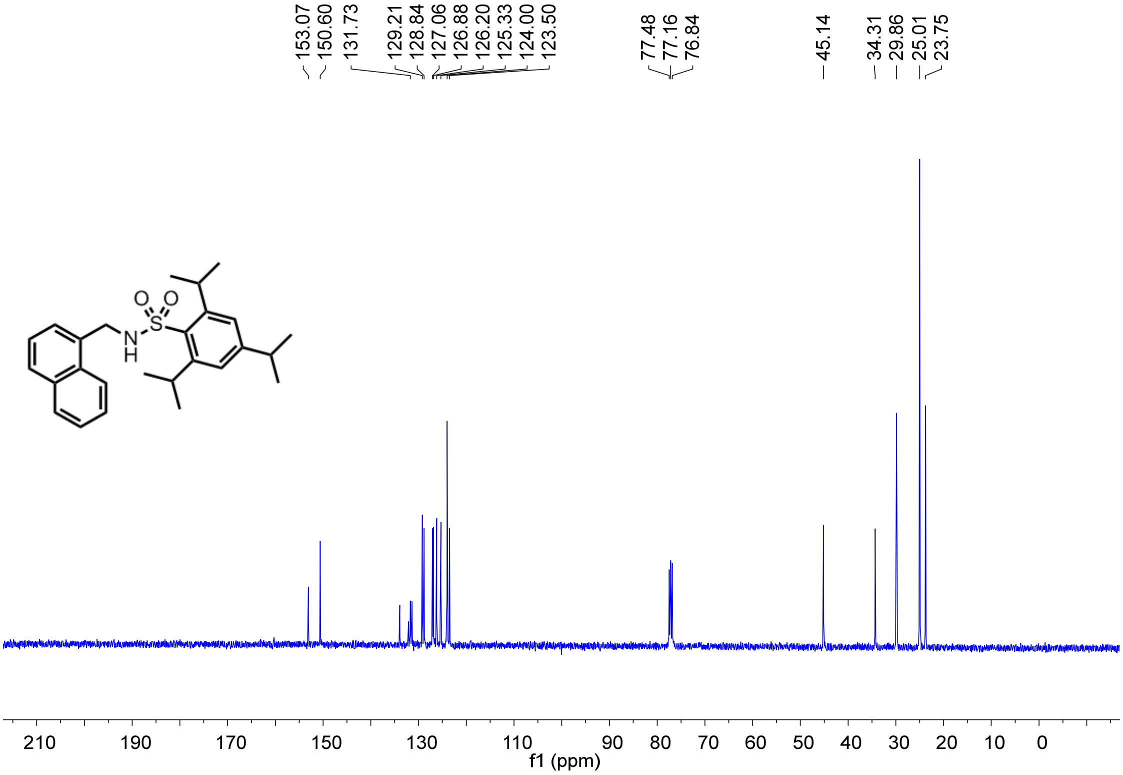

Supplementary Figure 23. ^{1}H and ^{13}C NMR spectra of compound **1o** in $CDCl_3$.

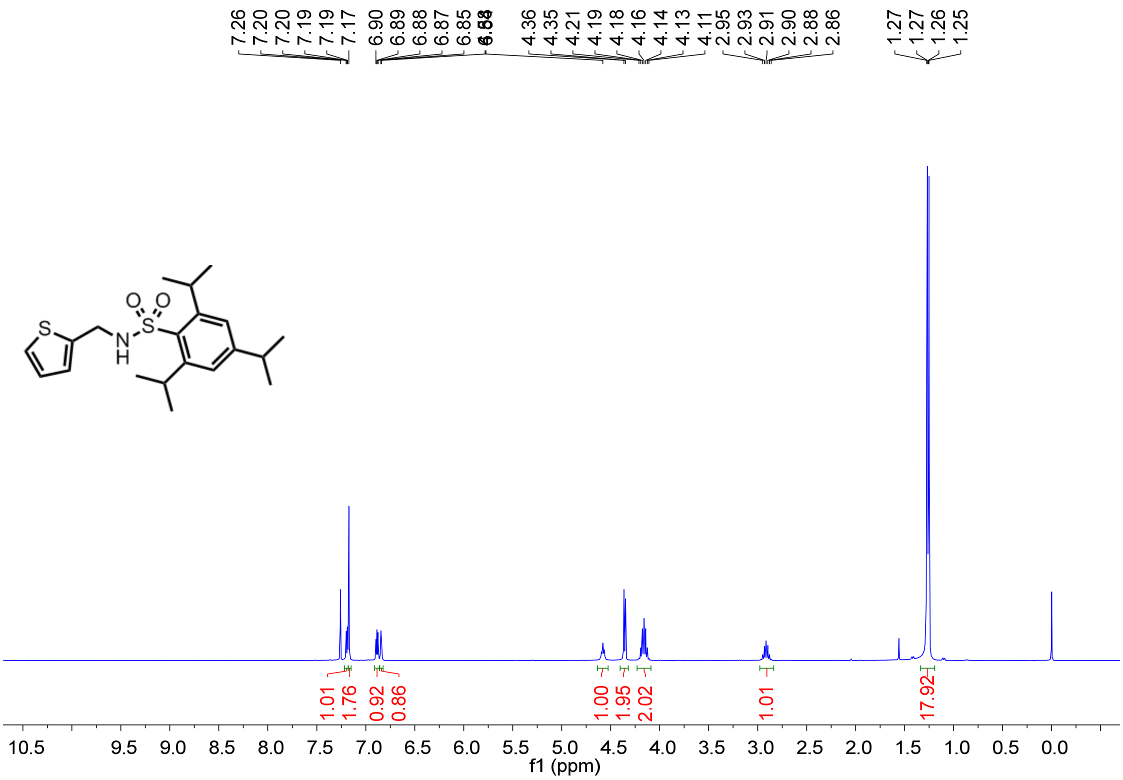

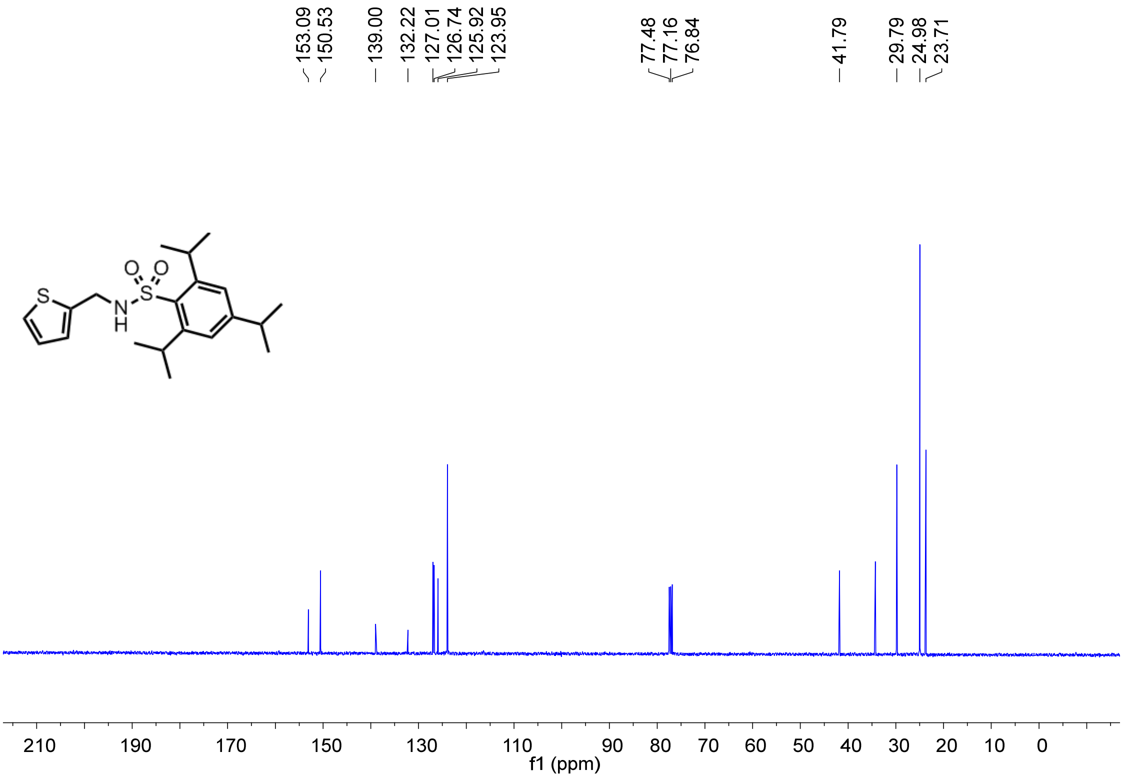

Supplementary Figure 24. ^{1}H and ^{13}C NMR spectra of compound **1p** in $CDCl_3$.

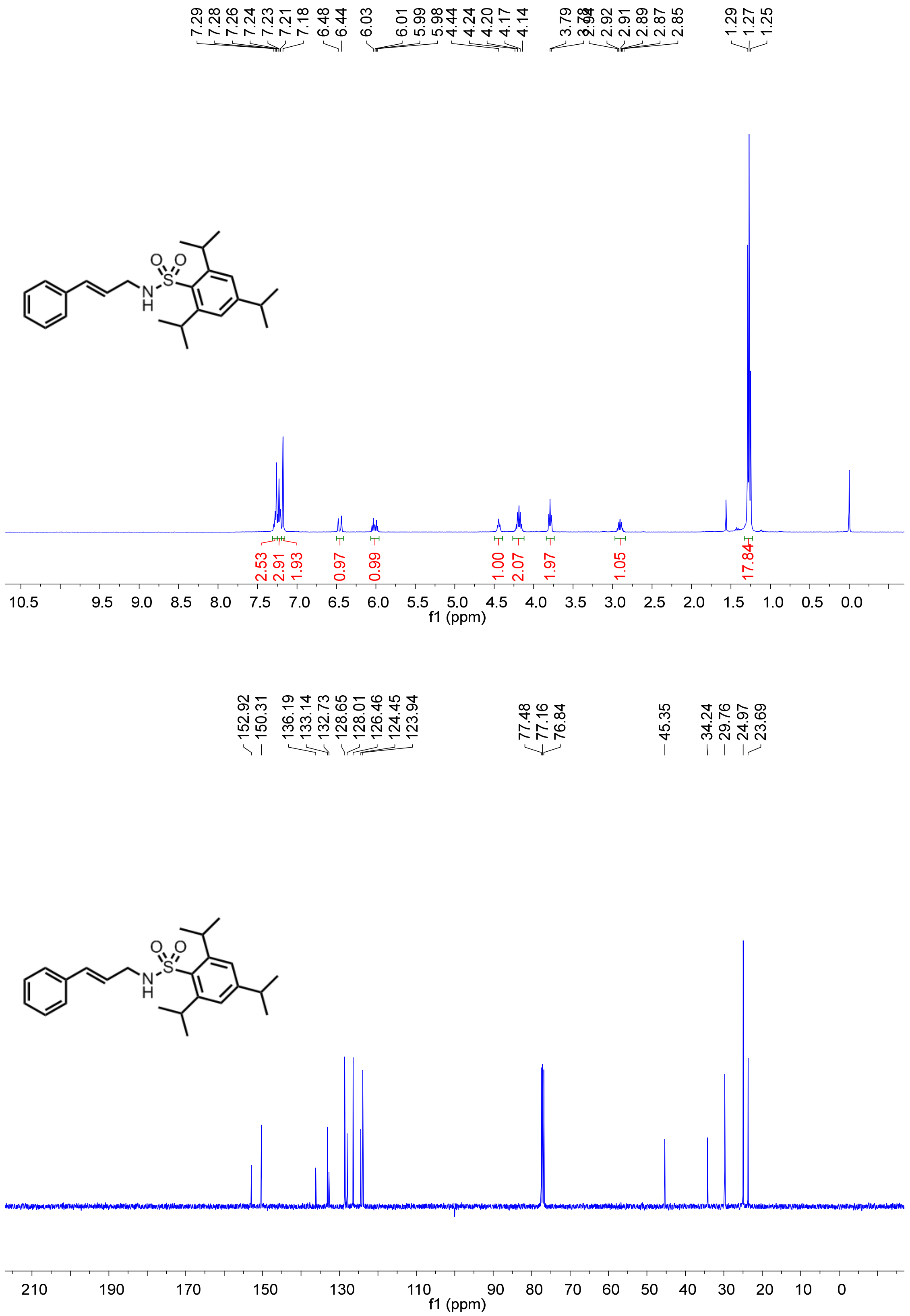

Supplementary Figure 25. ^{1}H and ^{13}C NMR spectra of compound **1q** in $CDCl_3$.

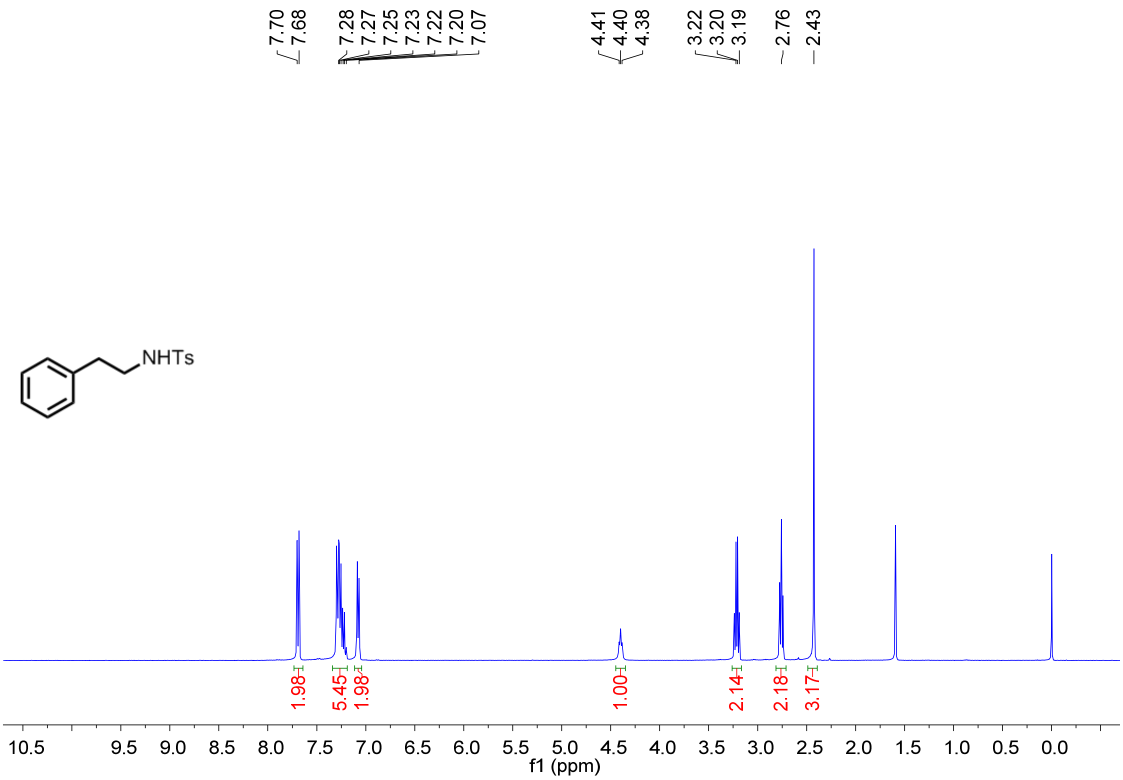

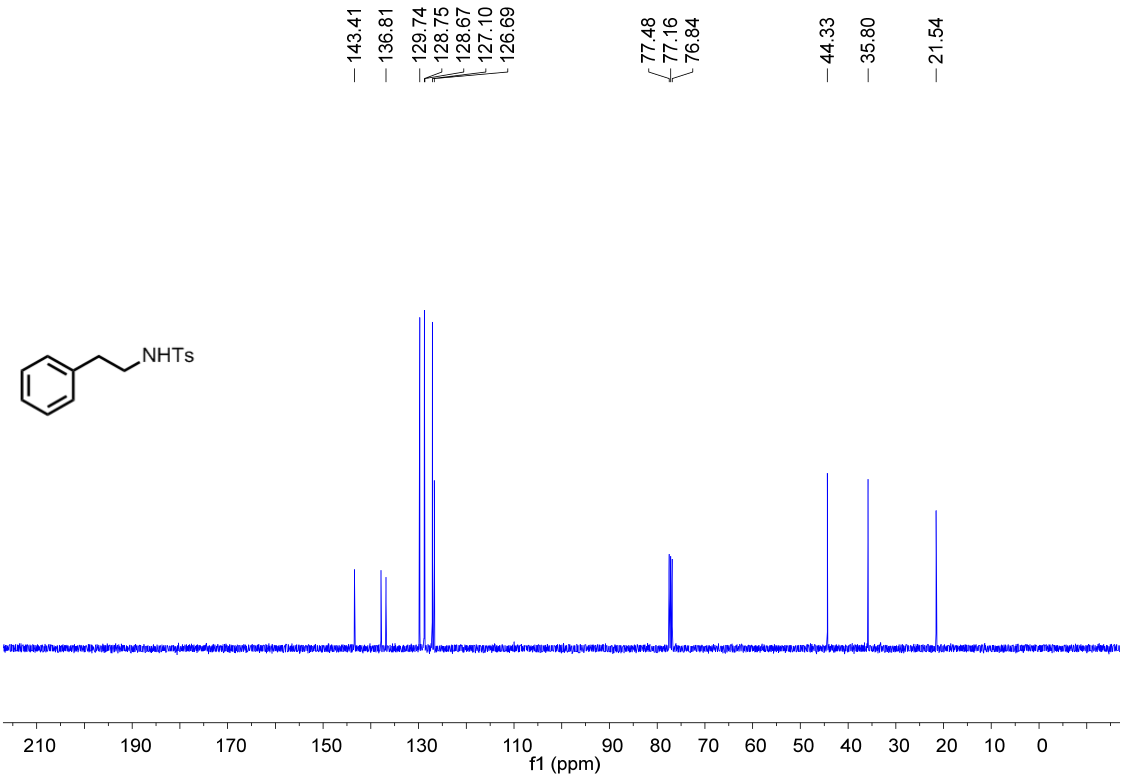

Supplementary Figure 26. ^{1}H and ^{13}C NMR spectra of compound **1r** in $CDCl_3$.

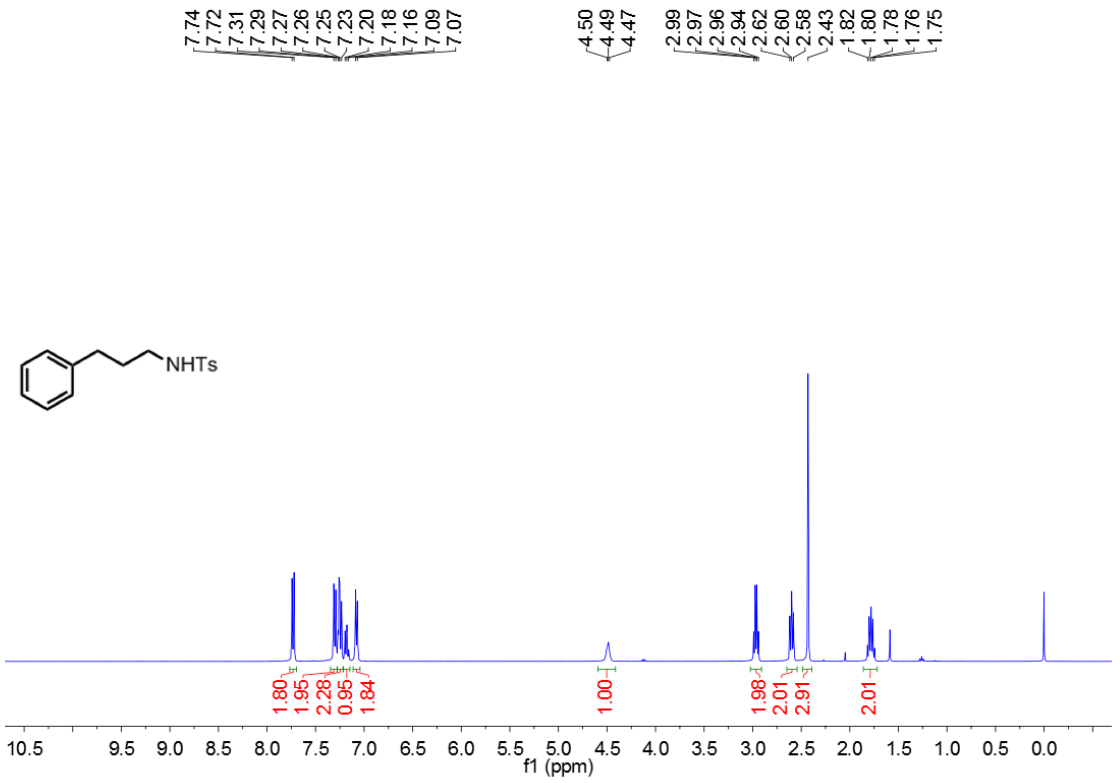

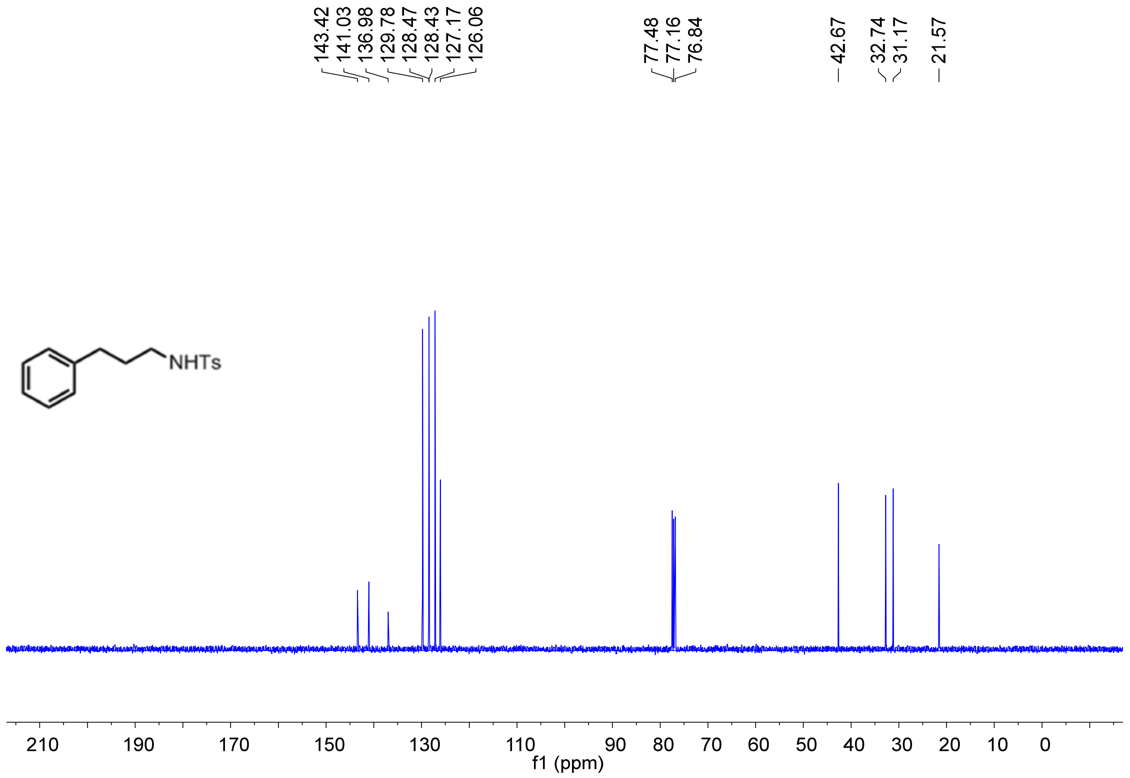

Supplementary Figure 27. ^{1}H and ^{13}C NMR spectra of compound **1s** in $CDCl_3$.

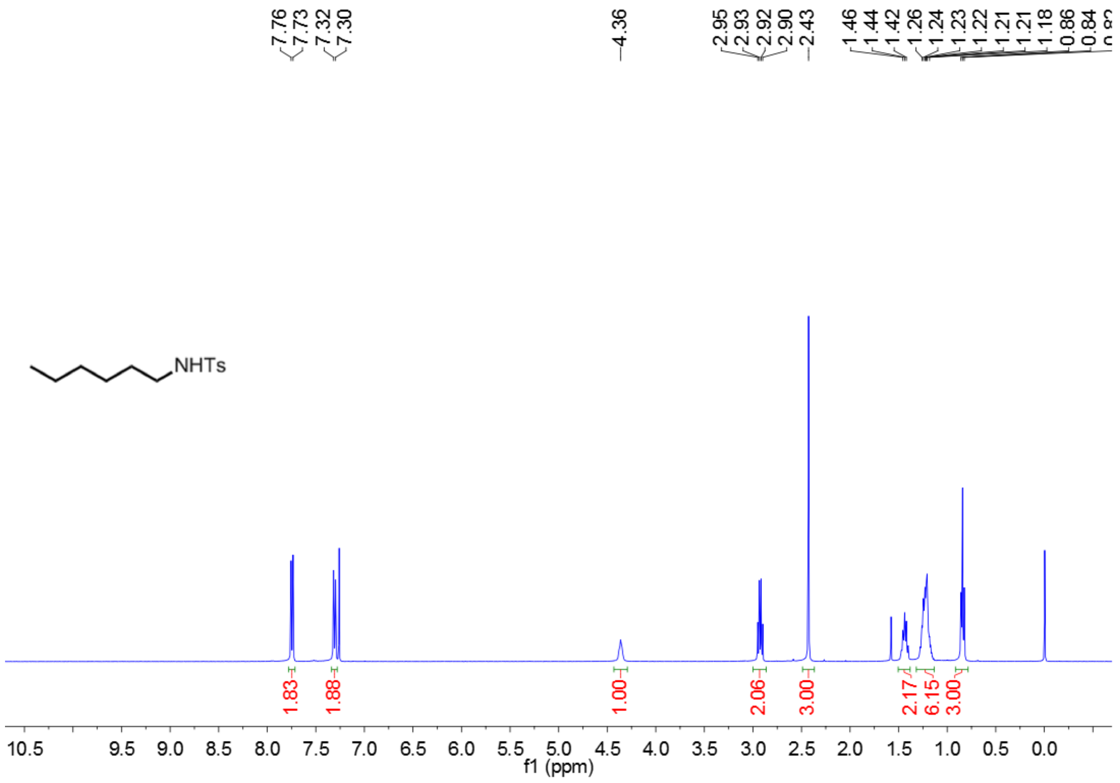

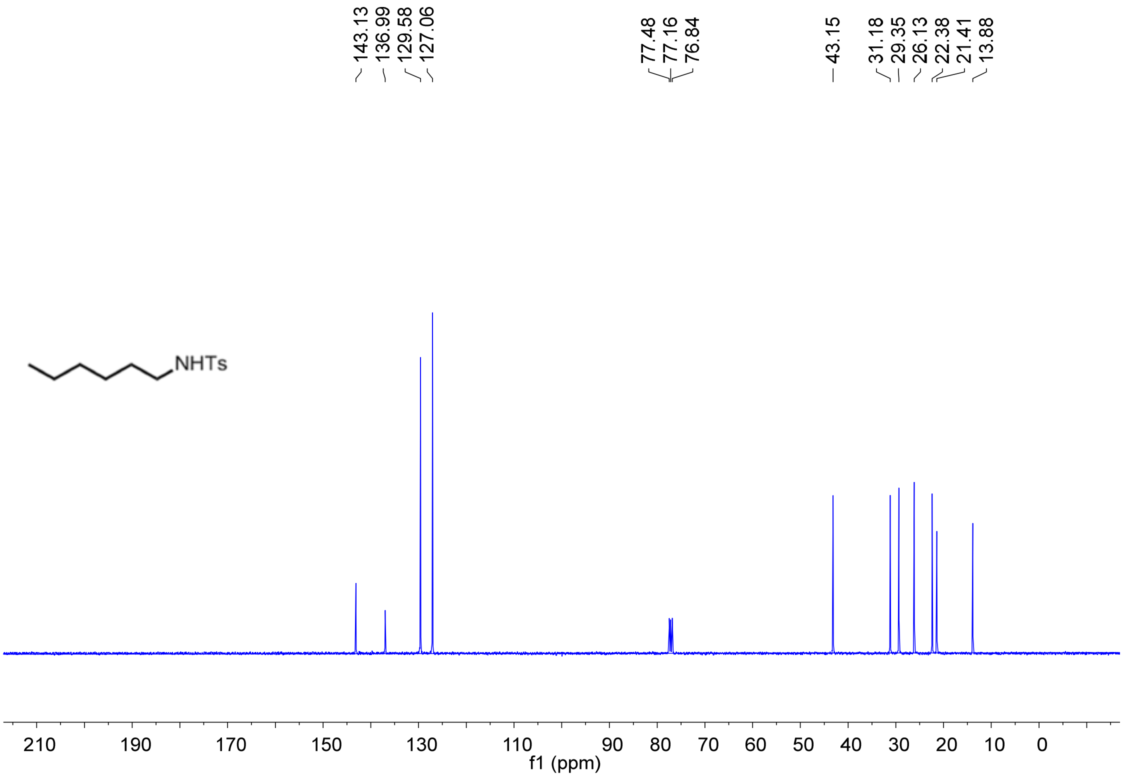

Supplementary Figure 28. ^{1}H and ^{13}C NMR spectra of compound **1t** in $CDCl_3$.

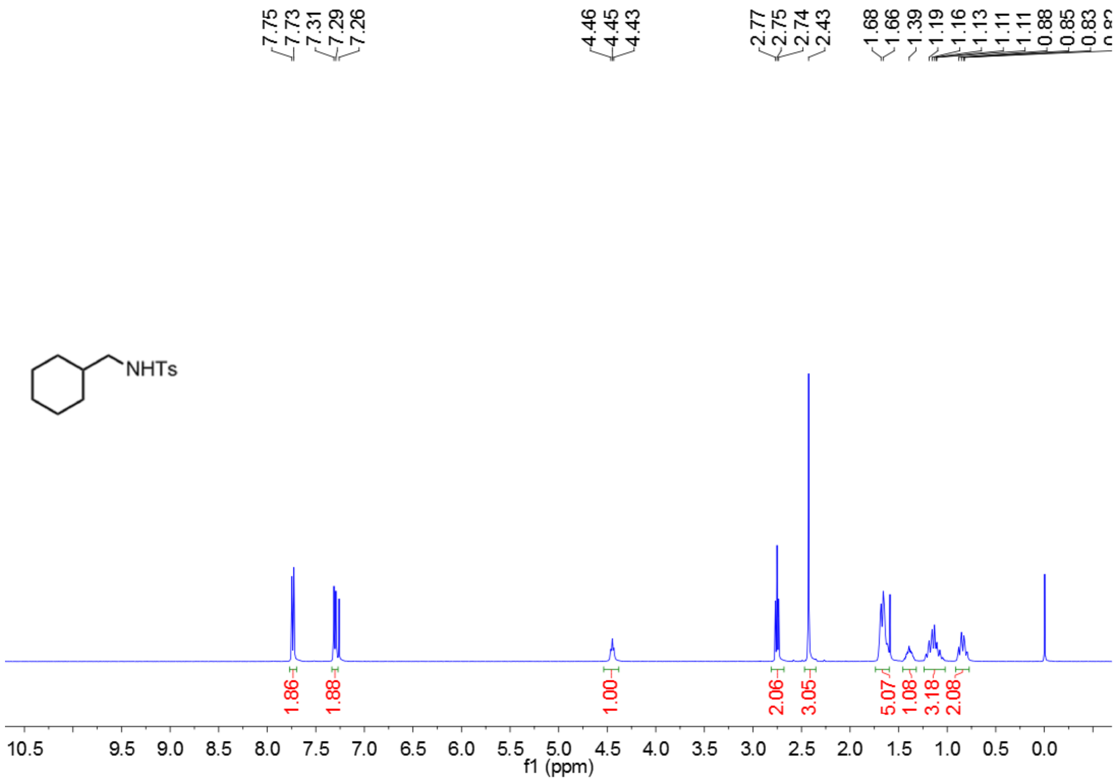

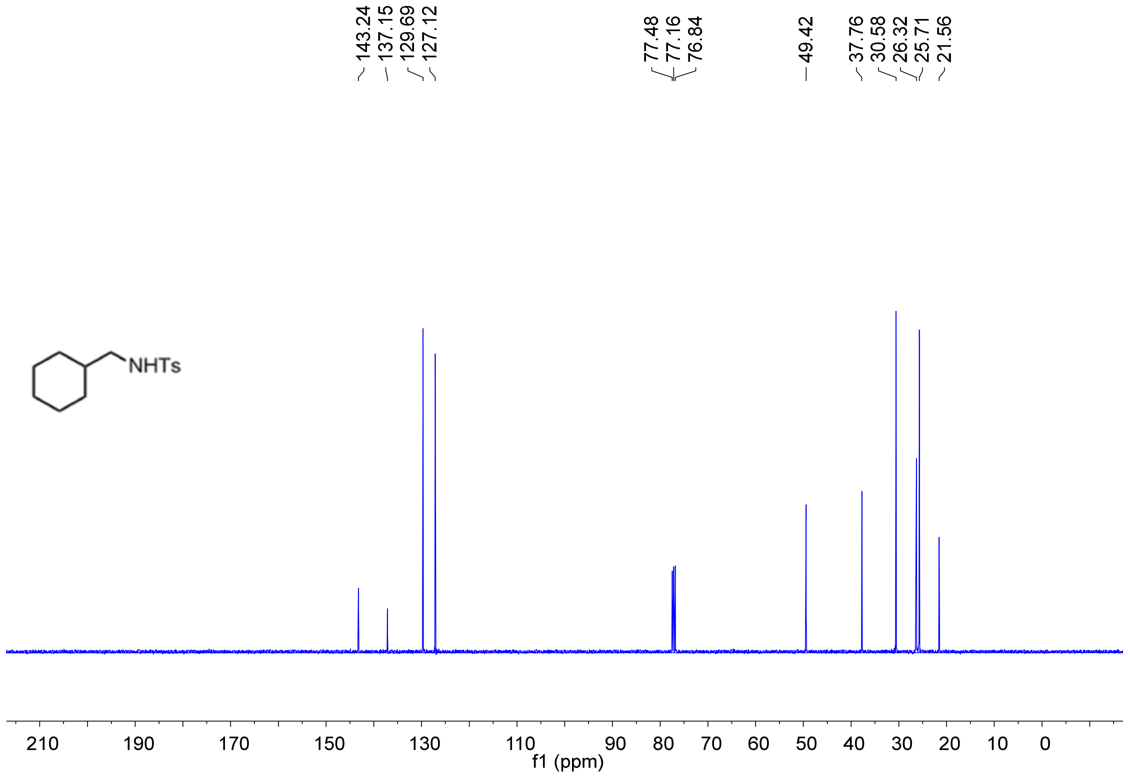

Supplementary Figure 29. ^{1}H and ^{13}C NMR spectra of compound **1u** in $CDCl_3$.

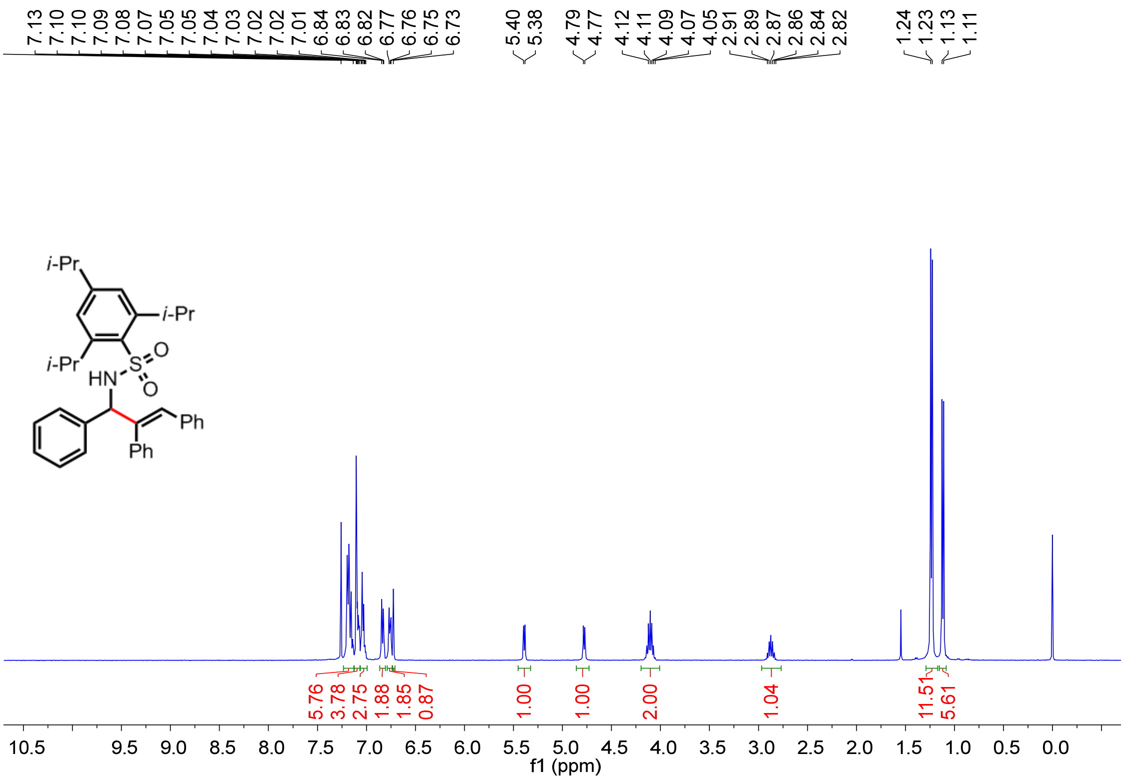

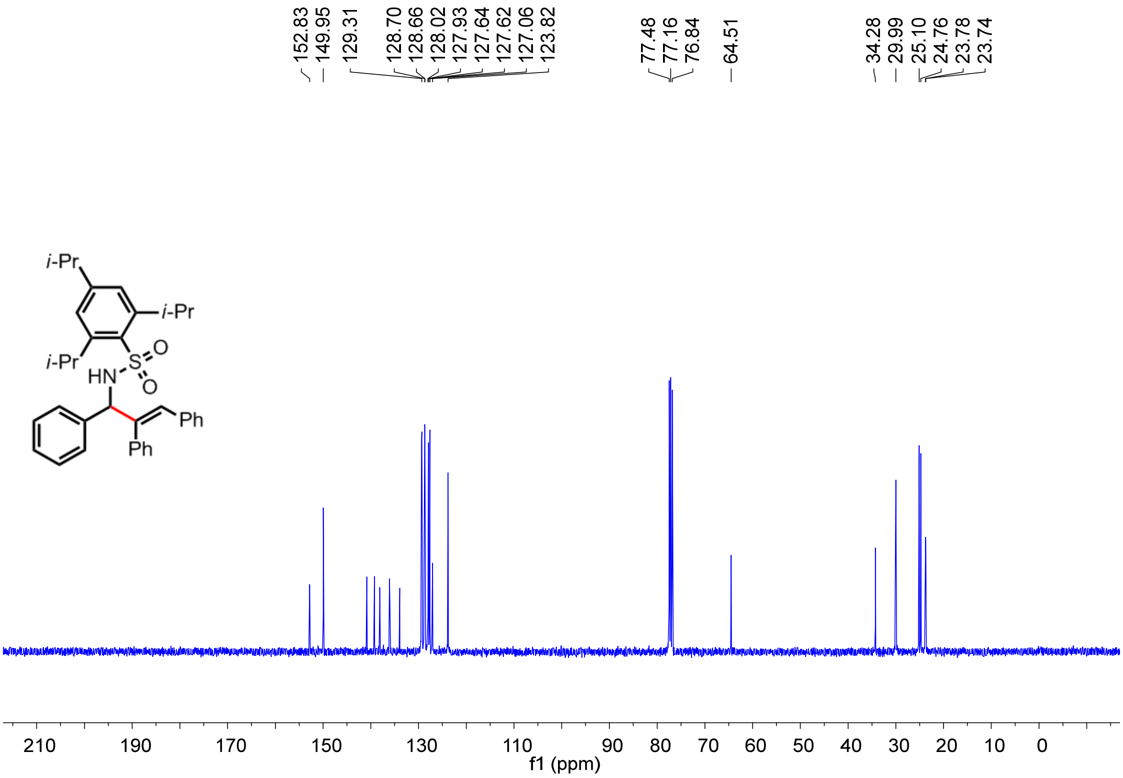

Supplementary Figure 30. ^{1}H and ^{13}C NMR spectra of compound **3a** in $CDCl_3$.

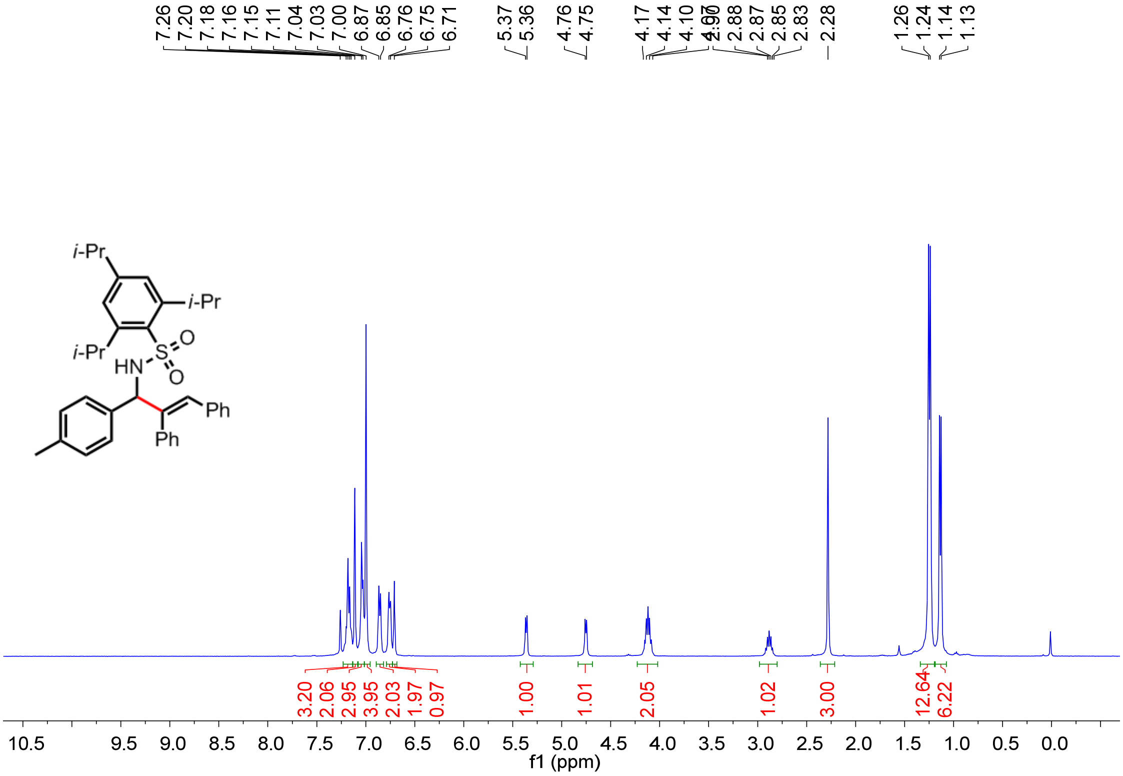

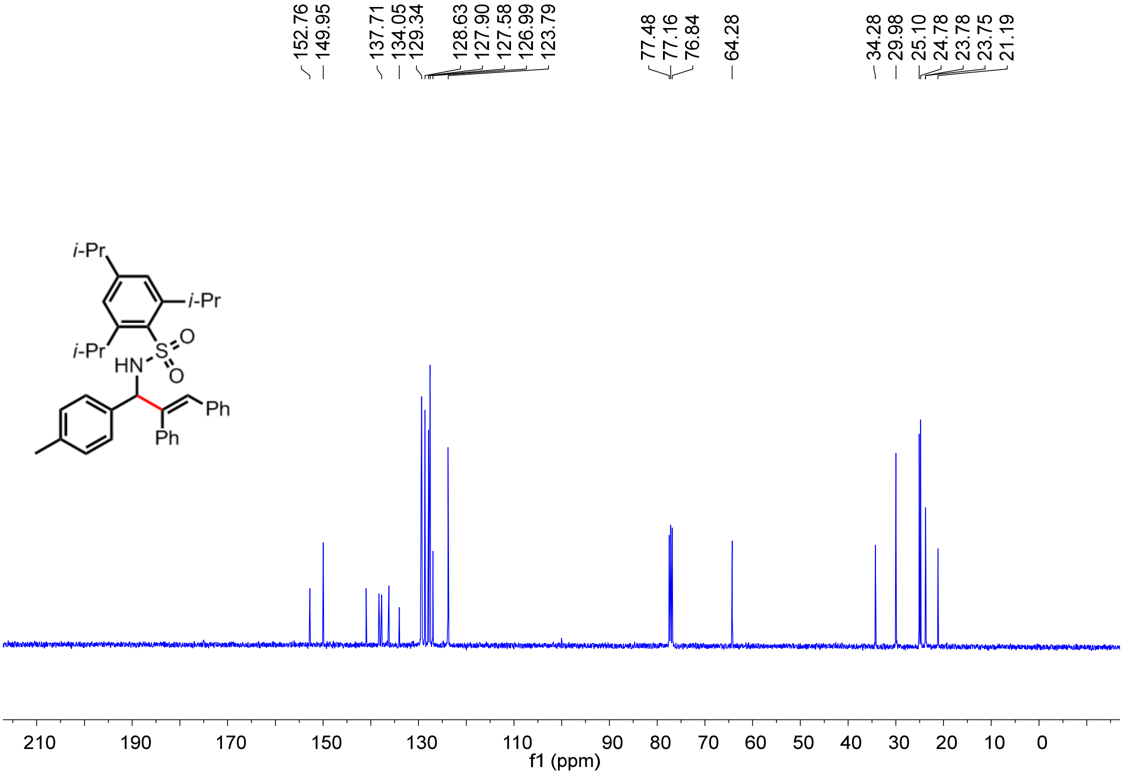

Supplementary Figure 31. ^{1}H and ^{13}C NMR spectra of compound **3b** in $CDCl_3$.

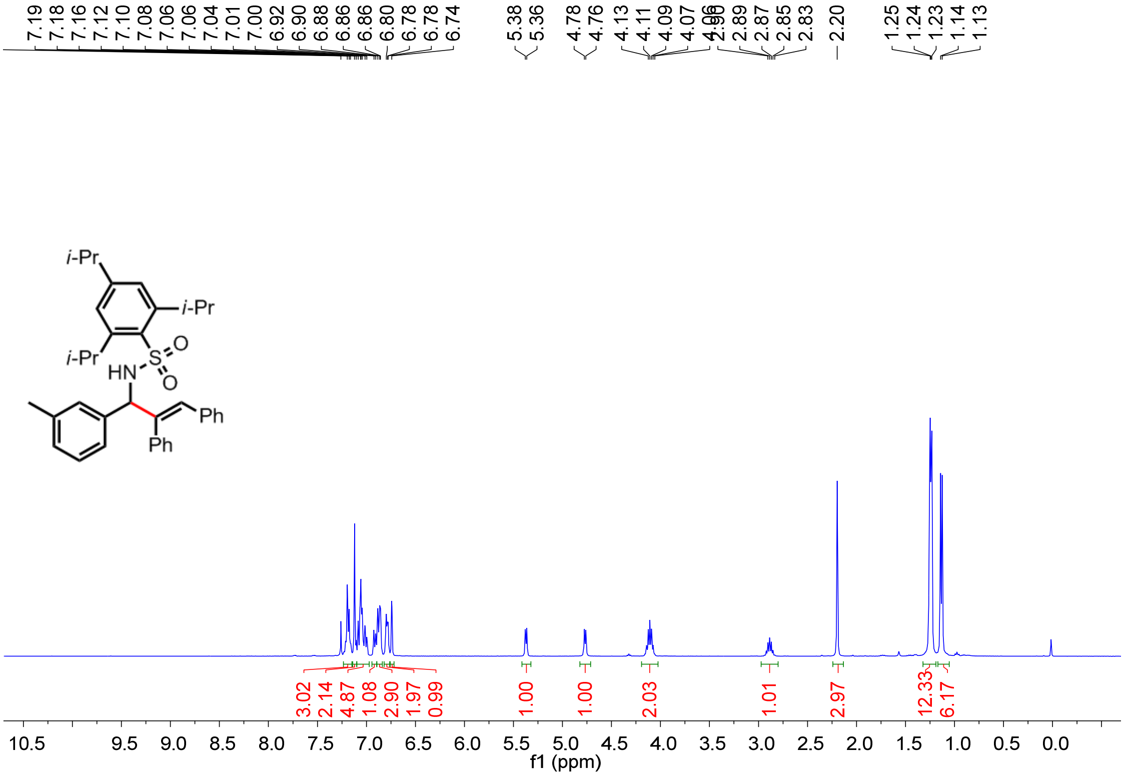

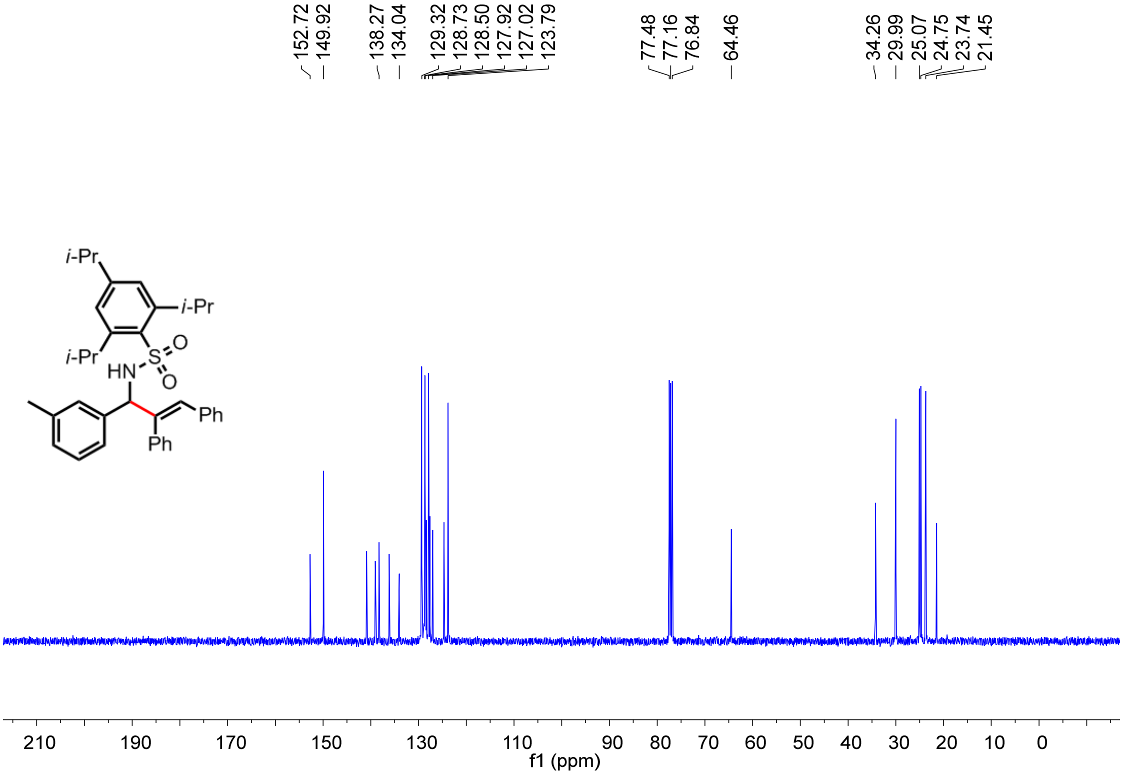

Supplementary Figure 32. ^{1}H and ^{13}C NMR spectra of compound **3c** in $CDCl_3$.

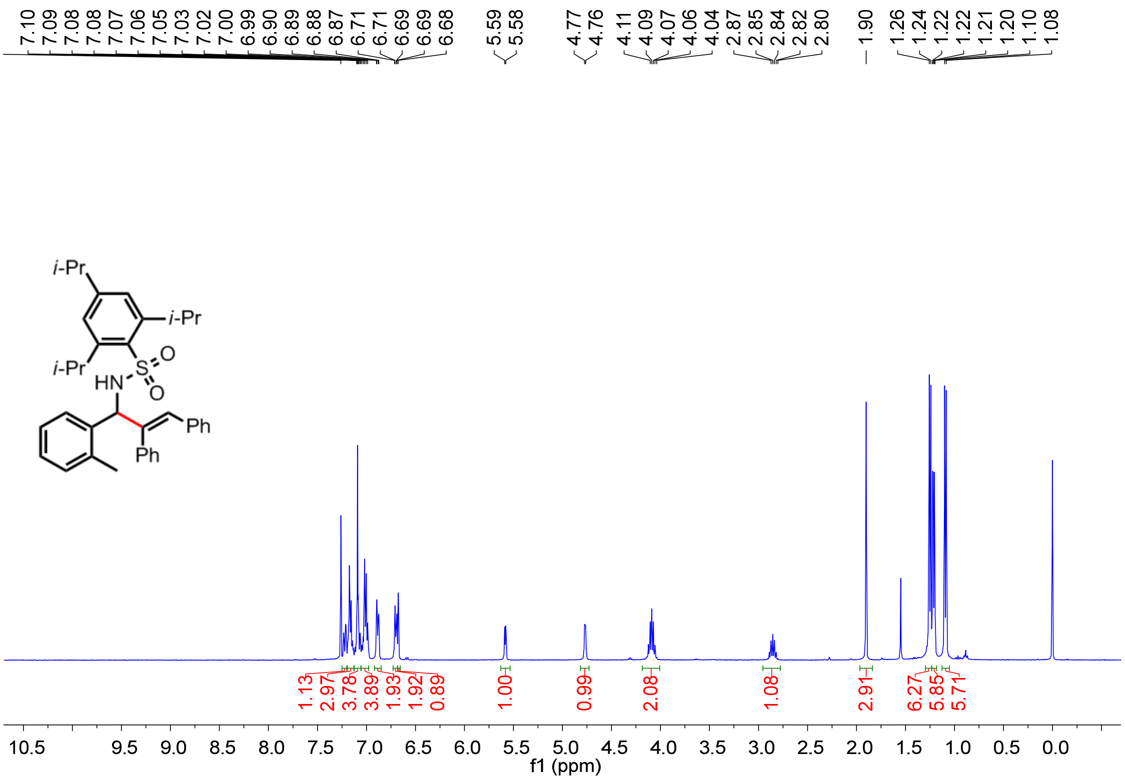

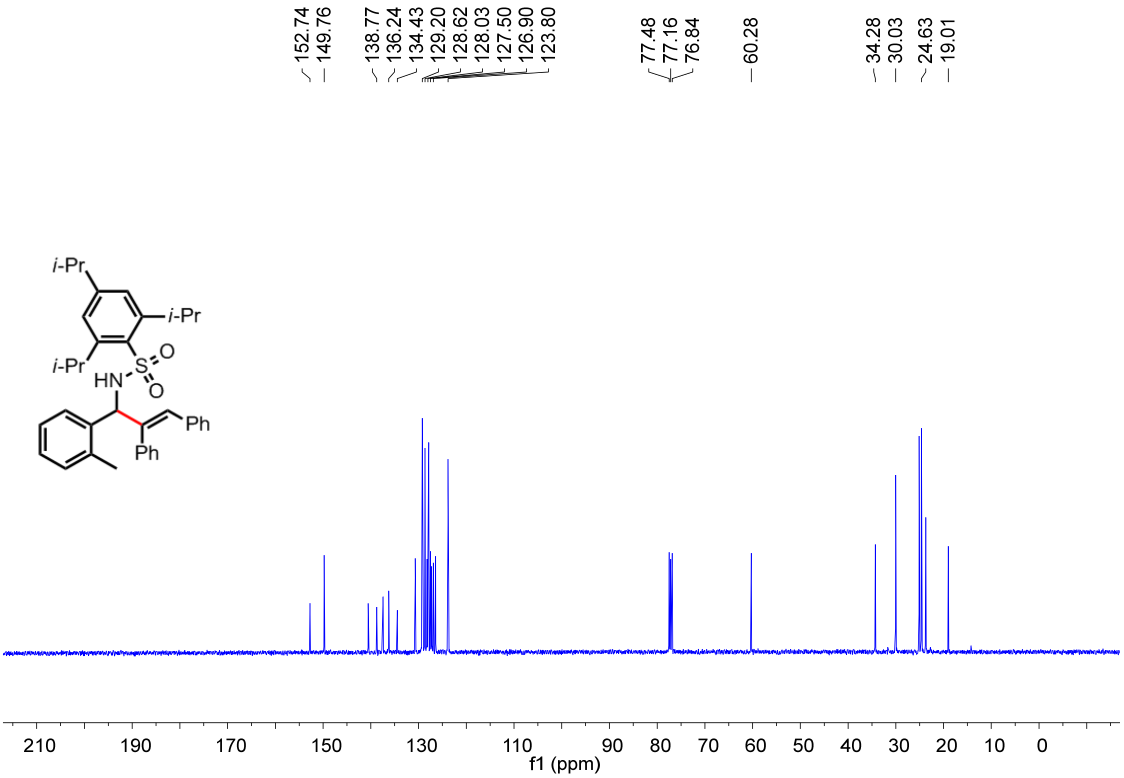

Supplementary Figure 33. ^{1}H and ^{13}C NMR spectra of compound **3d** in $CDCl_3$.

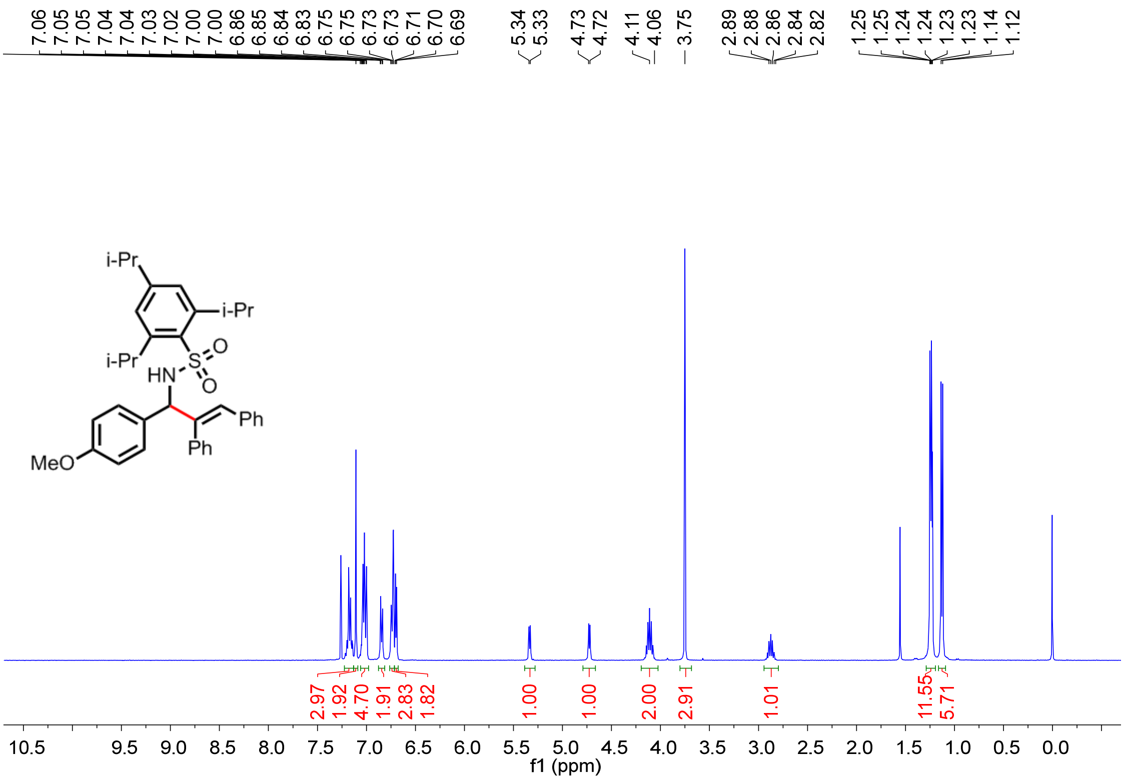

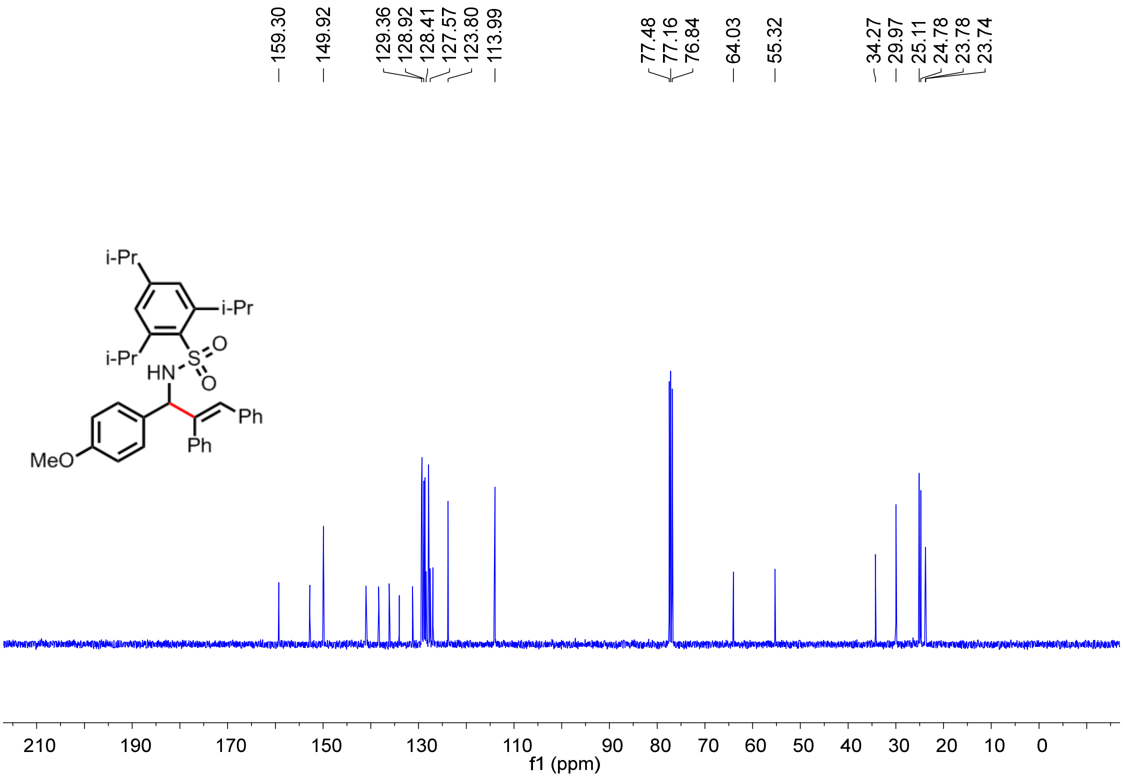

Supplementary Figure 34. ^{1}H and ^{13}C NMR spectra of compound **3e** in $CDCl_3$.

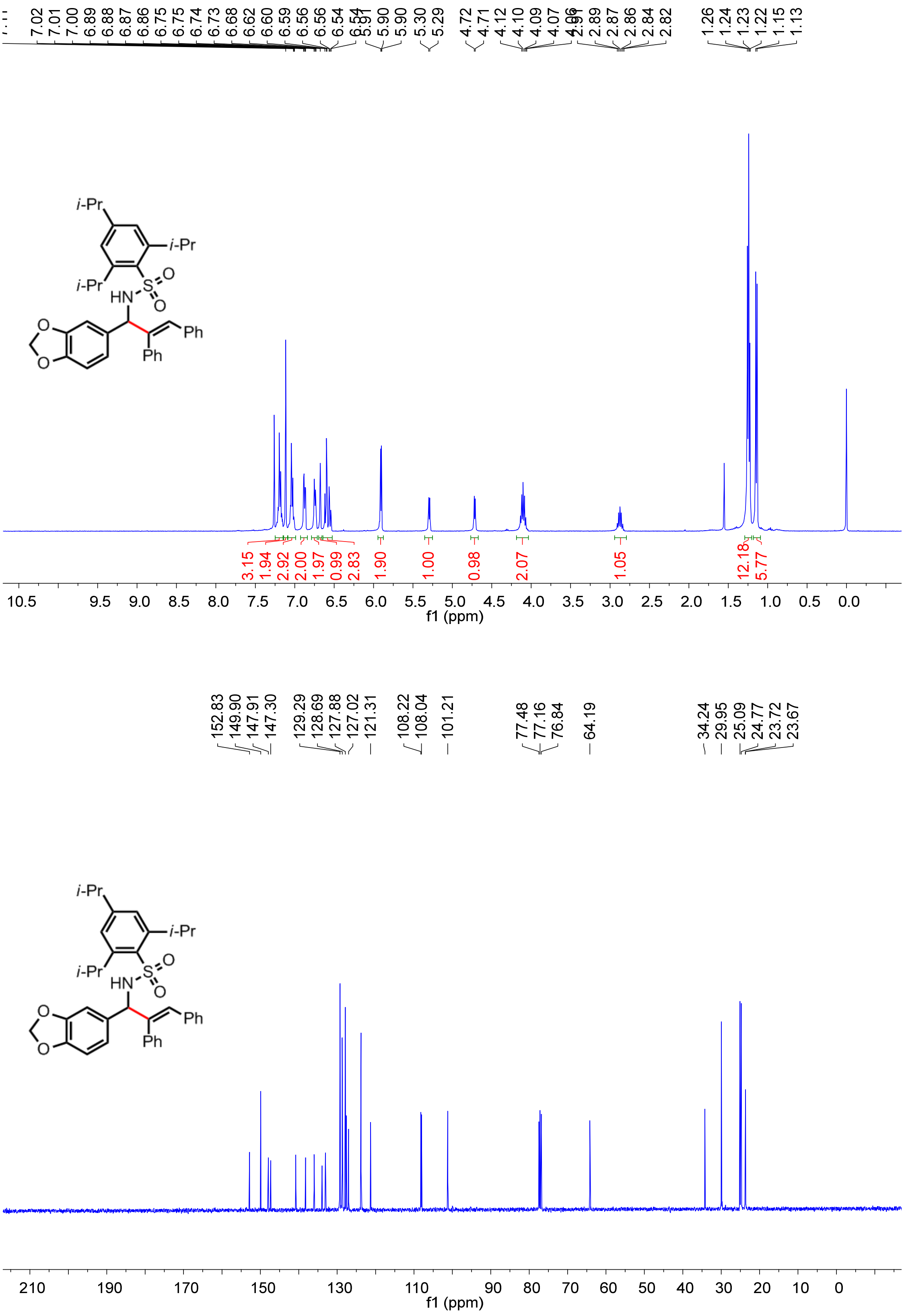

Supplementary Figure 35. ^{1}H and ^{13}C NMR spectra of compound **3f** in $CDCl_3$.

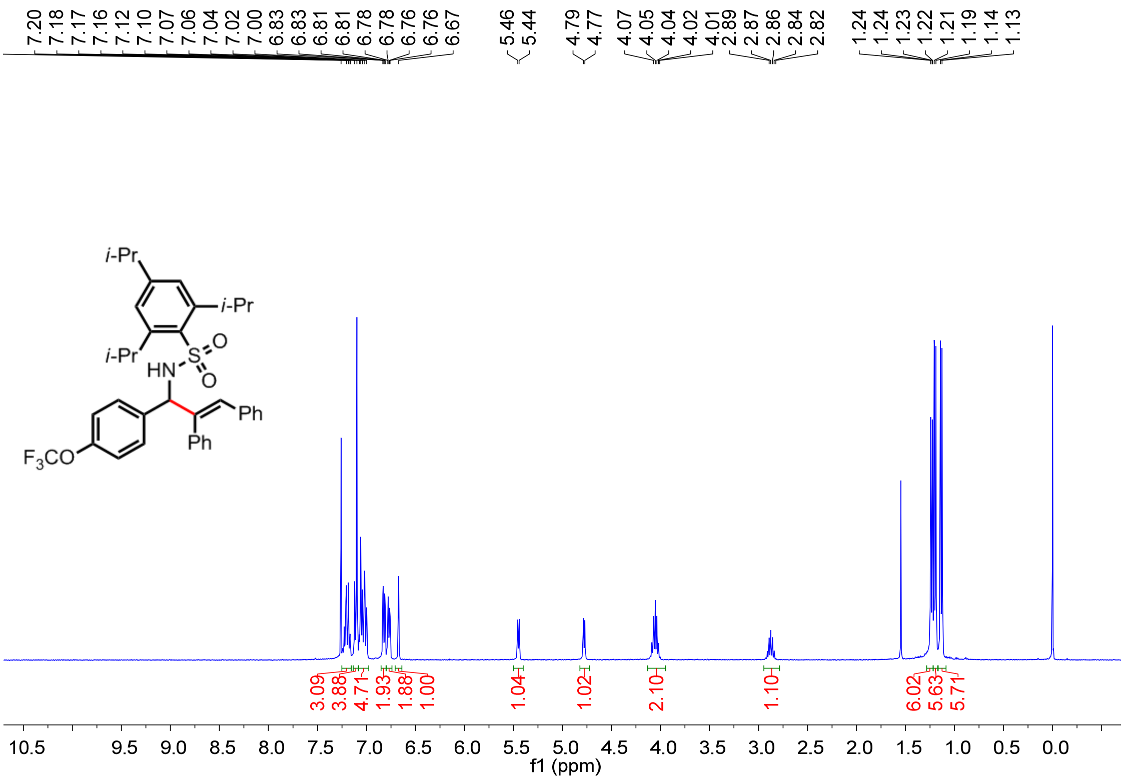

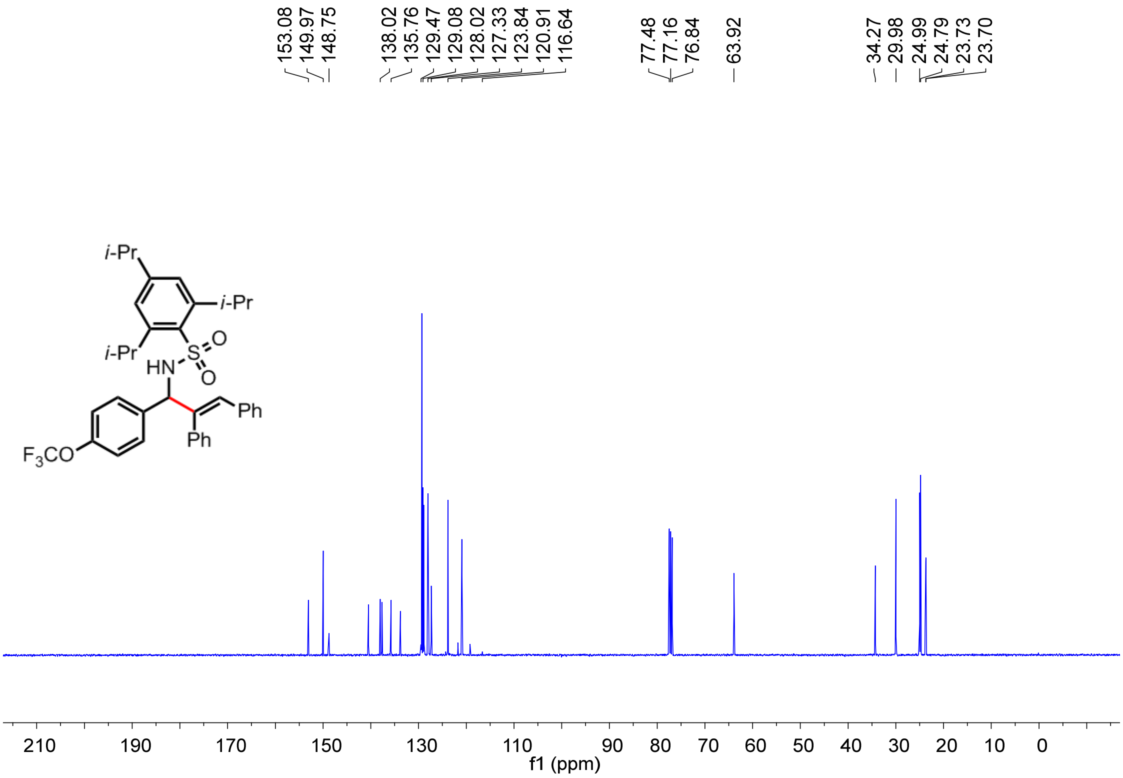

Supplementary Figure 36. ^{1}H and ^{13}C NMR spectra of compound **3g** in $CDCl_3$.

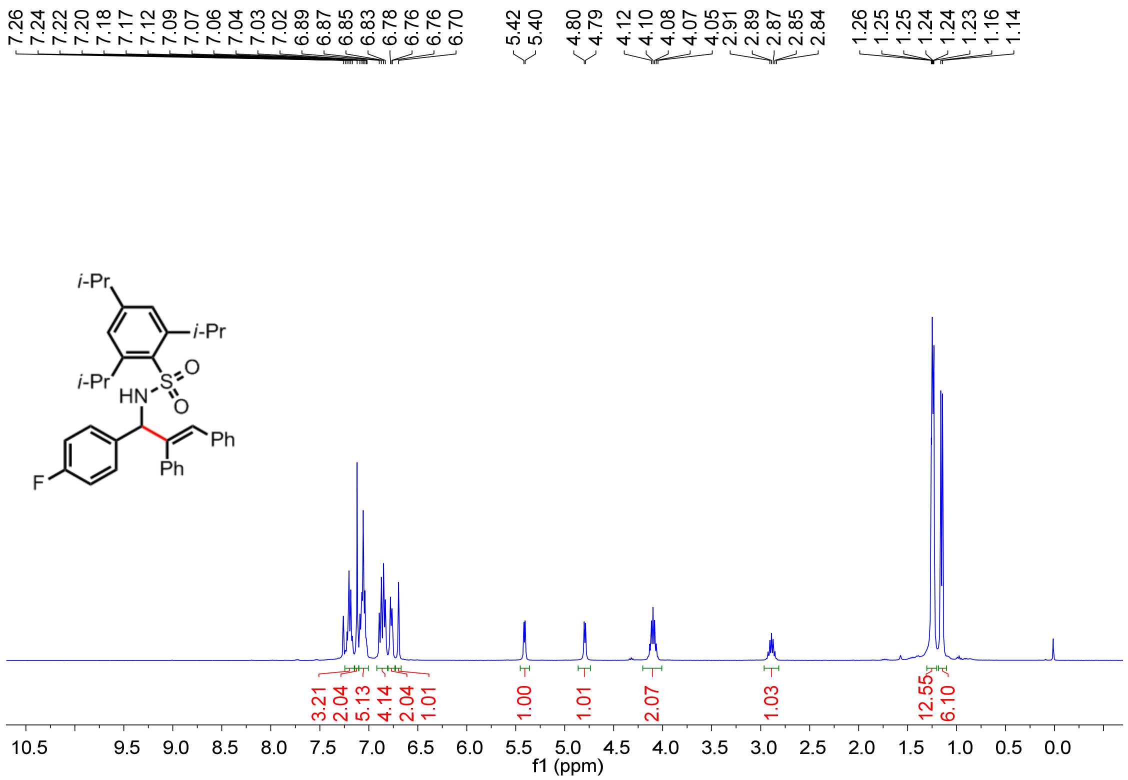

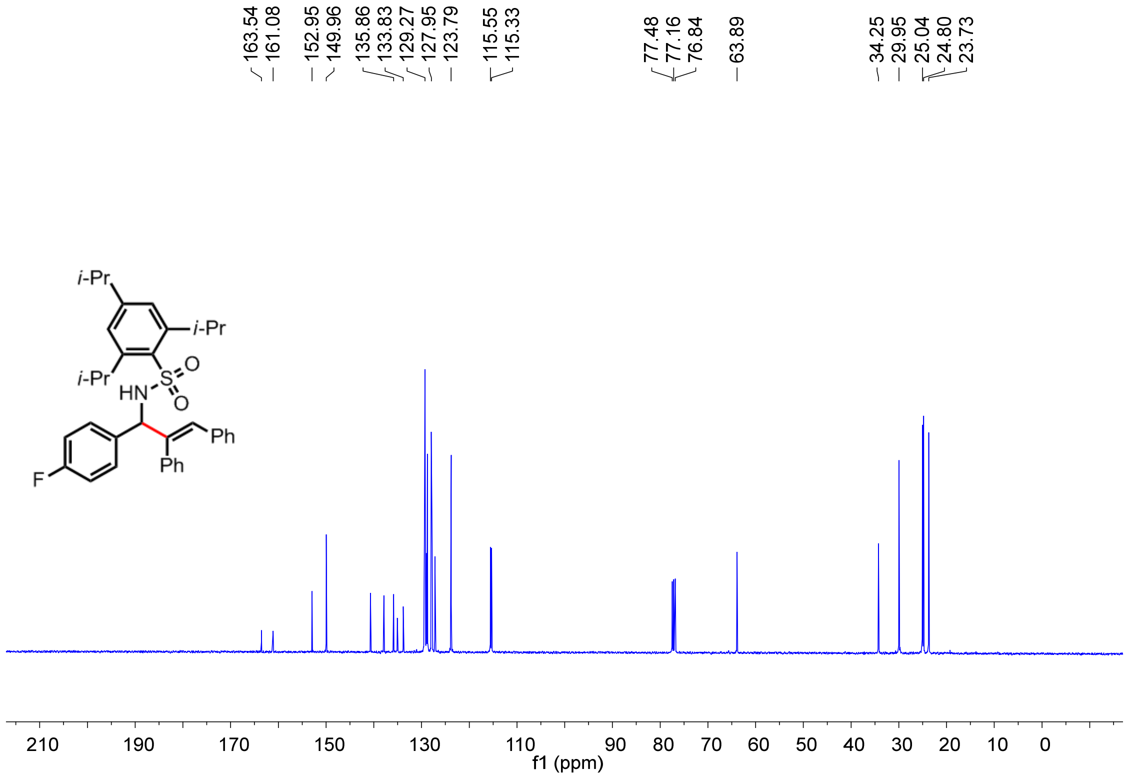

Supplementary Figure 37. ^{1}H and ^{13}C NMR spectra of compound **3h** in $CDCl_3$.

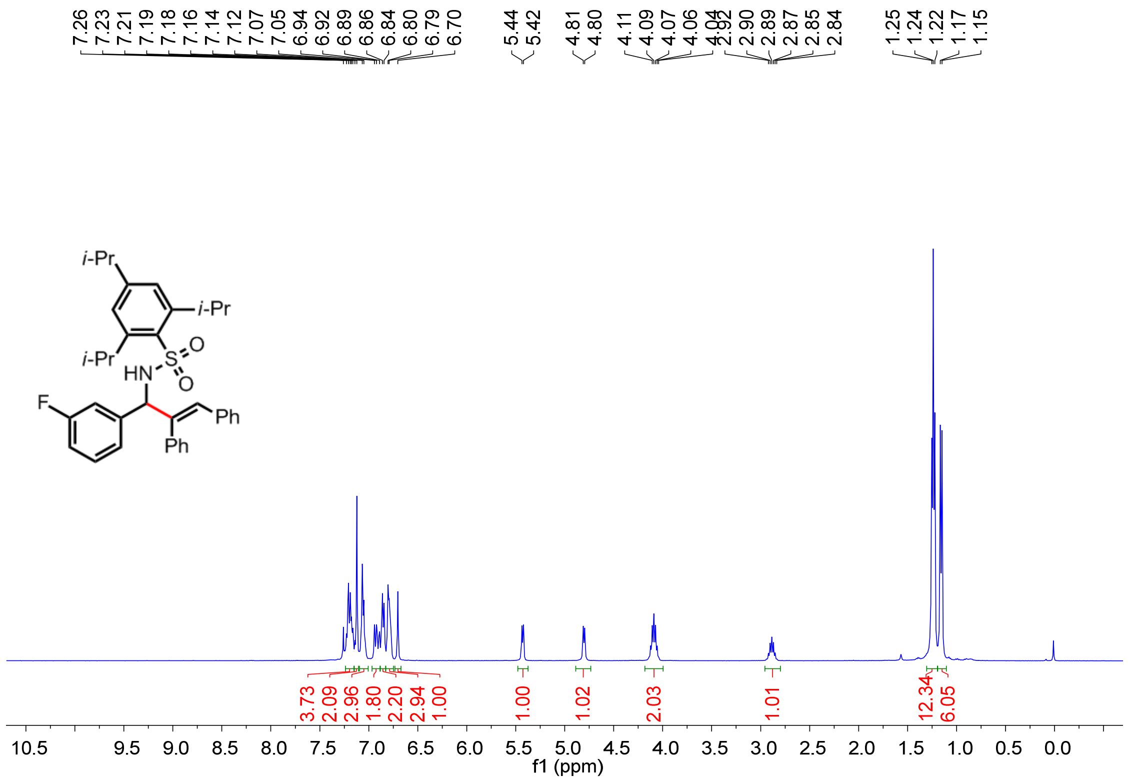

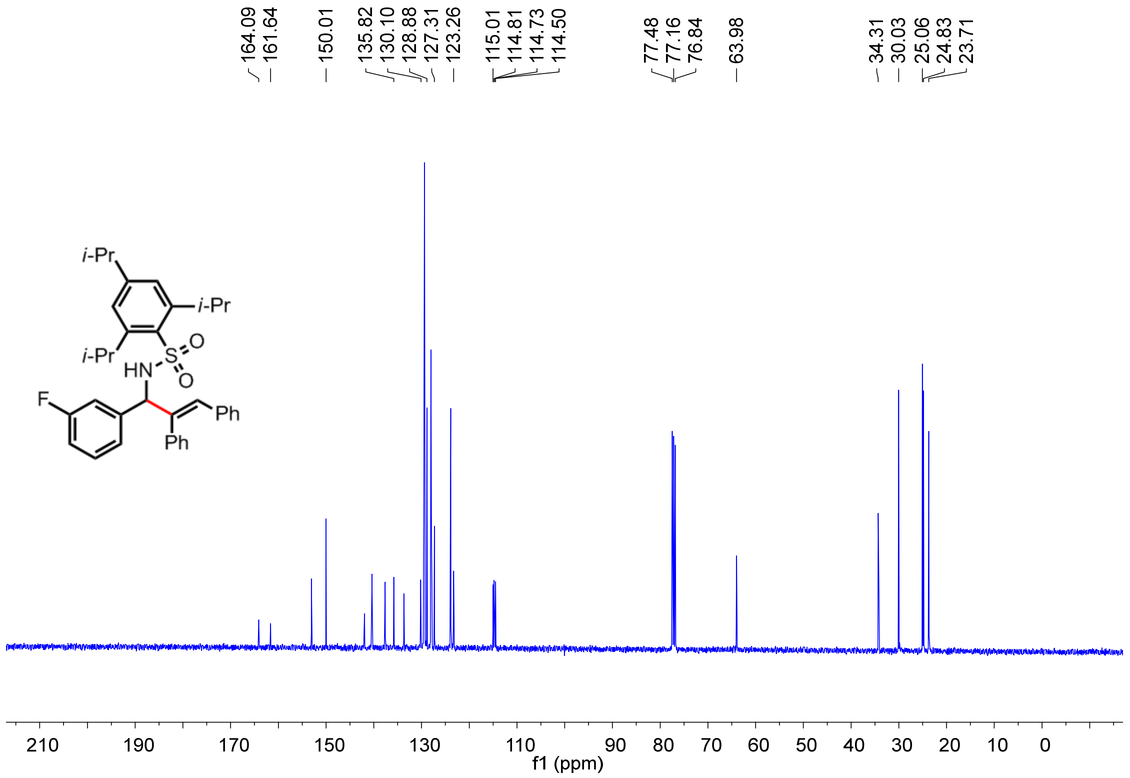

Supplementary Figure 38. ^{1}H and ^{13}C NMR spectra of compound **3i** in $CDCl_3$.

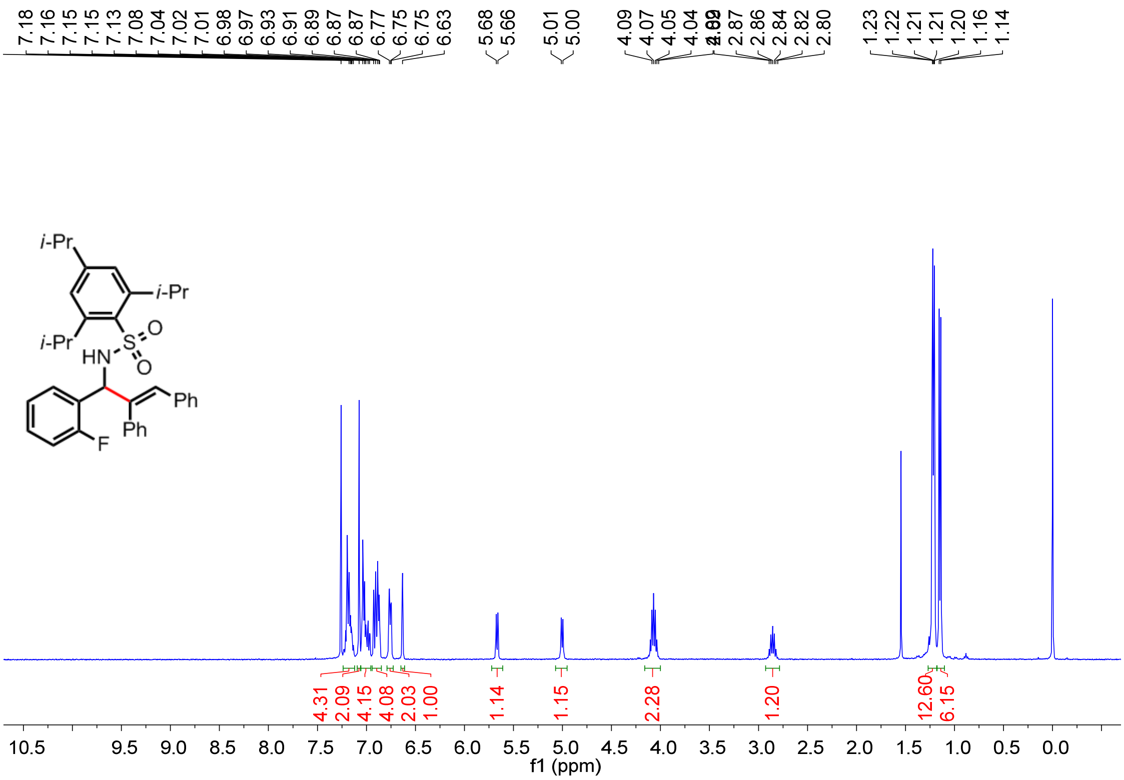

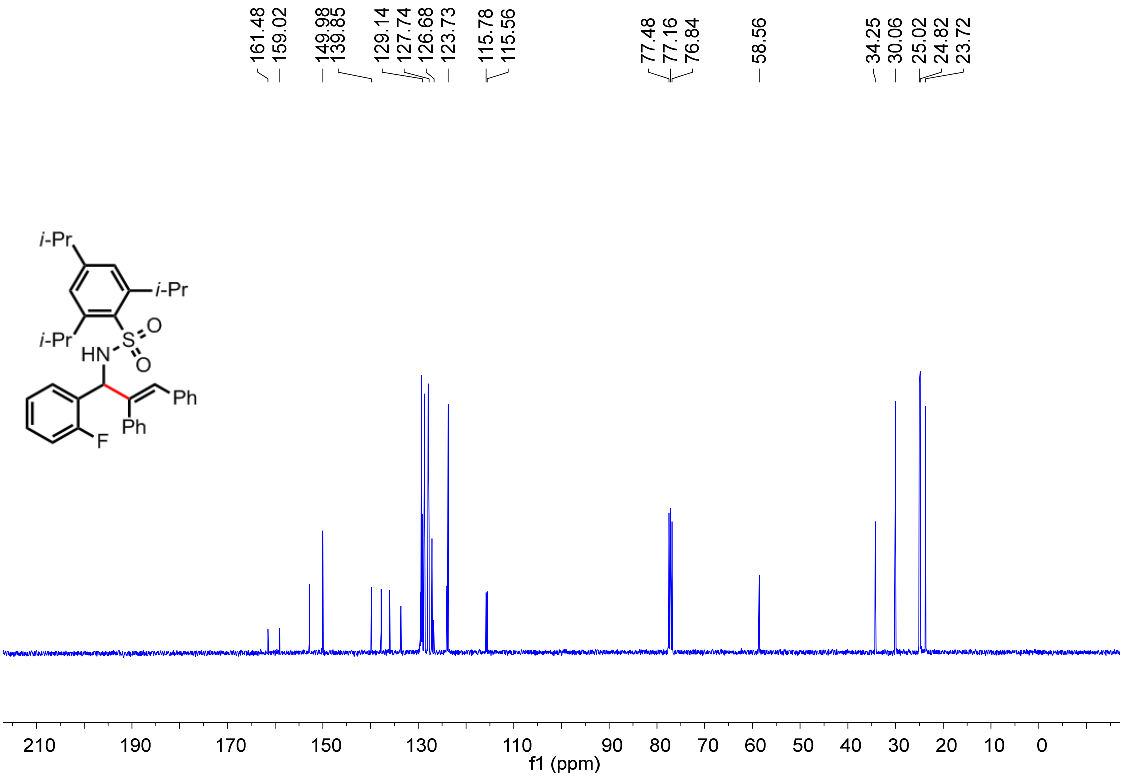

Supplementary Figure 39. ^{1}H and ^{13}C NMR spectra of compound **3j** in $CDCl_3$.

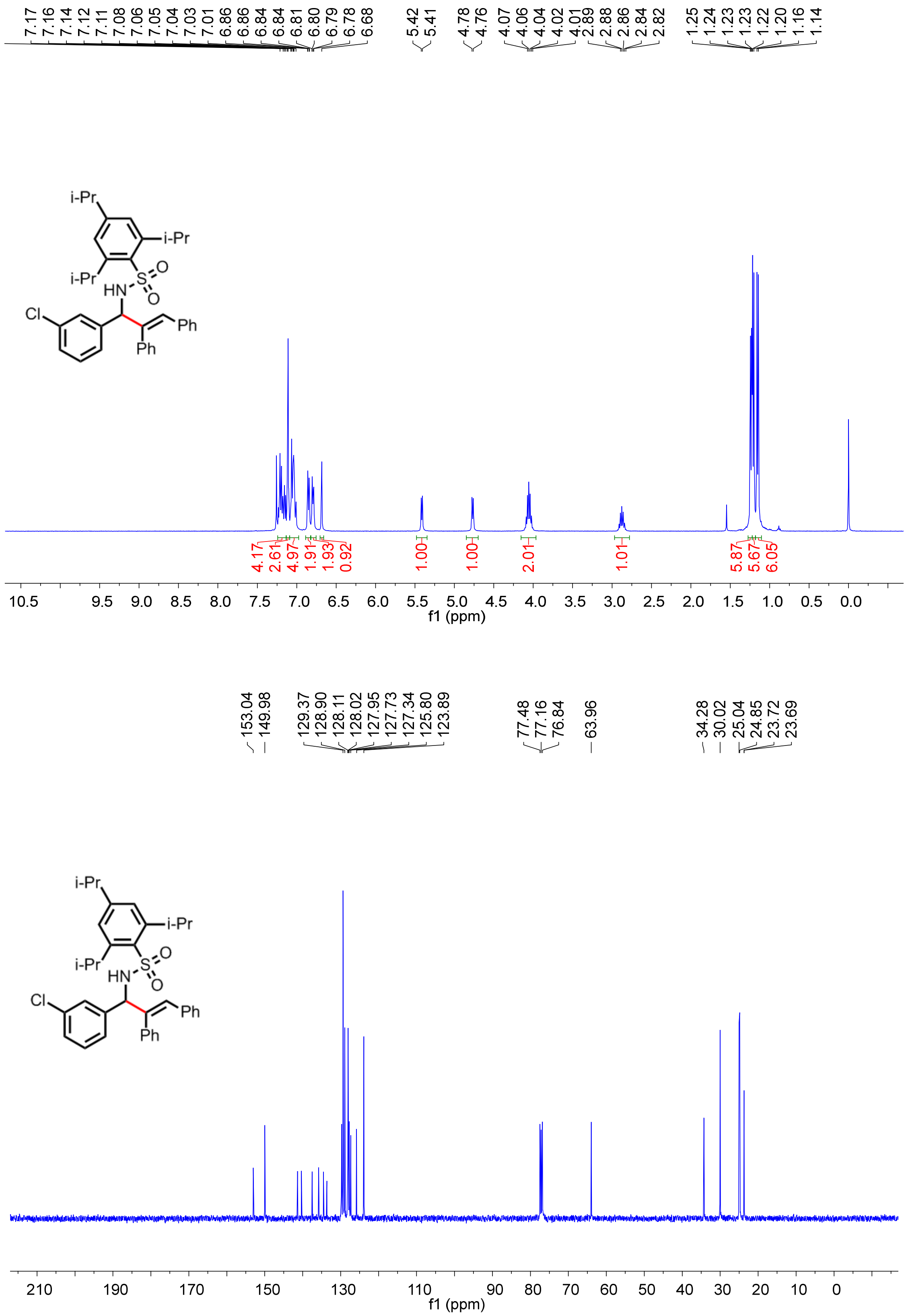

Supplementary Figure 40. ^{1}H and ^{13}C NMR spectra of compound **3k** in $CDCl_3$.

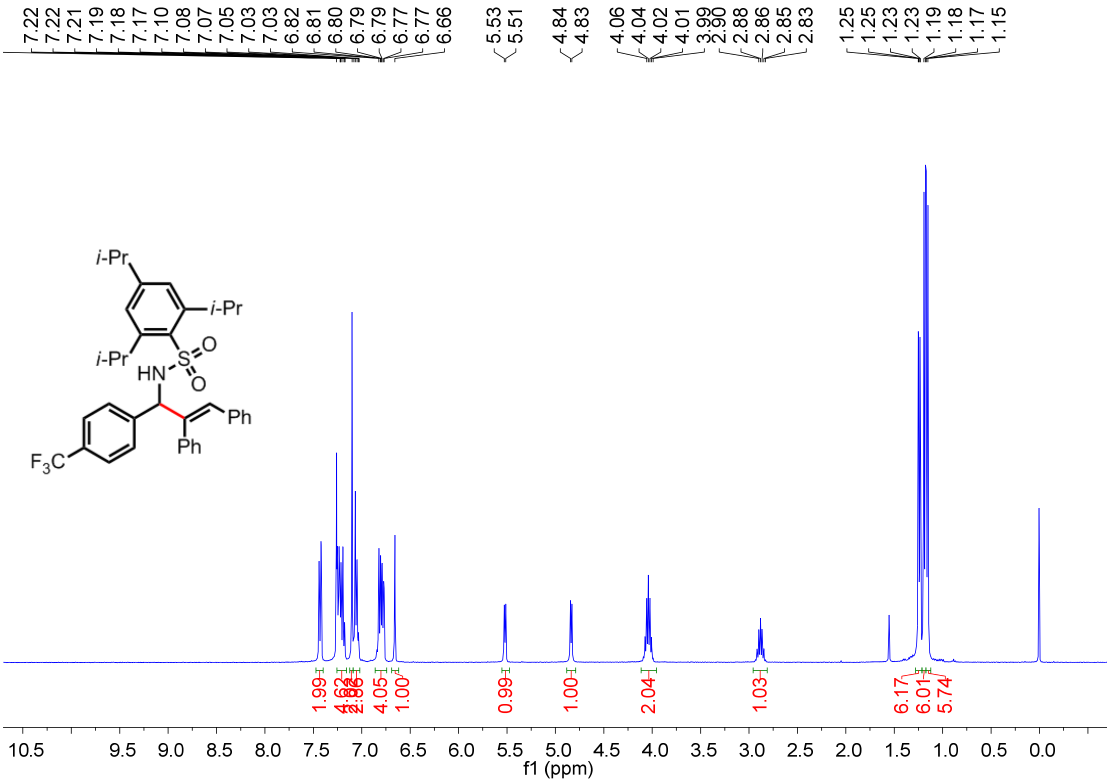

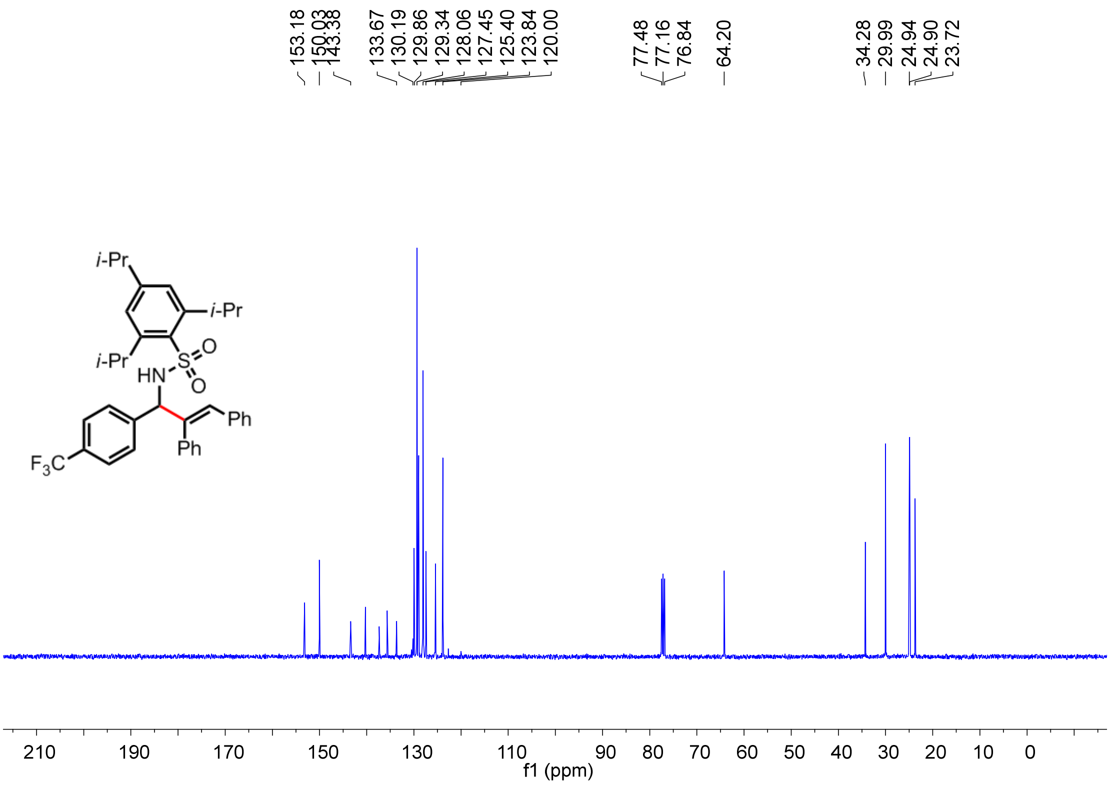

Supplementary Figure 41. ^{1}H and ^{13}C NMR spectra of compound **3l** in $CDCl_3$.

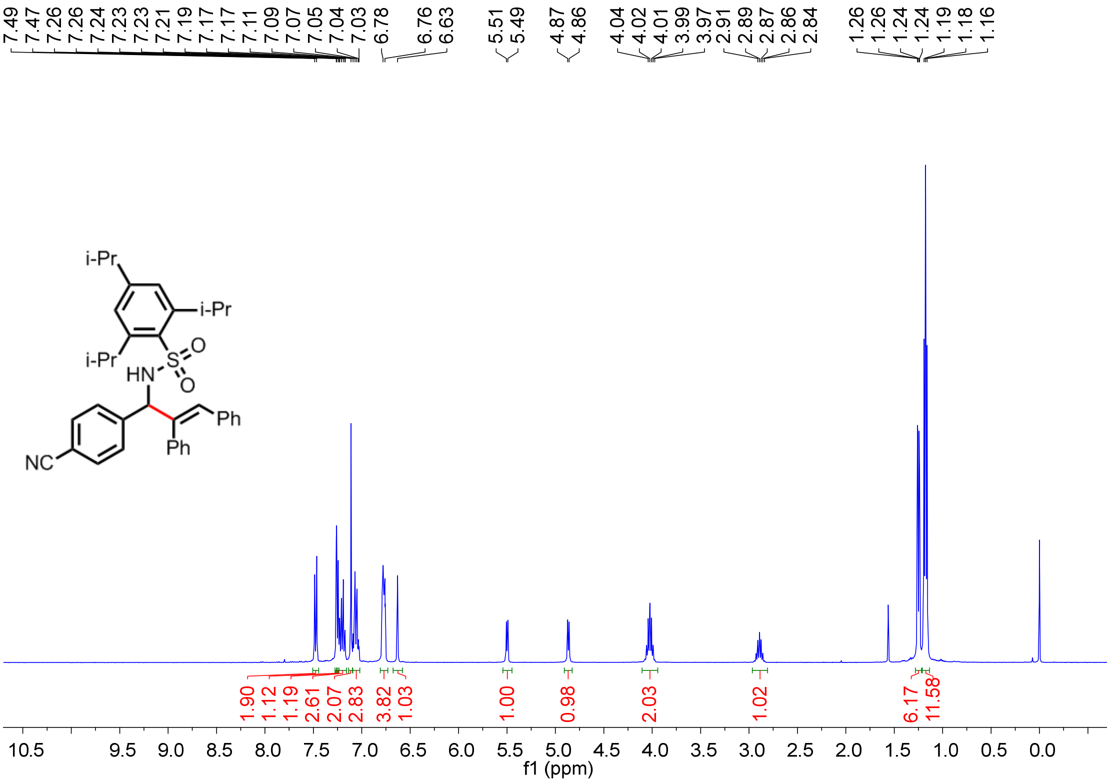

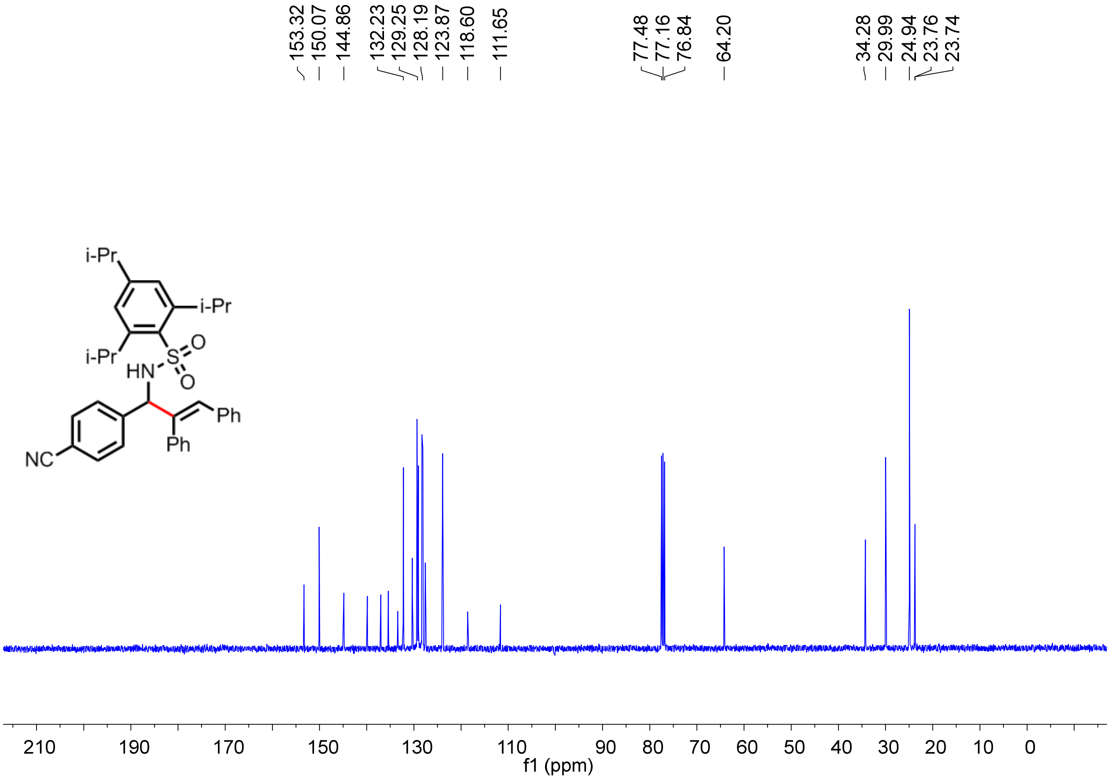

Supplementary Figure 42. ^{1}H and ^{13}C NMR spectra of compound **3m** in $CDCl_3$.

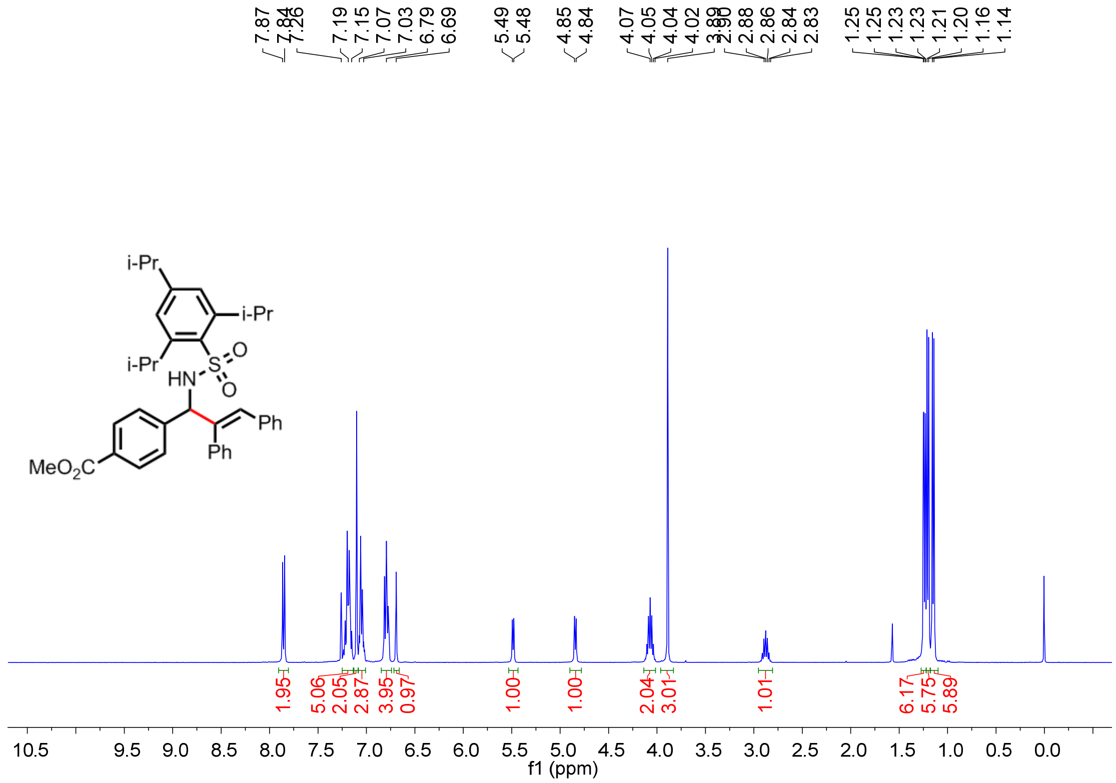

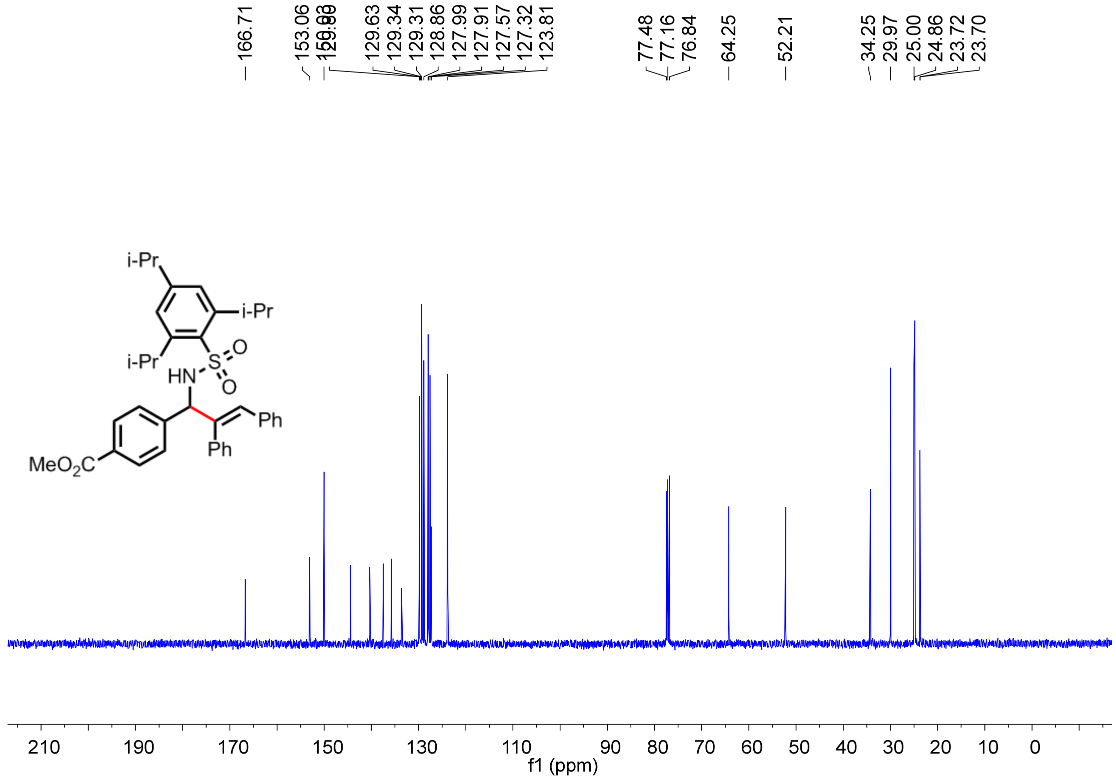

Supplementary Figure 43. ^{1}H and ^{13}C NMR spectra of compound **3n** in $CDCl_3$.

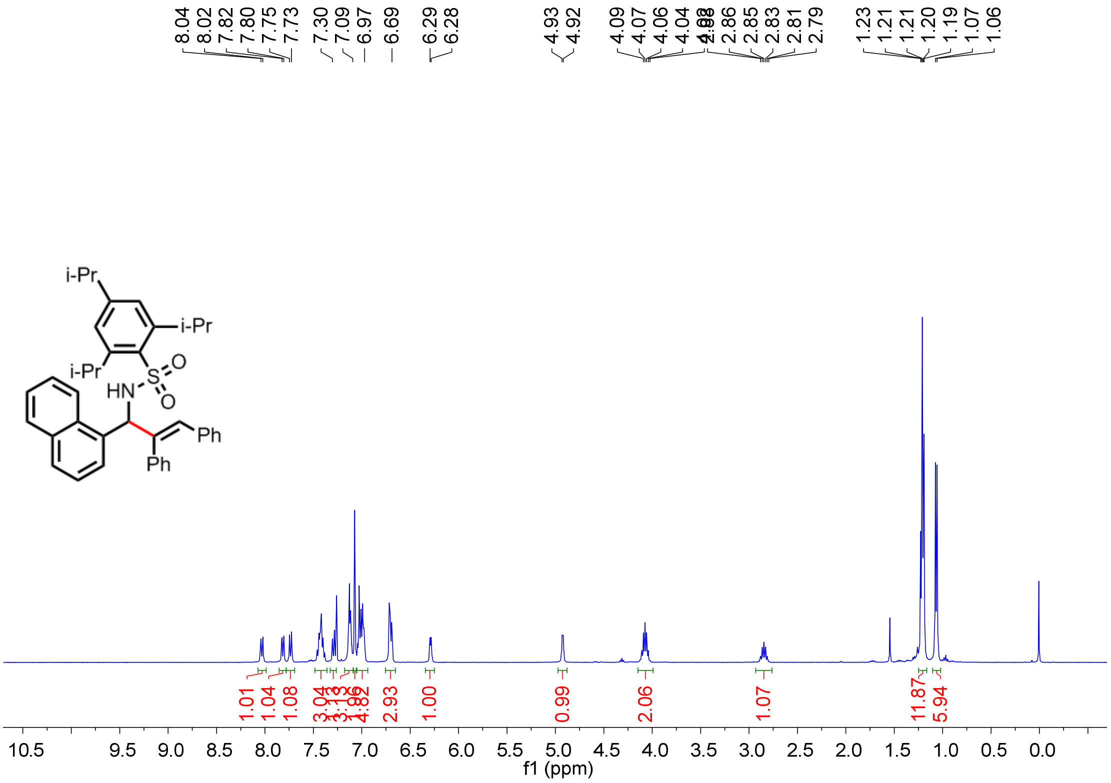

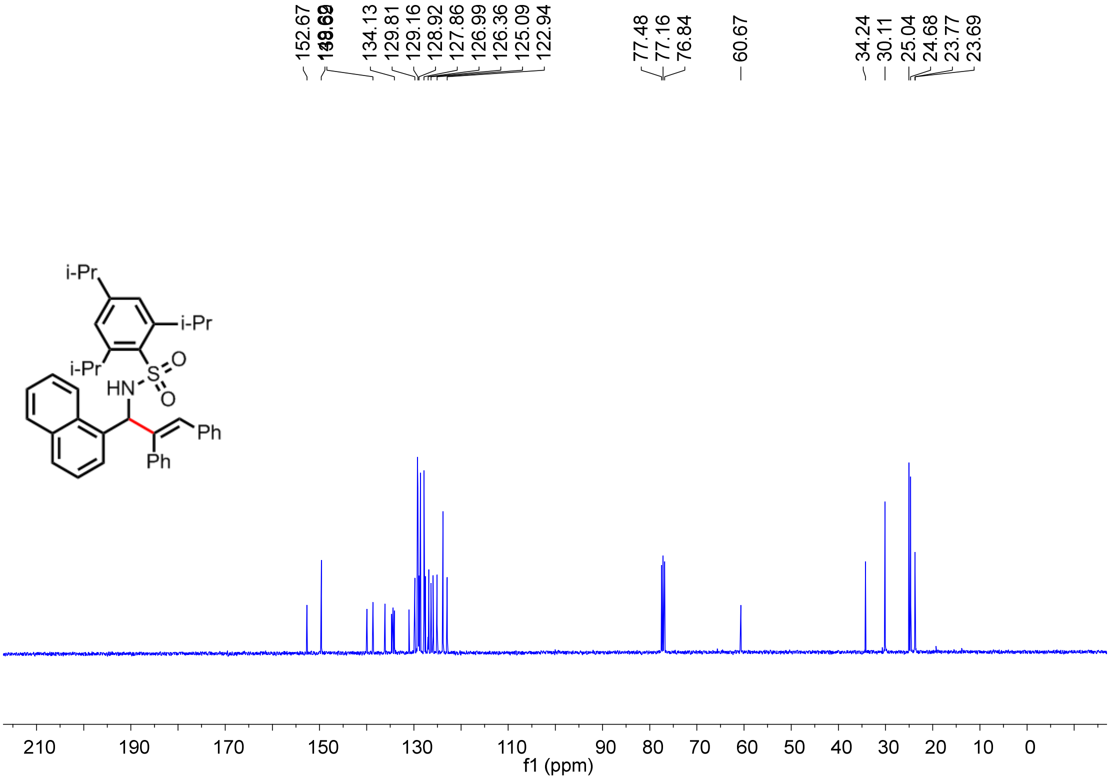

Supplementary Figure 44. ^{1}H and ^{13}C NMR spectra of compound **3o** in $CDCl_3$.

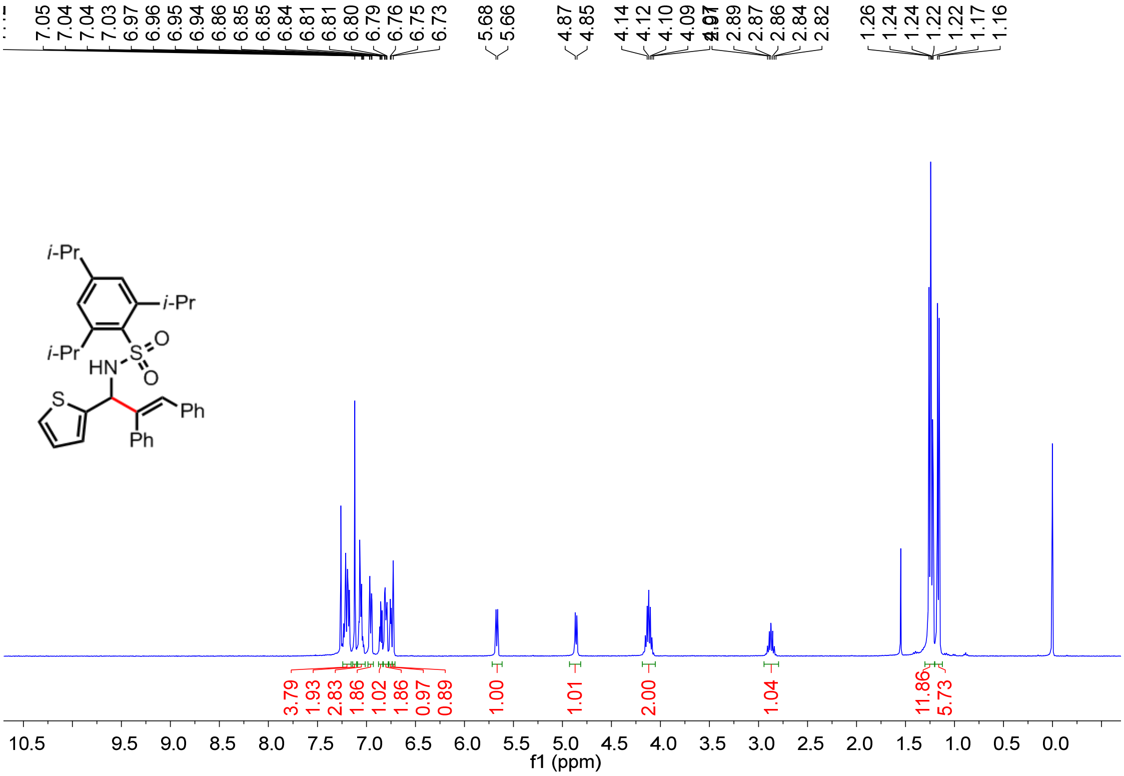

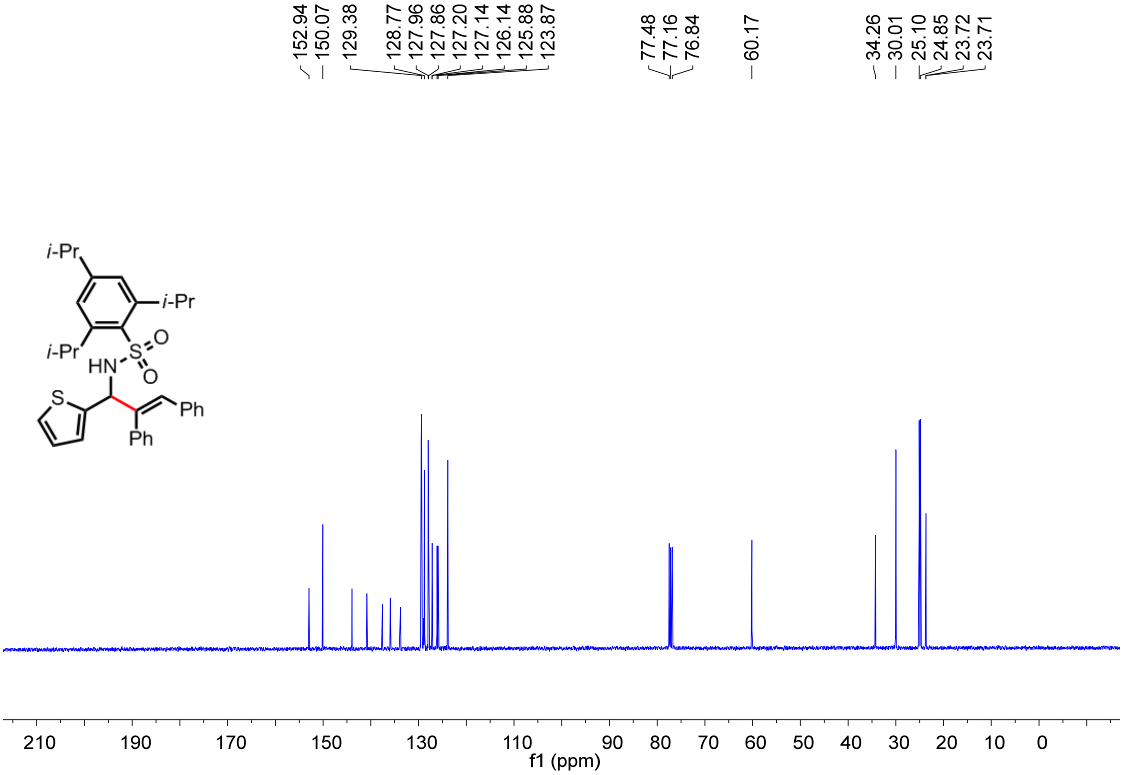

Supplementary Figure 45. ^{1}H and ^{13}C NMR spectra of compound **3p** in $CDCl_3$.

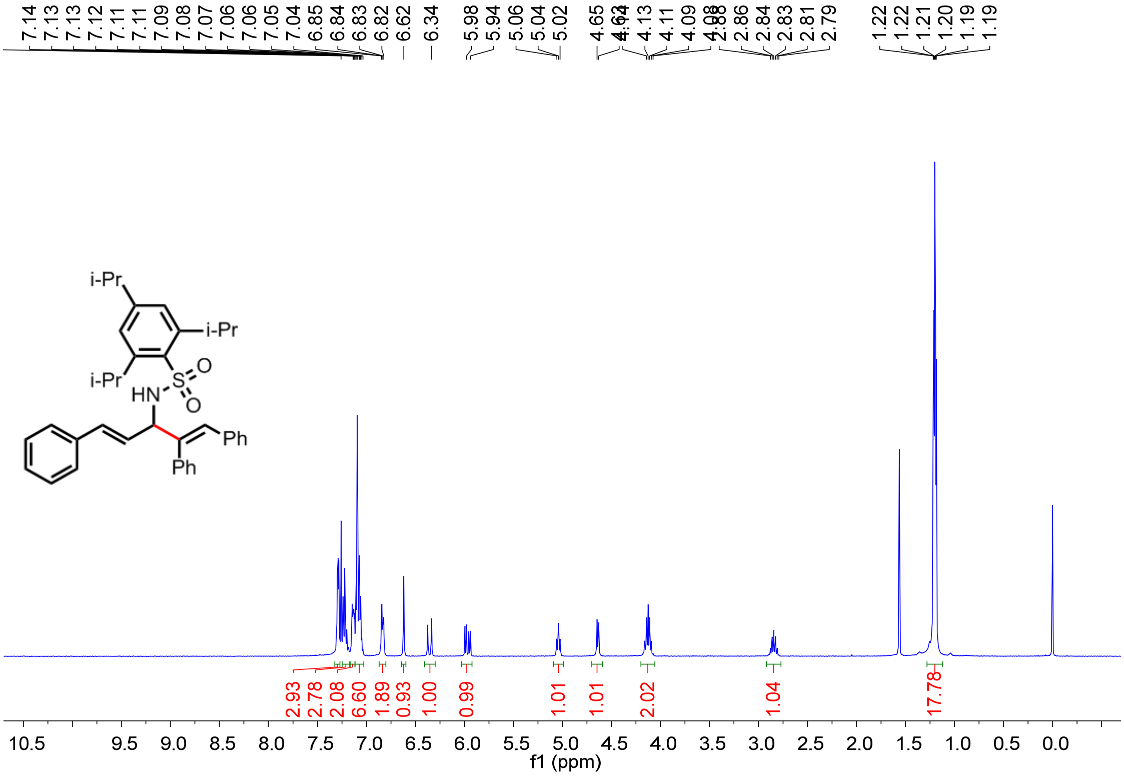

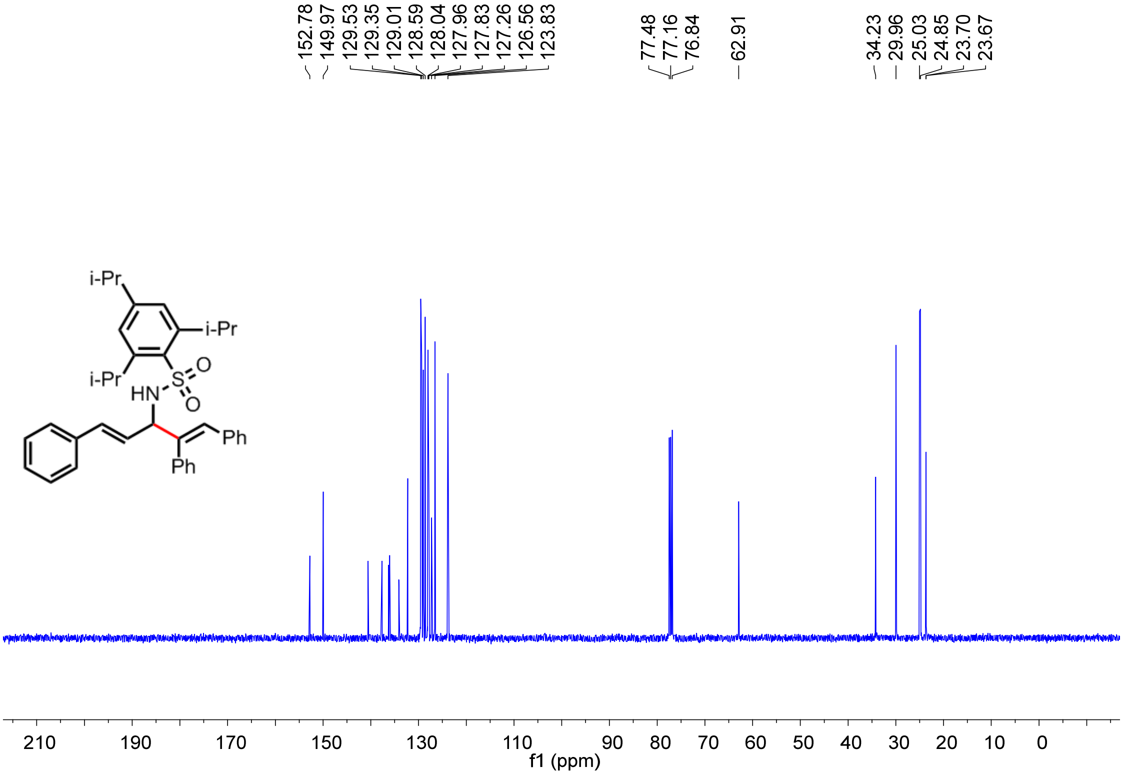

Supplementary Figure 46. ^{1}H and ^{13}C NMR spectra of compound **3q** in $CDCl_3$.

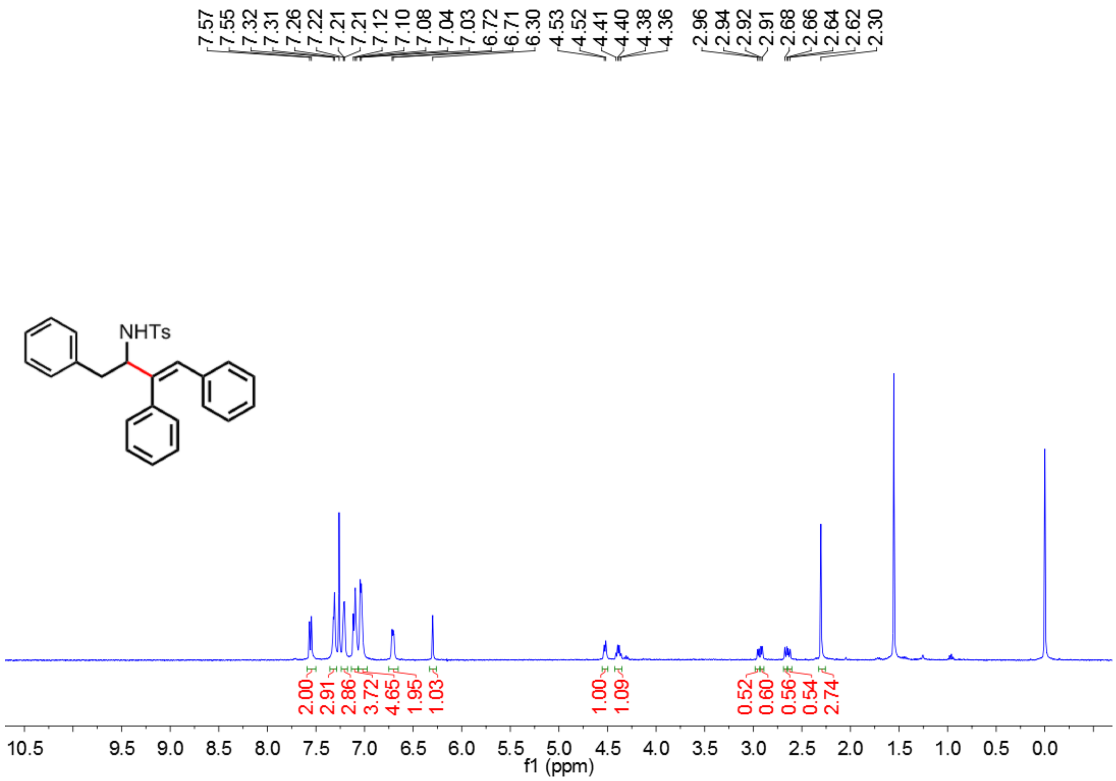

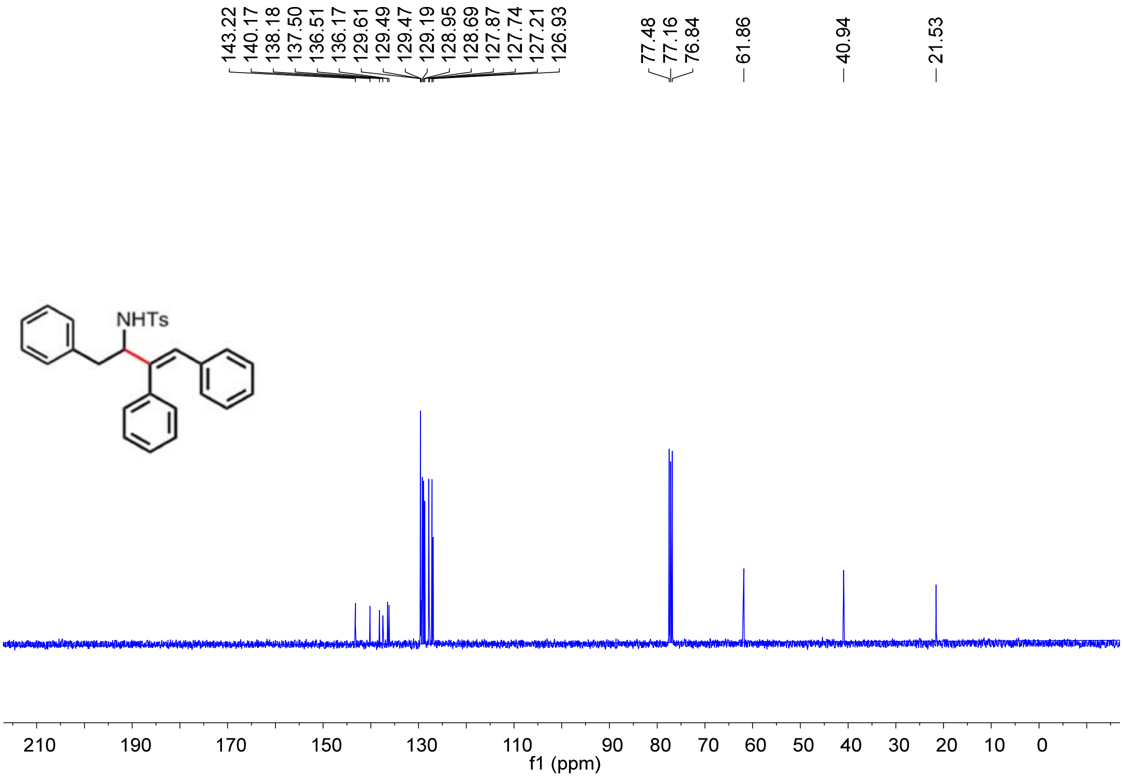

Supplementary Figure 47. ^{1}H and ^{13}C NMR spectra of compound **3r** in $CDCl_3$.

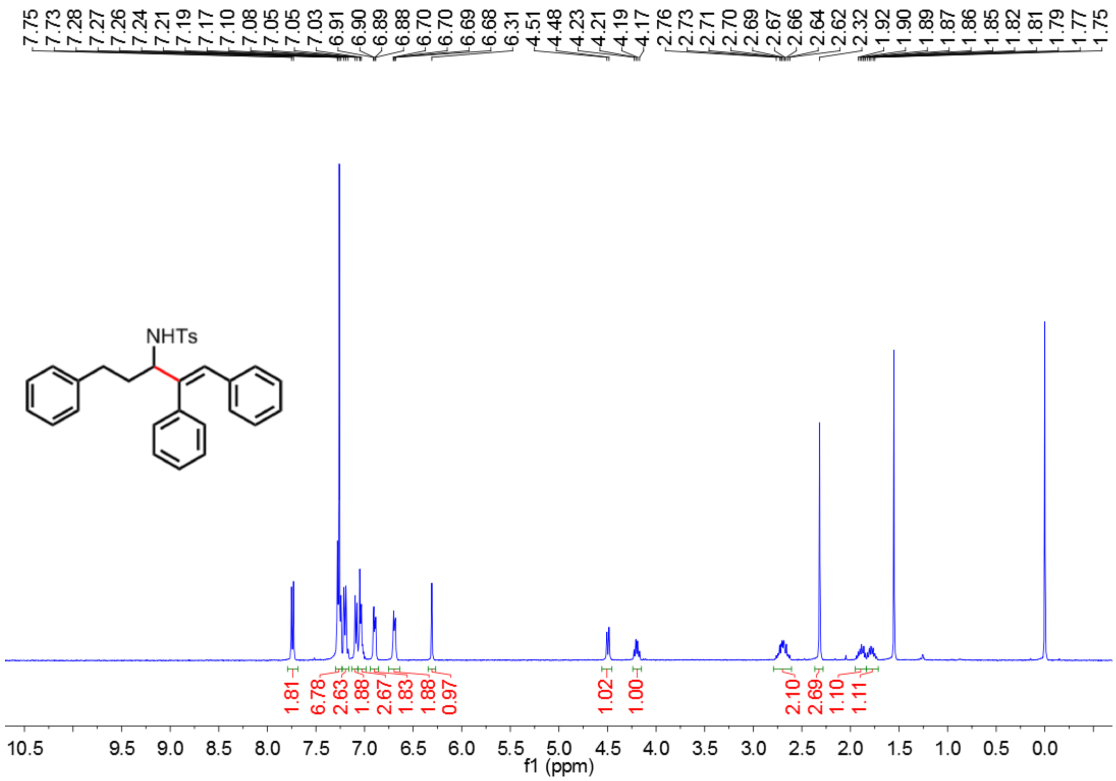

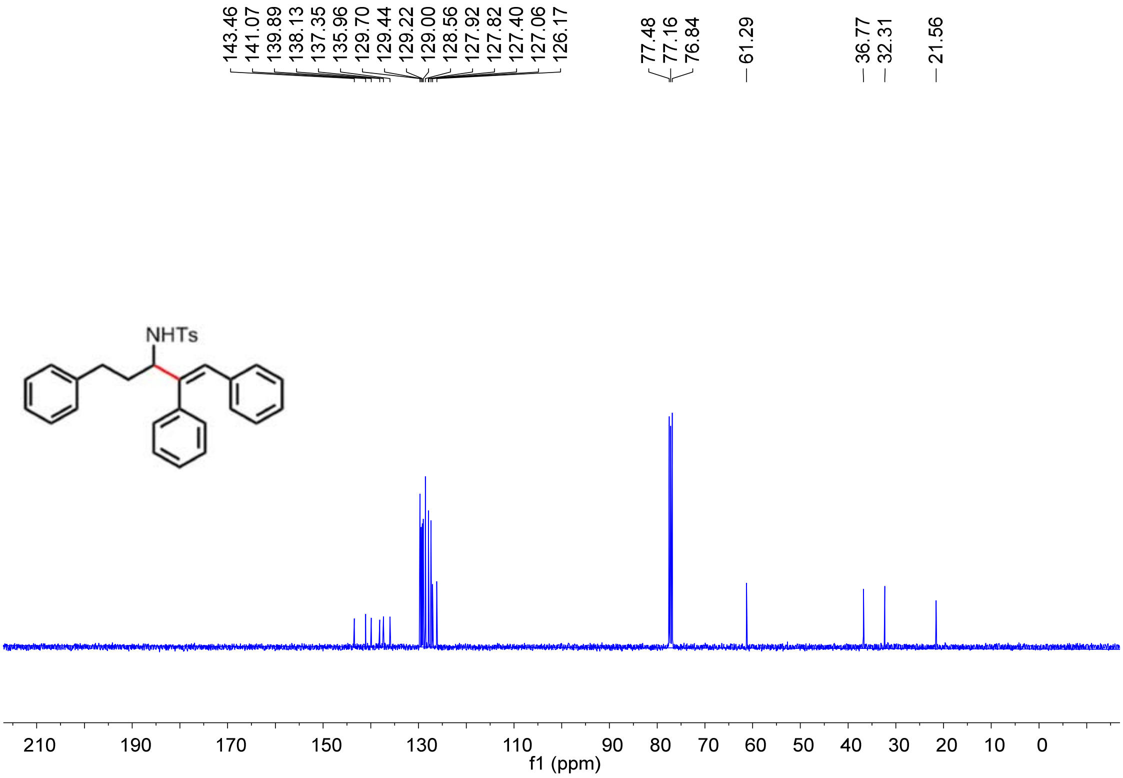

Supplementary Figure 48. ^{1}H and ^{13}C NMR spectra of compound **3s** in $CDCl_3$.

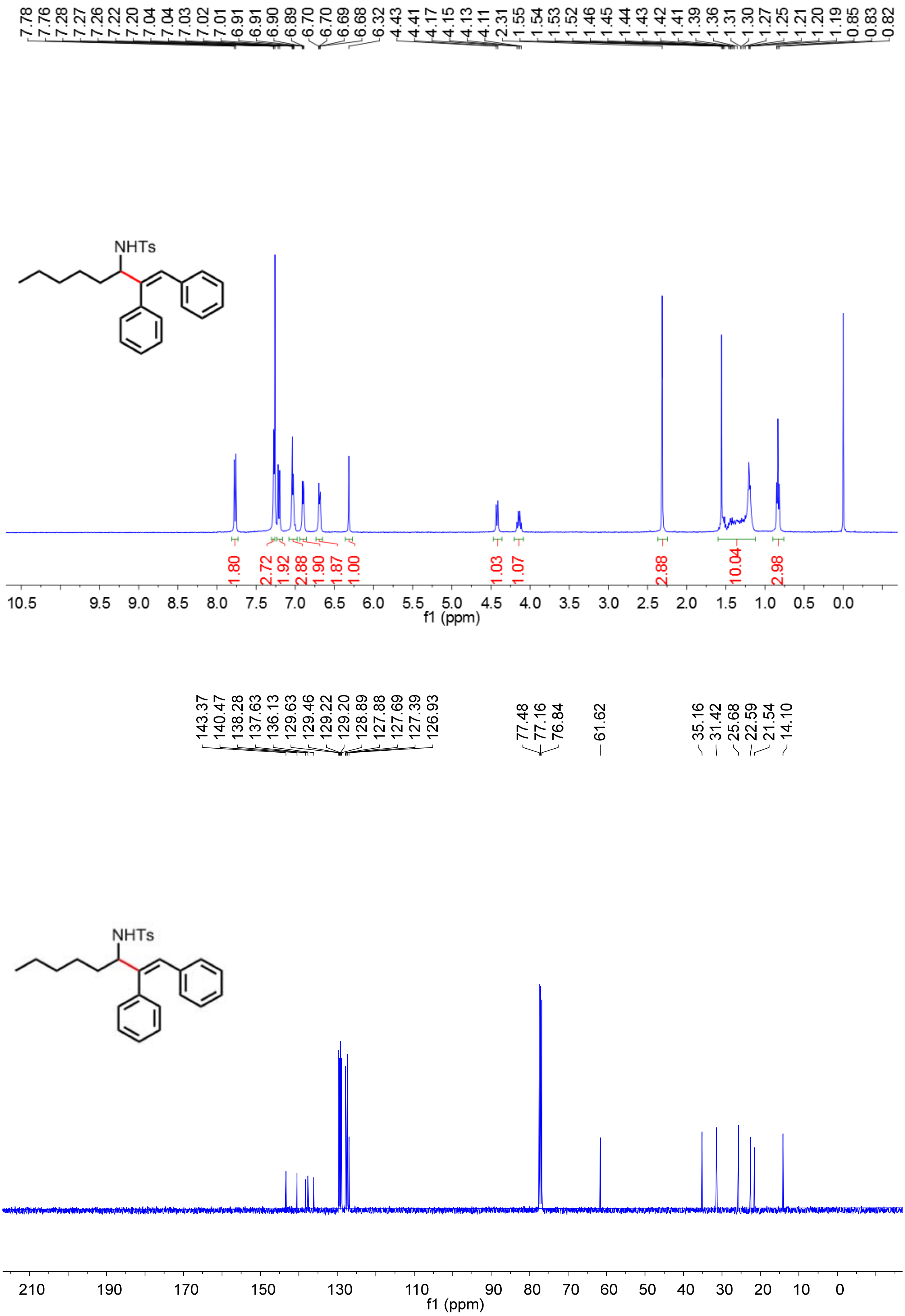

Supplementary Figure 49. ^{1}H and ^{13}C NMR spectra of compound **3t** in $CDCl_3$.

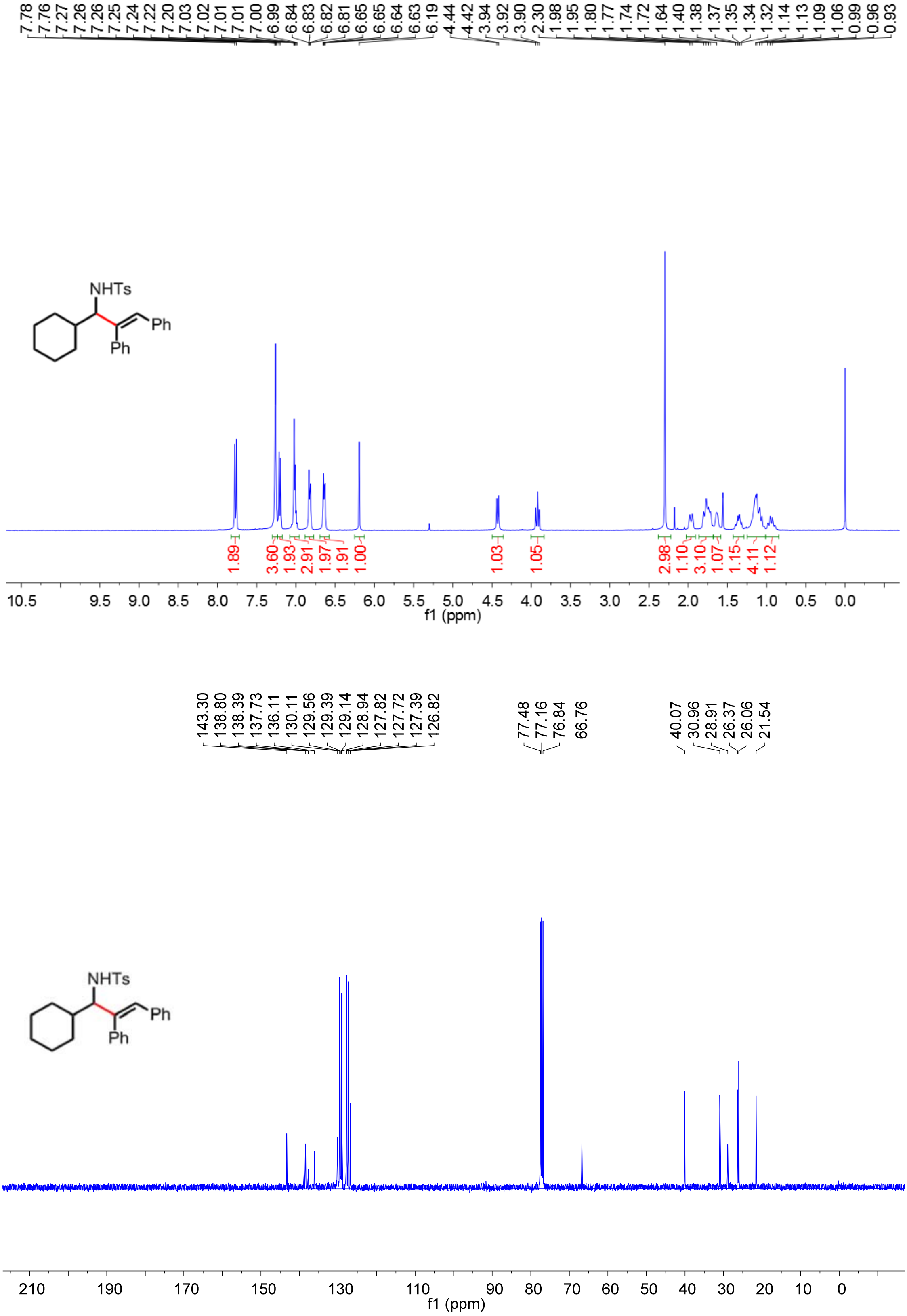

Supplementary Figure 50. ^{1}H and ^{13}C NMR spectra of compound **3u** in $CDCl_3$.

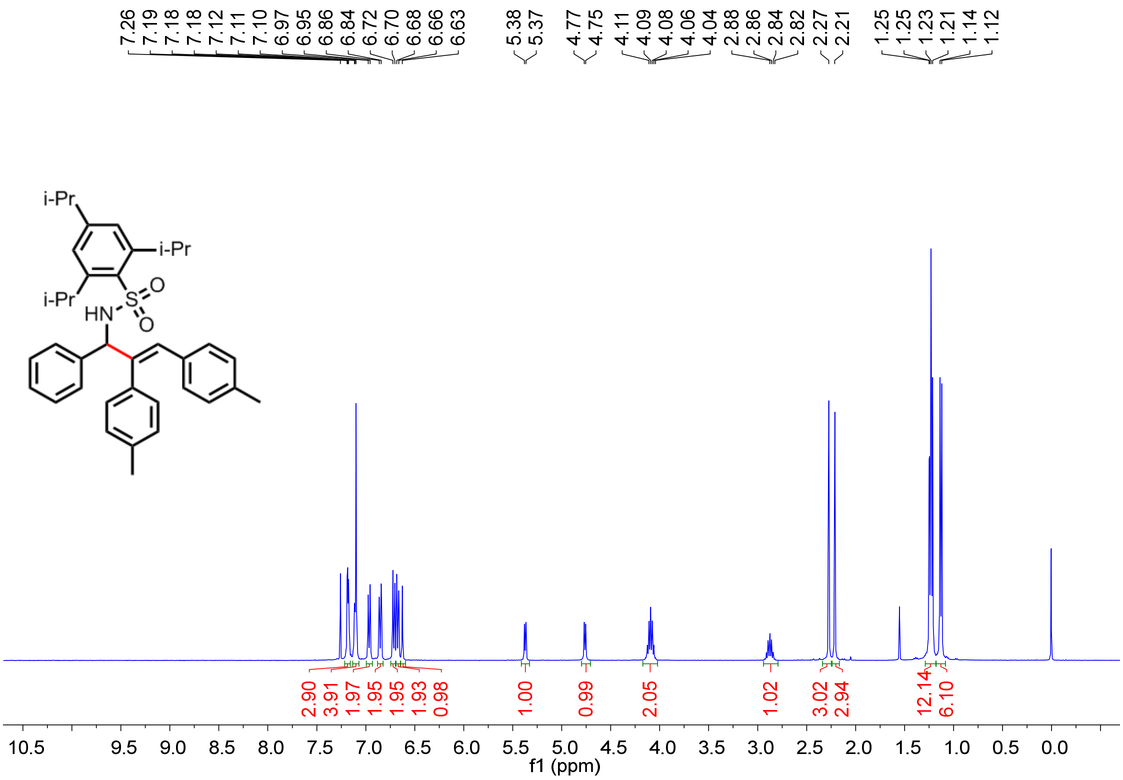

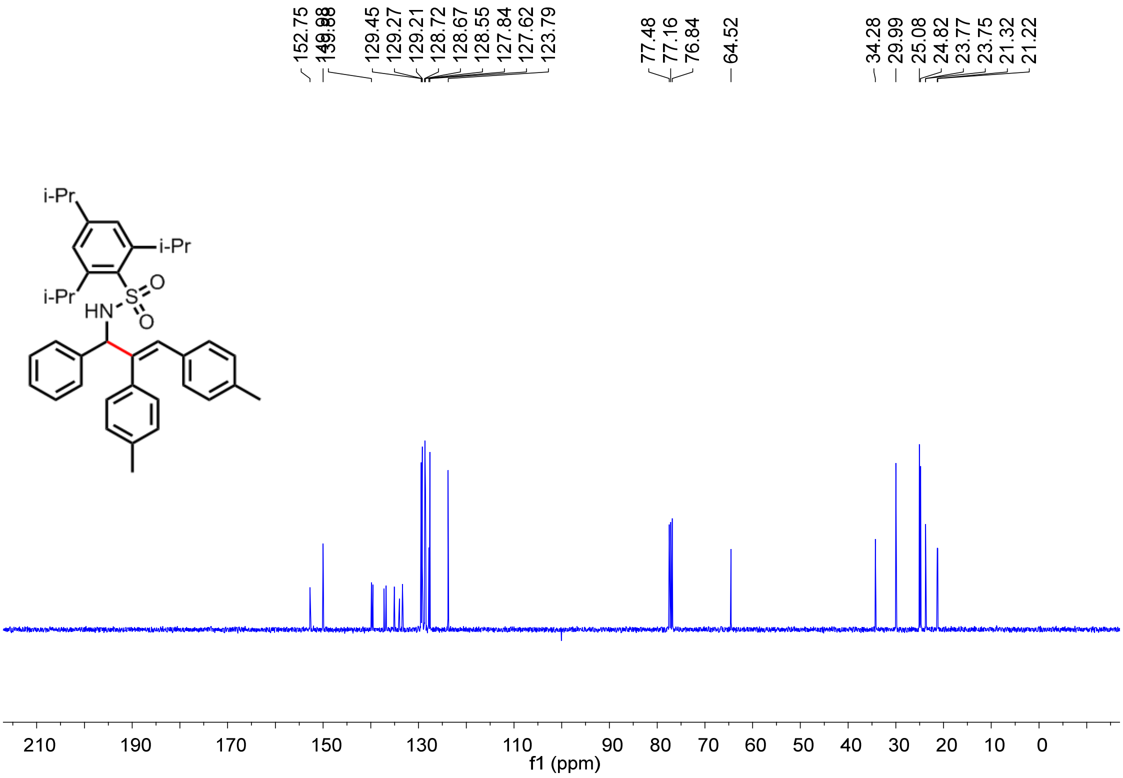

Supplementary Figure 51. ^{1}H and ^{13}C NMR spectra of compound **4a** in $CDCl_3$.

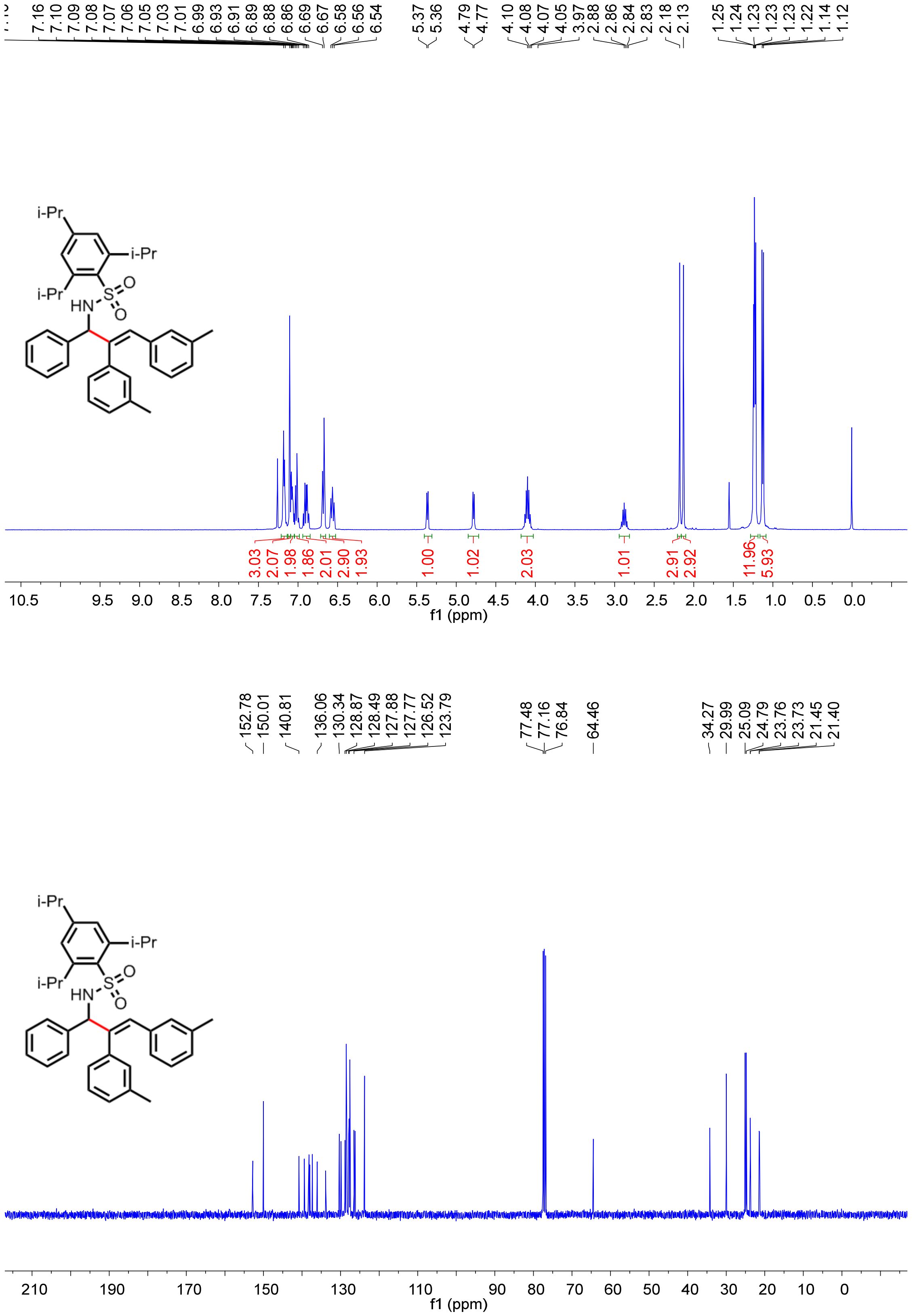

Supplementary Figure 52. ^{1}H and ^{13}C NMR spectra of compound **4b** in $CDCl_3$.

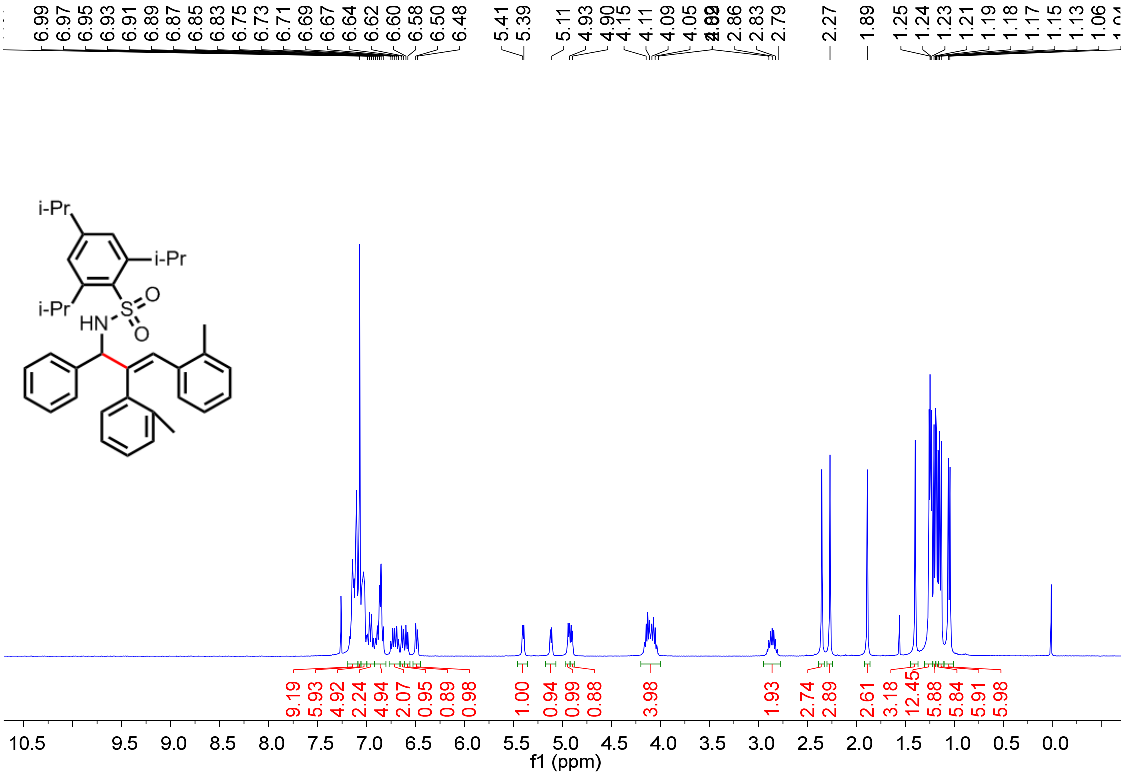

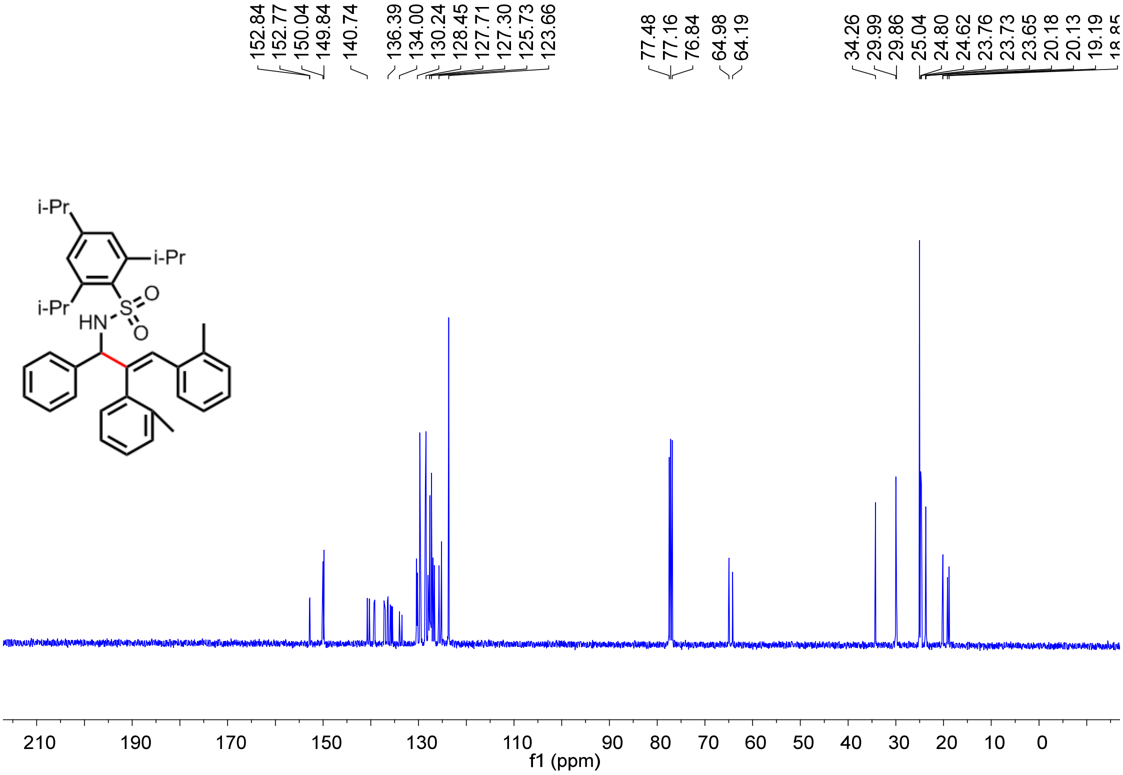

Supplementary Figure 53. ^{1}H and ^{13}C NMR spectra of compound **4c** in $CDCl_3$.

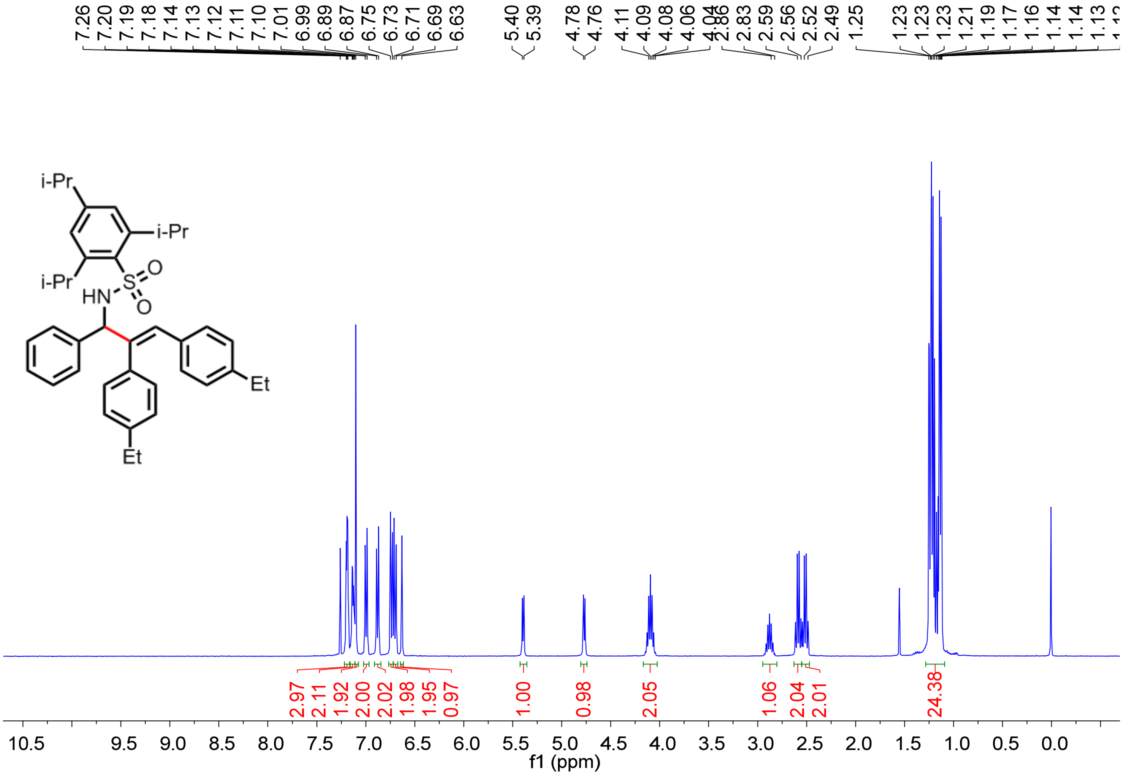

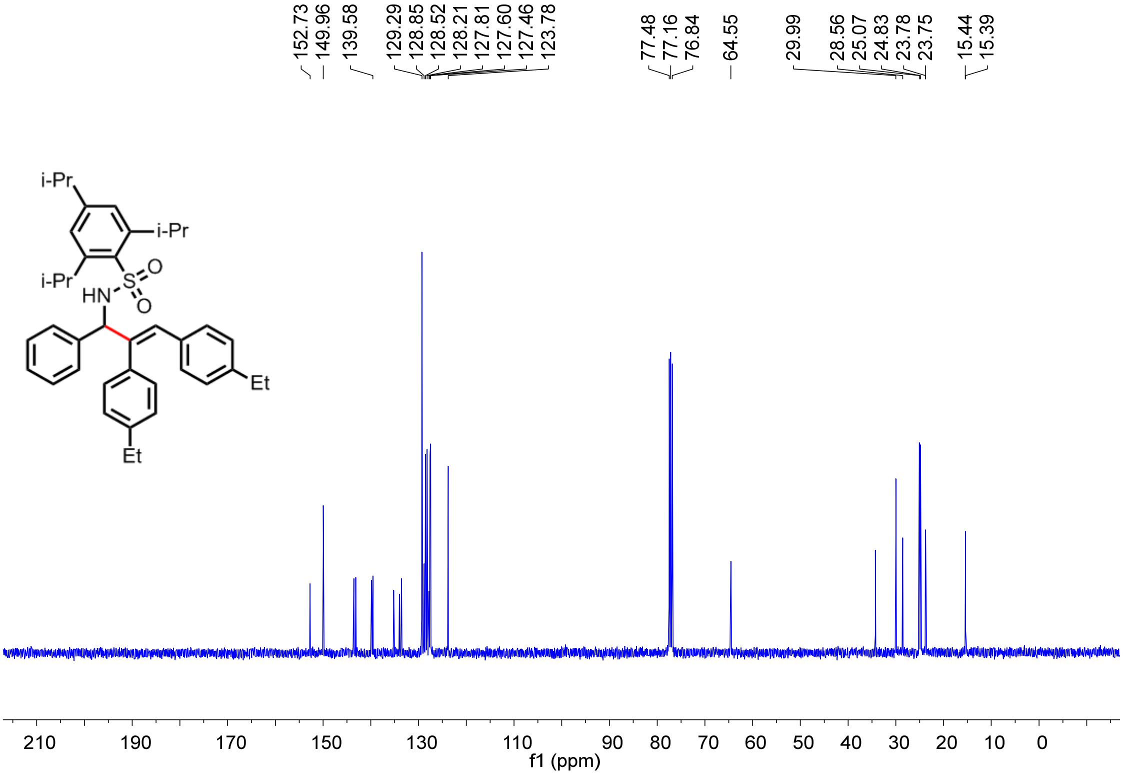

Supplementary Figure 54. ^{1}H and ^{13}C NMR spectra of compound **4d** in $CDCl_3$.

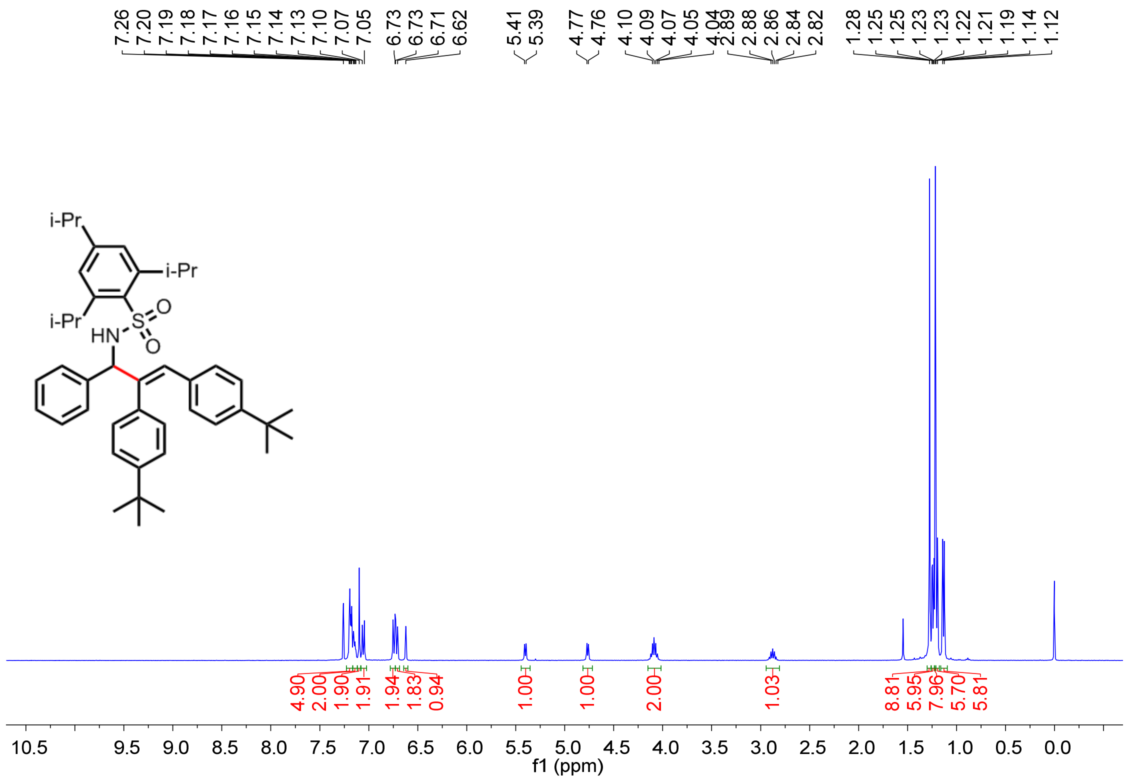

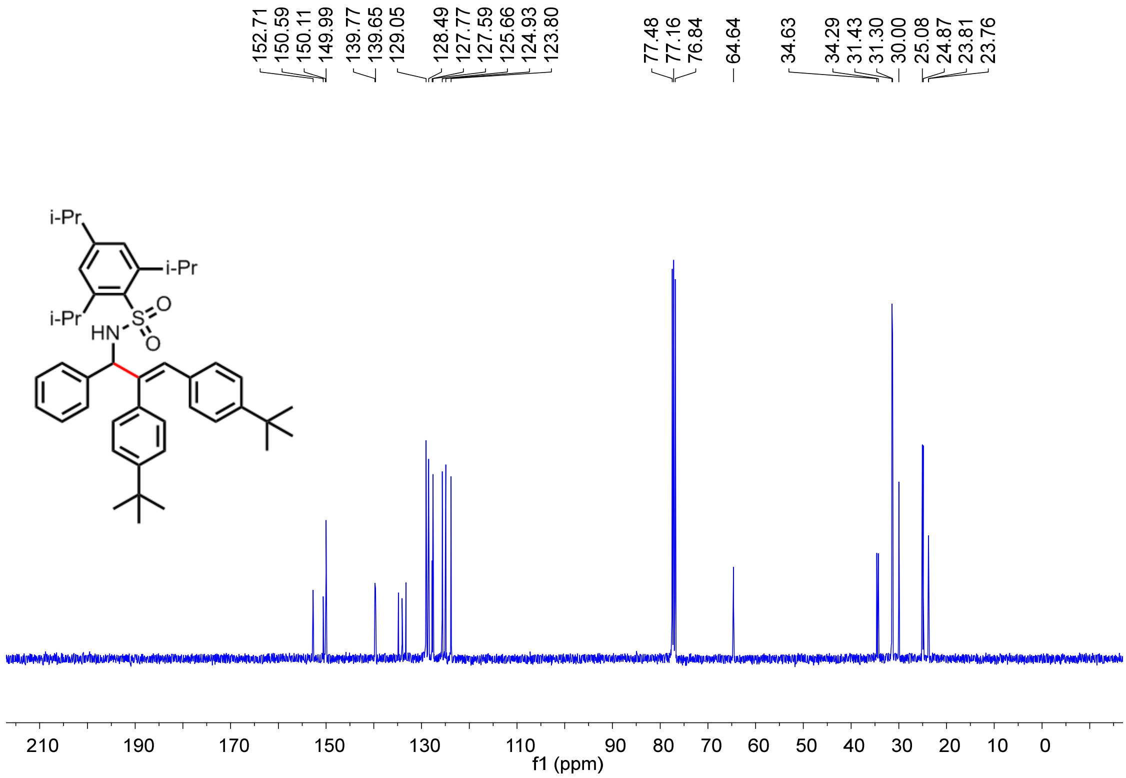

Supplementary Figure 55. ^{1}H and ^{13}C NMR spectra of compound **4e** in $CDCl_3$.

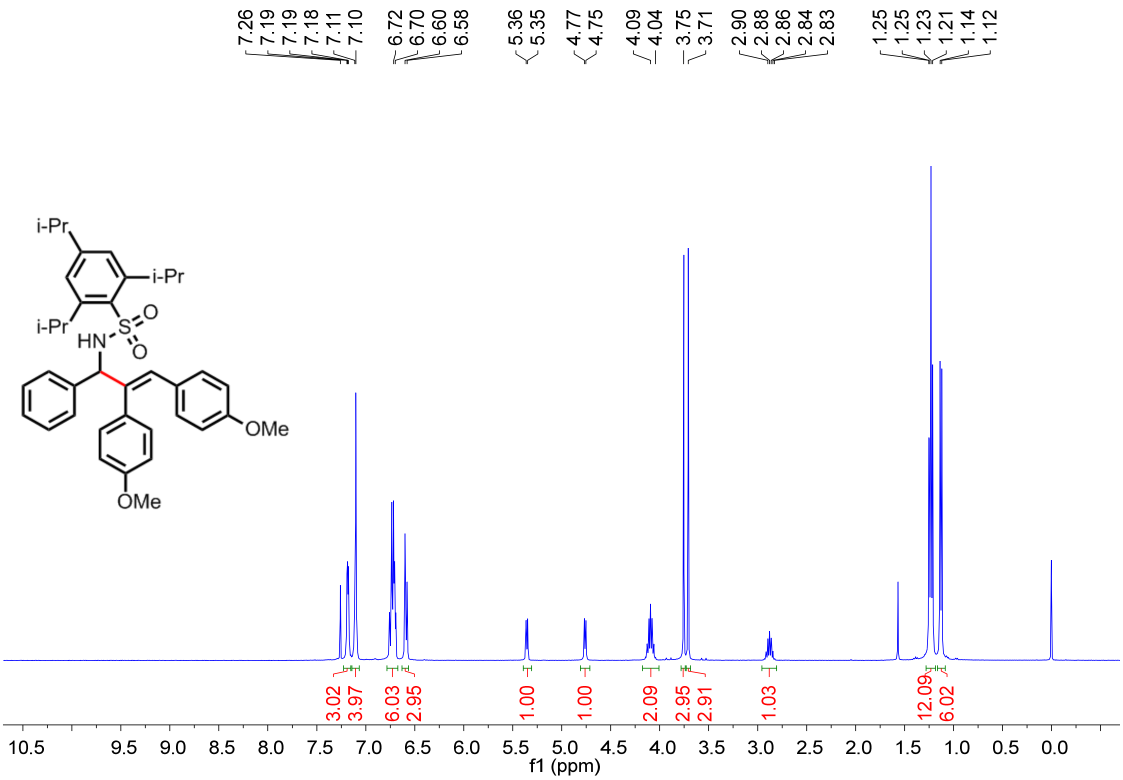

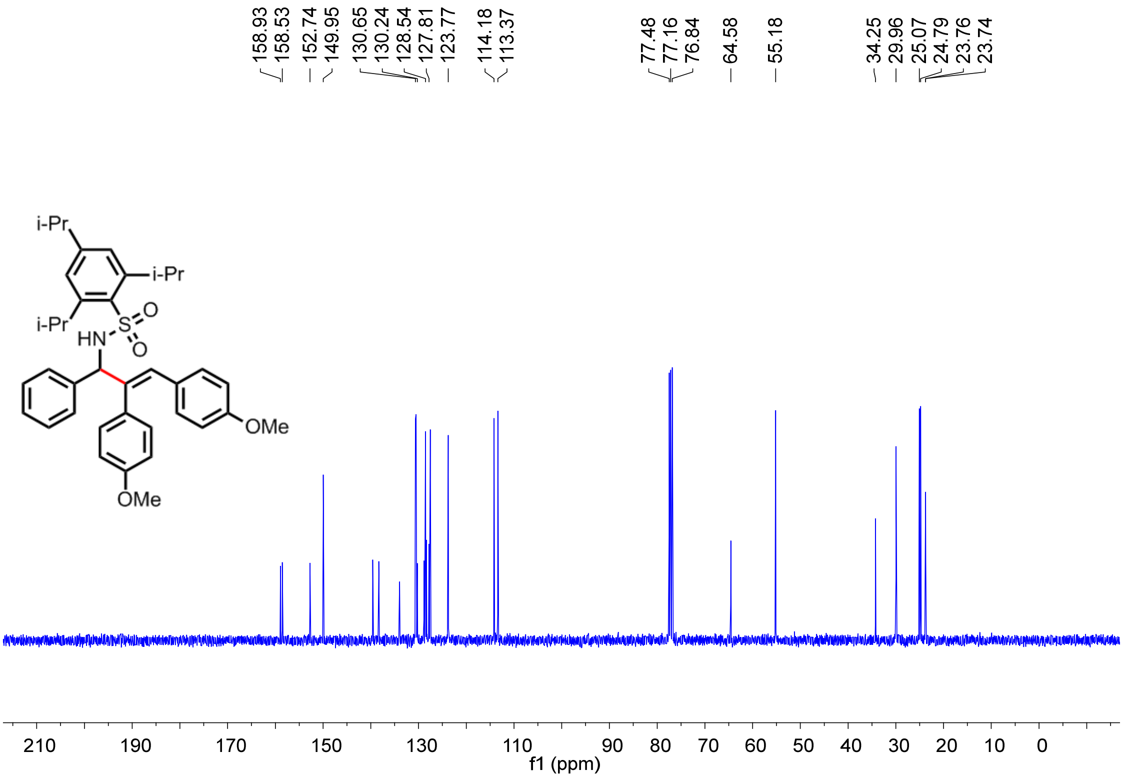

Supplementary Figure 56. ^{1}H and ^{13}C NMR spectra of compound **4f** in $CDCl_3$.

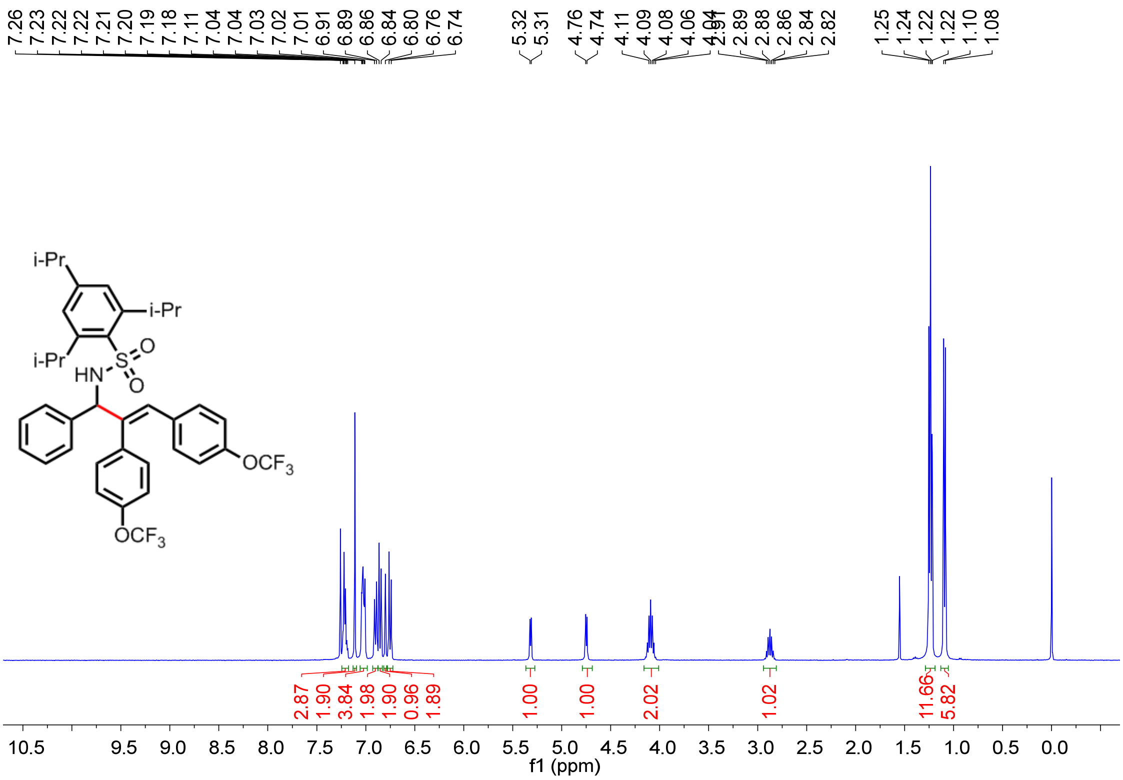

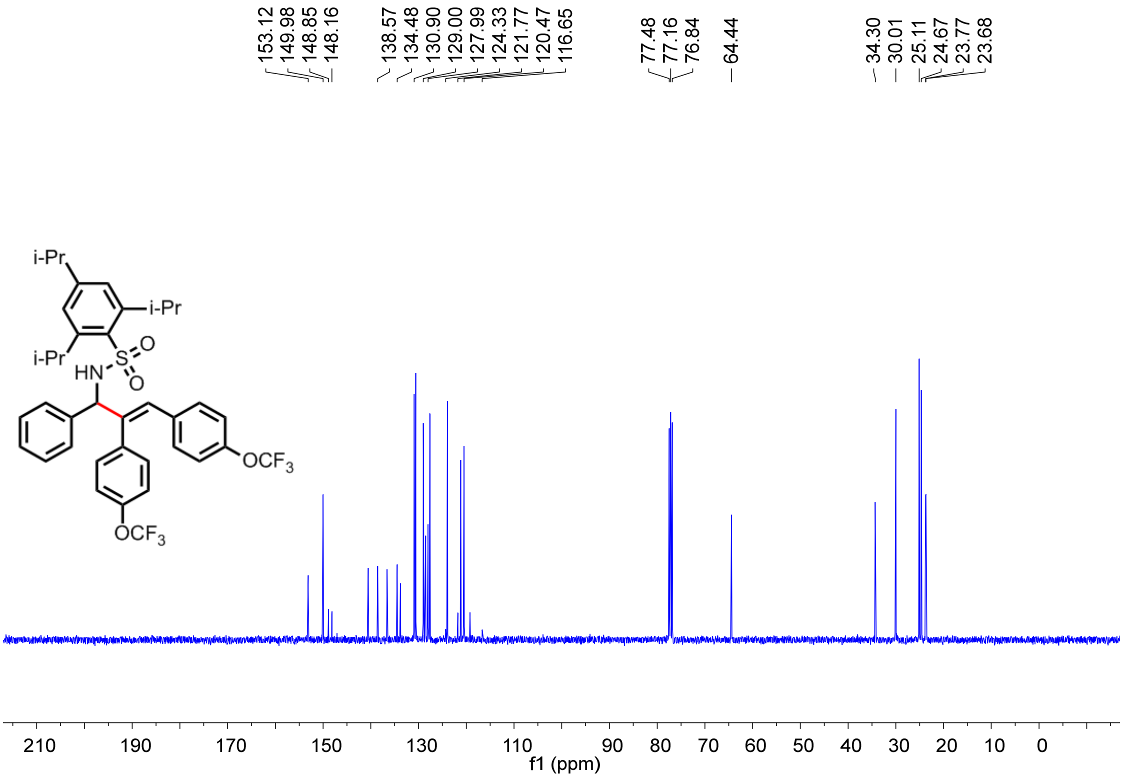

Supplementary Figure 57. ^{1}H and ^{13}C NMR spectra of compound **4g** in $CDCl_3$.

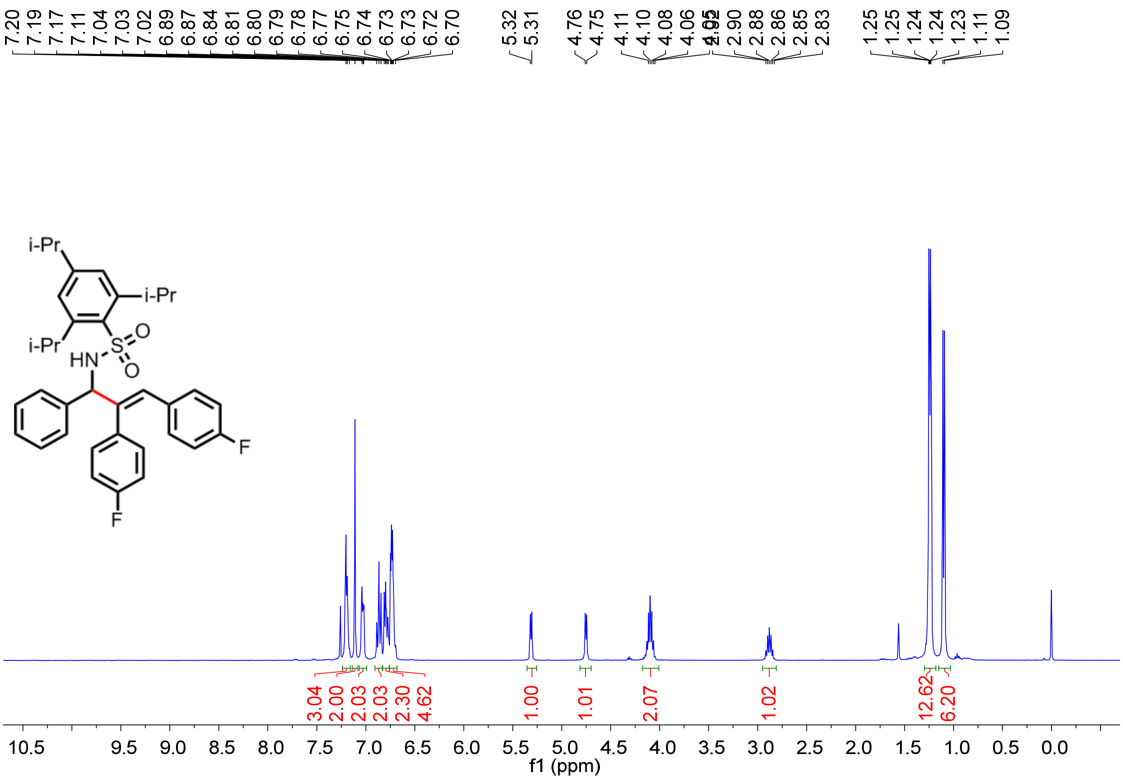

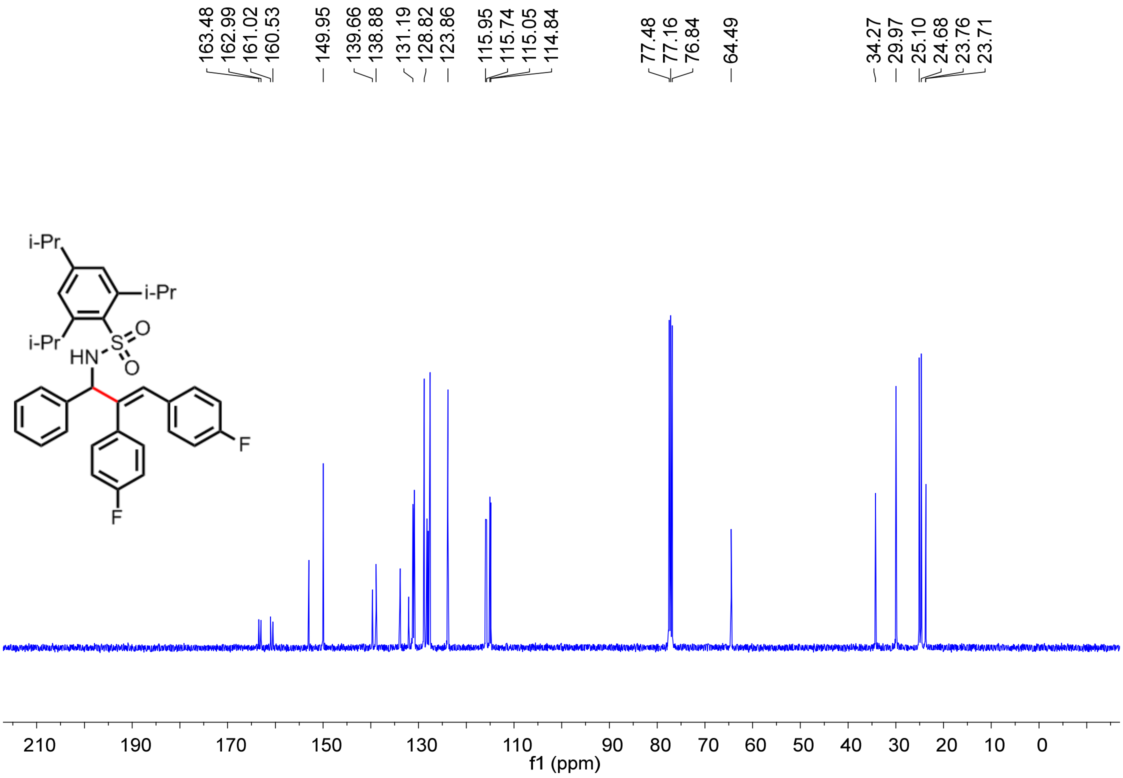

Supplementary Figure 58. ^{1}H and ^{13}C NMR spectra of compound **4h** in $CDCl_3$.

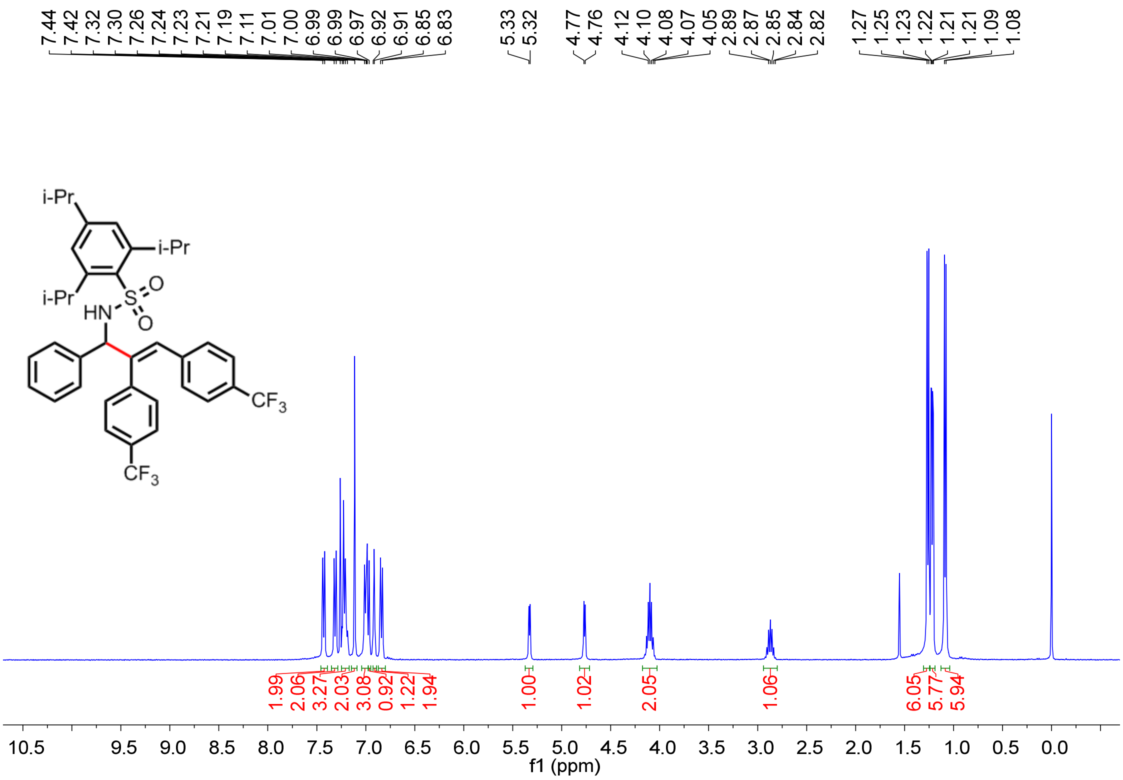

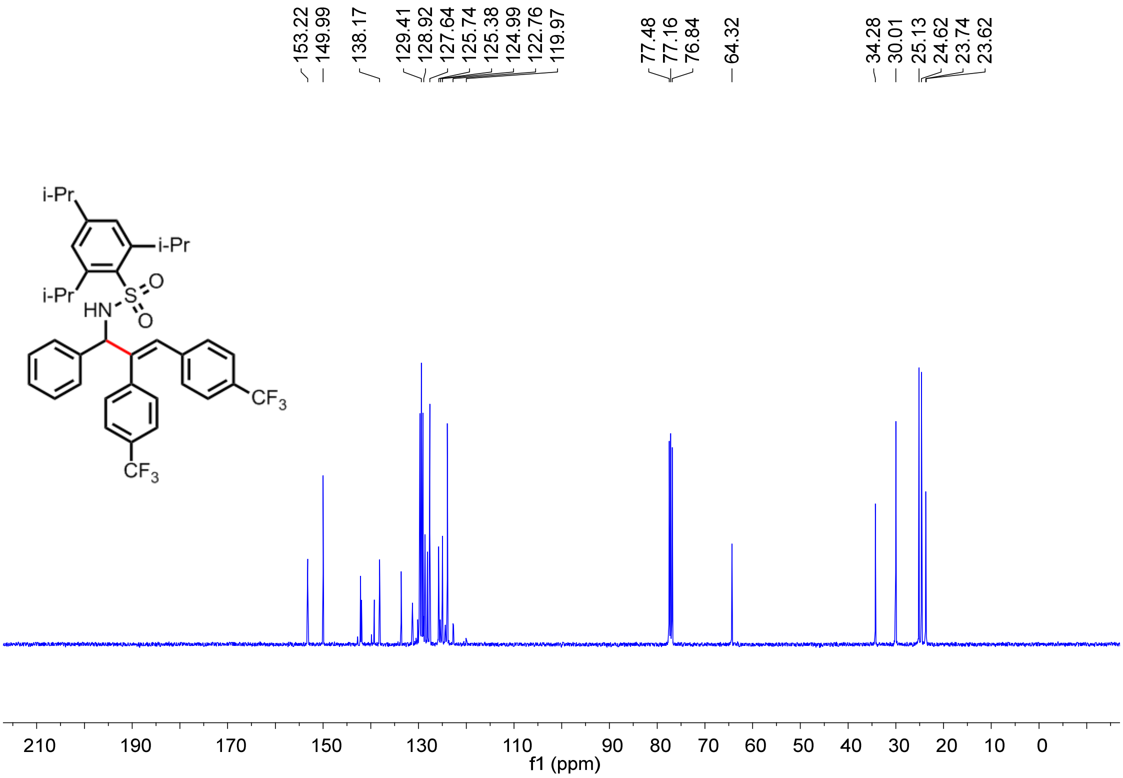

Supplementary Figure 59. ^{1}H and ^{13}C NMR spectra of compound **4i** in $CDCl_3$.

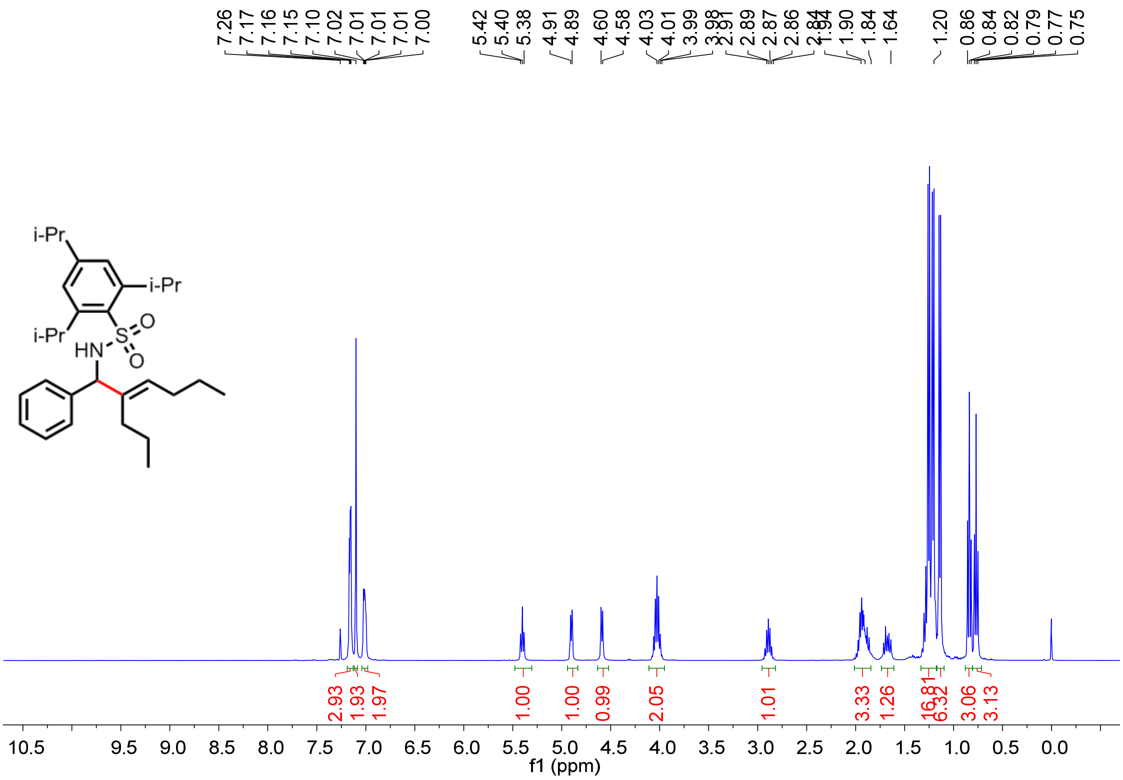

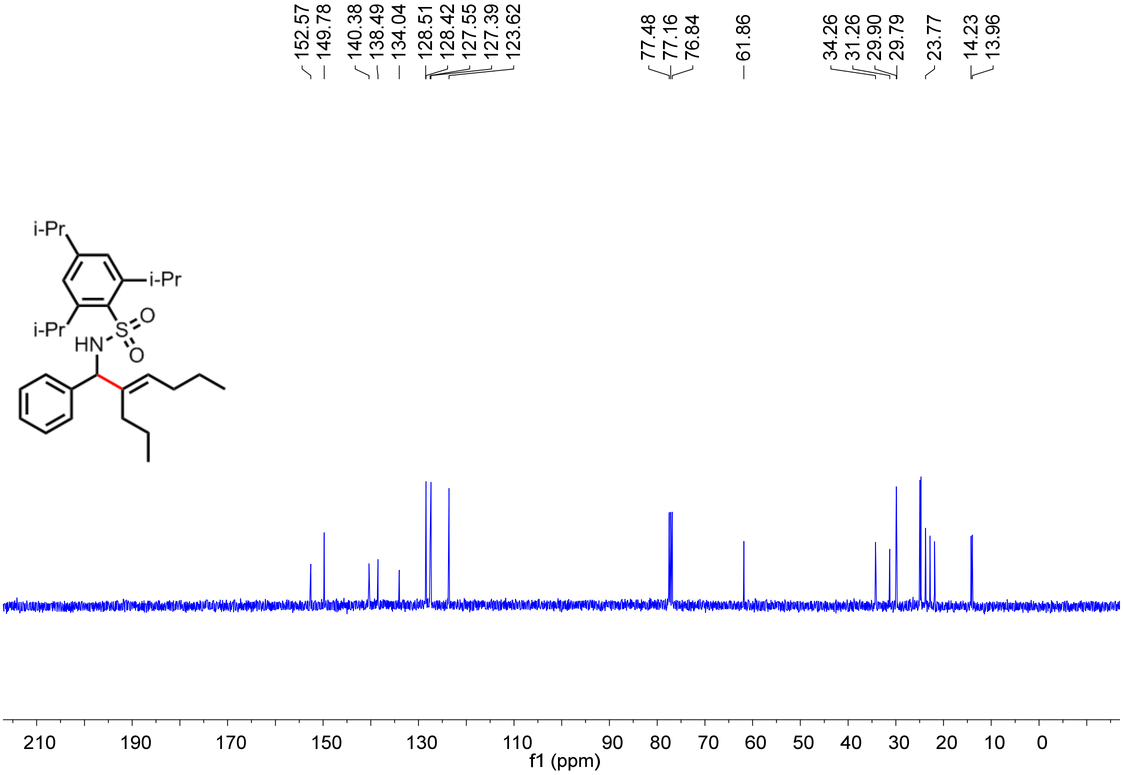

Supplementary Figure 60. ^{1}H and ^{13}C NMR spectra of compound **4j** in $CDCl_3$.

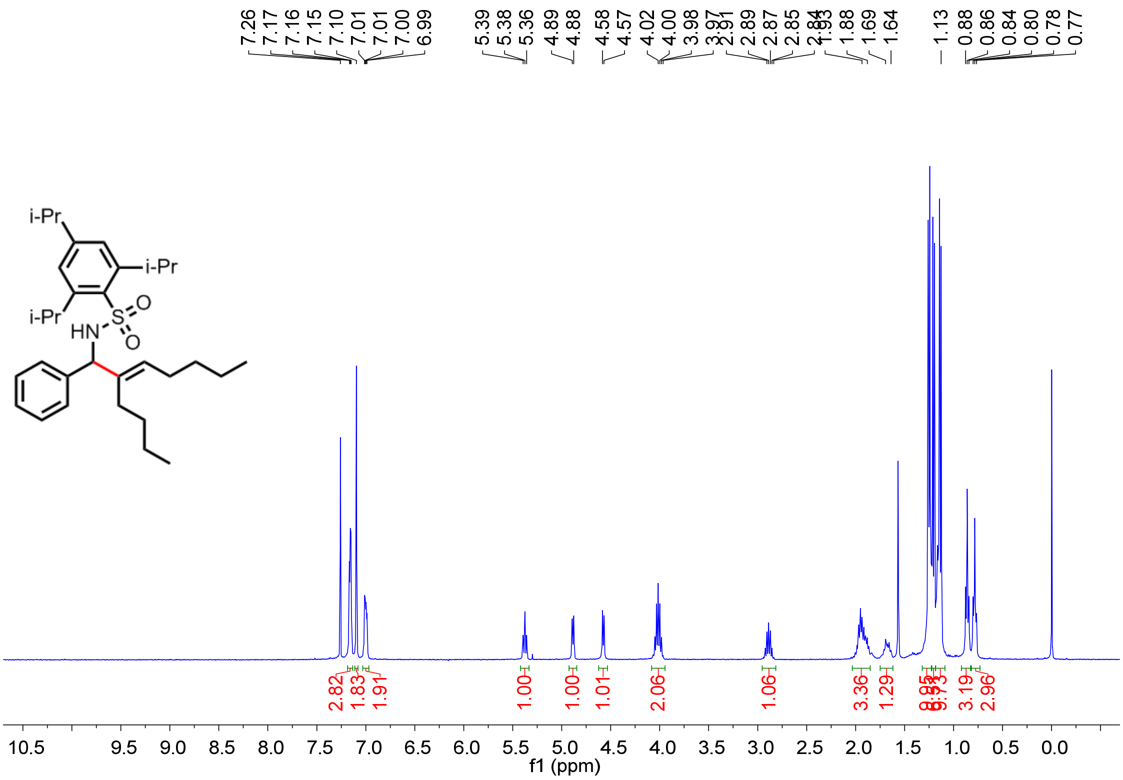

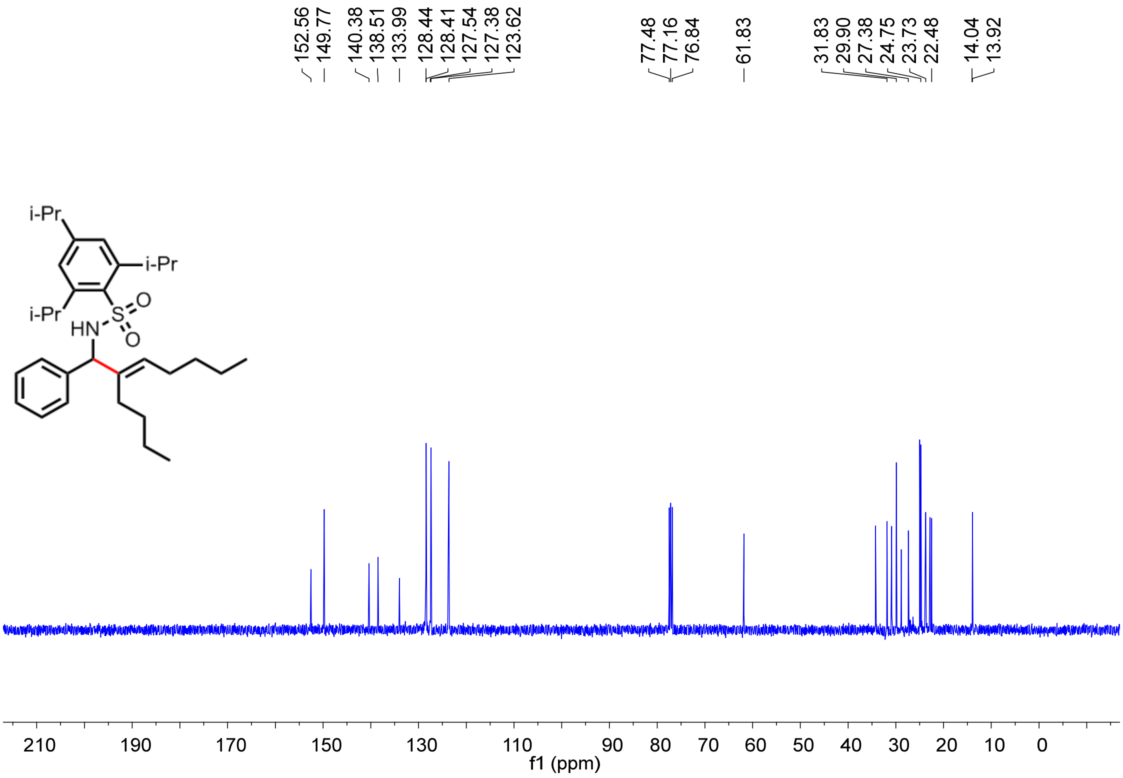

Supplementary Figure 61. ^{1}H and ^{13}C NMR spectra of compound **4k** in $CDCl_3$.

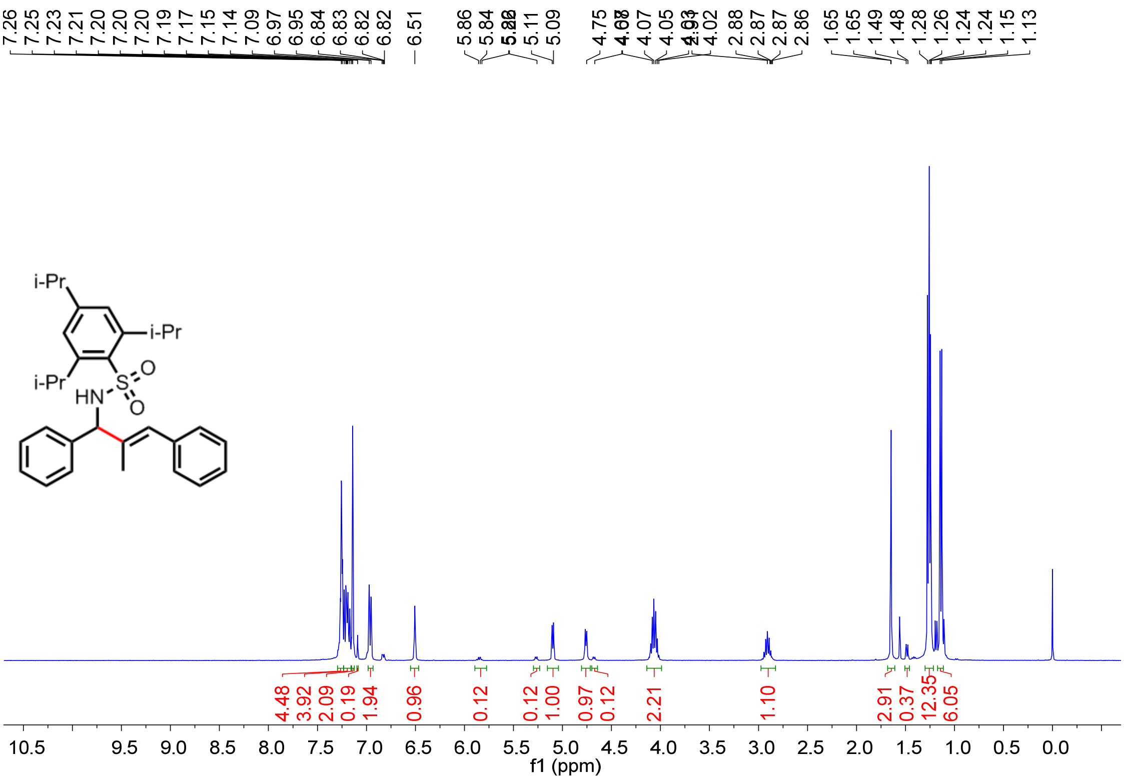

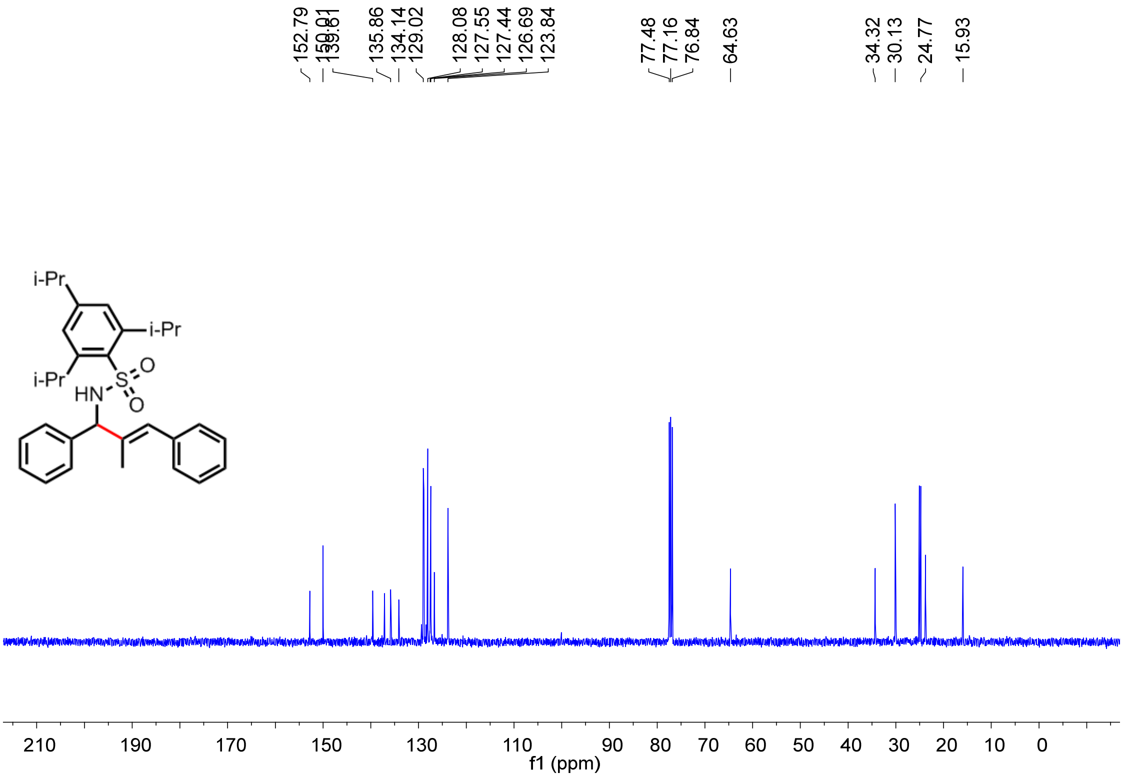

Supplementary Figure 62. ^{1}H and ^{13}C NMR spectra of compound **4l** in $CDCl_3$.

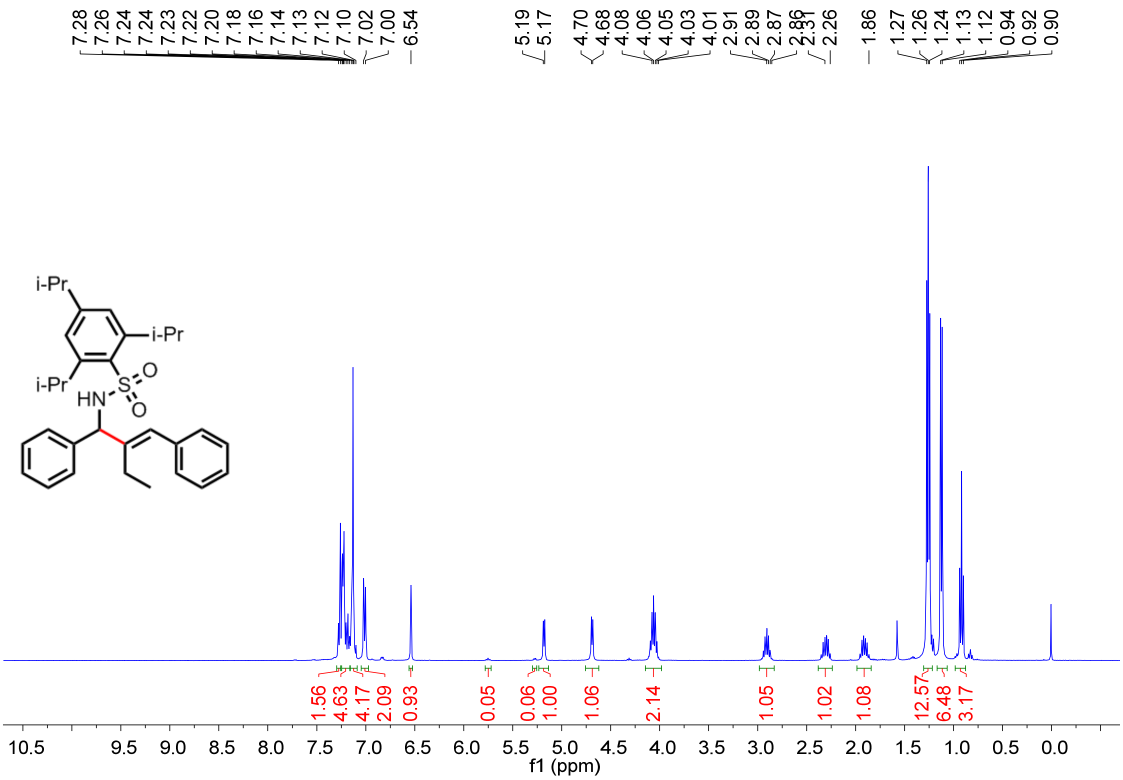

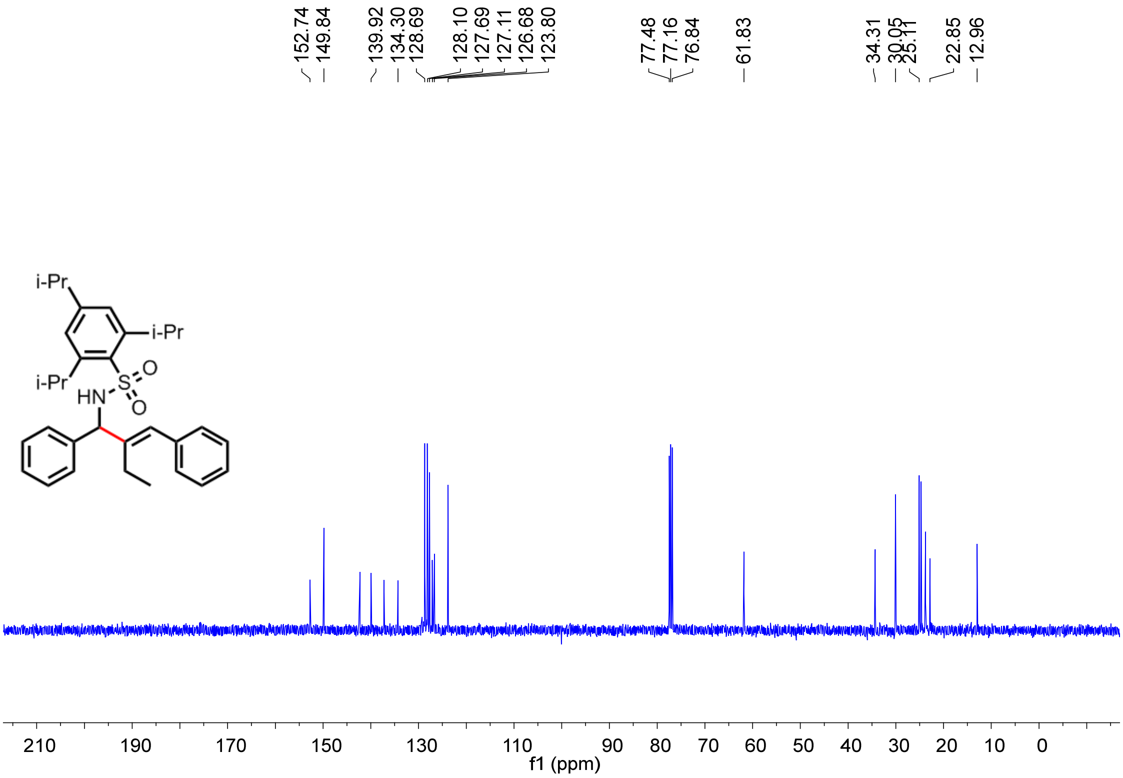

Supplementary Figure 63. ^{1}H and ^{13}C NMR spectra of compound **4m** in $CDCl_3$.

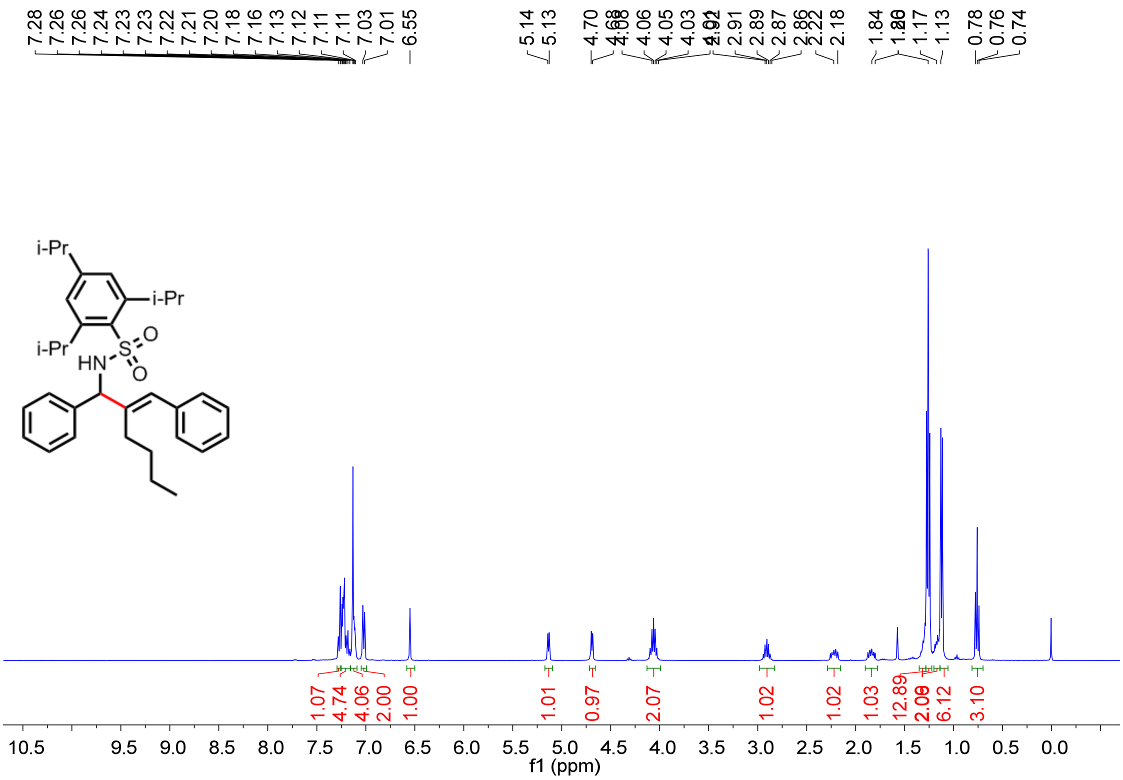

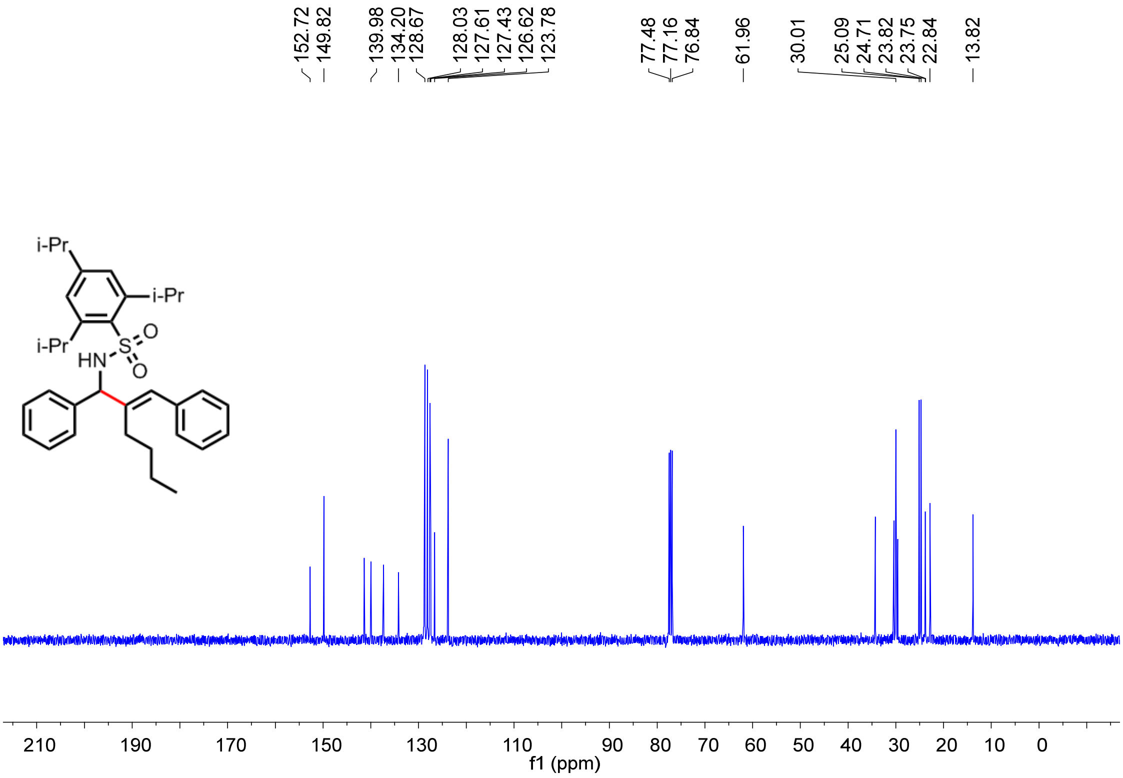

Supplementary Figure 64. ^{1}H and ^{13}C NMR spectra of compound **4n** in $CDCl_3$.

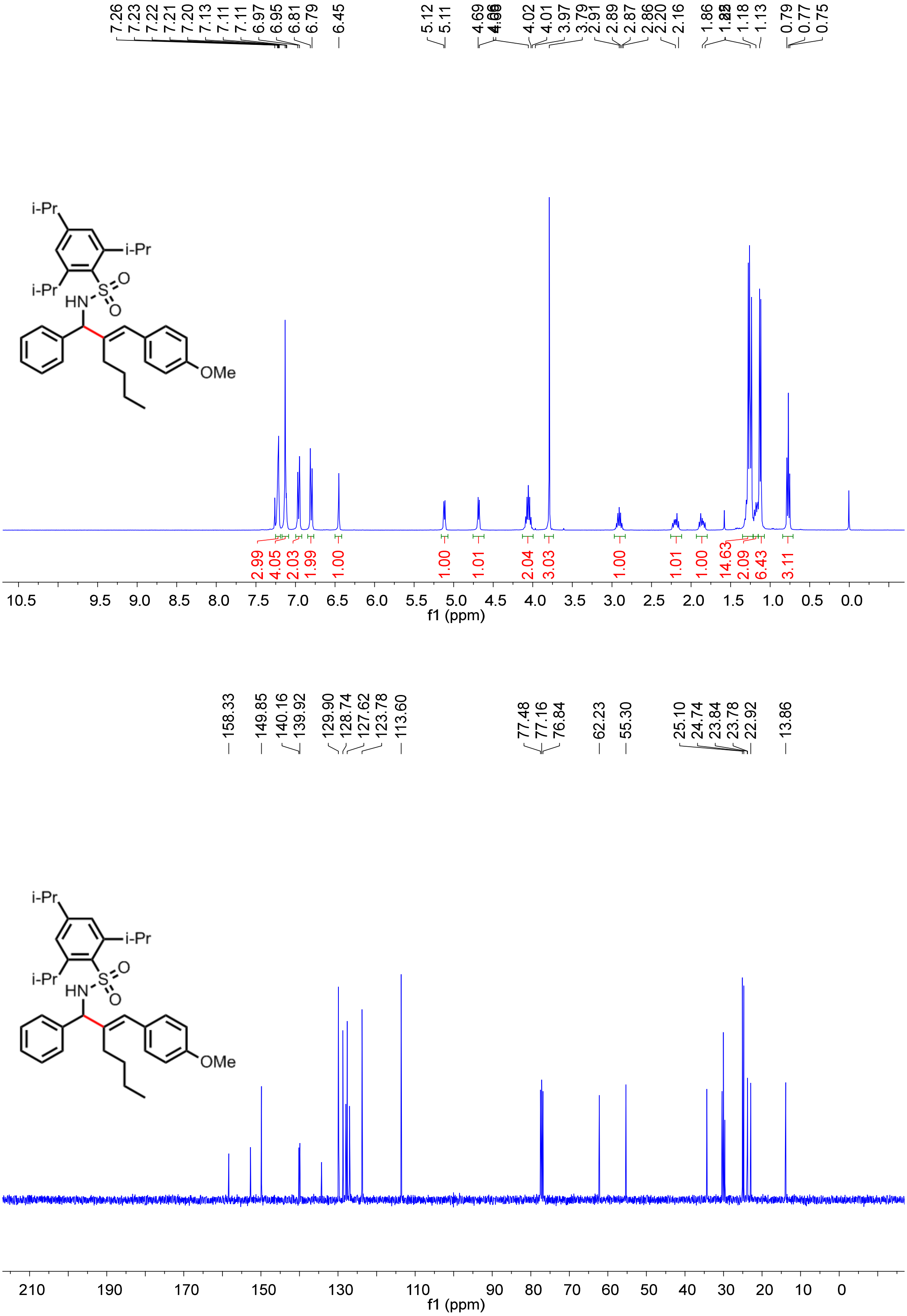

Supplementary Figure 65. ^{1}H and ^{13}C NMR spectra of compound **4o** in $CDCl_3$.

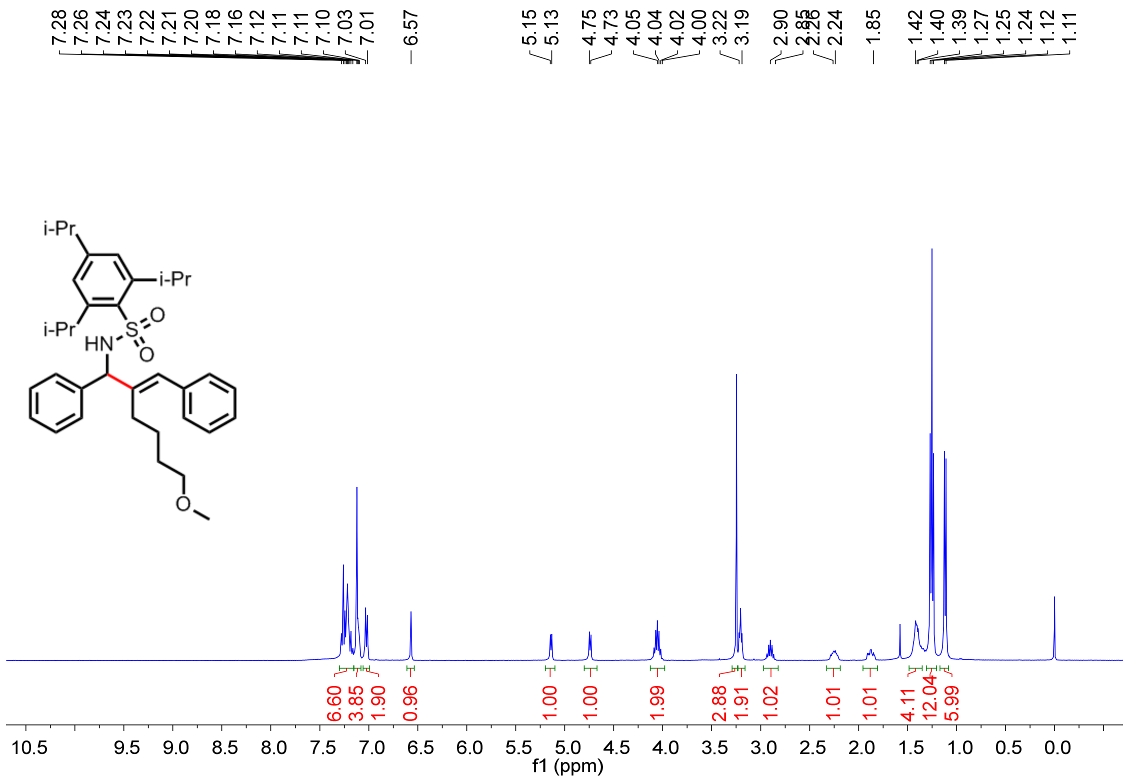

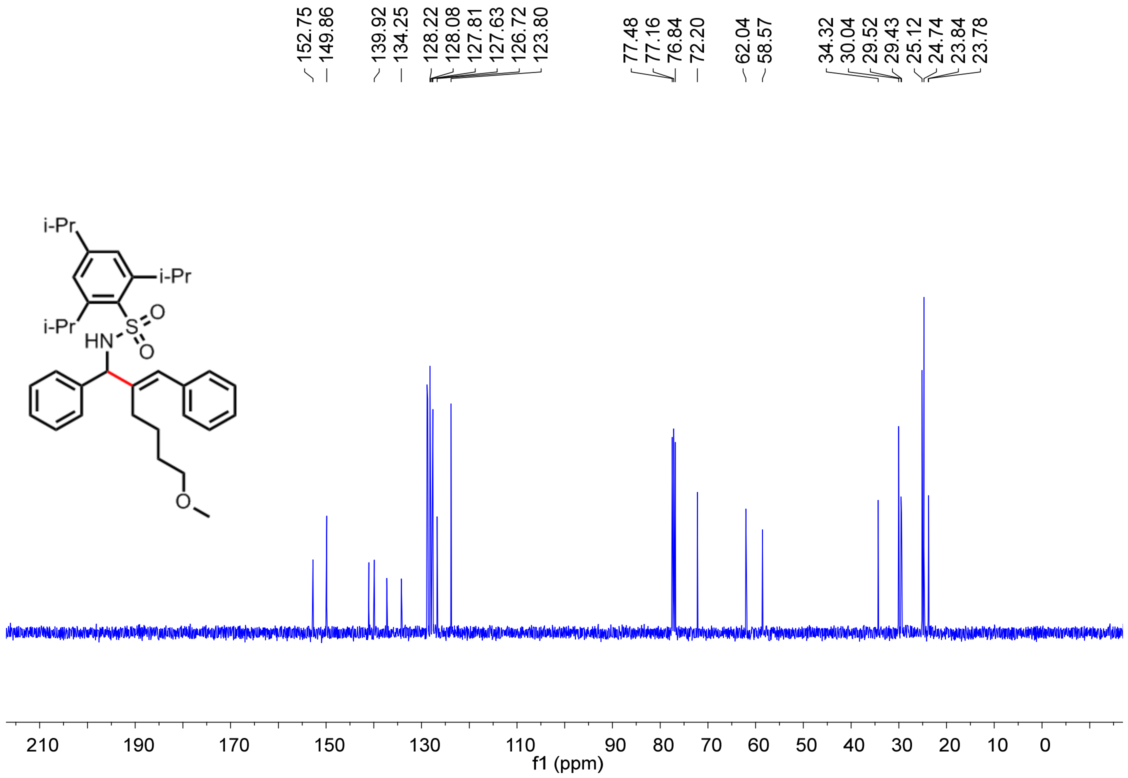

Supplementary Figure 66. ^{1}H and ^{13}C NMR spectra of compound **4p** in $CDCl_3$.

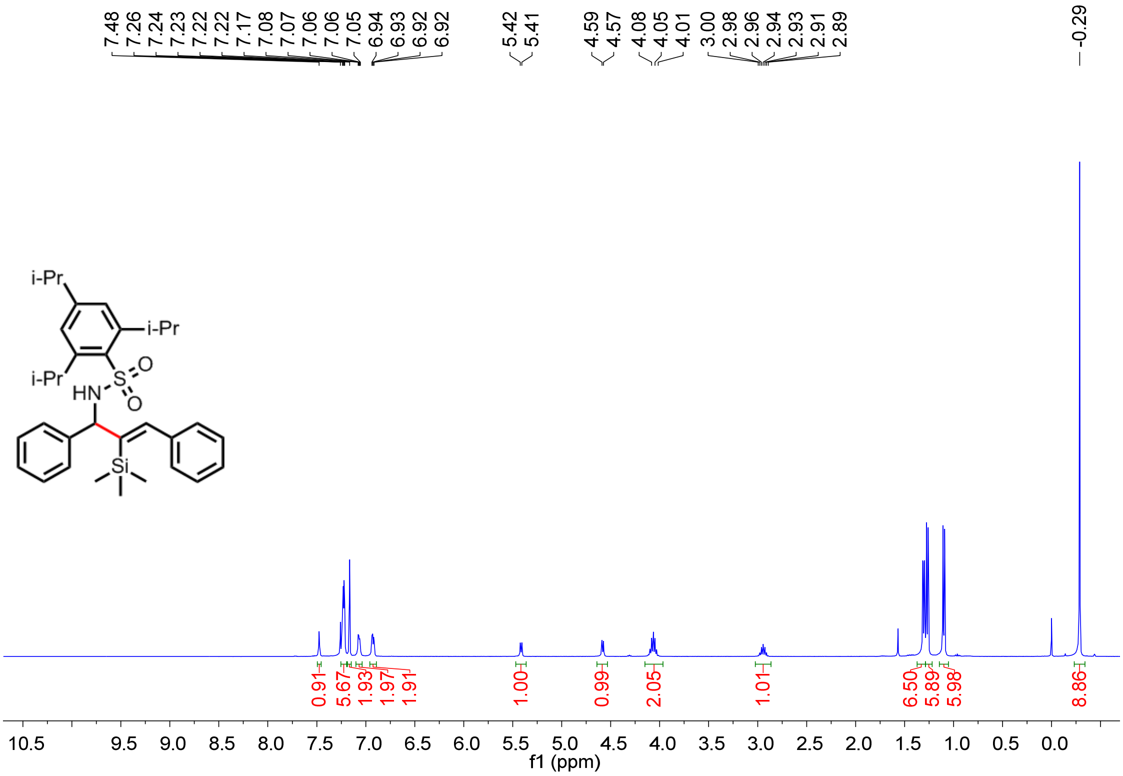

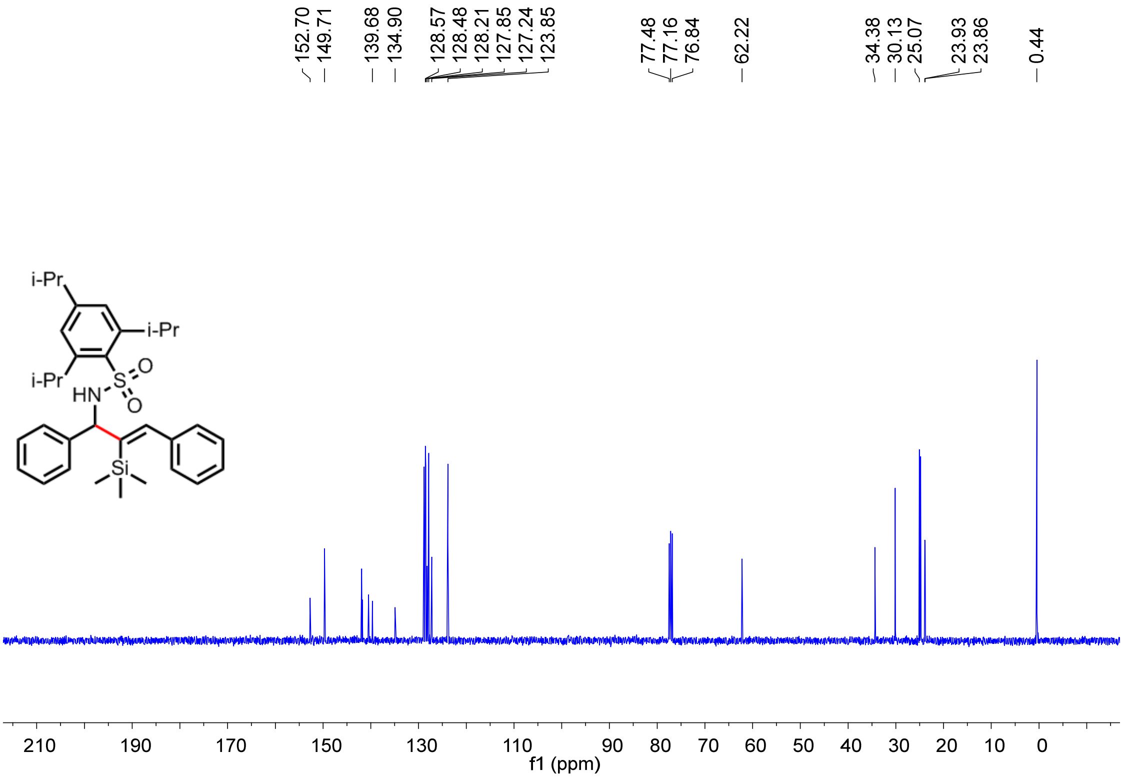

Supplementary Figure 67. ^{1}H and ^{13}C NMR spectra of compound **4q** in $CDCl_3$.

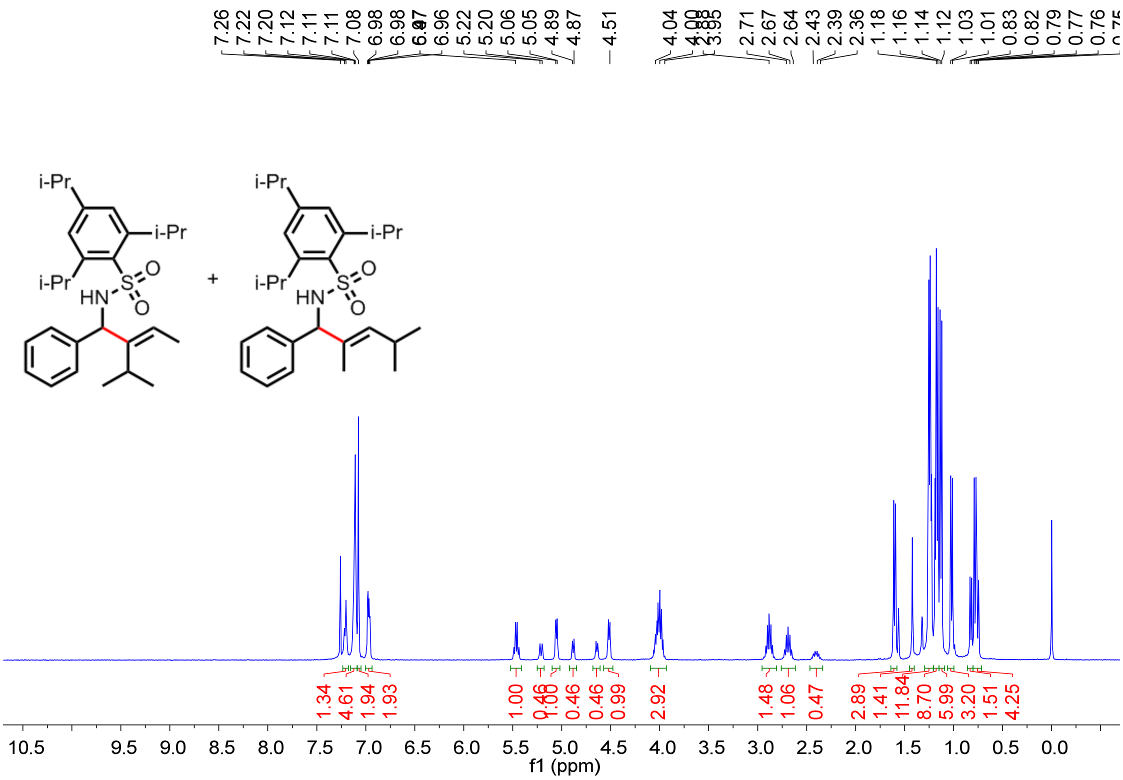

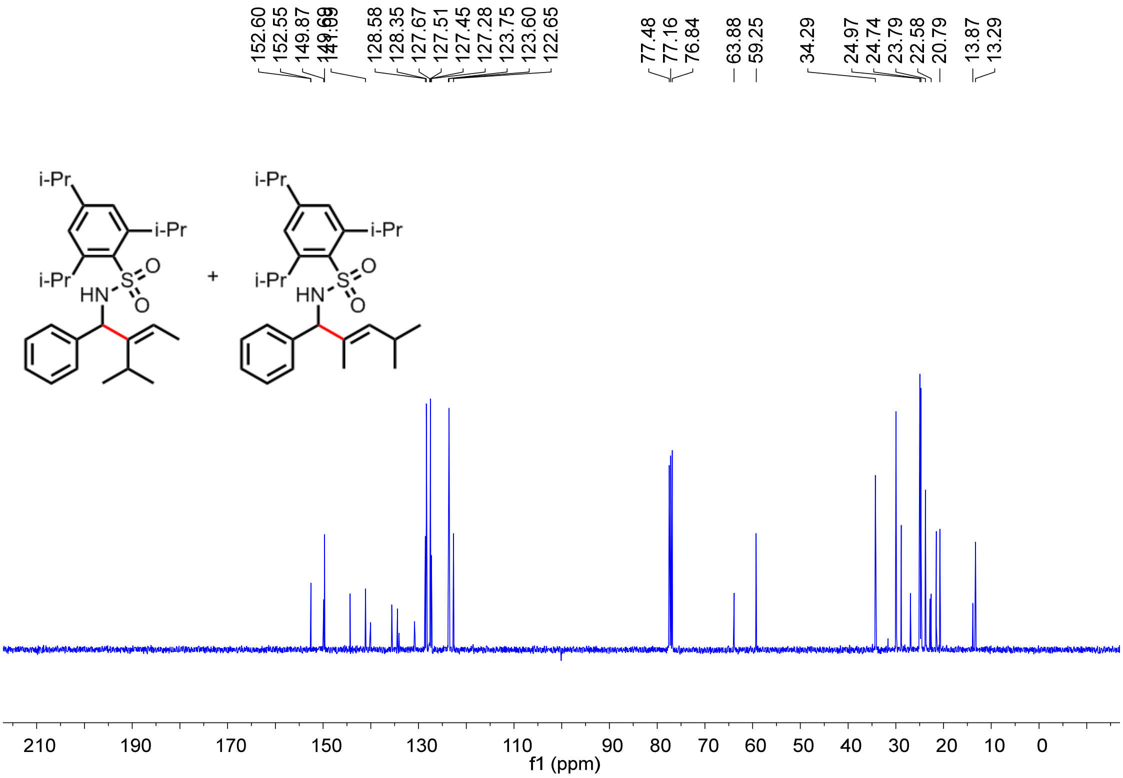

Supplementary Figure 68. ^{1}H and ^{13}C NMR spectra of compound **4r** in $CDCl_3$.

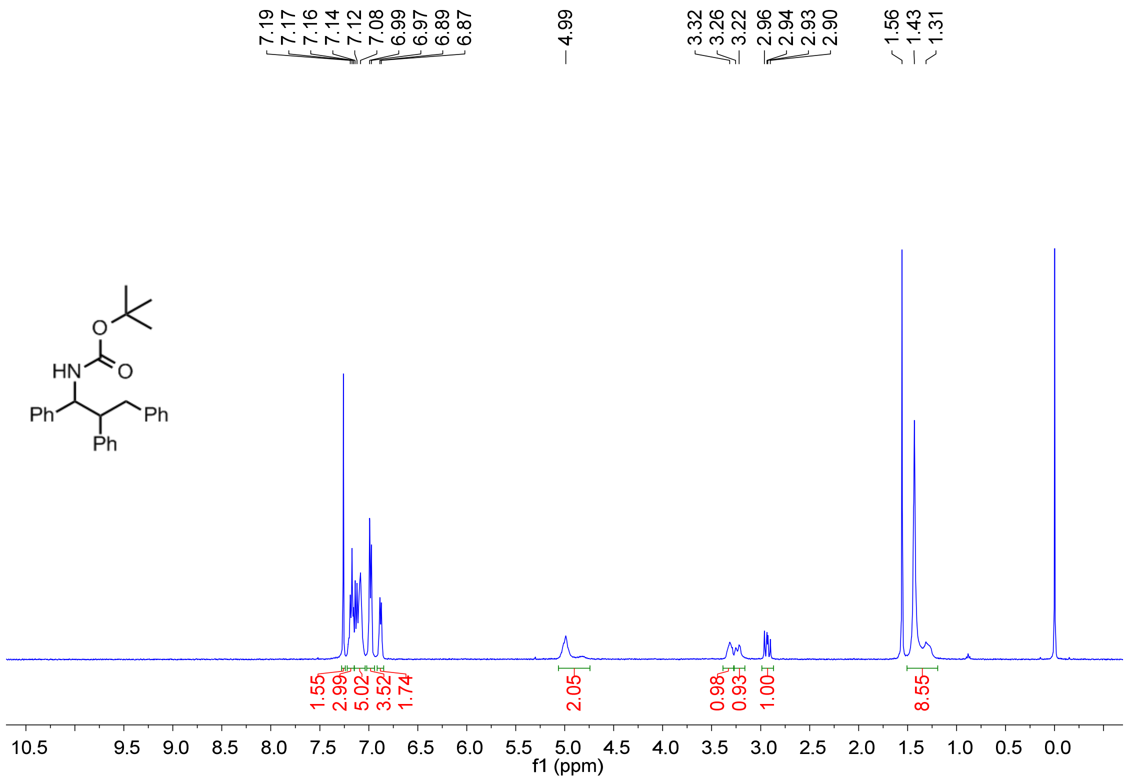

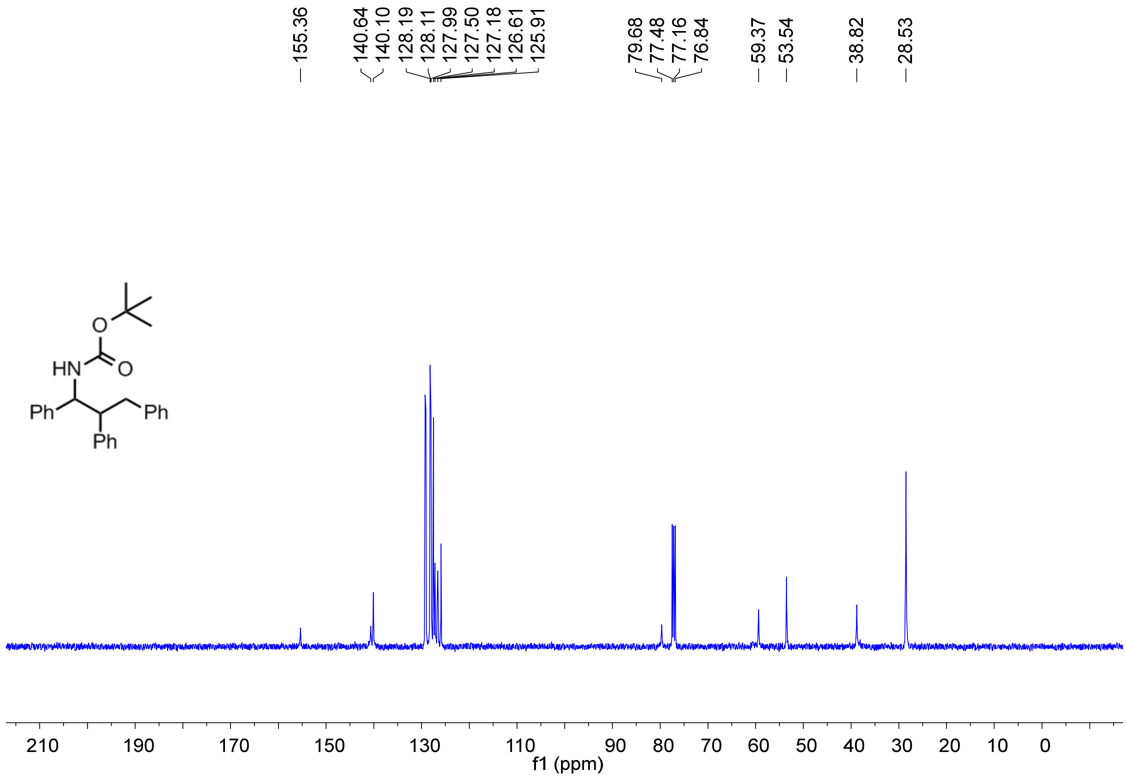

Supplementary Figure 69. ^{1}H and ^{13}C NMR spectra of compound **5** in $CDCl_3$.

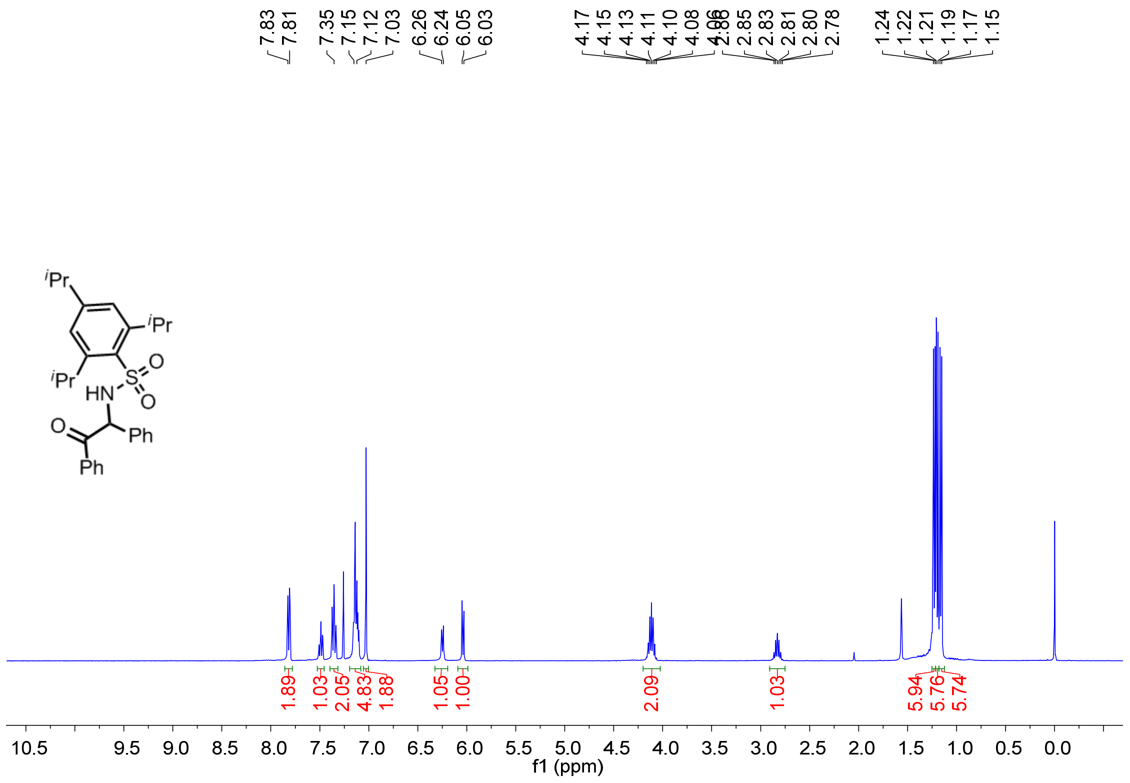

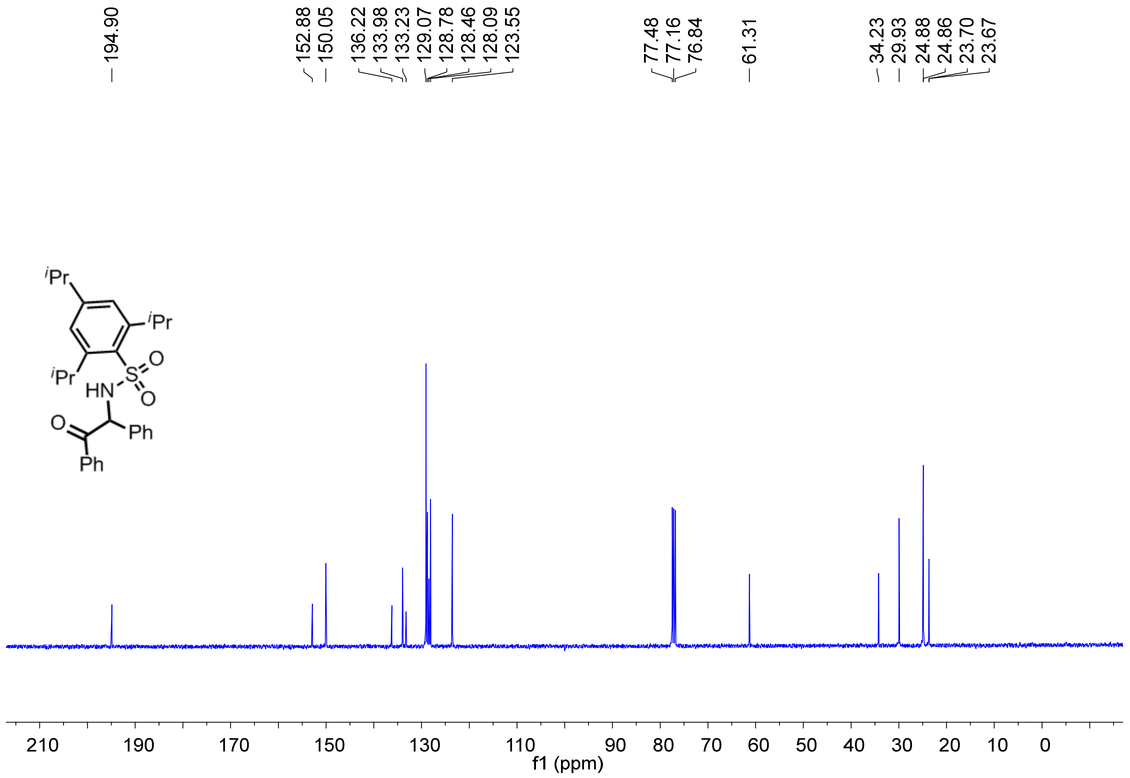

Supplementary Figure 70. ^{1}H and ^{13}C NMR spectra of compound **6** in $CDCl_3$.

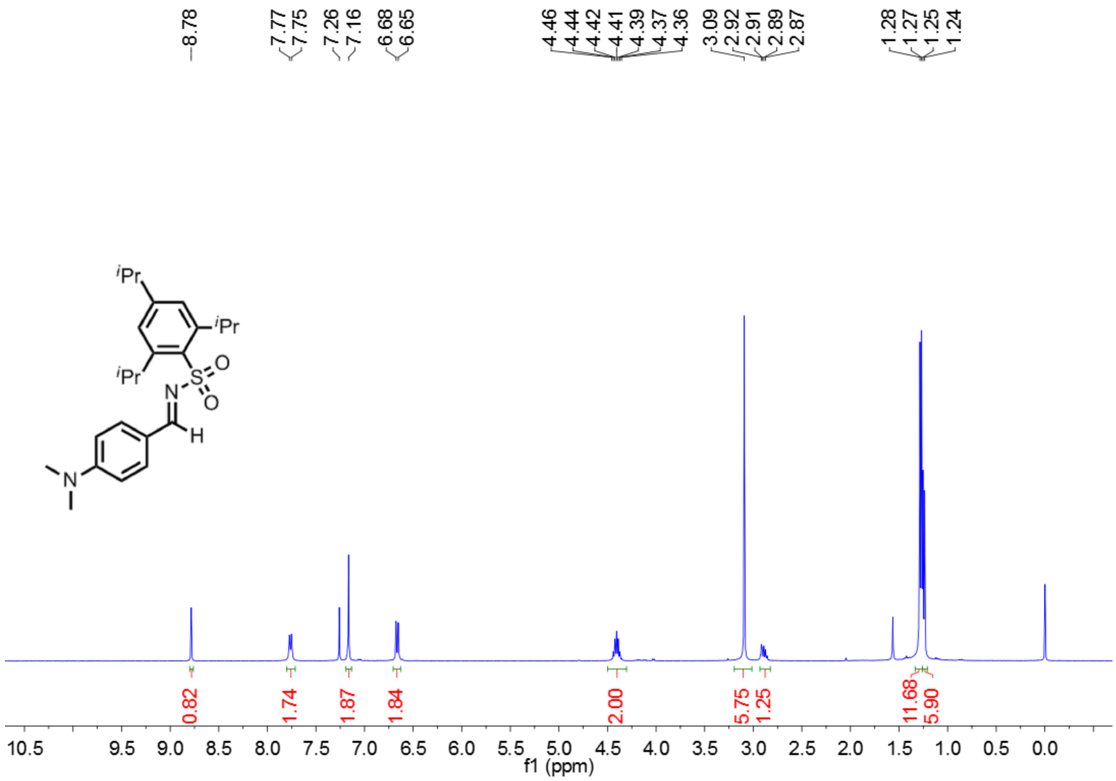

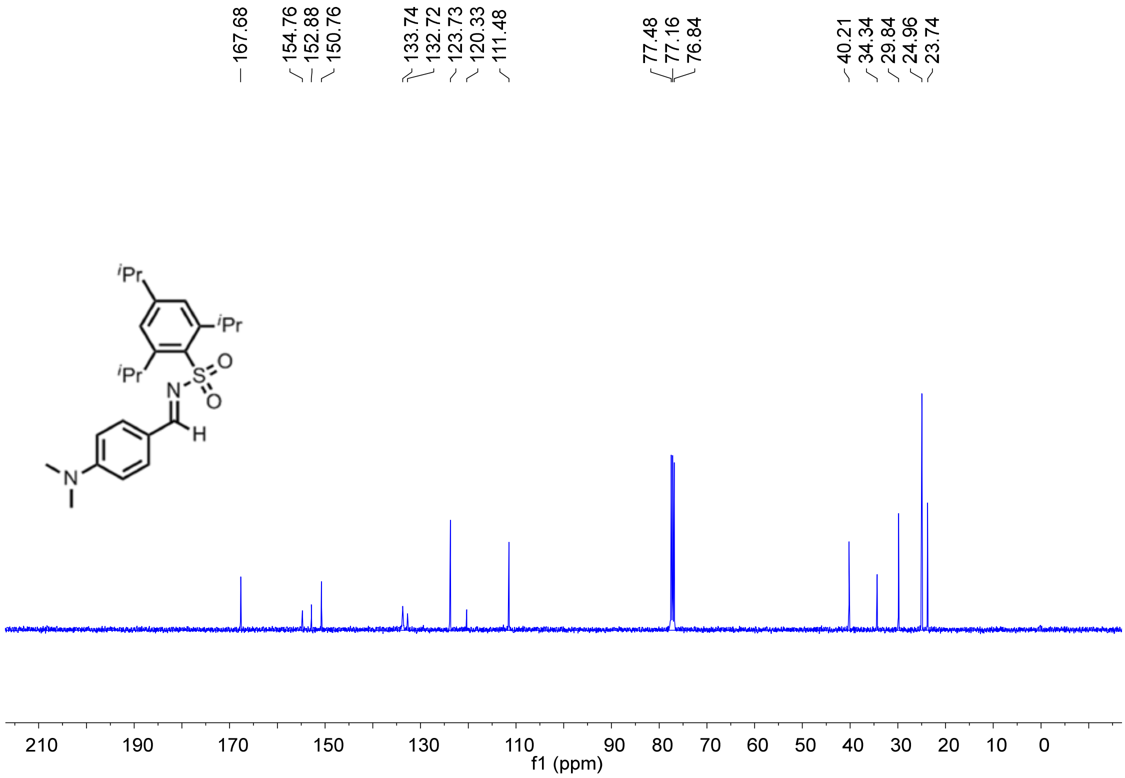

Supplementary Figure 71. ^{1}H and ^{13}C NMR spectra of TPS-imine in $CDCl_3$.

Supplementary references

1 Heffernan, S. J., Beddoes, J. M., Mahon, M. F., Hennessy, A. J. & Carbery, D. R. *Chem. Commun.* **49**, 2314 (2013).

2 Reed-Berendt, B. G. & Morrill, L. C. *J. Org. Chem.* **84**, 3715 (2019).

3 Molander, G. A., Fleury-Brégeot, N. & Hiebel, M.-A. *Org. Lett.* **13**, 1694 (2011).

4 Ghorai, M. K., Kumar, A. & Tiwari, D. P. *J. Org. Chem.* **75**, 137 (2010).

5 Zhu, M., Fujita, K.-i. & Yamaguchi, R. *Org. Lett.* **12**, 1336 (2010).

6 Chow, S. Y., Stevens, M. Y. & Odell, L. R. *J. Org. Chem.* **81**, 2681 (2016).

7 Yao, W.-W., Li, R., Li, J.-F., Sun, J. & Ye, M. *Green Chem.* **21**, 2240 (2019).

8 Ogoshi, S., Ikeda, H. & Kurosawa, H. *Angew. Chem., Int. Ed.* **46**, 4930 (2007).

9 Frisch, M. J. et al. Gaussian 09, Revision D.01; Gaussian, Inc.: Wallingford CT, 2009.

10 Lee, C., Yang, W. & Parr, R. G. *Phys. Rev. B* **37**, 785 (1988).

11 Becke, A. D. *J. Chem. Phys.* **98**, 5648 (1993).

12 Grimme, S., Antony, J., Ehrlich, S. & Krieg, H. *J. Chem. Phys.* **132**, 154104 (2010).

13 Hay, P. J. & Wadt, W. R. *J. Chem. Phys.* **82**, 299 (1985).

14 Hehre, W. J., Radom, L., Schleyer, P. v. R. & Pople, J. A. Ab Initio Molecular Orbital Theory, Wiley: New York, 1986.

15 Zhao, Y. & Truhlar, D. G. *Phys. Chem. Chem. Phys.* **10**, 2813 (2008)

16 Ribeiro, R. F., Marenich, A. V., Cramer, C. J. & Truhlar, D. G. *J. Phys. Chem. B* **115**, 14556 (2011).

17 Marenich, A. V., Cramer, C. J. & Truhlar, D. G. *J. Phys. Chem. B* **113**, 6378 (2009).

18 Zhao, Y. & Truhlar, D. G. *Theor. Chem. Acc.* **120**, 215 (2008).

19 Dolg, M., Wedig, U., Stoll, H. & Preuss, H. *J. Chem. Phys.* **86**, 866 (1987).
