# Supplementary material for: Ni-catalyzed hydroaminoalkylation of alkynes with amines
Source: Nat Commun. 2021 Jun 21;12:3800. doi: 10.1038/s41467-021-24032-9 (PMC8217523; doi:10.1038/s41467-021-24032-9)
Supplement: Supplementary file 3 — Description of Additional Supplementary Files [file 41467_2021_24032_MOESM3_ESM.docx]

**Description of Additional Supplementary Files:**

**Supplementary Data 1：**

Computed energies of the stationary points and cartesian coordinates of the stationary points
