# Supplementary material for: Ni-catalyzed hydroaminoalkylation of alkynes with amines
Source: Nat Commun. 2021 Jun 21;12:3800. doi: 10.1038/s41467-021-24032-9 (PMC8217523; doi:10.1038/s41467-021-24032-9)
Supplement: Supplementary file 4 — Supplementary Dataset 1 [file 41467_2021_24032_MOESM4_ESM.docx]

**Computed energies of the stationary points**

**Thermal corrections to Gibbs energies (TCGs) and single-point energies (SPEs)**

|  | TCG*^a^*^,^*^b^* (a.u.) | SPE*^a^* (a.u.) | SPE*^c^* (a.u.) |
| --- | --- | --- | --- |
| amine | 0.198617 | -1106.547590 | -1106.241427 |
| imine | 0.174178 | -1105.329701 | -1105.019825 |
| alkyne | 0.155379 | -539.465171 | -539.175397 |
| alkene | 0.179651 | -540.711203 | -540.412250 |
| product | 0.383361 | -1646.067838 | -1645.463652 |
| NHC | 0.194303 | -688.279819 | -687.942622 |
| PCy_3_ | 0.443264 | -1047.215295 | -1046.804797 |
| **IN1** | 0.602488 | -2503.734395 | -2504.437950 |
| **IN2** | 0.603946 | -2503.771352 | -2504.462442 |
| **IN3** | 0.602064 | -2503.751245 | -2504.442866 |
| **IN4** | 0.604244 | -2503.775136 | -2504.468224 |
| **IN5** | 0.579541 | -2502.535152 | -2503.245552 |
| **IN6** | 0.583612 | -2502.560698 | -2503.259154 |
| **IN7** | 0.812147 | -3609.160464 | -3609.537905 |
| **IN7-IPr-TPS** | 1.636291 | -4788.678666 | -4788.346013 |
| **IN7-IPr-Ts** | 1.193794 | -4159.611901 | -4159.655958 |
| **IN8***^d^* | 0.809755 | -3609.144983 | -3609.520721 |
| **IN9** | 0.808521 | -3609.115343 | -3609.502855 |
| **IN10** | 0.812009 | -3609.147657 | -3609.536770 |
| **TS1** | 0.597775 | -2503.710495 | -2504.410496 |
| **TS2** | 0.597459 | -2503.723919 | -2504.427110 |
| **TS3** | 0.580994 | -2502.504283 | -2503.209243 |
| **TS4***^d^* | 0.806132 | -3609.143437 | -3609.519570 |
| **TS5** | 0.804075 | -3609.097931 | -3609.490404 |
| **TS5-IPr-TPS** | 1.629842 | -4788.626571 | -4788.303900 |
| **TS5-IPr-Ts** | 1.188424 | -4159.555176 | -4159.610960 |
| **TS5-PCy_3_** | 1.055456 | -3968.013894 | -3968.343731 |

*^a^*Computed at the B3LYP-D3/LANL2DZ-6-31G(d) level. *^b^*A standard state at 1 atm and 298 K was used. *^c^*Computed at the SMD(toluene)/M06-D3/SDD-6-311++G(d,p)//B3LYP-D3/LANL2DZ-6-31G(d) level. *^d^*In the main text, **IN8** is higher than **TS4** by 1.6 kcal/mol in terms of Gibbs energy. However, SPE of **IN8** is lower than that of **TS4** by 1.0 kcal/mol at the B3LYP-D3/LANL2DZ-6-31G(d) level.

**Cartesian coordinates of the stationary points**

amine

S -1.95641500 -0.56462600 0.29424400

O -1.62981000 -1.25892200 1.54738800

H -2.16494400 -1.90473800 -1.45581100

C -0.99013900 0.94191200 0.23405500

C -0.01522200 1.16877900 1.20318900

C -1.22944800 1.85033200 -0.79930500

C 0.75271100 2.33120900 1.12324200

H 0.14614400 0.43659600 1.98521100

C -0.45690200 3.00787900 -0.86679300

H -2.00614500 1.64854600 -1.52936600

C 0.53501300 3.24530300 0.09080100

H 1.52536500 2.51586200 1.86352300

H -0.62851900 3.72532400 -1.66417800

O -3.34416200 -0.25539400 -0.07000200

N -1.35789800 -1.44036800 -1.04074800

C -0.22877100 -2.35832600 -0.78029400

H -0.45139300 -3.04982600 0.04165500

H -0.11838100 -2.94866700 -1.69780900

C 1.04924500 -1.60030300 -0.48906700

C 1.74333000 -1.81094000 0.70536000

C 1.53231000 -0.65341700 -1.40178400

C 2.90965300 -1.09303300 0.98360600

H 1.35647000 -2.52360000 1.42895800

C 2.69013600 0.06935600 -1.12381100

H 0.97506700 -0.46582500 -2.31537200

C 3.38379200 -0.15056800 0.07057500

H 3.43910800 -1.26369500 1.91721300

H 3.04865100 0.80996400 -1.83349100

H 4.28595000 0.41488300 0.28854000

H 1.13808600 4.14719400 0.03051200

imine

S 1.13704000 1.37113200 -0.34322400

O 1.04647400 2.23414400 0.84729800

C 2.29613300 0.05230000 0.00182800

C 2.72361800 -0.15626500 1.31348500

C 2.74589600 -0.74020600 -1.05682400

C 3.62357700 -1.19208400 1.56905800

H 2.36273900 0.49121200 2.10545700

C 3.64186900 -1.77365800 -0.78662200

H 2.40579200 -0.53636100 -2.06655800

C 4.07779200 -1.99932100 0.52295500

H 3.97195000 -1.36562700 2.58314300

H 4.00405900 -2.39923600 -1.59733900

O 1.44474400 1.94263100 -1.65660900

N -0.29693700 0.44448400 -0.55683800

C -1.28231700 0.76174500 0.20601000

C -2.57946800 0.08972400 0.11863200

C -2.82057500 -0.92796600 -0.82242700

C -3.60261700 0.47356300 1.00059600

C -4.06403700 -1.54762900 -0.87235200

H -2.02317200 -1.21263700 -1.50118600

C -4.84856000 -0.14970300 0.94769400

H -3.41664200 1.26201300 1.72606900

C -5.07926400 -1.16036000 0.01179800

H -4.24872200 -2.33289200 -1.59992500

H -5.63613400 0.15149400 1.63232300

H -6.04987300 -1.64701700 -0.03183800

H 4.77817300 -2.80451700 0.72726000

H -1.18426800 1.54902500 0.96353300

alkyne

C 0.60798400 0.00011700 -0.00013200

C -0.60798300 0.00001400 -0.00023800

C -2.03186100 0.00001400 -0.00009800

C -2.74868300 1.21411300 -0.00007800

C -2.74865600 -1.21411200 -0.00000200

C -4.14118700 1.20913000 0.00003700

H -2.20040700 2.15123800 -0.00015800

C -4.14115200 -1.20916400 0.00011100

H -2.20034500 -2.15121600 -0.00002300

C -4.84253900 -0.00002200 0.00013500

H -4.68173800 2.15183500 0.00005000

H -4.68168900 -2.15187800 0.00018200

H -5.92906600 -0.00004000 0.00022400

C 2.03186200 0.00006600 -0.00005100

C 2.74872600 1.21413700 0.00002600

C 2.74861200 -1.21408900 -0.00004900

C 4.14123100 1.20910100 0.00010600

H 2.20048700 2.15128300 0.00002500

C 4.14110700 -1.20919300 0.00003100

H 2.20026500 -2.15117100 -0.00010900

C 4.84253900 -0.00007500 0.00010800

H 4.68181500 2.15178800 0.00016700

H 4.68161100 -2.15192500 0.00003300

H 5.92906600 -0.00013500 0.00017100

alkene

C 0.67447800 1.86818700 -0.00462100

C -0.67446800 1.86820900 0.00453700

C -1.62322500 0.74111800 0.07821700

C -1.36547900 -0.42180900 0.82783600

C -2.86681600 0.85056800 -0.56915000

C -2.30527300 -1.44732000 0.89835900

H -0.42424800 -0.51253700 1.35951700

C -3.80548400 -0.17902300 -0.50474500

H -3.09195300 1.75165200 -1.13533600

C -3.52705400 -1.33476600 0.22747300

H -2.08694900 -2.33530900 1.48591600

H -4.75593600 -0.07587300 -1.02180500

H -4.25852900 -2.13638400 0.28436300

C 1.62321800 0.74108500 -0.07825800

C 1.36541600 -0.42195200 -0.82769400

C 2.86687100 0.85063300 0.56897200

C 2.30521100 -1.44746200 -0.89815900

H 0.42414400 -0.51276200 -1.35928600

C 3.80554400 -0.17896000 0.50462700

H 3.09206100 1.75179700 1.13500900

C 3.52705500 -1.33480700 -0.22739900

H 2.08684400 -2.33553400 -1.48557500

H 4.75604200 -0.07572400 1.02158500

H 4.25852800 -2.13643100 -0.28424600

H 1.15228700 2.84743300 0.03396300

H -1.15225300 2.84746500 -0.03406700

product

C -1.76465700 -1.05006000 -0.31923900

C -1.29846200 0.09726500 0.22150300

C 0.22082100 0.28378300 0.34544600

H 0.39277000 1.13070900 1.01746900

C 0.95265700 -0.91666900 0.93048800

C 1.58173000 -1.87133600 0.12525900

C 1.00296800 -1.06191600 2.32240500

C 2.25590800 -2.94922100 0.70208700

H 1.57228100 -1.74134300 -0.95136700

C 1.67198600 -2.14027500 2.90061000

H 0.51866400 -0.32052800 2.95454900

C 2.30391500 -3.08760800 2.09015600

H 2.75424900 -3.67404700 0.06387700

H 1.70770000 -2.23668300 3.98260000

H 2.83461900 -3.92309100 2.53891200

S 1.99101600 1.76762900 -1.11979900

C 3.42770100 0.75858300 -0.77416500

C 3.98173800 0.00265100 -1.80853200

C 3.90835700 0.68418300 0.53295500

C 5.03883500 -0.85815200 -1.51741900

H 3.58845800 0.09596100 -2.81546200

C 4.96165900 -0.18672200 0.81219200

H 3.45594900 1.29017900 1.30975500

C 5.52130000 -0.95810800 -0.20860400

H 5.48516100 -1.45167400 -2.31018300

H 5.33912900 -0.26627400 1.82717500

O 1.99716800 2.17088700 -2.53012500

O 1.87791300 2.76367700 -0.04364100

N 0.74081500 0.63215500 -1.00118700

H 0.02406400 0.86359900 -1.68463600

H -1.02848800 -1.83438300 -0.49148300

C -2.12700700 1.23476600 0.69737800

C -1.74176000 2.56572600 0.45335000

C -3.28646800 1.00409000 1.45987300

C -2.51223700 3.62836200 0.92664300

H -0.83156600 2.77891800 -0.09963500

C -4.05108400 2.06639100 1.93571300

H -3.58040700 -0.01752100 1.67795700

C -3.67139000 3.38423300 1.66505400

H -2.19944900 4.64824900 0.72025400

H -4.94111500 1.86524300 2.52612800

H -4.26803000 4.21274300 2.03708200

C -3.13196100 -1.41620400 -0.72684700

C -4.05126100 -0.48901000 -1.25250600

C -3.51972100 -2.76601800 -0.64936600

C -5.32015100 -0.89629400 -1.65605000

H -3.76241400 0.55206000 -1.34809900

C -4.79386300 -3.17230000 -1.04438200

H -2.81297300 -3.49816900 -0.26536100

C -5.70101800 -2.23747200 -1.54704700

H -6.01349400 -0.16513800 -2.06326100

H -5.07534400 -4.21903900 -0.96538300

H -6.69250500 -2.55148100 -1.86191600

H 6.33891700 -1.63796900 0.01453000

NHC

C 0.00001100 0.33823700 -0.09138900

C -0.67596100 -1.79010800 0.50242600

C 0.67593600 -1.79010800 0.50249700

H -1.37446900 -2.56710400 0.76940200

H 1.37442600 -2.56711200 0.76948400

N 1.06606900 -0.49616700 0.13963300

N -1.06607600 -0.49616300 0.13962200

C -2.41870900 -0.06654700 0.01158700

C -3.42745600 -0.97952100 -0.31287800

C -2.73296100 1.28238100 0.21443700

C -4.74986600 -0.54461200 -0.41655100

H -3.18283200 -2.01838900 -0.51077400

C -4.05414500 1.70748600 0.09642900

H -1.93023400 1.97148300 0.44943800

C -5.06998000 0.79795000 -0.21223900

H -5.52757200 -1.25981000 -0.67018000

H -4.29168900 2.75595900 0.25465000

H -6.09953100 1.13404300 -0.29671600

C 2.41871100 -0.06654800 0.01163300

C 2.73293500 1.28239700 0.21437400

C 3.42749100 -0.97953900 -0.31270300

C 4.05412500 1.70750900 0.09639400

H 1.93019100 1.97151200 0.44927700

C 4.74989500 -0.54462300 -0.41635500

H 3.18290000 -2.01843000 -0.51051500

C 5.06998400 0.79796700 -0.21214300

H 4.29163500 2.75600200 0.25453900

H 5.52762700 -1.25982600 -0.66988600

H 6.09953600 1.13405800 -0.29660800

PCy_3_

P 0.13802000 0.04146000 -0.96189900

C -0.64242300 1.48581900 0.00295200

C -2.17121800 1.41567100 0.19990500

C -2.69969400 2.64452800 0.96146500

C -2.32783100 3.95643500 0.25789500

C -0.81394700 4.03730200 0.02044600

C -0.30638800 2.80282800 -0.74088000

C 1.75689900 -0.17000700 0.01184600

C 2.64850700 1.08730600 -0.07002600

C 3.98892100 0.89671000 0.65990600

C 4.74874200 -0.32919900 0.13844900

C 3.87606800 -1.58599200 0.24381600

C 2.53632900 -1.40556900 -0.48935400

C -0.76002600 -1.54420400 -0.43685500

C -2.10471900 -1.72420000 -1.17667400

C -2.69174600 -3.12483200 -0.93350000

C -2.84919100 -3.41025900 0.56720800

C -1.51051400 -3.24600100 1.30072100

C -0.90243800 -1.85137000 1.06738700

H -0.17967700 1.51837500 1.00167600

H -2.66352000 1.36300300 -0.78212800

H -2.45617700 0.51094500 0.74294200

H -3.78867200 2.56780200 1.07834800

H -2.27414900 2.64465000 1.97613100

H -2.84558900 4.00383200 -0.71157600

H -2.67461600 4.81746300 0.84358400

H -0.56118200 4.95024100 -0.53425500

H -0.29606400 4.10356100 0.98910500

H -0.77857700 2.76843900 -1.73333400

H 0.76902100 2.89377100 -0.92284600

H 1.51165300 -0.32638900 1.07324400

H 2.84092700 1.33001500 -1.12551400

H 2.13110600 1.94912100 0.36417400

H 4.59939100 1.80289900 0.55445200

H 3.79678400 0.77013600 1.73589700

H 5.02227900 -0.16533400 -0.91452200

H 5.68697000 -0.46445300 0.69159200

H 4.40557900 -2.45833500 -0.16060600

H 3.67955400 -1.80091700 1.30483200

H 2.72555000 -1.29610000 -1.56741800

H 1.93857200 -2.31600200 -0.36673600

H -0.09220300 -2.31333200 -0.85494200

H -2.83009900 -0.97762700 -0.83428400

H -1.96290500 -1.55247200 -2.25090100

H -3.65817700 -3.22042700 -1.44523500

H -2.02431200 -3.87937600 -1.37609700

H -3.58248800 -2.70870700 0.99287700

H -3.25008300 -4.41953700 0.72610700

H -1.63717700 -3.42445500 2.37662100

H -0.80585100 -4.00863200 0.93647100

H -1.54031200 -1.09835000 1.54751000

H 0.07216400 -1.79548700 1.56507900

**IN1**

Ni 0.52540800 0.06746000 -0.09964600

C 0.51188200 -1.44874100 1.05873500

S -2.07825200 0.10497200 -2.03633400

O -1.42229500 -1.07310300 -2.60912200

C 1.69107000 -0.93695300 0.97933300

C -3.83873600 -0.23284100 -1.94866700

C -4.25512500 -1.48139500 -1.47573000

C -4.75040000 0.76616300 -2.29078100

C -5.62059100 -1.72720300 -1.34524900

H -3.52636000 -2.24041100 -1.20965400

C -6.11587900 0.50154300 -2.16537600

H -4.38640000 1.72265600 -2.65048300

C -6.54850500 -0.73912700 -1.69159400

H -5.95983300 -2.69039300 -0.97555100

H -6.83922300 1.26577600 -2.43442700

O -1.88820700 1.45195800 -2.58168700

N -1.58915900 0.15892300 -0.38018600

C -2.28992300 1.22316000 0.41192100

H -1.88953300 2.17376700 0.06948200

H -1.81724200 -0.76277800 0.01227600

C -2.05029700 1.02436800 1.88625100

C -2.98735000 0.33683600 2.66402900

C -0.87879500 1.50532700 2.48461300

C -2.76447200 0.13579300 4.02740400

H -3.89273600 -0.04833800 2.19912000

C -0.65219000 1.30007400 3.84439300

H -0.14238700 2.02018100 1.87593500

C -1.59570500 0.61796900 4.61869300

H -3.49645600 -0.40436400 4.62133800

H 0.26288100 1.67008400 4.29857200

H -1.41772300 0.46037700 5.67937200

C 3.10743300 -1.05902300 1.24792500

C 3.94740200 0.06838100 1.17779400

C 3.69276000 -2.31020600 1.52574200

C 5.32258100 -0.04945300 1.37123100

H 3.49923000 1.03125200 0.95184200

C 5.06857800 -2.42822300 1.70813200

H 3.05214800 -3.18634500 1.57253000

C 5.89210000 -1.29979200 1.63028300

H 5.95336100 0.83478100 1.31217800

H 5.50318500 -3.40461100 1.90927500

H 6.96563900 -1.39469400 1.77232800

C -0.50345200 -2.36515300 1.49917400

C -1.29064200 -3.04145400 0.53530000

C -0.82754300 -2.54057400 2.86027200

C -2.34758100 -3.86554300 0.92485400

H -1.03850800 -2.91883600 -0.51629300

C -1.89329200 -3.35314800 3.23858300

H -0.25083200 -2.00508800 3.60730400

C -2.66069700 -4.02121300 2.27788500

H -2.92470200 -4.39303900 0.16779000

H -2.13079100 -3.46498100 4.29397000

H -3.48837300 -4.65671900 2.58136800

H -7.61167700 -0.93809000 -1.59028200

C 1.68011100 1.23859700 -1.07866900

C 3.65752800 1.79445000 -2.11158000

C 3.17531100 2.92597400 -1.54708200

H 4.53807200 1.61957600 -2.70893100

H 3.54282100 3.94001100 -1.54925500

N 1.96725000 2.57631400 -0.94165700

N 2.74052700 0.78429300 -1.83614400

C 1.14319500 3.49314200 -0.22678200

C 1.63239900 4.11046500 0.92694500

C -0.14222800 3.77029000 -0.69713500

C 0.82167600 5.00887200 1.62290200

H 2.63026700 3.86654500 1.27952400

C -0.94584700 4.66911200 0.00668500

H -0.50198600 3.26394400 -1.58733800

C -0.46876200 5.28698300 1.16588600

H 1.19477400 5.48164500 2.52694900

H -1.94857500 4.88403800 -0.35265900

H -1.10070800 5.98167100 1.71181500

C 2.93793600 -0.58592000 -2.18709200

C 1.86490300 -1.33267200 -2.67818200

C 4.19250300 -1.17123800 -2.00286800

C 2.05486100 -2.67992600 -2.98131300

H 0.89396000 -0.86955300 -2.80034500

C 4.37383300 -2.51766600 -2.31949900

H 4.99730300 -0.59671500 -1.55665200

C 3.30703100 -3.27559500 -2.80699000

H 1.21595900 -3.26032900 -3.35486900

H 5.34456900 -2.97706200 -2.15642600

H 3.44911000 -4.32682000 -3.04296500

H -3.36740100 1.20025000 0.20472100

**IN2**

Ni -0.04385700 0.08048400 -0.31997200

C 2.55758900 -1.05679300 -0.72629500

S -1.91219300 0.51296100 -2.12565200

O -0.70900600 -0.42230900 -2.18567300

C 1.31208500 -1.24829700 -0.25153800

C -3.34615900 -0.56577800 -1.98881000

C -4.24142900 -0.68311800 -3.05162200

C -3.51437300 -1.29371200 -0.80784400

C -5.32512900 -1.55611200 -2.92862900

H -4.08550400 -0.09449600 -3.94936900

C -4.59725500 -2.16273700 -0.69749900

H -2.81163800 -1.17028100 0.00775500

C -5.50121500 -2.29599400 -1.75686600

H -6.03108300 -1.65645100 -3.74837700

H -4.73502600 -2.72856700 0.21941800

O -2.16559800 1.40389700 -3.27048100

N -1.63290600 1.18443300 -0.71140500

C -2.28116900 2.42947500 -0.31222600

H -3.10858800 2.66824600 -0.99320600

H 2.83053300 -0.04591400 -1.01846100

C -1.28847500 3.57402800 -0.29682000

C -0.75644300 4.05309000 0.90350500

C -0.83068800 4.11369800 -1.50793500

C 0.21562000 5.05780300 0.90243300

H -1.08937700 3.62515600 1.84674800

C 0.13068000 5.12401400 -1.51142000

H -1.21920800 3.71372700 -2.44140800

C 0.65832800 5.59834800 -0.30525700

H 0.62838800 5.41242100 1.84357500

H 0.47556600 5.53805600 -2.45549100

H 1.41541600 6.37782200 -0.30970000

C 0.77511900 -2.50713600 0.29944000

C -0.38076000 -3.09857700 -0.24954800

C 1.33325600 -3.09010200 1.45472400

C -0.95846200 -4.22268400 0.34023100

H -0.82206400 -2.65118300 -1.13453200

C 0.75160700 -4.21257900 2.04503100

H 2.22958000 -2.64769600 1.88069800

C -0.40131300 -4.78266800 1.49444000

H -1.85268200 -4.65697800 -0.10087100

H 1.19751200 -4.64337500 2.93839200

H -0.85486300 -5.65580600 1.95640400

C 3.64881700 -2.03358600 -0.92538400

C 4.97335700 -1.55504900 -0.95256700

C 3.44699700 -3.41422500 -1.11630100

C 6.05342000 -2.41890400 -1.12698800

H 5.14642100 -0.48861300 -0.82757000

C 4.52657900 -4.27740900 -1.29417200

H 2.43724700 -3.80752700 -1.13759000

C 5.83619700 -3.78853500 -1.29418000

H 7.06609800 -2.02213900 -1.13743900

H 4.34308100 -5.33881000 -1.44325300

H 6.67517100 -4.46525900 -1.43473900

H -6.34631300 -2.97330300 -1.66654600

C 0.39402000 0.75537200 1.36664500

C 1.35844000 1.95602700 3.04431800

C 0.20263800 1.44574500 3.53114800

H 2.10436700 2.59295400 3.49157300

H -0.25686500 1.51119500 4.50438200

N -0.37482900 0.71039400 2.49662000

N 1.46167300 1.52499000 1.72459300

C -1.62427500 0.01861200 2.62910300

C -1.67046200 -1.36685500 2.47921700

C -2.77458200 0.74686000 2.94288800

C -2.89229300 -2.02653200 2.62086000

H -0.76556200 -1.91437200 2.25437100

C -3.99075100 0.07818100 3.08613200

H -2.71448200 1.82495100 3.05825600

C -4.05182700 -1.30815600 2.92070400

H -2.92171300 -3.10367200 2.48951200

H -4.88953100 0.64184500 3.31851100

H -5.00136400 -1.82501400 3.02749200

C 2.52679800 1.90670200 0.84890100

C 2.22916200 2.45752700 -0.39752000

C 3.84698400 1.72055000 1.26070800

C 3.27529800 2.80593300 -1.25085100

H 1.19741600 2.60219900 -0.69085400

C 4.88644500 2.08648400 0.40486500

H 4.05352400 1.26246900 2.22333600

C 4.60227800 2.62305300 -0.85398500

H 3.04526600 3.22792500 -2.22439900

H 5.91597100 1.93360700 0.71535600

H 5.41328900 2.89552400 -1.52325900

H -2.71455100 2.29321900 0.68529400

**IN3**

Ni -0.28930700 -0.20886500 -0.22259000

C -2.01093400 0.52878400 -0.43795100

S 2.17872700 -2.07786200 -0.21725000

O 1.69432900 -3.06906800 -1.20882400

C -3.01250800 -0.16953900 -1.01593900

C 3.93508700 -1.84956900 -0.54950000

C 4.64592000 -2.87709400 -1.17130000

C 4.56336000 -0.66271300 -0.16204600

C 6.01314200 -2.71428500 -1.40186200

H 4.12295900 -3.77654400 -1.47881800

C 5.92849900 -0.50878700 -0.40263800

H 3.98113000 0.13221700 0.28992800

C 6.65359000 -1.53325800 -1.01801500

H 6.57572000 -3.50646200 -1.88830700

H 6.42313400 0.41607700 -0.11842200

O 2.00054400 -2.41051400 1.21144600

N 1.56386300 -0.58274600 -0.43306300

C 1.49917900 -0.07229000 -1.79996400

H 1.74874800 -0.82099900 -2.56198700

H 0.40868900 0.16572700 -2.03252100

C 2.27337100 1.21778600 -1.98476700

C 2.94946300 1.48698100 -3.17718800

C 2.29961400 2.16463500 -0.95250400

C 3.64447700 2.68869300 -3.34018200

H 2.94273300 0.75076600 -3.97807900

C 2.99089800 3.36393100 -1.11441900

H 1.79173400 1.94432300 -0.02015300

C 3.66559200 3.63054700 -2.30990100

H 4.17370600 2.88426300 -4.26914600

H 3.00483600 4.08541100 -0.30163300

H 4.20741700 4.56432500 -2.43599600

C -4.35480300 0.27327000 -1.43967700

C -5.35148900 -0.70636300 -1.61669100

C -4.69708000 1.61501000 -1.69853800

C -6.64531200 -0.36074100 -2.00284600

H -5.09892300 -1.74836600 -1.43603700

C -5.98946900 1.95916100 -2.08906200

H -3.94277700 2.38741900 -1.60274200

C -6.97280800 0.97655900 -2.23803300

H -7.39768300 -1.13630900 -2.12513300

H -6.22890600 3.00127200 -2.28600600

H -7.97941500 1.24989200 -2.54360700

C -2.07441200 1.91484200 0.06325700

C -1.18764000 2.89680300 -0.41777400

C -2.94093100 2.26607200 1.11725600

C -1.16865000 4.18030100 0.12748500

H -0.49885600 2.63951300 -1.21794800

C -2.91892900 3.54789500 1.66616000

H -3.63262000 1.51772500 1.49416400

C -2.03131200 4.51264800 1.17542600

H -0.47043600 4.91669900 -0.26245400

H -3.59670800 3.79703600 2.47911000

H -2.01510300 5.51067400 1.60517600

H 7.71653500 -1.40748700 -1.20632100

C -0.61545200 -0.76852000 1.50191100

C -1.28355000 -1.91178800 3.33954900

C -0.43333600 -0.94074500 3.75177600

H -1.80190100 -2.68800400 3.87887200

H -0.06722400 -0.67679800 4.73082200

N -0.03945100 -0.24392400 2.61290400

N -1.38655400 -1.79139300 1.95454300

C 0.92513600 0.82294900 2.60454800

C 0.48843200 2.14477400 2.54277100

C 2.28256900 0.50033100 2.64427600

C 1.43703100 3.17023300 2.53277300

H -0.57156900 2.36343600 2.48366700

C 3.21935700 1.53363400 2.64235300

H 2.57740900 -0.54321300 2.62482000

C 2.79891600 2.86662000 2.58903800

H 1.10194400 4.20101900 2.47037800

H 4.27904000 1.29526700 2.66507100

H 3.53439100 3.66649700 2.57981700

C -2.15815800 -2.65779200 1.11812600

C -1.54213500 -3.30610800 0.04550400

C -3.51820200 -2.82379200 1.38240400

C -2.31745300 -4.11425400 -0.78705500

H -0.48070400 -3.18084800 -0.13708400

C -4.27849500 -3.64939600 0.55375700

H -3.97620400 -2.28535000 2.20668800

C -3.68112700 -4.28928600 -0.53597000

H -1.84447500 -4.61369900 -1.62737600

H -5.33959500 -3.77504200 0.74864200

H -4.27733600 -4.92322600 -1.18629900

H -2.82941300 -1.22691600 -1.20150700

**IN4**

Ni 0.02223700 -0.09638500 -0.30841600

C -1.32892300 1.44607500 -1.53060200

S 2.32434900 -1.91765500 0.48978500

O 1.81605100 -3.07353900 -0.28201800

C -1.54440800 0.19319500 -2.05378000

C 4.11089300 -1.88152000 0.28466100

C 4.75911300 -3.00371300 -0.22942700

C 4.82117800 -0.74092200 0.66954400

C 6.15010400 -2.98396900 -0.35501600

H 4.17371000 -3.86655300 -0.52855300

C 6.20828800 -0.73096200 0.53601300

H 4.28582100 0.12544700 1.04326500

C 6.87263200 -1.85188700 0.02696000

H 6.66705200 -3.85170200 -0.75540900

H 6.77198400 0.15191700 0.82482100

O 2.01901700 -1.86197700 1.93224000

N 1.88858100 -0.44574400 -0.13889000

C 1.59937800 -0.34306000 -1.49095100

H 1.53577500 -1.25279700 -2.09497000

H -0.45492300 1.95860200 -1.92678000

C 1.89563100 0.91265400 -2.19532500

C 1.67653000 1.00372200 -3.58246400

C 2.28215400 2.07835800 -1.50235500

C 1.81109000 2.22116300 -4.25052700

H 1.39065300 0.11075800 -4.13517600

C 2.41449900 3.29085800 -2.17266300

H 2.44299700 2.01829900 -0.43219300

C 2.17473400 3.37405800 -3.54966900

H 1.63260400 2.26876300 -5.32196800

H 2.70201200 4.17882300 -1.61437500

H 2.27640200 4.32282400 -4.06968600

C -2.72534500 -0.69384300 -2.00992800

C -2.52132600 -2.08297500 -2.08327800

C -4.04291300 -0.20629900 -2.03229300

C -3.60065300 -2.95807100 -2.16809700

H -1.50537900 -2.46765700 -2.05526500

C -5.12316200 -1.08444700 -2.11171600

H -4.21703000 0.86430600 -2.01017200

C -4.90667200 -2.46252600 -2.18102000

H -3.42192800 -4.02770600 -2.21069600

H -6.13555300 -0.68924100 -2.13585200

H -5.74936000 -3.14559400 -2.24825500

C -2.16357200 2.30789200 -0.67245100

C -1.96749900 3.69769000 -0.76935100

C -3.11061400 1.83492400 0.25474000

C -2.71476500 4.58737200 0.00214800

H -1.21542200 4.07701900 -1.45676100

C -3.84086800 2.72120900 1.04161900

H -3.25769100 0.76802900 0.36493700

C -3.65439900 4.10268700 0.91461900

H -2.55241900 5.65716800 -0.09941800

H -4.55632300 2.33156800 1.76069900

H -4.23182200 4.79146900 1.52544400

H 7.95432800 -1.83891600 -0.07637500

C -0.86752400 -0.04817800 1.43366300

C -2.28326200 -0.30521100 3.21026000

C -1.57099200 0.82636300 3.42463100

H -3.02267600 -0.80867100 3.81202600

H -1.57197600 1.52885800 4.24269600

N -0.70706800 0.95746000 2.34291100

N -1.83591700 -0.83245700 1.99847900

C 0.29121800 1.97926500 2.23429000

C -0.09672800 3.31147800 2.09940500

C 1.63769200 1.61085100 2.28124200

C 0.88614600 4.29775700 1.99978800

H -1.15071200 3.56058300 2.04538100

C 2.60966500 2.60687200 2.18467200

H 1.89816700 0.56131600 2.36589800

C 2.23798100 3.94743900 2.04159100

H 0.59245200 5.33690400 1.88085800

H 3.66087600 2.33372000 2.21987100

H 3.00106700 4.71716100 1.96322900

C -2.25482800 -2.10506200 1.49985200

C -1.28763700 -3.03101200 1.10503500

C -3.61320800 -2.42765800 1.47686100

C -1.69144200 -4.29780900 0.68625000

H -0.23922000 -2.76986900 1.15592700

C -4.00384000 -3.70487200 1.07502000

H -4.35232200 -1.68280100 1.75747100

C -3.04511100 -4.64284300 0.68404700

H -0.93492900 -5.01358100 0.37854200

H -5.05955100 -3.95895900 1.05122800

H -3.35465900 -5.63611300 0.37030000

H -0.78933600 -0.13305300 -2.76687400

**IN5**

S 1.83670900 -0.69846900 -1.77014700

O 1.55943200 -2.14456200 -1.77295600

O 1.21886300 0.13910900 -2.82487100

Ni -0.34183200 0.06204800 0.17885800

C -1.50232000 1.67632900 0.28805400

C 1.29587100 1.20986500 -0.08027600

C 1.74332900 1.89347600 1.14691600

C 1.66422400 3.29355800 1.23919200

C 2.25807900 1.17084300 2.23717600

C 2.05818500 3.94893500 2.40536800

H 1.28886300 3.86740500 0.39760500

C 2.65370700 1.82929000 3.39784400

H 2.34591200 0.09493200 2.15162500

C 2.54837800 3.22137900 3.49242200

H 1.98278000 5.03191400 2.46168800

H 3.05141300 1.25478400 4.23142100

H 2.85657400 3.73407000 4.40001000

H 1.18312600 1.83016700 -0.96941800

C -2.10404800 0.66773200 0.75829700

N 1.54167600 -0.13385100 -0.22181500

C -3.34138100 0.04891900 1.18196400

C -4.56352300 0.67676200 0.87150600

C -3.37014100 -1.17895200 1.86352900

C -5.77373900 0.08275100 1.21826600

H -4.54526100 1.62105200 0.33578300

C -4.58327600 -1.77082400 2.21173800

H -2.43283200 -1.66985200 2.10096800

C -5.78995200 -1.14616900 1.88586800

H -6.70783000 0.57529200 0.96076100

H -4.58643600 -2.72228400 2.73740500

H -6.73553300 -1.61047400 2.15305300

C -1.34940800 3.06519000 -0.05817900

C -0.93000200 3.43512100 -1.35036400

C -1.58775900 4.07036700 0.89654900

C -0.75228500 4.77797200 -1.67492700

H -0.73497700 2.65531100 -2.08106800

C -1.40079100 5.41212400 0.56728100

H -1.89534600 3.78356200 1.89730100

C -0.98026200 5.77108100 -0.71601800

H -0.42684400 5.05117000 -2.67522100

H -1.57582300 6.17874900 1.31755400

H -0.82971800 6.81729500 -0.96815100

C -0.80798700 -1.80932500 0.01407300

C -0.71559100 -4.08423500 0.09503700

C -1.69094300 -3.75228500 -0.78350400

H -0.34660600 -5.04120100 0.42761100

H -2.33756000 -4.35792500 -1.39783400

N -1.73416300 -2.36101200 -0.82127500

N -0.19797900 -2.88689000 0.58199800

C 3.61544700 -0.46326700 -1.86461500

C 4.42096900 -0.99427300 -0.85281600

C 4.15811900 0.22841000 -2.94595600

C 5.80145200 -0.82245100 -0.93378300

H 3.96674400 -1.51311300 -0.01524700

C 5.54402700 0.38966400 -3.01719100

H 3.49843500 0.63184500 -3.70668000

C 6.36266600 -0.13345400 -2.01447600

H 6.44076600 -1.22224300 -0.15140400

H 5.98140300 0.92710600 -3.85400600

H 7.43983500 -0.00140100 -2.07108400

C 0.89689600 -2.80892500 1.49531400

C 0.74891200 -2.12341300 2.70063300

C 2.10296100 -3.42775600 1.16192900

C 1.82155100 -2.06777200 3.59150000

H -0.18765700 -1.62137100 2.91868600

C 3.16878600 -3.36976100 2.06043700

H 2.20545900 -3.89632800 0.18933900

C 3.03029300 -2.69258100 3.27582600

H 1.71241700 -1.52654500 4.52683300

H 4.11242500 -3.84357800 1.80397000

H 3.86395100 -2.64630700 3.97089400

C -2.63484200 -1.61309800 -1.64454300

C -2.13665700 -0.63177300 -2.50390500

C -4.00414500 -1.86891100 -1.55542600

C -3.03445300 0.11374500 -3.26813800

H -1.06820300 -0.45596200 -2.56839800

C -4.89036000 -1.12634800 -2.33504600

H -4.36902200 -2.60288400 -0.84408500

C -4.40800200 -0.13056300 -3.18758100

H -2.65309700 0.88304700 -3.93358200

H -5.95769800 -1.31075200 -2.25501800

H -5.10070600 0.45431500 -3.78657900

**IN6**

S -1.95766100 0.69062600 -1.94551800

O -1.04869200 1.90866600 -1.95694600

O -2.26070200 0.03909400 -3.23053600

Ni 0.33006900 0.78492100 -0.52108100

C 0.07745900 -1.88274100 0.11214900

C -1.21501500 -1.62259000 -0.65837000

C -2.49624700 -2.05479400 0.04141400

C -3.58594800 -2.49927300 -0.71259800

C -2.64684600 -1.88905300 1.42377200

C -4.81135500 -2.77163800 -0.09901800

H -3.47855900 -2.61097600 -1.78935200

C -3.86618400 -2.16482700 2.03971600

H -1.80045600 -1.53994200 2.00881400

C -4.95417900 -2.60506500 1.27892100

H -5.65240500 -3.11097800 -0.69822100

H -3.96997700 -2.03784800 3.11459800

H -5.90574800 -2.81796100 1.75929600

H -1.16808200 -2.11797600 -1.64015600

C 0.91700400 -0.82837900 0.29079400

N -1.21559100 -0.17410400 -0.82942400

C 2.24070800 -0.89871400 0.93813100

C 3.26167000 -1.71266100 0.41819200

C 2.53463600 -0.09929200 2.05930000

C 4.52955400 -1.72973200 1.00021500

H 3.04862400 -2.32634200 -0.45148300

C 3.80105000 -0.11517200 2.63990600

H 1.75333000 0.53935700 2.46288200

C 4.80823700 -0.92984100 2.11102900

H 5.30408000 -2.36655200 0.57950000

H 4.00436200 0.50748200 3.50831000

H 5.79711500 -0.94193200 2.56213400

C 0.29963000 -3.26295900 0.61690000

C -0.03881400 -4.37512300 -0.17489200

C 0.81191500 -3.50347400 1.90442600

C 0.14837200 -5.67572100 0.29229000

H -0.44666000 -4.22170000 -1.16974700

C 0.99899100 -4.80247700 2.37239300

H 1.05906800 -2.65978200 2.53998300

C 0.67086900 -5.89677200 1.56800000

H -0.11673200 -6.51778500 -0.34219200

H 1.39507200 -4.96035700 3.37247800

H 0.81409500 -6.91000100 1.93426000

C 1.84050000 1.90688500 -0.21231400

C 3.21950500 3.44931400 0.73527400

C 3.92530300 2.81187700 -0.23078900

H 3.48619500 4.26159500 1.39210100

H 4.93275700 2.95620500 -0.58611300

N 3.06442900 1.88290400 -0.80863500

N 1.94218600 2.89560000 0.72293300

C -3.52584000 1.22037000 -1.24549800

C -3.63469400 1.36524600 0.14010100

C -4.60207500 1.48462800 -2.09282900

C -4.84714800 1.79055500 0.68149600

H -2.78989300 1.11489000 0.77172200

C -5.80959800 1.91398100 -1.53932000

H -4.49129500 1.33703300 -3.16195200

C -5.93173300 2.06818100 -0.15617700

H -4.94901300 1.88937300 1.75881200

H -6.65665300 2.11936200 -2.18797000

H -6.87659700 2.39366500 0.27079000

C 0.88254100 3.23612300 1.61815900

C 1.15423000 3.39045400 2.97956500

C -0.41963500 3.37160800 1.12491700

C 0.10927700 3.68332000 3.85743700

H 2.16671700 3.25184700 3.34721300

C -1.45818100 3.64452600 2.01556100

H -0.61776200 3.26249100 0.06291200

C -1.19758500 3.80379800 3.37918800

H 0.31697800 3.79932500 4.91727800

H -2.47095100 3.73195100 1.63330000

H -2.00968800 4.02076400 4.06726800

C 3.41541300 0.98421800 -1.86326100

C 2.50795300 0.75202900 -2.90096300

C 4.65140100 0.33648800 -1.82931700

C 2.84440200 -0.15589600 -3.90514000

H 1.55355800 1.26924100 -2.91718800

C 4.98459800 -0.55281700 -2.85076600

H 5.31574400 0.48662400 -0.98466100

C 4.08145900 -0.80500100 -3.88603900

H 2.13656900 -0.34626000 -4.70634900

H 5.94170800 -1.06571300 -2.82215300

H 4.33952300 -1.50733400 -4.67354300

**IN7**

Ni 1.32179400 -0.03092100 -1.08307300

C 1.54499200 -0.68169300 0.68484500

S -2.02669100 2.48585000 -0.80098000

O -3.24234000 3.22241700 -1.17284200

C 0.68277100 -1.68808100 0.97647000

C -0.24322100 -2.15966300 -0.14171300

H 0.27514500 -2.97550000 -0.66519200

C -1.60832300 -2.67639200 0.27640600

C -2.13931400 -3.81119400 -0.34836200

C -2.37002200 -2.02262000 1.25135000

C -3.41304900 -4.27749600 -0.01392200

H -1.55190400 -4.31939100 -1.10970700

C -3.63891700 -2.48797200 1.59517600

H -1.96793800 -1.14178700 1.74131200

C -4.16528100 -3.61580500 0.95921600

H -3.81409800 -5.15806500 -0.50983200

H -4.21850300 -1.95965700 2.34600100

H -5.15550700 -3.97735200 1.22406300

S -0.44121100 -1.28963000 -2.67982700

C -2.08833600 -0.84953600 -3.22723100

C -2.34913400 0.45487400 -3.64715900

C -3.09666700 -1.81430600 -3.14661100

C -3.65955800 0.80287400 -3.97888700

H -1.54815000 1.18148800 -3.67713200

C -4.39970800 -1.45374000 -3.48757600

H -2.86290500 -2.81995500 -2.81798000

C -4.68111800 -0.14692100 -3.89862600

H -3.87961500 1.82127500 -4.28380000

H -5.19415100 -2.19211400 -3.42702900

O 0.50602800 -0.24774700 -3.21824500

O -0.22065300 -2.70095900 -3.05287500

N -0.34366300 -0.99196600 -1.05987200

H -1.69538400 0.25887300 -0.60743100

C -1.36418000 3.23155900 0.69045300

C -1.81945900 4.48777600 1.08316600

C -0.37272000 2.55163800 1.40294000

C -1.26371800 5.08165800 2.21968000

H -2.60026300 4.97687200 0.51063500

C 0.16643400 3.15049600 2.53994500

H -0.03917000 1.56903800 1.07809100

C -0.27514500 4.41506400 2.94515100

H -1.61087500 6.06010700 2.53997100

H 0.93676200 2.63219800 3.10284300

O -0.90441200 2.39221500 -1.75255200

N -2.42069000 0.95038300 -0.34660500

C -3.81535200 0.51411100 -0.32852300

H -4.38928300 1.04716900 -1.09267400

H -3.80247400 -0.53936400 -0.62363800

C -4.50064500 0.64456500 1.01813400

C -5.85985300 0.31825000 1.11195900

C -3.81649600 1.02094800 2.17644300

C -6.51862300 0.35044200 2.34004900

H -6.40065000 0.02330800 0.21458500

C -4.47406800 1.05693500 3.40914300

H -2.76472800 1.27622200 2.11127200

C -5.82497500 0.71794400 3.49757200

H -7.57230200 0.08869100 2.39609000

H -3.92541000 1.34887500 4.30106700

H -6.33555900 0.74253200 4.45664900

C 0.63251900 -2.41966900 2.26896800

C 0.64104700 -3.82416100 2.29777400

C 0.55152200 -1.72753000 3.48820500

C 0.58847000 -4.51394400 3.50872600

H 0.69570600 -4.37660600 1.36375000

C 0.49652800 -2.41573500 4.69864000

H 0.53262200 -0.64172600 3.47449300

C 0.51691600 -3.81285800 4.71466100

H 0.60003200 -5.60098400 3.51003100

H 0.43169700 -1.86078200 5.63111300

H 0.47073400 -4.35033200 5.65812800

C 2.64078900 -0.23422000 1.57056800

C 3.50274300 -1.15293900 2.20150700

C 2.89524000 1.13489200 1.76035600

C 4.55556700 -0.71567500 3.00449000

H 3.33610700 -2.21415600 2.05505400

C 3.93933500 1.57484700 2.57279900

H 2.27443800 1.85629200 1.24384300

C 4.77722000 0.65038500 3.20162300

H 5.20400800 -1.44650600 3.48199700

H 4.09952800 2.64232400 2.70369400

H 5.59586700 0.98852400 3.83159200

H -5.69985600 0.13186200 -4.15422000

H 0.15042800 4.87671500 3.83207200

C 3.14016400 0.56192900 -1.29856700

C 5.38892700 0.28607500 -1.53113300

C 5.15326300 1.61549300 -1.42221000

H 6.30177600 -0.26679700 -1.68437900

H 5.81997800 2.46112700 -1.46825700

N 3.77570600 1.76934700 -1.28741900

N 4.15169100 -0.34292000 -1.46426000

C 3.13532500 3.01774200 -1.01338300

C 3.74170300 3.91588700 -0.12735600

C 1.89475600 3.29875700 -1.58104100

C 3.08906100 5.10528000 0.19261800

H 4.68564700 3.65904400 0.34238800

C 1.24505100 4.48645300 -1.24134300

H 1.42380500 2.58743600 -2.24837500

C 1.83683800 5.39018100 -0.35915500

H 3.55087900 5.79765400 0.89094300

H 0.26472000 4.67834700 -1.66076300

H 1.31767600 6.30477900 -0.08843100

C 3.96410600 -1.76276900 -1.43145100

C 2.99320800 -2.35113400 -2.24375200

C 4.71491900 -2.52672600 -0.53633800

C 2.73827600 -3.71735100 -2.12252700

H 2.43500500 -1.74583000 -2.94772500

C 4.47166800 -3.89780200 -0.44207900

H 5.43509400 -2.04132600 0.11419100

C 3.47597800 -4.49110900 -1.22265400

H 1.95168400 -4.15495300 -2.72789100

H 5.04392500 -4.49561400 0.26168900

H 3.27661700 -5.55509000 -1.12951000

**IN7-IPr-TPS**

Ni 2.21472600 0.17947500 0.12913000

C 1.67186000 -1.62608600 0.44005800

S -2.76987400 0.11840000 1.64045500

O -2.89236100 1.56701600 1.87058600

C 1.14101200 -2.11942700 -0.70808300

C 0.97169500 -1.08568200 -1.84021300

H 1.93030300 -0.99240700 -2.36668400

C -0.07530200 -1.39777300 -2.89539900

C 0.24167900 -1.20091700 -4.24519600

C -1.34608300 -1.88613500 -2.56660500

C -0.69095800 -1.47439600 -5.24765700

H 1.22121900 -0.80957100 -4.50396000

C -2.28022300 -2.16121500 -3.56567700

H -1.59993900 -2.05544300 -1.52549600

C -1.95690600 -1.95542000 -4.90925300

H -0.42840900 -1.31005900 -6.28973500

H -3.26353200 -2.53402000 -3.29648800

H -2.68770300 -2.16901100 -5.68481700

S 1.15560500 1.60259700 -1.79386000

C -0.22948800 2.77929700 -1.62405100

C -0.44190900 3.46610600 -0.39382800

C -1.13571000 2.92036300 -2.71254800

C -1.55700100 4.30533900 -0.31204200

C -2.20337500 3.81306300 -2.55283100

C -2.44378800 4.50277900 -1.36813800

H -1.73529100 4.82769500 0.61917400

H -2.89383500 3.94565700 -3.37873500

O 2.28391300 2.05262000 -0.87884800

O 1.54560100 1.49643800 -3.21061600

N 0.69104600 0.18909100 -1.07837900

H -1.21107900 -0.15990500 0.05194800

C -4.40524400 -0.67819600 1.66829900

C -5.60196100 0.08399300 1.76471700

C -4.43509300 -2.09355500 1.56228800

C -6.80675600 -0.62463200 1.79763600

C -5.68377300 -2.72128400 1.61290100

C -6.87654500 -2.01454300 1.72707800

H -7.73120700 -0.06493300 1.86541900

H -5.72698600 -3.80370000 1.54476800

O -1.86075700 -0.62949700 2.52161900

N -2.24193400 -0.18204700 0.06308400

C -2.77178600 0.71705700 -0.97978900

H -2.69325200 1.76324300 -0.67278300

H -2.09193500 0.57320700 -1.82273000

C -4.18741400 0.41380400 -1.42109800

C -5.01948900 1.46122800 -1.83125900

C -4.67808500 -0.89446200 -1.48111300

C -6.31773200 1.20877000 -2.28255000

H -4.64196900 2.47877000 -1.80202500

C -5.97798400 -1.15094200 -1.91104700

H -4.04081600 -1.70437800 -1.15376500

C -6.80550400 -0.09877500 -2.31380500

H -6.94981300 2.03611900 -2.59476800

H -6.34683600 -2.17318100 -1.92614000

H -7.82094700 -0.29764400 -2.64593800

C 0.90490500 -3.55345300 -1.03008200

C 1.37375000 -4.05723000 -2.26024200

C 0.27476300 -4.45906000 -0.15953700

C 1.24220200 -5.40336800 -2.59215700

H 1.86221500 -3.38667400 -2.95739100

C 0.14570400 -5.80941700 -0.48924400

H -0.11714100 -4.10883000 0.78464100

C 0.62982200 -6.29244400 -1.70531800

H 1.62002200 -5.75831000 -3.54802900

H -0.34462600 -6.48208500 0.21026400

H 0.52586300 -7.34339000 -1.96181800

C 1.77716600 -2.32077600 1.73758600

C 3.00221100 -2.67148300 2.33135300

C 0.60775800 -2.59362500 2.47297000

C 3.05995100 -3.32331600 3.56183000

H 3.92035800 -2.42487700 1.82384000

C 0.66027600 -3.23350000 3.71113500

H -0.34429400 -2.25403200 2.08756000

C 1.88610100 -3.61607200 4.25938500

H 4.02716100 -3.59397000 3.97837900

H -0.26350600 -3.42092200 4.25065900

H 1.92752900 -4.11915000 5.22181900

C 3.98190800 0.02796700 0.85925000

C 6.10521100 -0.76263100 1.10223700

C 5.85052000 0.05199900 2.15471800

H 6.98117000 -1.33201400 0.83607700

H 6.46297500 0.35508500 2.98872200

N 4.55974000 0.55006800 1.97927100

N 4.96245100 -0.76005100 0.30458600

C 4.10244200 1.73213400 2.68728700

C 3.12638400 1.63806800 3.69232000

C 4.72007400 2.95650300 2.34650900

C 2.81243100 2.80739800 4.40035300

C 4.37740700 4.08821200 3.09417900

C 3.43890300 4.01624800 4.12022300

H 2.06202400 2.76035300 5.18353600

H 4.84120100 5.04090500 2.85601600

H 3.18286800 4.90708800 4.68742500

C 4.88429000 -1.32158900 -1.03235200

C 4.84648800 -0.41360300 -2.12181900

C 4.87878800 -2.71621500 -1.22416000

C 4.62187100 -0.94363500 -3.39613000

C 4.67469500 -3.18962700 -2.52860500

C 4.50706600 -2.31833900 -3.59659400

H 4.54925800 -0.27434700 -4.24488100

H 4.63760100 -4.26055600 -2.69903200

H 4.32096900 -2.70827800 -4.59388500

C 2.38504300 0.35375700 4.01115600

H 2.77500000 -0.43674200 3.37053100

C 0.88840600 0.51279400 3.68901700

H 0.34081500 -0.39932100 3.92643200

H 0.43697100 1.33614800 4.25339700

H 0.73624800 0.71194600 2.62303200

C 2.60539600 -0.08944700 5.46831600

H 2.18980000 0.63384700 6.18029500

H 2.11465900 -1.05324800 5.63636200

H 3.67271400 -0.20654300 5.69167200

C 5.70797000 3.10261300 1.19405600

H 5.84168500 2.12883700 0.72255300

C 7.09400400 3.55564200 1.68675500

H 7.79977100 3.59454200 0.84882100

H 7.05469400 4.55369100 2.13814400

H 7.49412900 2.86489000 2.43805000

C 5.15576100 4.04786600 0.11070500

H 4.22129600 3.66017600 -0.30331400

H 4.96290800 5.04992200 0.50983900

H 5.87949000 4.14863800 -0.70645200

C 5.19706600 -3.72966200 -0.13042800

H 5.24907300 -3.20812700 0.82612900

C 4.13858300 -4.83625600 0.00718300

H 4.07774000 -5.45569900 -0.89382000

H 4.39504100 -5.49172700 0.84735800

H 3.14686100 -4.42094200 0.19031100

C 6.59615400 -4.33119200 -0.38041900

H 6.86471100 -5.02054000 0.42874000

H 6.62286800 -4.88985300 -1.32287300

H 7.36554700 -3.55136500 -0.43842900

C 5.19375600 1.06445200 -1.95106200

H 4.66162700 1.44326600 -1.07720000

C 4.79114700 1.95666300 -3.13240300

H 4.96227000 3.00371000 -2.86040400

H 5.39818600 1.74718400 -4.02241800

H 3.73641500 1.84314400 -3.38420600

C 6.71621700 1.19678000 -1.71493300

H 7.26770900 0.81098400 -2.58081800

H 6.99001500 2.24953100 -1.58287100

H 7.05382700 0.65052700 -0.82977500

C -3.21647000 -3.00899400 1.39140400

H -2.37314900 -2.41914600 1.03945400

C -5.68935200 1.61168400 1.85154400

H -4.87094400 2.03313600 1.27180800

C -8.21662300 -2.73192700 1.72395800

H -8.01292400 -3.81138500 1.71046300

C -9.03609400 -2.42696200 2.99063800

H -9.29101500 -1.36246500 3.05103800

H -9.97417600 -2.99450700 2.98859900

H -8.47706000 -2.68885100 3.89556400

C -9.01419300 -2.39036000 0.45042800

H -9.95911200 -2.94596700 0.42329600

H -9.24875800 -1.32021100 0.41017200

H -8.44066300 -2.63618600 -0.44983100

C -5.51477100 2.07391300 3.31208700

H -4.55995600 1.73912800 3.72258300

H -5.54142400 3.16898900 3.36739700

H -6.32616700 1.68053300 3.93767800

C -6.98926600 2.19241400 1.26664300

H -7.19216900 1.79644300 0.26704100

H -7.86221500 2.00134000 1.90233000

H -6.88592700 3.28072100 1.18879700

C -2.82915500 -3.62814400 2.74707800

H -2.58762400 -2.84437600 3.46943300

H -3.65113100 -4.23513400 3.14622200

H -1.95258400 -4.27644000 2.63256300

C -3.43599000 -4.11465000 0.33887600

H -2.48279700 -4.60854200 0.12670000

H -4.13665900 -4.88502500 0.67982800

H -3.81601600 -3.71161500 -0.60579200

C 0.48540200 3.45530000 0.82503700

H 1.00750000 2.50117300 0.87528900

C 1.53726200 4.56886900 0.64858700

H 1.04934900 5.55209000 0.66654100

H 2.26759200 4.53043100 1.46207400

H 2.06961300 4.46369400 -0.29822500

C -0.23548800 3.62525100 2.17390800

H 0.48825700 3.44176800 2.97359100

H -0.61617900 4.64381400 2.31827500

H -1.06590800 2.92434900 2.28526500

C -1.06557800 2.17384000 -4.05053600

H -0.51978800 1.24543600 -3.90952800

C -0.29483600 3.01349900 -5.08719700

H 0.71290000 3.24235000 -4.73423300

H -0.21158000 2.46123400 -6.03106800

H -0.82155300 3.95496200 -5.28929000

C -2.44336300 1.76949300 -4.60915900

H -3.01418700 2.62312200 -4.99292800

H -2.29000700 1.07737100 -5.44345000

H -3.05815800 1.25548700 -3.86506000

C -3.63756000 5.43308600 -1.22018300

H -4.22278300 5.36941000 -2.14811200

C -3.18076300 6.89487900 -1.05373600

H -4.04555500 7.56620900 -0.99318000

H -2.59418600 7.01780000 -0.13566000

H -2.55563700 7.21217700 -1.89554900

C -4.55358000 5.00678700 -0.05648700

H -4.90879900 3.97952800 -0.18115100

H -4.02883800 5.05708300 0.90416900

H -5.42742500 5.66649100 0.00328900

**IN7-IPr-Ts**

Ni -1.28054800 -0.32959600 -0.44658500

C -0.70309600 1.48776200 -0.68007900

S 2.05099300 -0.76955300 1.74397300

O 2.64525700 -1.98773400 2.32065600

C 0.09280900 1.64051300 -1.77406000

C 0.25774800 0.40223900 -2.66816000

H -0.59756200 0.35936200 -3.35762200

C 1.52363100 0.26792700 -3.49951400

C 1.45111400 -0.26171600 -4.79313600

C 2.78253300 0.58100400 -2.97452200

C 2.61170100 -0.47780100 -5.54095700

H 0.48269500 -0.53345800 -5.20256500

C 3.94417100 0.36964400 -3.71463500

H 2.84987200 0.99563900 -1.97856200

C 3.86184000 -0.16414200 -5.00375300

H 2.53694700 -0.89391600 -6.54249600

H 4.90729200 0.62140700 -3.27815400

H 4.76480900 -0.33164600 -5.58539600

S -0.29899000 -2.19893900 -2.13442000

C 1.02759300 -3.27121400 -1.61114400

C 1.14082200 -3.58522300 -0.25381600

C 1.97699200 -3.69682000 -2.53938000

C 2.22013700 -4.35267300 0.17049100

H 0.40743000 -3.22157400 0.45738600

C 3.05133100 -4.46760900 -2.09330200

H 1.87172300 -3.42530400 -3.58473800

C 3.18987300 -4.80320000 -0.73927400

H 2.32592500 -4.56688200 1.22958800

H 3.79703800 -4.80611000 -2.80823500

O -1.43442000 -2.45873800 -1.17409200

O -0.52942600 -2.39772900 -3.57326500

N 0.14323900 -0.69160300 -1.67150400

H 1.62328800 -0.67453800 -0.47011700

C 2.86157400 0.62683600 2.51600900

C 4.07571600 0.44627900 3.17564200

C 2.31178600 1.89773100 2.34657200

C 4.75545900 1.56477700 3.65399700

H 4.48313700 -0.55206000 3.28782200

C 3.00202000 3.00410000 2.83439500

H 1.35740200 2.01704200 1.84787100

C 4.23901900 2.85643000 3.47980000

H 5.71059700 1.43370100 4.15632400

H 2.57178000 3.99456800 2.70731400

O 0.59589100 -0.55672400 1.80147500

N 2.45599100 -0.59920100 0.14298600

C 3.71073300 -1.18776500 -0.32864800

H 3.86092800 -2.18334700 0.10110900

H 3.58581500 -1.32477800 -1.40691300

C 4.92374000 -0.31752200 -0.04993000

C 6.15506100 -0.91481300 0.24120600

C 4.84703100 1.08098400 -0.08245700

C 7.28827800 -0.13627600 0.48775000

H 6.22396600 -2.00005100 0.28602600

C 5.97492100 1.86198600 0.16510400

H 3.89014400 1.55907700 -0.25714700

C 7.20148400 1.25688200 0.45071800

H 8.23515700 -0.61809300 0.71816700

H 5.88986600 2.94550400 0.14487700

H 8.08014100 1.86505000 0.64952500

C 0.86535600 2.86027800 -2.13340900

C 0.77241900 3.43220400 -3.41095400

C 1.77100500 3.42260500 -1.21631600

C 1.54648400 4.54159500 -3.75649400

H 0.09193100 2.99856100 -4.13913100

C 2.54732400 4.52635800 -1.56022600

H 1.86399600 2.97530900 -0.23222000

C 2.43675700 5.09364900 -2.83337300

H 1.45788100 4.97062400 -4.75138100

H 3.24501700 4.93950400 -0.83553400

H 3.04411200 5.95292200 -3.10531200

C -0.98681000 2.56866200 0.28375200

C -1.13173900 3.91856500 -0.09675900

C -1.08200600 2.26967800 1.65563500

C -1.29133000 4.92232500 0.85567000

H -1.10285400 4.18047700 -1.14802400

C -1.27004400 3.27123900 2.60814400

H -0.96453700 1.23692800 1.95425300

C -1.36270900 4.60806900 2.21514200

H -1.38414500 5.95539500 0.53054000

H -1.33063800 3.00725900 3.65937900

H -1.49780400 5.39265400 2.95519500

C -2.87446900 -0.24218100 0.67082800

C -5.06564200 -0.06373900 1.27556900

C -4.46690200 -0.87035200 2.17972600

H -6.09203500 0.24076600 1.16506800

H -4.85273300 -1.37808700 3.04760600

N -3.13491300 -0.98459900 1.79728700

N -4.08367500 0.33131100 0.37080100

C -2.23218100 -1.91732500 2.44665100

C -1.51994000 -1.49768600 3.58433300

C -2.15826900 -3.23904400 1.94991500

C -0.62930500 -2.40802700 4.16434000

C -1.23530400 -4.09888200 2.55709500

C -0.46064800 -3.68180500 3.63689400

H -0.04454100 -2.10905700 5.02712500

H -1.12713200 -5.11162700 2.18495400

H 0.26133300 -4.36110100 4.08169000

C -4.43397700 1.01988600 -0.85631700

C -4.57194700 0.23547300 -2.02374800

C -4.72975900 2.40046800 -0.82766500

C -4.98928200 0.87912100 -3.19406700

C -5.16250700 2.98078000 -2.02900700

C -5.28510200 2.23737000 -3.19805300

H -5.09644000 0.30510100 -4.10791200

H -5.40073800 4.03718700 -2.05584100

H -5.61655300 2.71977000 -4.11400300

C -1.75864000 -0.14306600 4.24094400

H -2.20417600 0.52533400 3.49864700

C -0.47205500 0.51998900 4.75995000

H -0.69905100 1.51164500 5.16684700

H -0.01797600 -0.05787300 5.57256800

H 0.26616500 0.62670200 3.96375300

C -2.77596600 -0.29930600 5.39131600

H -2.38230200 -0.96947300 6.16482000

H -2.98555800 0.67211800 5.85503000

H -3.72489400 -0.71829000 5.03958800

C -3.11448600 -3.77486400 0.88768400

H -3.34752000 -2.96298100 0.19742000

C -4.43188400 -4.24075000 1.54706500

H -5.12336100 -4.61462100 0.78230600

H -4.23774800 -5.05317500 2.25769200

H -4.93666400 -3.43524900 2.08596600

C -2.54172800 -4.93573900 0.05666600

H -1.54527300 -4.70834300 -0.32239800

H -2.49992700 -5.86501600 0.63829200

H -3.19367100 -5.12030500 -0.80420900

C -4.60504000 3.27095700 0.42615000

H -3.72167800 2.93432300 0.97948900

C -4.41466000 4.76267900 0.09245800

H -5.35124200 5.21626400 -0.25531900

H -4.10333400 5.29694200 0.99428400

H -3.64825400 4.92042000 -0.66925800

C -5.83364800 3.16327400 1.35842900

H -5.76185200 3.91763800 2.15062700

H -6.75931500 3.34903600 0.79995700

H -5.91337500 2.19178700 1.84656300

C -4.36633700 -1.27501400 -2.02517600

H -3.63626200 -1.52272800 -1.25685700

C -3.78870300 -1.81617300 -3.34002500

H -3.49179200 -2.85991700 -3.20398500

H -4.51700300 -1.77771500 -4.15935500

H -2.89327200 -1.26639800 -3.64105800

C -5.68283400 -1.99671200 -1.67121400

H -6.45112300 -1.79808200 -2.42864100

H -5.51860900 -3.08013900 -1.62627200

H -6.07285400 -1.67535500 -0.69978400

C 4.37991100 -5.59243800 -0.24825100

H 5.06611700 -4.94763500 0.31691100

H 4.07244400 -6.40021600 0.42566100

H 4.94352200 -6.03432700 -1.07597900

C 5.01847200 4.06254600 3.94630200

H 4.36947600 4.93272200 4.08896700

H 5.53886000 3.86545700 4.89002500

H 5.78198600 4.33793800 3.20643500

**IN8**

Ni 0.89449400 0.38097300 -0.92370900

C 1.91082600 -0.14617900 0.58803300

S -2.27830100 0.98349600 -0.94134500

O -3.42908400 1.40291700 -1.77050500

C 1.62426200 -1.39410100 1.03281300

C 0.80537200 -2.32322600 0.14772500

H 1.50019500 -2.96120600 -0.40510700

C -0.19357000 -3.21032600 0.86753800

C -0.27307200 -4.57470400 0.57373000

C -1.08470200 -2.65833000 1.79509200

C -1.23912400 -5.37594300 1.18845200

H 0.41438400 -5.00356800 -0.15108500

C -2.05328600 -3.45243400 2.40555900

H -1.01907800 -1.59821500 2.01842200

C -2.13397900 -4.81510300 2.10124000

H -1.29224500 -6.43572400 0.95263200

H -2.74925300 -3.00702600 3.11126100

H -2.88916300 -5.43515900 2.57685600

S 0.00602900 -2.13867600 -2.48005700

C -1.72570500 -2.49662500 -2.71960200

C -2.53454400 -1.51018200 -3.28553900

C -2.22914600 -3.72973200 -2.30497300

C -3.89069200 -1.78241400 -3.45742000

H -2.11274000 -0.55071200 -3.55878500

C -3.58870100 -3.98565100 -2.48341700

H -1.57322700 -4.46413900 -1.85277000

C -4.41390600 -3.01674900 -3.06157700

H -4.53639600 -1.01847000 -3.87849900

H -4.00140600 -4.93976800 -2.16908900

O 0.37622500 -1.06442100 -3.41144800

O 0.76781800 -3.39823200 -2.48338200

N 0.10736900 -1.45818200 -0.86023600

H -0.91109500 -1.21430000 -0.56415000

C -2.20455200 2.13405600 0.45285700

C -3.23948300 3.04281800 0.65806100

C -1.18464900 1.98505900 1.39440700

C -3.24251900 3.82397900 1.81582300

H -4.03502000 3.11205900 -0.07460100

C -1.17946100 2.78421500 2.53509500

H -0.41613100 1.23269000 1.24610400

C -2.21193300 3.70340900 2.74907200

H -4.05666200 4.52237600 1.98934500

H -0.37518700 2.67948900 3.25895800

O -0.94100700 1.09262600 -1.65160600

N -2.33403300 -0.47338800 -0.32169800

C -3.65577100 -1.00556200 0.02994500

H -4.29570600 -1.07659700 -0.85834100

H -3.46951400 -2.02879200 0.37238700

C -4.39221200 -0.22975200 1.10938500

C -5.61101400 0.39594500 0.82984800

C -3.83999900 -0.07845300 2.38873200

C -6.27220200 1.14691700 1.80581800

H -6.03154600 0.31038600 -0.16896200

C -4.49074800 0.67355500 3.36426100

H -2.87632600 -0.52819400 2.60500200

C -5.71366200 1.28814500 3.07636800

H -7.21764200 1.62885200 1.56867200

H -4.04001900 0.79093700 4.34647300

H -6.22059100 1.87847500 3.83545600

C 2.09159200 -1.96961600 2.32421700

C 2.70425800 -3.23288100 2.36658900

C 1.90046100 -1.28126300 3.53309700

C 3.12983400 -3.78119400 3.57647400

H 2.85588900 -3.78695100 1.44403900

C 2.32285900 -1.82864400 4.74282900

H 1.42041800 -0.30767200 3.51245600

C 2.94149000 -3.08126300 4.77010200

H 3.60702200 -4.75785700 3.58642500

H 2.16425300 -1.27847600 5.66679700

H 3.26895100 -3.51006100 5.71346600

C 2.81509400 0.77304100 1.31842600

C 4.10244300 0.36408400 1.71190000

C 2.42360300 2.08883700 1.62705600

C 4.96007500 1.23252800 2.38670700

H 4.42230400 -0.64610400 1.48151800

C 3.27518600 2.95506900 2.31083100

H 1.44252500 2.43157400 1.32056100

C 4.55207000 2.53335800 2.69189300

H 5.94956800 0.88906200 2.67878300

H 2.93918200 3.96217600 2.54825300

H 5.21953000 3.20892400 3.22038700

H -5.47312000 -3.22064400 -3.19316300

H -2.21851500 4.31315000 3.64885400

C 2.08405300 1.80443300 -1.40825800

C 4.06023300 2.78653000 -1.98059000

C 3.15028100 3.77432200 -1.81119800

H 5.09505200 2.81315200 -2.28116500

H 3.22244200 4.84408100 -1.92338300

N 1.94844000 3.15823000 -1.47781900

N 3.39651900 1.58880700 -1.74606400

C 0.75181300 3.91561700 -1.23960900

C 0.70854400 4.77917100 -0.14438100

C -0.31404700 3.82276800 -2.13271500

C -0.42810800 5.56087300 0.06148200

H 1.55257700 4.82096400 0.53730700

C -1.44751900 4.60438400 -1.91592500

H -0.26265200 3.12492100 -2.95839500

C -1.50337600 5.47364800 -0.82439400

H -0.47986900 6.22054300 0.92219800

H -2.29515300 4.51034800 -2.58726300

H -2.39475300 6.06942000 -0.65195700

C 4.03800100 0.31065600 -1.82089400

C 3.40887200 -0.73770000 -2.49294500

C 5.28014000 0.12853300 -1.21007300

C 4.00179000 -1.99979600 -2.50255700

H 2.46244100 -0.57090000 -2.99122800

C 5.88156000 -1.13045300 -1.25202900

H 5.74076000 0.94529700 -0.66461700

C 5.23808600 -2.19860000 -1.88207200

H 3.48449900 -2.81854100 -2.99323300

H 6.84313000 -1.27897100 -0.76866300

H 5.70199700 -3.18102800 -1.89336500

**IN9**

Ni -1.04504200 -0.28032800 0.23892200

C 0.11046900 -1.69541500 -0.28452400

S -1.69668900 2.38085200 1.25187200

O -0.21069600 2.52609400 1.31195400

C 1.45929700 -1.68060700 -0.31503400

C 2.26084700 -0.43194400 0.13092200

H 2.22511600 -0.40677200 1.22125500

C 3.74482000 -0.42241400 -0.23506600

C 4.66212900 0.05637500 0.70785900

C 4.22607700 -0.84613500 -1.48075100

C 6.02742900 0.10413400 0.42615800

H 4.29794000 0.40974600 1.66715200

C 5.59078500 -0.80743700 -1.76405900

H 3.53075900 -1.20160200 -2.22941400

C 6.49743400 -0.33555700 -0.81191200

H 6.71996100 0.48785700 1.17074300

H 5.94499700 -1.14857000 -2.73322100

H 7.56086700 -0.30735500 -1.03557600

S 2.00449500 1.84445500 -1.49601000

C 3.49028600 2.70082600 -0.96764700

C 4.66790400 2.57923000 -1.69801100

C 3.41420900 3.48386700 0.18854700

C 5.80805700 3.24975300 -1.24987500

H 4.68943700 1.94706200 -2.57755700

C 4.55940100 4.14478500 0.62587800

H 2.47530800 3.56985400 0.72927700

C 5.75552500 4.02649000 -0.09185600

H 6.73856700 3.15293700 -1.80162700

H 4.52003200 4.75547100 1.52371000

O 2.30759100 1.03438000 -2.67881600

O 0.95542600 2.87427700 -1.55669000

N 1.57160000 0.83644900 -0.24924400

H 1.09073000 1.35660900 0.49035200

C -2.31708200 3.95209100 0.64890400

C -1.50266900 4.74150300 -0.16678300

C -3.62343100 4.32665800 0.96953000

C -2.02242000 5.93336600 -0.67617500

H -0.49112200 4.42329200 -0.39645800

C -4.12545100 5.52324300 0.45947700

H -4.21875700 3.69359000 1.61918200

C -3.32720800 6.32299100 -0.36473800

H -1.40390500 6.55736800 -1.31507500

H -5.13730200 5.83317600 0.70568700

O -2.37353300 2.05417600 2.52318300

N -2.20858400 1.26668700 0.15479800

C -1.62644700 1.33776600 -1.17607300

H -1.18415800 2.30339000 -1.43276200

H -0.66997200 0.69426100 -1.22493000

C -2.51046100 0.80454500 -2.28076800

C -1.94737800 0.65148200 -3.55643000

C -3.82551900 0.40080300 -2.05503900

C -2.69568600 0.09464200 -4.59221300

H -0.91513900 0.95316200 -3.72557800

C -4.57392200 -0.15850000 -3.09384300

H -4.24282800 0.50992200 -1.06298400

C -4.01345800 -0.31638100 -4.36181100

H -2.25032400 -0.02471100 -5.57645800

H -5.59371000 -0.48028300 -2.90104600

H -4.59580300 -0.75607900 -5.16758000

C 2.22696500 -2.92700300 -0.62146300

C 3.14621600 -3.44320500 0.30770700

C 2.03231800 -3.63002000 -1.82065100

C 3.84065200 -4.62474700 0.05092000

H 3.32345200 -2.90398700 1.23357200

C 2.72637100 -4.81172800 -2.07977500

H 1.32886700 -3.24055300 -2.54956000

C 3.63331500 -5.31608100 -1.14504900

H 4.54776700 -5.00433500 0.78472600

H 2.55970100 -5.33763100 -3.01650300

H 4.17632500 -6.23546400 -1.34813500

C -0.71577800 -2.86741600 -0.66067000

C -1.54229400 -2.80106400 -1.79849200

C -0.76762800 -4.02979200 0.12924100

C -2.36358500 -3.87120900 -2.15229900

H -1.53581100 -1.89914600 -2.40420900

C -1.59739800 -5.09610800 -0.21803700

H -0.13248100 -4.09516200 1.00837500

C -2.39564900 -5.02609100 -1.36489300

H -2.98686200 -3.79334600 -3.03946400

H -1.61358200 -5.98943200 0.40202900

H -3.03642400 -5.86048000 -1.63814700

H 6.64688000 4.54194500 0.25559300

H -3.72393000 7.25311400 -0.76279700

C -1.51918400 -1.15237100 1.81997200

C -1.50440000 -2.30892600 3.77894700

C -2.75009100 -2.40591700 3.25969900

H -1.07620600 -2.67811600 4.69685100

H -3.64103200 -2.89697500 3.61640200

N -2.74379700 -1.68627900 2.06872000

N -0.76322700 -1.53294300 2.88881500

C -3.93750500 -1.48034100 1.29262700

C -4.35738700 -2.46961800 0.40739800

C -4.67996800 -0.31856900 1.50878400

C -5.56683000 -2.29759200 -0.26861700

H -3.74096800 -3.34689100 0.24708800

C -5.89293700 -0.16567100 0.83670300

H -4.28610000 0.45053200 2.16507900

C -6.33950200 -1.15609300 -0.04454500

H -5.89848100 -3.05661500 -0.97099200

H -6.48289600 0.73313900 0.99166200

H -7.28426600 -1.02983900 -0.56660200

C 0.55764100 -1.07408800 3.18952400

C 0.77319300 0.29380700 3.36520700

C 1.58434200 -2.00183600 3.37274100

C 2.04683300 0.73612100 3.72738600

H -0.04269200 0.99091800 3.21934300

C 2.84938900 -1.54950000 3.75001500

H 1.39459000 -3.05655400 3.20014700

C 3.07964200 -0.18246200 3.93288800

H 2.22249000 1.80021700 3.85332300

H 3.65663400 -2.26269400 3.88819100

H 4.06668900 0.16465200 4.22505400

**IN10**

Ni 0.94721300 0.13517700 -0.02215000

C -0.00004100 1.15466000 1.55501800

S 3.69287800 -1.26869700 0.03585900

O 4.27346900 -0.30075700 0.98581300

C -0.69903500 1.39250600 0.35661900

C -1.99643100 0.51362100 0.30318900

H -1.73960500 -0.45521000 0.74214800

C -3.05499400 1.13839800 1.19969300

C -3.31962700 0.52325700 2.43113300

C -3.70971600 2.33639900 0.88363900

C -4.22459300 1.08803700 3.33096200

H -2.82887800 -0.41469600 2.67787900

C -4.61864600 2.89693200 1.78050900

H -3.53227600 2.80822500 -0.07375700

C -4.87869400 2.27794900 3.00636600

H -4.42192600 0.59390500 4.27890200

H -5.12898200 3.81908500 1.51641600

H -5.58850200 2.71954800 3.70113500

S -3.97622400 -0.14045800 -1.58635300

C -4.55432100 -1.48896100 -0.54605200

C -5.34547800 -1.20205200 0.56831400

C -4.21352200 -2.80056400 -0.88323300

C -5.78792700 -2.25554800 1.36901500

H -5.60712600 -0.17515100 0.79447600

C -4.67803100 -3.84547700 -0.08474800

H -3.60480700 -2.99388600 -1.75934200

C -5.45722300 -3.57341300 1.04343400

H -6.39732300 -2.04392600 2.24279900

H -4.43049000 -4.87161700 -0.34264900

O -4.86810000 0.99382700 -1.35828600

O -3.78360900 -0.67766800 -2.94101400

N -2.41988900 0.27595600 -1.09406800

C 4.26181700 -2.90700900 0.50837900

C 4.60335700 -3.15097600 1.83891900

C 4.30858600 -3.91509400 -0.45625100

C 4.99078700 -4.43923800 2.21070900

H 4.57791400 -2.33891900 2.55804400

C 4.69951700 -5.19798600 -0.07333000

H 4.06056600 -3.68412600 -1.48694400

C 5.03499000 -5.46067500 1.25825800

H 5.26248100 -4.64355500 3.24251900

H 4.74688700 -5.99135300 -0.81416800

O 3.95665900 -1.10687900 -1.40700900

N 2.04363900 -1.44879600 0.19915200

C 1.48069500 -1.18520100 1.42260600

H 2.07188600 -0.68597100 2.19338700

H -0.46409200 0.40176900 2.18413300

C 0.33634800 -1.99577700 1.87221900

C -0.11510800 -1.89480600 3.19975600

C -0.37701100 -2.81865900 0.98033300

C -1.26980800 -2.56151100 3.61432400

H 0.44172600 -1.28139900 3.90367000

C -1.52360900 -3.48562600 1.39886700

H -0.02762000 -2.90667600 -0.04174200

C -1.98765000 -3.35010600 2.71204100

H -1.60826500 -2.46244800 4.64267700

H -2.06895000 -4.10334900 0.69352200

H -2.89799700 -3.85440700 3.02316200

C -0.79769300 2.71273000 -0.34480100

C -1.03033700 2.80114400 -1.72886700

C -0.82690000 3.90505500 0.39951200

C -1.28746800 4.02771500 -2.34072300

H -1.02660400 1.90329800 -2.32991300

C -1.05406300 5.13677100 -0.21437300

H -0.69078500 3.86368700 1.47361100

C -1.29382600 5.20506900 -1.58808100

H -1.47970900 4.06041900 -3.41000200

H -1.06845800 6.04052200 0.38926500

H -1.49100500 6.16146700 -2.06532800

C 0.95796600 1.96463500 2.33259300

C 1.86918200 2.88223700 1.78502800

C 0.96848800 1.77206900 3.72830900

C 2.74996900 3.58269900 2.60550200

H 1.88286300 3.04163900 0.71681700

C 1.84915400 2.47396500 4.54886700

H 0.26142800 1.07459200 4.17013400

C 2.74760100 3.38378000 3.98848200

H 3.44543600 4.28286900 2.15500900

H 1.83214800 2.31075000 5.62340700

H 3.43986000 3.93191600 4.62200300

H -5.81431700 -4.39089600 1.66410100

H 5.33778600 -6.46192800 1.55234800

C 1.25634800 0.58138300 -1.90997200

C 1.73662100 1.72201500 -3.83242200

C 1.07416200 0.60010400 -4.19313200

H 2.16267400 2.52257400 -4.41539200

H 0.79597800 0.20749100 -5.15791400

N 0.80196100 -0.09182300 -3.01381500

N 1.85056800 1.68973800 -2.44552900

C 0.23235400 -1.40631300 -3.01979100

C -1.03129600 -1.60258600 -3.57652300

C 0.98056800 -2.47928500 -2.52833300

C -1.57198800 -2.89081200 -3.60919100

H -1.60558300 -0.76188000 -3.94822700

C 0.42919800 -3.75925500 -2.56023400

H 1.96356800 -2.28867500 -2.12149500

C -0.84660200 -3.96785000 -3.09660100

H -2.56522600 -3.03168400 -4.02338600

H 1.00106800 -4.59420900 -2.16484900

H -1.26955600 -4.96863300 -3.11916400

C 2.62461300 2.66693400 -1.73529700

C 3.80276400 2.26730100 -1.10370700

C 2.22562700 4.00492200 -1.74760100

C 4.58514300 3.22575400 -0.45993100

H 4.09712800 1.22705200 -1.12498400

C 3.02175800 4.95604800 -1.10862600

H 1.29344000 4.28648700 -2.22185400

C 4.20247400 4.56928900 -0.46864100

H 5.49202100 2.91279000 0.04818600

H 2.70897100 5.99618000 -1.10271400

H 4.81849400 5.31371200 0.02899100

H -1.75668800 -0.32915800 -1.57583600

**TS1**

Ni 0.45699700 0.36084800 -0.11845200

C -0.01212000 -1.44039300 0.60148400

S -2.23736300 0.93828000 -1.77560500

O -1.21545900 0.38324200 -2.68147700

C 1.26414800 -1.13069400 0.58492600

C -3.70405300 -0.08951400 -1.94341200

C -3.55731200 -1.41524000 -2.36003900

C -4.95154200 0.42315400 -1.58044600

C -4.67956600 -2.24289400 -2.39864300

H -2.57715300 -1.77813900 -2.65115200

C -6.06778600 -0.41495000 -1.62036000

H -5.03907800 1.46550300 -1.29285000

C -5.93154000 -1.74572800 -2.02306300

H -4.57674600 -3.27445200 -2.72349600

H -7.04338900 -0.02665500 -1.34142500

O -2.67615300 2.33998700 -1.93637100

N -1.69769500 0.68627700 -0.24437300

C -2.51247200 1.25751800 0.85129700

H -2.59195100 2.34071000 0.71410700

H -1.01420000 -0.43774800 0.08379100

C -1.87008700 0.93982700 2.18290600

C -2.28969500 -0.16163300 2.93463800

C -0.79537100 1.70958700 2.64716700

C -1.63644100 -0.50382400 4.12022600

H -3.11050200 -0.77565100 2.57489500

C -0.14431800 1.37433100 3.83329800

H -0.46445400 2.56398100 2.06965200

C -0.55934400 0.26149000 4.57028300

H -1.96140000 -1.37616600 4.68043300

H 0.68911400 1.98064800 4.17916100

H -0.04712600 -0.00578600 5.49101500

C 2.59364600 -1.63148700 0.85705200

C 3.60034100 -0.75365800 1.29995300

C 2.92315400 -2.98404300 0.64574800

C 4.89314900 -1.21467900 1.53647700

H 3.34384800 0.29217600 1.44274100

C 4.22065100 -3.43930900 0.87023300

H 2.15395600 -3.66241000 0.28939000

C 5.21039700 -2.55894100 1.31747800

H 5.65785200 -0.52495800 1.88559000

H 4.46225100 -4.48427300 0.69269900

H 6.22110900 -2.91778100 1.49367900

C -0.83756700 -2.53457100 1.10757800

C -2.16387700 -2.70180100 0.67004200

C -0.33440100 -3.43538000 2.06720600

C -2.95876800 -3.73508600 1.16352400

H -2.57387000 -2.01355900 -0.06047400

C -1.13169400 -4.46293400 2.56423600

H 0.68257100 -3.30766300 2.42534200

C -2.44828900 -4.62005500 2.11589500

H -3.97859600 -3.84315900 0.80205300

H -0.72681000 -5.14380300 3.30905900

H -3.06691600 -5.42384200 2.50624800

H -6.80275400 -2.39443800 -2.05287700

C 1.80479000 1.51849300 -0.76279200

C 3.75873700 2.21461700 -1.72114600

C 3.22192600 3.28388200 -1.08638500

H 4.64880400 2.11625600 -2.32158700

H 3.54489900 4.31021200 -1.01418500

N 2.02741400 2.84646000 -0.51834400

N 2.88213400 1.15049900 -1.52737000

C 1.09526700 3.70590400 0.14235400

C 1.48712500 4.42347100 1.27429700

C -0.19717100 3.81978200 -0.37186500

C 0.56478100 5.26328000 1.90178900

H 2.49375600 4.30557700 1.66487200

C -1.11410200 4.65380100 0.26737200

H -0.48924600 3.25359300 -1.24722100

C -0.73582100 5.37680800 1.40194600

H 0.85965100 5.81761700 2.78816100

H -2.12070600 4.72599200 -0.13285500

H -1.45230200 6.02415200 1.89952000

C 3.07494000 -0.15021400 -2.09104900

C 2.00510800 -0.79388700 -2.71791600

C 4.32787500 -0.75854200 -1.99511600

C 2.20044500 -2.07422300 -3.23641700

H 1.03808400 -0.30562900 -2.78085600

C 4.51286800 -2.03273200 -2.52952800

H 5.13094400 -0.26126900 -1.46122700

C 3.44882200 -2.69452400 -3.14626000

H 1.36959200 -2.58288500 -3.71689200

H 5.48070800 -2.51632800 -2.43707000

H 3.59167000 -3.69245100 -3.55164400

H -3.52879300 0.83789000 0.82333300

**TS2**

Ni -0.07036800 -0.18297200 -0.23316900

C -1.84699500 0.45464500 -0.61857900

S 2.37516000 -1.94005400 -0.43017900

O 1.75954200 -2.78245700 -1.48027600

C -2.84164300 -0.25662900 -1.19080000

C 4.10818800 -1.72704800 -0.85542000

C 4.66831200 -2.52076500 -1.85502900

C 4.86600700 -0.78120800 -0.15832300

C 6.02246800 -2.36495300 -2.16147700

H 4.04566300 -3.23810900 -2.37888100

C 6.21443700 -0.63275400 -0.47653600

H 4.39648700 -0.16605900 0.60213500

C 6.79261000 -1.42435100 -1.47489700

H 6.47241700 -2.97614400 -2.93883200

H 6.81507000 0.10318500 0.05098100

O 2.29062200 -2.38722900 0.97005500

N 1.84163300 -0.36238100 -0.46018400

C 1.35468000 0.15618200 -1.65034000

H 1.31176500 -0.49605500 -2.52704200

H -0.38502400 0.31350000 -1.61974100

C 1.63144000 1.59765100 -1.91692300

C 1.24965400 2.16713300 -3.13932400

C 2.21589000 2.40828700 -0.93549400

C 1.43517300 3.52951800 -3.37218900

H 0.78624700 1.54254700 -3.90039400

C 2.39869300 3.77027200 -1.16897000

H 2.50184000 1.96222100 0.00909100

C 2.00641500 4.33779000 -2.38408100

H 1.13133300 3.96141700 -4.32197300

H 2.84315200 4.38956600 -0.39407600

H 2.14641300 5.40047000 -2.56344500

C -4.26796000 0.08526300 -1.34127500

C -5.19324400 -0.96620700 -1.49230700

C -4.76380000 1.40394200 -1.37161000

C -6.55737000 -0.71700100 -1.63279700

H -4.82763400 -1.98930000 -1.48650500

C -6.12720200 1.65232900 -1.51598700

H -4.07461400 2.23665800 -1.29319100

C -7.03409200 0.59577600 -1.64083600

H -7.24902200 -1.54934300 -1.74039300

H -6.48319200 2.67947900 -1.53864700

H -8.09675200 0.79446200 -1.75336400

C -1.98618600 1.75526700 0.07157100

C -1.29121700 2.90044500 -0.35411400

C -2.75886100 1.84277300 1.24663300

C -1.37145700 4.09412700 0.36480400

H -0.67801100 2.84967200 -1.24751100

C -2.83901400 3.03561500 1.96310100

H -3.29629200 0.96045100 1.58269400

C -2.14241900 4.16923000 1.52749300

H -0.82011400 4.96294100 0.01437200

H -3.44267900 3.08180500 2.86625200

H -2.20033700 5.09724400 2.09007600

H 7.84438700 -1.30316000 -1.71965300

C -0.37385700 -0.85455300 1.52110900

C -1.03503900 -2.06652500 3.32830600

C -0.15758800 -1.13170000 3.76730500

H -1.55932500 -2.85477200 3.84422400

H 0.22352800 -0.91384600 4.75219200

N 0.22880600 -0.39729000 2.65021900

N -1.15678900 -1.88132900 1.95148100

C 1.18694000 0.67069500 2.66648800

C 0.75153000 1.98395200 2.49923300

C 2.53867700 0.36513100 2.83067700

C 1.69096800 3.01655400 2.51281800

H -0.30177600 2.18713100 2.34767900

C 3.46821400 1.40582500 2.84977200

H 2.84274400 -0.67445800 2.88954100

C 3.04584300 2.73086200 2.69475400

H 1.35544000 4.03934300 2.37043100

H 4.52393300 1.18091600 2.97585400

H 3.77487200 3.53670700 2.70601400

C -1.94133800 -2.71409800 1.09414900

C -1.35255500 -3.28888700 -0.03434400

C -3.28161100 -2.94683200 1.40646900

C -2.13014500 -4.09508100 -0.86582800

H -0.30723100 -3.10928000 -0.25680200

C -4.04447700 -3.76970600 0.57741100

H -3.72412000 -2.46776300 2.27491200

C -3.47165900 -4.34233400 -0.56127500

H -1.67582100 -4.53839900 -1.74709100

H -5.09035900 -3.94530000 0.81181800

H -4.06966000 -4.97712400 -1.20926500

H -2.58093500 -1.24385600 -1.56872700

**TS3**

S 1.78585800 -0.52569300 -2.10184100

O 1.63659800 -1.90513500 -2.60516500

O 1.05886600 0.55523200 -2.81712900

Ni -0.39844400 -0.62455000 -0.06493500

C -0.15250800 1.42826800 0.58057600

C 1.37952800 0.71581900 0.18060100

C 2.14107600 0.66143200 1.49098600

C 2.98344900 1.71178700 1.86639600

C 2.02093600 -0.45287500 2.33290900

C 3.69711000 1.65222000 3.06648900

H 3.07780700 2.58076700 1.22127300

C 2.73204400 -0.51281300 3.52923000

H 1.38125600 -1.27749500 2.03332300

C 3.57302000 0.54150900 3.90154500

H 4.35032300 2.47519500 3.34470800

H 2.63278600 -1.38654700 4.16848600

H 4.12869200 0.49431300 4.83456100

H 1.75547000 1.51372900 -0.46166000

C -1.31104300 0.77198600 0.63861600

N 1.47491600 -0.58587800 -0.49675800

C -2.68309200 1.13380800 0.95941900

C -3.22624900 2.37607200 0.58350800

C -3.51432400 0.20300300 1.60802100

C -4.56046500 2.67367900 0.85278900

H -2.59780400 3.09312500 0.06628700

C -4.84428500 0.50921400 1.88834900

H -3.09926300 -0.76417300 1.87753400

C -5.37445500 1.74524400 1.50744700

H -4.96771600 3.63381600 0.54661300

H -5.47065700 -0.21946300 2.39707100

H -6.41448100 1.98175900 1.71646500

C 0.06684300 2.85209000 0.92703900

C 0.52495200 3.75613300 -0.04478100

C -0.19789800 3.31660800 2.22322400

C 0.71482800 5.10041500 0.27830400

H 0.71291400 3.40215900 -1.05555600

C -0.00512400 4.66013300 2.54313000

H -0.54610000 2.61318600 2.97307100

C 0.45362600 5.55529700 1.57335300

H 1.06323400 5.79283000 -0.48357000

H -0.20735600 5.00710000 3.55302600

H 0.60588800 6.60150200 1.82485600

C -1.64793700 -1.93251100 -0.36615200

C -2.75191000 -3.91651400 -0.28922700

C -3.49586800 -3.06576800 -1.03868100

H -2.89533400 -4.95348600 -0.03081500

H -4.41949200 -3.21133400 -1.57524800

N -2.80529700 -1.85677600 -1.08450200

N -1.61830700 -3.21155100 0.10667200

C 3.52863300 -0.06048300 -2.16457500

C 4.39855800 -0.49465900 -1.16061500

C 3.98484200 0.71579400 -3.23155100

C 5.74718200 -0.14485900 -1.23271700

H 4.00929500 -1.07038100 -0.32762300

C 5.33706500 1.05531000 -3.29694200

H 3.27965100 1.05518500 -3.98294400

C 6.21702300 0.62548700 -2.30015000

H 6.42925500 -0.46801300 -0.45103500

H 5.70175400 1.66128500 -4.12199200

H 7.26806700 0.89746200 -2.35096000

C -0.54198000 -3.73710800 0.89327200

C -0.81541400 -4.30435000 2.13932900

C 0.76470200 -3.64301300 0.40638300

C 0.24075700 -4.78032400 2.91768900

H -1.83959400 -4.34932000 2.49880600

C 1.81206000 -4.10589400 1.20363000

H 0.95770100 -3.18919200 -0.56044800

C 1.55484800 -4.67426600 2.45404200

H 0.03668000 -5.21881500 3.89034200

H 2.83110600 -4.01732500 0.83960500

H 2.37641700 -5.03380900 3.06736000

C -3.24459100 -0.68926500 -1.78722000

C -2.33402900 0.03151400 -2.56342600

C -4.57132000 -0.27403900 -1.65761800

C -2.76287800 1.19660200 -3.19900000

H -1.30350000 -0.29231500 -2.66050000

C -4.99128700 0.88251200 -2.31246000

H -5.24871500 -0.82005200 -1.00923500

C -4.08759500 1.62229100 -3.07937900

H -2.04908100 1.76277800 -3.78968700

H -6.01738500 1.21886600 -2.19762600

H -4.41497500 2.53090600 -3.57706300

**TS4**

Ni 0.92199600 0.34300000 -0.95156200

C 1.87877400 -0.20349700 0.58906200

S -2.25509100 1.07175800 -0.90996800

O -3.44574100 1.45301200 -1.69394600

C 1.54327700 -1.43910600 1.03174800

C 0.67405300 -2.32233900 0.15104300

H 1.33616700 -3.00138400 -0.39494700

C -0.36188600 -3.15923700 0.88201400

C -0.59569500 -4.48297100 0.49562700

C -1.12663400 -2.60872200 1.91667900

C -1.58974800 -5.24012700 1.12078000

H -0.00038100 -4.91254000 -0.30619000

C -2.11875200 -3.36055200 2.54401200

H -0.93817800 -1.58405500 2.22133000

C -2.35611200 -4.67907800 2.14389900

H -1.76253700 -6.26746300 0.81019900

H -2.71284800 -2.91549800 3.33746600

H -3.13033800 -5.26544000 2.63148500

S -0.06178400 -2.06662600 -2.45729400

C -1.79934600 -2.35163100 -2.77695300

C -2.55557600 -1.31107900 -3.31811200

C -2.36324900 -3.58582600 -2.45172000

C -3.91642400 -1.51855100 -3.53956800

H -2.08880500 -0.35859000 -3.53649700

C -3.72490400 -3.78080700 -2.68424000

H -1.74852000 -4.37250000 -2.03145800

C -4.49810300 -2.75050500 -3.22700300

H -4.51897900 -0.70806100 -3.93696000

H -4.18021300 -4.73610900 -2.43959600

O 0.36600000 -0.98311800 -3.36195600

O 0.64106700 -3.36240700 -2.49926700

N 0.01817600 -1.42645300 -0.85605800

H -1.10895700 -1.05361700 -0.53753700

C -2.17155300 2.19584600 0.49749700

C -3.18842000 3.12175100 0.71174400

C -1.13886300 2.02723600 1.42125400

C -3.16159500 3.90254200 1.86950400

H -3.99345500 3.20212600 -0.00947700

C -1.10727300 2.82411100 2.56278100

H -0.38536600 1.26318100 1.25572300

C -2.12141700 3.76105100 2.78932200

H -3.96010700 4.61580900 2.05426500

H -0.29626300 2.70528200 3.27668700

O -0.94066800 1.19072500 -1.63926300

N -2.27469800 -0.40273000 -0.29030300

C -3.57608600 -0.98976400 0.05284600

H -4.19835400 -1.10083200 -0.84279500

H -3.34809200 -1.99546100 0.41748600

C -4.34474400 -0.21225100 1.10708700

C -5.56146500 0.39978300 0.79119500

C -3.81951100 -0.03724100 2.39474500

C -6.24753200 1.16167500 1.74107900

H -5.95885300 0.29703800 -0.21519500

C -4.49594200 0.72543900 3.34420400

H -2.85797500 -0.47680900 2.63721200

C -5.71633800 1.32689400 3.02053900

H -7.19076100 1.63337700 1.47660700

H -4.06680400 0.86166000 4.33358000

H -6.24293700 1.92589800 3.75914800

C 2.00983700 -2.04002600 2.31239800

C 2.56825000 -3.32868400 2.33684400

C 1.87378300 -1.35057300 3.52826300

C 2.99476900 -3.90080800 3.53539200

H 2.67557900 -3.88397000 1.40890700

C 2.29649200 -1.92178400 4.72675900

H 1.43633600 -0.35683300 3.52197200

C 2.86128100 -3.19981900 4.73581900

H 3.42935200 -4.89722100 3.53127900

H 2.18085000 -1.37002700 5.65619800

H 3.18936000 -3.64721000 5.67032000

C 2.82298200 0.67827200 1.31469400

C 4.09900500 0.22290800 1.69348200

C 2.47917100 2.00388100 1.63669800

C 4.99111700 1.05679500 2.36730100

H 4.38203400 -0.79595500 1.45378700

C 3.36512300 2.83584600 2.31936900

H 1.50776100 2.38095500 1.34033300

C 4.63011500 2.36825000 2.68606800

H 5.97088100 0.67781300 2.64808600

H 3.06563100 3.85179000 2.56808900

H 5.32448800 3.01658200 3.21401600

H -5.55968700 -2.90618500 -3.39934500

H -2.10575100 4.37008400 3.68942800

C 2.17221800 1.72488800 -1.41577500

C 4.18582800 2.64243100 -1.96499600

C 3.31116500 3.66059400 -1.78970000

H 5.22275900 2.63575400 -2.25924700

H 3.42320800 4.72823200 -1.88892700

N 2.08610400 3.08430300 -1.47043600

N 3.47849000 1.46661300 -1.74726400

C 0.91781700 3.87985200 -1.21630900

C 0.90169300 4.71636200 -0.09920700

C -0.14900100 3.84934600 -2.11229000

C -0.21141100 5.52447700 0.13149800

H 1.74764100 4.71617800 0.58097400

C -1.25834500 4.65867000 -1.87228500

H -0.11760900 3.17619500 -2.95940300

C -1.29022200 5.49426400 -0.75388600

H -0.24200000 6.16115200 1.01034800

H -2.10620800 4.61498200 -2.54877400

H -2.16380900 6.11019600 -0.56257700

C 4.07834800 0.16767000 -1.81724000

C 3.41174600 -0.86523300 -2.47643400

C 5.31628600 -0.05082200 -1.20907100

C 3.96125400 -2.14686100 -2.47535400

H 2.47078800 -0.67199100 -2.97507800

C 5.87515000 -1.32942500 -1.24229300

H 5.80626200 0.75374800 -0.67104700

C 5.19299600 -2.38148900 -1.85883500

H 3.41203700 -2.95156300 -2.95444600

H 6.83341700 -1.50563300 -0.76162600

H 5.62326300 -3.37918000 -1.86184500

**TS5**

Ni -1.21087500 -0.23635800 0.29659800

C 0.01374600 -1.53638300 -0.44166100

S -1.67240800 2.44814100 1.35192500

O -0.18368100 2.47439700 1.42558100

C 1.35779200 -1.63037400 -0.34395500

C 2.22426500 -0.48247800 0.22702400

H 2.12464300 -0.53270800 1.31473500

C 3.72466600 -0.56429100 -0.06229700

C 4.61386500 -0.12778100 0.92712100

C 4.24602100 -0.99873800 -1.28705500

C 5.99152800 -0.13469200 0.71089000

H 4.21641200 0.23451600 1.87040200

C 5.62384300 -1.01778100 -1.50339900

H 3.57326400 -1.31377100 -2.07355600

C 6.50202300 -0.58837100 -0.50648200

H 6.66318100 0.21639400 1.48993100

H 6.00991900 -1.36658000 -2.45764600

H 7.57517100 -0.60208000 -0.67892500

S 2.14352800 1.79176600 -1.38124300

C 3.71488900 2.54896300 -0.95831100

C 4.83441900 2.33130200 -1.75506500

C 3.76467000 3.36176000 0.17799300

C 6.04237400 2.93348800 -1.39540900

H 4.75743700 1.68341000 -2.61975400

C 4.97418100 3.95791300 0.52477400

H 2.87077800 3.51680900 0.77536100

C 6.11288400 3.74136600 -0.26017000

H 6.92836400 2.76131800 -1.99964800

H 5.03109600 4.59101400 1.40590600

O 2.33647600 0.93888000 -2.55932400

O 1.16736100 2.89688100 -1.42639700

N 1.71181200 0.86848600 -0.08835700

H 1.07397600 1.34040800 0.55756900

C -2.17441900 4.06872200 0.77456300

C -1.32999900 4.77382400 -0.08710800

C -3.41862100 4.56682900 1.16348800

C -1.75815100 6.01053100 -0.57213100

H -0.36577400 4.36320400 -0.36890900

C -3.82883900 5.80693300 0.67403800

H -4.03659000 3.99286700 1.84591400

C -3.00154300 6.52493100 -0.19436600

H -1.11613400 6.57259000 -1.24439300

H -4.79180900 6.21284900 0.97122900

O -2.40710600 2.13277700 2.58952900

N -2.24609000 1.41266300 0.17933800

C -1.49360800 1.31799200 -0.99432000

H -0.66622100 2.00972000 -1.14180800

H -0.45142300 0.05278200 -0.98747800

C -2.20958100 0.93550000 -2.24417500

C -1.50587100 0.98269900 -3.45720600

C -3.52397200 0.45628600 -2.22342000

C -2.11202200 0.54363500 -4.63388500

H -0.47710000 1.33575500 -3.46607700

C -4.12592600 0.01782900 -3.40186900

H -4.05274700 0.41547000 -1.28003900

C -3.42309800 0.05622300 -4.60910800

H -1.55983500 0.57726800 -5.56930200

H -5.14345600 -0.36287000 -3.37122700

H -3.89226200 -0.29051200 -5.52631400

C 2.04285900 -2.93294600 -0.60323400

C 2.83373300 -3.53357600 0.39054000

C 1.90398100 -3.59818700 -1.83197700

C 3.45376000 -4.76282900 0.16892000

H 2.96844200 -3.02625800 1.34073900

C 2.52496600 -4.82629900 -2.05581000

H 1.30143500 -3.14274400 -2.61124700

C 3.30147300 -5.41650200 -1.05586300

H 4.06071300 -5.20865900 0.95333400

H 2.40363300 -5.32175200 -3.01576500

H 3.78701800 -6.37296700 -1.23117800

C -0.85332600 -2.65156600 -0.90603000

C -1.59568700 -2.54420600 -2.09428500

C -0.99396500 -3.82208000 -0.13743800

C -2.42948100 -3.58180900 -2.51268500

H -1.51264300 -1.64343700 -2.69306800

C -1.82491800 -4.85955600 -0.55665700

H -0.42475900 -3.91521200 0.78275800

C -2.54655000 -4.74673700 -1.75039900

H -2.99004400 -3.47195800 -3.43740000

H -1.90582100 -5.76102300 0.04621800

H -3.19323600 -5.55642700 -2.07871500

H 7.05602500 4.20441400 0.01732600

H -3.32619300 7.48937500 -0.57567000

C -1.74831800 -1.20562000 1.84399100

C -1.81781300 -2.57226800 3.66802300

C -3.04199600 -2.60701800 3.08911400

H -1.43196200 -3.04161400 4.55877200

H -3.94653300 -3.12965500 3.35620500

N -2.98274000 -1.75925100 1.98716000

N -1.04200600 -1.70401500 2.89943400

C -4.11107700 -1.45511800 1.15076100

C -4.52956600 -2.38043200 0.19684300

C -4.77118100 -0.23835600 1.33222700

C -5.65392500 -2.09038500 -0.57811600

H -3.96553600 -3.29490400 0.05102000

C -5.89835900 0.03609300 0.55737500

H -4.37680400 0.48166600 2.04169400

C -6.34476200 -0.89091800 -0.39039000

H -5.97907900 -2.79972100 -1.33355400

H -6.41846000 0.98116900 0.68358000

H -7.22077800 -0.66889300 -0.99392000

C 0.27697000 -1.29347300 3.26098000

C 0.53671100 0.06699800 3.43964300

C 1.26899500 -2.25274400 3.47327800

C 1.81352200 0.46793500 3.83508700

H -0.25002900 0.79179700 3.27218900

C 2.53812700 -1.84180300 3.88419200

H 1.05126600 -3.30163000 3.29641600

C 2.81054400 -0.48318200 4.06925700

H 2.01943400 1.52640100 3.96253800

H 3.31645700 -2.58189500 4.04588400

H 3.80051700 -0.16858500 4.38701600

**TS5-IPr-TPS**

Ni -0.99348000 -1.20853600 -0.19538500

C 0.69358200 -2.08444900 -0.76613200

S -1.96328000 1.53459200 0.62913000

O -0.54792600 2.00697100 0.72730400

C 1.92705000 -1.81340300 -0.27411300

C 2.16063800 -0.54029400 0.57449700

H 1.41281500 -0.56018700 1.37465700

C 3.52451700 -0.34352300 1.23455700

C 3.56981900 0.03562500 2.58052600

C 4.73143100 -0.40944700 0.52398800

C 4.77982200 0.33406800 3.20991000

H 2.64563400 0.10502200 3.14052000

C 5.94154200 -0.10639500 1.14544400

H 4.72619600 -0.70257600 -0.51745100

C 5.97357800 0.26432200 2.49153100

H 4.78499400 0.62654200 4.25705300

H 6.86223400 -0.15372000 0.57078200

H 6.92007200 0.49614300 2.97305500

S 2.46968700 1.25226800 -1.55563100

C 3.90479100 2.26603700 -1.06924900

C 5.20530700 2.02677100 -1.59384500

C 3.67065300 3.27831700 -0.10983100

C 6.25668400 2.76540000 -1.04557400

C 4.78106400 3.96198000 0.39929600

C 6.07804400 3.70605600 -0.02907500

H 7.26078600 2.58758400 -1.40977200

H 4.62089200 4.71956000 1.16129000

O 2.90883400 0.08040800 -2.32386900

O 1.47828000 2.16117100 -2.16745200

N 1.78369800 0.68580400 -0.16895300

H 1.00227700 1.23418000 0.19313000

C -2.93829800 3.05084300 0.25536500

C -3.73379600 3.25942400 -0.89715700

C -2.87316600 4.02919900 1.28530900

C -4.42750200 4.47261200 -0.99640300

C -3.61788600 5.20079800 1.11977000

C -4.39332800 5.45276800 -0.00954300

H -5.02126100 4.64627800 -1.88794000

H -3.59302400 5.94777700 1.90771200

O -2.56144800 0.97771400 1.85695900

N -2.12519200 0.37719800 -0.54800700

C -1.12105400 0.36091500 -1.53784200

H -0.39396900 1.16933200 -1.52540700

H -0.00292100 -0.67023600 -1.26614300

C -1.53288200 -0.03727500 -2.91542000

C -0.72548200 0.33762200 -3.99907700

C -2.70453600 -0.76553800 -3.14653200

C -1.08853700 -0.02305100 -5.29711800

H 0.17912700 0.91064200 -3.81650000

C -3.06703400 -1.12056800 -4.44450700

H -3.32953100 -1.03629700 -2.30592300

C -2.25784000 -0.75494200 -5.52443300

H -0.45972100 0.27312800 -6.13248500

H -3.98226400 -1.68364700 -4.60694100

H -2.53947800 -1.03290900 -6.53668400

C 3.08887300 -2.71494100 -0.54067300

C 3.71002600 -3.38300400 0.52610300

C 3.59476000 -2.90597700 -1.83569300

C 4.79816600 -4.22818900 0.30706400

H 3.34283200 -3.22268000 1.53474800

C 4.68625200 -3.74676000 -2.05419200

H 3.13499800 -2.37806900 -2.66125200

C 5.29172400 -4.41338800 -0.98597500

H 5.26333300 -4.73555800 1.14876200

H 5.06532600 -3.87963900 -3.06442100

H 6.14204300 -5.06798300 -1.15949100

C 0.52142200 -3.17411900 -1.76845300

C 0.41141600 -2.86510100 -3.13396100

C 0.57208700 -4.52685200 -1.40514600

C 0.39946100 -3.87158200 -4.09960200

H 0.38643900 -1.82827800 -3.44457700

C 0.57358400 -5.53630200 -2.36723500

H 0.65776600 -4.77712400 -0.36035600

C 0.49568600 -5.21351700 -3.72437000

H 0.32819800 -3.59893600 -5.14947200

H 0.64575000 -6.57538000 -2.05423400

H 0.49989000 -5.99709800 -4.47746800

C -1.85391200 -2.24793700 1.19422500

C -2.50512200 -3.25252800 3.14176000

C -3.60206000 -3.14556300 2.36463100

H -2.36844900 -3.63711500 4.13815200

H -4.62018200 -3.45811600 2.51945900

N -3.19959700 -2.53189100 1.18382900

N -1.44141200 -2.72916900 2.41066400

C -4.16792800 -2.30906200 0.13091200

C -4.12245300 -3.12921200 -1.01877500

C -5.19857100 -1.37324600 0.35547400

C -5.14819600 -2.97061900 -1.95928400

C -6.18885200 -1.25270400 -0.62890900

C -6.16695300 -2.03939700 -1.77343400

H -5.14810700 -3.57476100 -2.85819100

H -6.99135600 -0.53574300 -0.48861200

H -6.94634400 -1.93158700 -2.52325500

C -0.12598800 -2.71567300 3.01245500

C 0.22231400 -1.60886900 3.81178200

C 0.66080000 -3.88685800 2.95141000

C 1.42191400 -1.68275900 4.53048400

C 1.86303800 -3.89060000 3.67018800

C 2.24459200 -2.80058500 4.44789000

H 1.70973900 -0.85388600 5.16928400

H 2.50447000 -4.76412800 3.63225900

H 3.18092400 -2.82786300 4.99813000

C 0.20663900 -5.14780400 2.21953500

H -0.44064600 -4.83882900 1.39141200

C -0.70813600 -0.41768200 3.99440700

H -1.45236300 -0.42631100 3.19869200

C 1.37520100 -5.97308000 1.64681500

H 1.92527500 -6.48672100 2.44409500

H 0.98476000 -6.74915500 0.97869900

H 2.08747900 -5.36345200 1.08586300

C -0.62208600 -6.06370000 3.14744900

H -0.02971600 -6.35654200 4.02263000

H -1.53692800 -5.58366600 3.49934100

H -0.91090700 -6.97564100 2.61171700

C 0.01126900 0.93505200 3.89365600

H 0.43848400 1.08560600 2.89996500

H -0.71080600 1.73971000 4.05608900

H 0.80500600 1.04829600 4.64165700

C -1.45661600 -0.53487000 5.33754800

H -0.75908900 -0.50000100 6.18374800

H -2.16734100 0.29275300 5.44408700

H -2.01838900 -1.47332500 5.40401800

C -3.00147300 -4.13970900 -1.25027800

H -2.05730000 -3.63624100 -1.01958600

C -5.31341300 -0.53198600 1.62292300

H -4.35380800 -0.55532700 2.14144700

C -5.61560200 0.94444100 1.31663000

H -5.48878600 1.54708800 2.22174100

H -4.93702400 1.33735500 0.56230500

H -6.64377600 1.08749600 0.96269800

C -6.40769900 -1.08942100 2.55719300

H -7.38628900 -1.06499100 2.06264100

H -6.21948100 -2.12409500 2.85984200

H -6.47353200 -0.48093800 3.46685300

C -3.11287200 -5.36376600 -0.31739200

H -3.10587500 -5.08946300 0.74070900

H -4.03662400 -5.92045900 -0.51842500

H -2.26501100 -6.03563200 -0.49457700

C -2.90437800 -4.61335000 -2.70599600

H -2.80431500 -3.77197900 -3.39777300

H -2.02003800 -5.24108600 -2.82206400

H -3.77750400 -5.21052600 -2.99782200

C -2.09742300 3.89448000 2.60045400

H -1.37729600 3.08774900 2.51214000

C -3.88989300 2.29902400 -2.07277900

H -3.49929500 1.33249400 -1.77726500

C -1.27635600 5.15370000 2.93396400

H -0.64221800 5.44695900 2.09106700

H -1.90503000 6.01069800 3.20088000

H -0.62586000 4.94837100 3.79222000

C -3.05677600 3.53236400 3.74906000

H -3.81576700 4.31107600 3.89307600

H -3.55948700 2.58488300 3.53786300

H -2.50051600 3.42589200 4.68905600

C -5.17575900 6.74817500 -0.15344300

H -4.98268700 7.35035600 0.74487000

C -6.69151500 6.48437800 -0.22272800

H -6.94852900 5.89678300 -1.11206800

H -7.03609000 5.92826200 0.65600900

H -7.24646400 7.42873400 -0.27320100

C -4.69903300 7.56026400 -1.37191700

H -5.23360700 8.51565700 -1.43266000

H -3.62532900 7.76868300 -1.31224800

H -4.88208600 7.01423800 -2.30494000

C -3.07817700 2.80911800 -3.27812100

H -3.45247800 3.78358000 -3.61645100

H -2.01940300 2.92700400 -3.02418800

H -3.14678000 2.10247600 -4.11155700

C -5.35836700 2.05189400 -2.46514700

H -5.39393100 1.25827000 -3.22033000

H -5.95006000 1.72490200 -1.60425900

H -5.83956200 2.93818200 -2.89493600

C 2.30063300 3.71609700 0.41603400

H 1.50971800 3.16893600 -0.08468000

C 5.51587300 1.06858300 -2.74841500

H 4.95764900 0.14800300 -2.59075800

C 7.26524600 4.41140600 0.60471500

H 6.86840500 5.13709900 1.32785900

C 8.13648900 3.40626500 1.38287500

H 8.57781000 2.66650700 0.70408000

H 8.95540600 3.91944900 1.90127600

H 7.53955500 2.86218600 2.12261000

C 8.10126100 5.19072200 -0.42581100

H 7.48414100 5.91439000 -0.96946900

H 8.91402700 5.73467500 0.07004000

H 8.55461600 4.51688700 -1.16232200

C 2.16327800 3.45249100 1.92491700

H 2.83986600 4.08646800 2.51052300

H 1.13703000 3.65448700 2.24585300

H 2.39724800 2.41088100 2.15873800

C 2.03283500 5.19373500 0.07079500

H 2.10941700 5.35888600 -1.00911400

H 1.01838600 5.46584000 0.38489700

H 2.73359100 5.87123300 0.57228600

C 5.02052200 1.67925500 -4.07598600

H 5.19396600 0.98149600 -4.90383500

H 3.95030100 1.89973500 -4.04115000

H 5.55622200 2.61156000 -4.29489700

C 6.99546600 0.67162400 -2.86715900

H 7.09392000 -0.11167500 -3.62684000

H 7.63487000 1.50633000 -3.17919600

H 7.38685700 0.27297400 -1.92377200

**TS5-IPr-Ts**

Ni 1.02772400 -0.13929700 0.04889900

C -0.39136300 -1.12369600 1.00237300

S 1.14102800 2.56828400 -1.53756400

O -0.33615300 2.42815800 -1.68664200

C -1.69227800 -1.21252400 0.63390900

C -2.27519100 -0.24277800 -0.41831800

H -1.62174700 -0.29848900 -1.29524200

C -3.70821600 -0.45925000 -0.90090900

C -3.97612300 -0.36106800 -2.27065100

C -4.79167700 -0.60830000 -0.02219200

C -5.28254900 -0.42507200 -2.75985700

H -3.15324600 -0.22166500 -2.96254000

C -6.09744700 -0.67004400 -0.50504300

H -4.61668100 -0.66439700 1.04464400

C -6.35020100 -0.58222600 -1.87592800

H -5.46227600 -0.34637400 -3.82924900

H -6.91978800 -0.77682700 0.19682900

H -7.36992200 -0.63117200 -2.24957100

S -2.75923900 1.91972500 1.29765200

C -4.44237400 2.35092600 0.85540900

C -5.45315500 2.20716400 1.80309100

C -4.72266300 2.79937700 -0.43655400

C -6.76725300 2.50275600 1.44126300

H -5.21225700 1.83898200 2.79448300

C -6.03904800 3.08203700 -0.78250900

H -3.92798600 2.88474400 -1.17045100

C -7.08136900 2.92959800 0.14509900

H -7.56234700 2.38239600 2.17303900

H -6.26667600 3.40735200 -1.79450700

O -2.84295700 0.99139000 2.43525400

O -1.98796200 3.17214800 1.43630300

N -2.10448300 1.16737100 0.00181200

H -1.53501700 1.76642300 -0.60211400

C 1.41546400 4.28023300 -1.07635300

C 0.49730500 4.92226700 -0.23951700

C 2.54288400 4.94578000 -1.55631900

C 0.74137500 6.24098000 0.13662800

H -0.39714400 4.41381200 0.10525600

C 2.76315900 6.26824600 -1.17382000

H 3.22024500 4.43921700 -2.23327600

C 1.87587900 6.93123700 -0.31544800

H 0.03143400 6.74454900 0.78811900

H 3.63655700 6.79422700 -1.55185200

O 1.95626900 2.26022700 -2.72139700

N 1.75190300 1.69964900 -0.25359300

C 0.94096800 1.72678400 0.89430500

H 0.03882700 2.33330100 0.87125300

H 0.03396600 0.47927500 1.06281800

C 1.63254100 1.77938600 2.21484800

C 0.86021800 1.99858200 3.36650500

C 3.02040300 1.64047200 2.32756000

C 1.46973900 2.05522700 4.61829200

H -0.21559700 2.12586800 3.27141200

C 3.62650500 1.70444400 3.58290800

H 3.61150100 1.49344500 1.43164600

C 2.85551400 1.90413700 4.73048700

H 0.86477600 2.22354700 5.50537300

H 4.70410700 1.59135800 3.65812600

H 3.33151500 1.94982000 5.70661900

C -2.59581700 -2.25305500 1.21477200

C -3.09592900 -3.27448400 0.39309400

C -2.98891800 -2.22427700 2.56161000

C -3.94534100 -4.25510000 0.90573900

H -2.82185500 -3.29102000 -0.65657700

C -3.83879400 -3.20458900 3.07444000

H -2.64421000 -1.41374200 3.19065900

C -4.31744200 -4.22673200 2.25110600

H -4.31719900 -5.03979700 0.25130700

H -4.13153100 -3.16545500 4.12072000

H -4.97919500 -4.98983400 2.65296900

C 0.09510900 -1.94607300 2.14177900

C 0.05829300 -1.44262200 3.45160800

C 0.50428800 -3.27269900 1.95231000

C 0.34202100 -2.26792200 4.53983200

H -0.24390000 -0.41341900 3.61267500

C 0.77940600 -4.10358500 3.03773500

H 0.58062900 -3.64861600 0.94147700

C 0.68093400 -3.60890500 4.34101400

H 0.28465000 -1.86365800 5.54730100

H 1.06050100 -5.13957000 2.86420900

H 0.88469300 -4.25562000 5.19035900

C 2.00297800 -1.47774200 -0.93442500

C 2.73661500 -3.19865300 -2.24605400

C 3.81586400 -2.68656000 -1.61880700

H 2.64080200 -3.99241500 -2.96610400

H 4.85791800 -2.95824600 -1.64984900

N 3.36262000 -1.63220000 -0.83191100

N 1.63274700 -2.46368800 -1.81487000

C 4.32208900 -0.84301400 -0.08316700

C 4.62326600 -1.22097300 1.24001700

C 5.01043300 0.18588500 -0.75789100

C 5.68808400 -0.56651100 1.87124100

C 6.05679000 0.81446500 -0.06971200

C 6.40454000 0.43327100 1.22232900

H 5.94891800 -0.83144500 2.88908300

H 6.60806000 1.61434700 -0.55100300

H 7.22726500 0.92944400 1.73093300

C 0.32446200 -2.72134100 -2.37559800

C -0.23122000 -1.76968800 -3.26451700

C -0.28037700 -3.97388900 -2.13659000

C -1.45030200 -2.09463000 -3.86789000

C -1.50489700 -4.23520400 -2.76854400

C -2.09236300 -3.30472700 -3.61451700

H -1.90424800 -1.39505000 -4.55886100

H -1.99759400 -5.18660900 -2.59452500

H -3.04625300 -3.52141300 -4.08708500

C 0.48599900 -0.46666600 -3.61092200

H 0.86339800 -0.03764100 -2.68167700

C -0.41678000 0.59448700 -4.25339100

H -1.28906400 0.82019100 -3.63330700

H 0.15136100 1.52120300 -4.35267300

H -0.75660600 0.29129500 -5.25164400

C 1.70806400 -0.71306500 -4.52013300

H 1.39346200 -1.11454000 -5.49158900

H 2.22723100 0.23662000 -4.68527400

H 2.42386500 -1.41199300 -4.07932600

C 0.35654400 -5.08714300 -1.30551400

H 1.20194300 -4.66840200 -0.75089500

C -0.61217600 -5.71173700 -0.28229800

H -1.40907500 -6.27785000 -0.77774900

H -0.06687800 -6.41322200 0.36003800

H -1.08545100 -4.96175800 0.35276300

C 0.90311100 -6.20016600 -2.22630200

H 0.08512200 -6.67463400 -2.78062700

H 1.61878000 -5.82289400 -2.96361200

H 1.40304200 -6.97437300 -1.63268500

C 3.82157700 -2.28597000 1.97520800

H 2.78766300 -2.21011200 1.62890000

C 4.31990400 -3.70956500 1.65713400

H 4.26756700 -3.93727400 0.58838400

H 5.35972900 -3.83759700 1.98300700

H 3.70179000 -4.44329800 2.18709200

C 3.80054100 -2.06947200 3.49602800

H 3.48065800 -1.05525900 3.75087800

H 3.09852300 -2.76977400 3.95075700

H 4.78481800 -2.24968900 3.94611000

C 4.65401700 0.62361000 -2.17376800

H 3.58323900 0.45651700 -2.31566900

C 4.90249300 2.12447600 -2.39352600

H 4.42291900 2.43937700 -3.32351900

H 4.47513200 2.71148900 -1.57728200

H 5.97282500 2.35361700 -2.46929100

C 5.42777600 -0.17750900 -3.24123500

H 6.50967400 -0.04895600 -3.11138100

H 5.20555200 -1.24672800 -3.20520400

H 5.16068100 0.18177300 -4.24198700

C -8.51153800 3.17954600 -0.26881300

H -8.59862600 4.07805000 -0.89013200

H -8.89441000 2.33782300 -0.86103100

H -9.16887700 3.30008800 0.59825500

C 2.14107900 8.34991000 0.12928400

H 2.77518500 8.88458900 -0.58569700

H 1.20943200 8.91382800 0.24769000

H 2.65513800 8.36479600 1.09956800

**TS5-PCy_3_**

Ni -0.00954800 -0.77826500 -0.21321600

C 1.34178100 0.38215000 -1.08613100

P 1.04849800 -2.38695900 0.92196300

C 2.91074300 -2.59070400 0.84988900

C 3.60778400 -1.29475100 1.31046600

C 5.13833100 -1.41352800 1.27002700

C 5.62877500 -1.83247300 -0.12054500

C 4.96140000 -3.14320500 -0.55848500

C 3.42665000 -3.04498000 -0.53125600

C 0.40700400 -4.02820000 0.24628500

C 0.18793200 -3.90277900 -1.27888500

C -0.30923400 -5.21252400 -1.90457700

C -1.59735600 -5.69410100 -1.22460100

C -1.39385000 -5.82477800 0.29021100

C -0.89319400 -4.51434700 0.92162900

C 0.60648400 -2.39899600 2.73765200

C 0.62189000 -1.00376900 3.39299700

C 0.01543200 -1.07146600 4.80269000

C 0.74709200 -2.09990700 5.67828200

C 0.77206100 -3.48494900 5.01329900

C 1.37691200 -3.41574400 3.60017100

H 3.15077400 -3.38743500 1.56911400

H 3.28532800 -0.46584000 0.66836600

H 3.29358600 -1.04904000 2.32981000

H 5.58593400 -0.45627000 1.56675900

H 5.46433300 -2.15934200 2.01068500

H 5.38144400 -1.04458800 -0.84357500

H 6.72087600 -1.94016700 -0.12557900

H 5.28487800 -3.41721300 -1.57045900

H 5.28669000 -3.95417000 0.11081200

H 3.09920700 -2.33982500 -1.29955300

H 3.00396100 -4.02205700 -0.78910900

H 1.19374900 -4.77475900 0.43369500

H -0.56326500 -3.12477200 -1.45058300

H 1.09371500 -3.56407100 -1.78715400

H -0.47380600 -5.06217100 -2.97934900

H 0.46701400 -5.98638000 -1.80758900

H -2.39942500 -4.96674200 -1.41948400

H -1.92678800 -6.65017900 -1.65115700

H -2.32783100 -6.13077900 0.77884100

H -0.66146900 -6.62257700 0.48602600

H -1.66126700 -3.73695300 0.82986500

H -0.73623000 -4.67728100 1.99283500

H -0.44733800 -2.69156600 2.71218700

H 1.64791200 -0.62286700 3.46322900

H 0.05280800 -0.29511500 2.78495900

H 0.05146700 -0.07834400 5.26787300

H -1.04386800 -1.34417700 4.71013700

H 1.78217200 -1.76364900 5.84329900

H 0.27652700 -2.16391400 6.66760000

H 1.33873400 -4.19609700 5.62825600

H -0.25465500 -3.87231800 4.94326300

H 2.43050800 -3.11237100 3.67884400

H 1.36821500 -4.41105700 3.13659500

S -2.59709000 -0.61442700 1.45802900

O -2.10567600 0.73257900 1.86016800

C 1.72010100 1.56366400 -0.52305600

C 1.06435400 1.98883800 0.80628800

H 0.84194700 1.05604400 1.33124900

C 1.95880800 2.80244300 1.72829400

C 2.79036000 2.11522100 2.62150300

C 2.01812800 4.20020100 1.69034900

C 3.67764300 2.80207900 3.45110400

H 2.75224100 1.03247100 2.65650100

C 2.90034000 4.88877800 2.52436000

H 1.38955700 4.74480300 0.99637600

C 3.73611600 4.19610300 3.40339500

H 4.31478800 2.24885600 4.13655900

H 2.93999600 5.97407100 2.47865900

H 4.42356200 4.73765600 4.04804800

S -0.79602600 3.50686700 -0.62797700

C -2.45640100 3.88713300 -0.04761900

C -2.65538200 4.35156500 1.25565900

C -3.51477400 3.75608900 -0.94813500

C -3.94852100 4.67860500 1.66273400

H -1.81397400 4.43324000 1.93521100

C -4.80143100 4.10468300 -0.53364300

H -3.32566800 3.37993100 -1.94751200

C -5.01790300 4.56206400 0.76830600

H -4.12100500 5.02688200 2.67677400

H -5.63307500 4.00777200 -1.22540700

O 0.02389800 4.72119400 -0.71070100

O -0.96328700 2.72815400 -1.87438100

N -0.29272100 2.60050900 0.67465000

H -1.02626900 1.94901600 0.97172200

C -4.32324400 -0.41485400 1.01465000

C -4.86982700 0.86467800 0.92683400

C -5.07373100 -1.55924900 0.73138600

C -6.20624300 0.99654100 0.54336300

H -4.26428400 1.73289700 1.16004400

C -6.40521900 -1.41207900 0.34843900

H -4.61855900 -2.54053800 0.81822200

C -6.96987400 -0.13534700 0.25334100

H -6.64293400 1.98783300 0.47221700

H -7.00310800 -2.29103100 0.12514700

O -2.48506900 -1.71137400 2.42931200

N -1.92256100 -1.14331900 0.03430300

C -1.83139100 -0.18225500 -0.98007000

H -2.15591600 0.83579300 -0.76507000

H -0.29902800 0.38478300 -1.18342000

C -2.17228100 -0.65424900 -2.35698400

C -2.01873200 0.20300300 -3.45814000

C -2.64963300 -1.95596000 -2.56410000

C -2.31136800 -0.24962700 -4.74316700

H -1.63801300 1.20552000 -3.29981800

C -2.93576300 -2.40706200 -3.85287900

H -2.79651200 -2.59920100 -1.70439300

C -2.76152000 -1.55789100 -4.94768600

H -2.17749400 0.41842400 -5.58971200

H -3.29875500 -3.42125300 -3.99852900

H -2.98091900 -1.90909400 -5.95248200

C 2.67652700 2.50611500 -1.17860000

C 3.92942500 2.81925500 -0.62434500

C 2.32709400 3.07708400 -2.41331300

C 4.80998200 3.66989100 -1.29280800

H 4.22097700 2.38437600 0.32545700

C 3.20493800 3.93339100 -3.07581400

H 1.35487900 2.84978500 -2.83601100

C 4.45115600 4.23079700 -2.52051100

H 5.77771300 3.89413500 -0.85130500

H 2.91232600 4.37080000 -4.02677100

H 5.13677200 4.89658800 -3.03847000

C 1.96272800 -0.22252600 -2.29077500

C 1.20979600 -1.05935100 -3.13608600

C 3.33991000 -0.09693600 -2.57461300

C 1.79051200 -1.72685600 -4.21408000

H 0.15589100 -1.20504700 -2.94314700

C 3.92442700 -0.77182100 -3.64365000

H 3.96116800 0.52282300 -1.94175200

C 3.15499300 -1.59217400 -4.47354100

H 1.16898500 -2.35902900 -4.84310100

H 4.99059200 -0.65925900 -3.82372800

H 3.61375100 -2.11952100 -5.30567000

H -6.02172700 4.82572000 1.09022200

H -8.00842700 -0.02571400 -0.04668000
